# A binuclear guanidinate yttrium carbyne complex: unique reactivity toward unsaturated C–N, C–O and C–S bonds†

**DOI:** 10.1039/d3sc03483f

**Published:** 2023-08-03

**Authors:** Wen Jiang, Feng Kong, Iker del Rosal, Meng Li, Kai Wang, Laurent Maron, Lixin Zhang

**Affiliations:** a Department of Chemistry, Shanghai Key Laboratory of Molecular Catalysis and Innovative Materials, Fudan University 2005 Songhu Road, Jiangwan Campus Shanghai 200438 P. R. China lixinzh@fudan.edu.cn; b LPCNO, Université de Toulouse 31077 Toulouse France maron@irsamc.ups-tlse.fr

## Abstract

A guanidinato-stabilized binuclear yttrium carbyne complex [(PhCH_2_)_2_NC(NC_6_H_3_^i^Pr_2_-2,6)_2_]_2_Y_2_(μ_2_-Me)(AlMe_3_)_2_(μ_4_-CH) (1) was synthesized *via* C–H bond activation and its versatile reactivities were investigated. Complex 1 underwent σ-bond metathesis with PhSSPh and nucleophilic addition with PhCN to form the corresponding yttrium thiolate complex 3 and aza–allyl complex 4 respectively. Additionally, the rare yttrium carbide complex 5 was also prepared by treatment of complex 1 with S_8_. Interestingly, in the reaction with PhNCS, the C=S double bond was cleaved, followed by C–H bond activation to give the yttrium sulfide complex 7 with a ketenimine dianion ligand. Unexpectedly, the reaction of complex 1 with CO (1 atm) resulted in deoxygenative coupling of CO, to afford mono- or dioxo-yttrium complexes at different temperatures. The mechanism of the possible formation processes of complexes 3 and 9 was elucidated by DFT calculations.

## Abstract 2

We describe the synthesis and versatile reactivity of a guanidinato-stabilized binuclear yttrium carbyne complex. The rare-earth-metal carbyne complex induced deoxygenative coupling reaction of CO was presented firstly, and a possible reaction mechanism was elucidated by DFT calculations.

## Graphical abstract

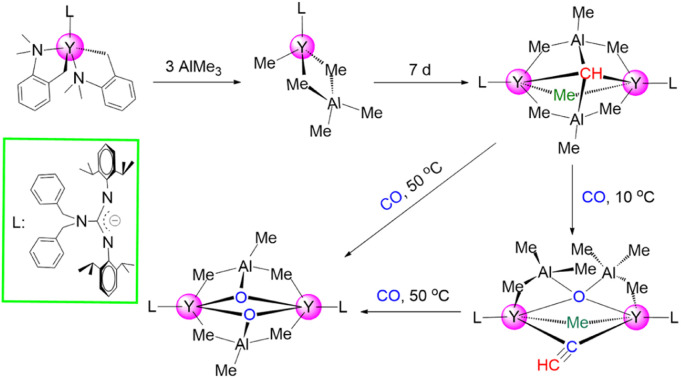

## Introduction

Since the first typical metal carbyne M≡CR (M = Cr, Mo, W) was reported,^1^ transition-metal alkylidyne complexes have been extensively investigated because of their unique structural features and great potential in alkyne metathesis and stoichiometric reactions.^2–4^ In stark contrast, the studies on the well-defined rare-earth-metal alkylidyne complexes are relatively limited.^5–8^ This might be because the empty orbitals of rare-earth-metals are very high-energy d-orbitals and highly contracted f-orbitals, preventing the formation of strong covalent bonds with carbon atom orbitals. To date, four types of rare-earth-metal carbyne complexes have been successfully isolated and fully characterized. Firstly, Anwander *et al.* reported successfully a pentanuclear carbyne complex La_5_Al_9_(CH)_6_(CH_3_)_30_ (A) and tetranuclear carbyne complexes [{(C_5_Me_5_)Y(μ_2_-Me)_2_AlMe}_2_(μ_2_-Me)(μ_4_-CH)]_2_ (B), La_4_Al_8_(CH)_4_ (CH_2_)_2_(CH_3_)_20_(PMe_3_) (C), and La_4_Al_8_(C)(CH)_2_(CH_2_)_2_(CH_3_)_22_(toluene) (D) *via* donor-induced alkylaluminate cleavage of heterobimetallic Ln–(CH_3_)–Al linkages.^6^ Subsequently, Mitzel and co-workers isolated the mononuclear Sm derivative [(TCyTAC)Sm^III^(μ_4_-CH)(AlMe_3_)_3_]^7*a*,*b*^ (TCyTAC = 1,3,5-tricyclohexyl-1,3,5-triazacyclo-hexane) (F) and Pr derivative (η^3^-TMTAC)Pr(η^4^-{CH(AlMe_3_)_3_})^7*c*^ (TMTAC = 1,3,5-trimethyl-1,3,5-triazacyclohexane) (G) through bulky base cyclic triamine initiated multiple C–H activation.

More recently, Cheng *et al.* discussed a rigid trinuclear scandium carbyne complex [(C_5_Me_5_)Sc(μ_2_-Br)]_3_(μ_3_-CH) (E) *via* methyl/halogenido exchange protocol of [(C_5_Me_5_)Sc(Me_2_)]_2_ with ^*t*^BuBr.^8^ However, a significant knowledge gap in the area involving binuclear rare-earth-metal alkylidyne complexes was incontrovertible. Additionally, compared to transition-metal alkylidyne complexes, the reactivity exploration of rare-earth-metal alkylidyne complexes remains scarce.^8^

In the last few decades, investigations on the synthesis and reactivity of rare-earth-metal alkylidene complexes have been documented widely.^9–15^ Our previous studies on amidinate rare-earth-metal carbene derivatives have also shown the unique reactivity of μ_3_-CH_2_ dianions.^16,17^ The results indicate that there are significant ligand effects on the structure and reactivity of these complexes. However, attempts to prepare guanidinate rare-earth-metal methylidene complexes by a similar synthetic process for the synthesis of amidinate methylidene complexes were unsuccessful, and an unexpected binuclear carbyne complex was isolated. Interestingly, this complex shows high and intriguing reactivity under mild conditions, which is distinct from those already reported for other transition-metal carbyne complexes.

## Results and discussion

### Synthesis and structural characterization

**Scheme 1:**
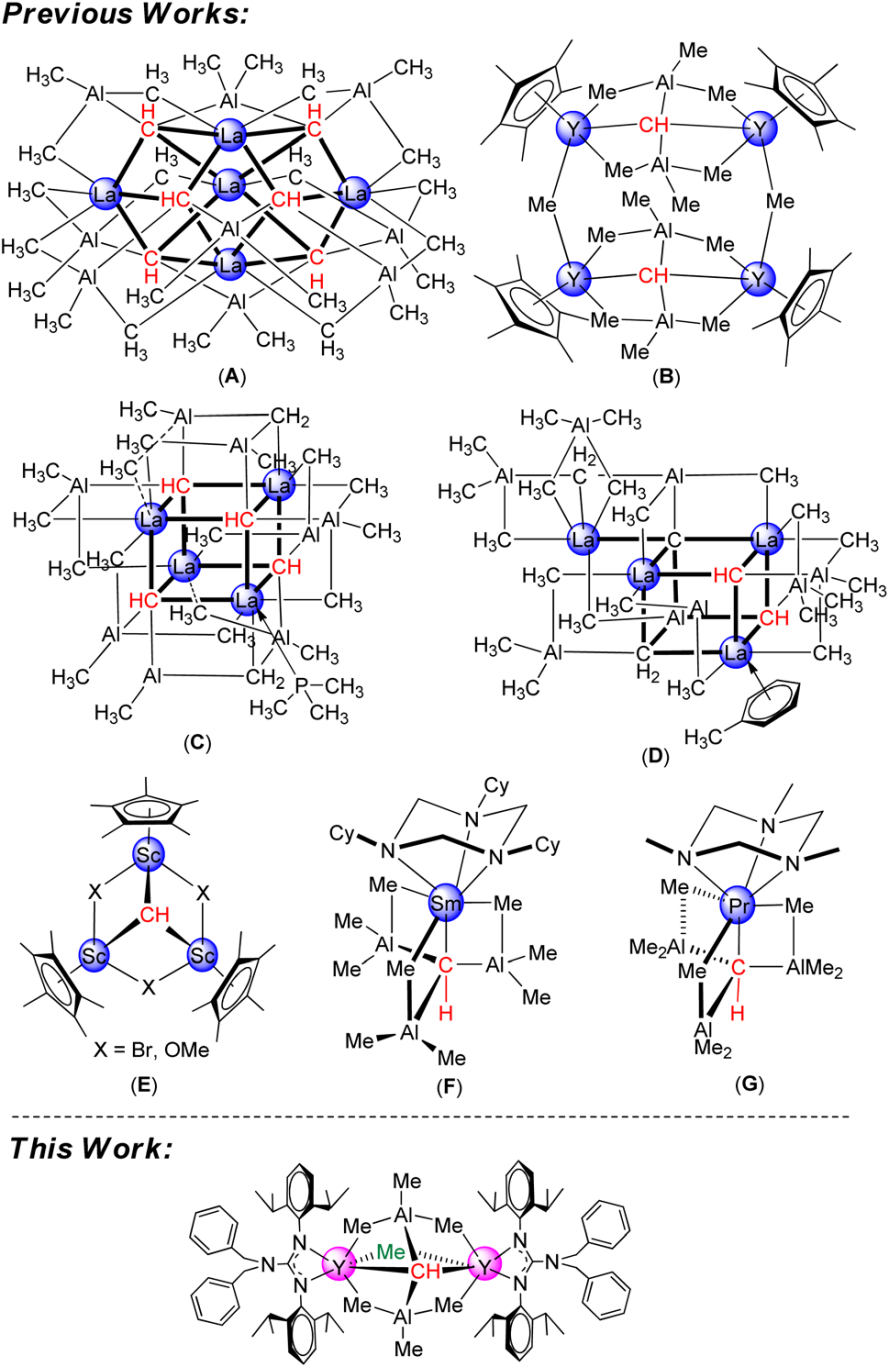
Rare-earth-metal carbyne complexes.

The first binuclear yttrium carbyne complex [(PhCH_2_)_2_NC(NC_6_H_3_^i^Pr_2_-2,6)_2_]_2_Y_2_(μ_2_-Me)(AlMe_3_)_2_(μ_4_-CH) (1) was obtained in 67% yield *via* a metal-assisted multiple C–H bond activation process when three equivalents of AlMe_3_ were added into a stirred toluene solution of the guanidinate yttrium bis(*o*-aminobenzyl) complex^18^ at 60 °C for 18 h (Scheme 1). In order to get more insights into the formation process of the yttrium carbyne complex, the same reaction was carried out at room temperature for 6 h which gave a heterobimetallic Y/Al complex (PhCH_2_)_2_NC(NC_6_H_3_^i^Pr_2_-2,6)_2_Y(Me)(AlMe_4_) (2) in 53% yield. It is interesting that complex 2 could turn into complex 1 in high yield in toluene at room temperature for 7 days with the release of CH_4_ (ESI Fig. S6†). This is sufficient to show that complex 2 is an intermediate in the formation of complex 1.

**Fig. 1:**
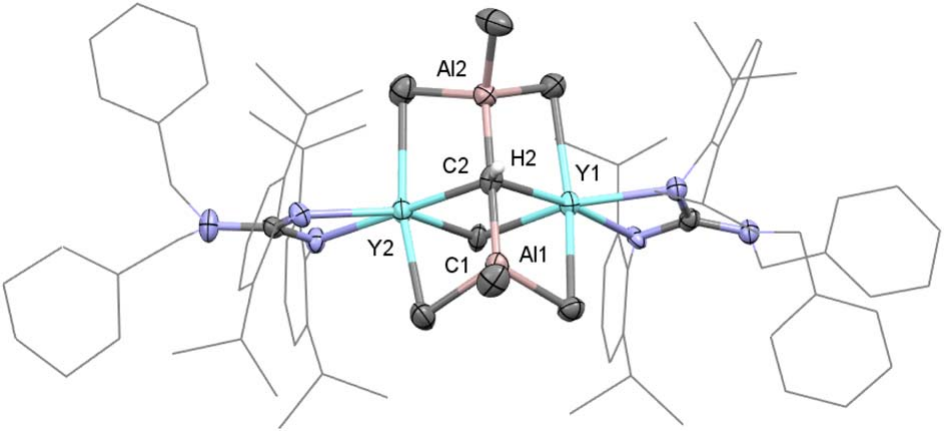
Molecular structure of complex 1 with thermal ellipsoids at 30% probability except for the 2,6-(^i^Pr)_2_C_6_H_3_ groups and benzyl groups in the guanidinate ligand. All of the hydrogen atoms (except for H2) are omitted for clarity. Selected bond distances (Å) and angles (°): Y(1)–C(1) 2.528(6), Y(1)–C(2) 2.418(5), Y(2)–C(1) 2.543(5), Y(2)–C(2) 2.398(5), C(2)–Al(1) 1.974(5), and C(2)–Al(2) 1.970(5); C(1)–Y(1)–C(2) 85.47(15), C(1)–Y(2)–C(2) 85.55(15), Y(1)–C(1)–Y(2) 91.29(17), and Y(1)–C(2)–Y(2) 97.65(16).

The single crystal X-ray diffraction analysis of complex 1 shows that two distorted octahedral yttrium centers are linked by μ_4_-CH and μ_2_-Me units to form a distorted square while two AlMe_3_ groups work as bulky ligands to stabilize this binuclear carbyne complex (Fig. 1). The average Y–C1(μ_2_-Me) bond length (2.535(5) Å) is similar to that in [(η-C_5_H_5_)_2_YMe]_2_ (2.537(9) Å).^19*a*^ It is noticeable that the Y–C2(μ_4_-CH) bond lengths of 2.398(5) Å and 2.418(5) Å are shorter than those in [{(C_5_Me_5_)Y(μ_2_-Me)_2_AlMe}_2_(μ_2_-Me)(μ_4_-CH)]_2_ (2.444(3)–2.464(3) Å). A similar trend is observed for the Al–C2(μ_4_-CH) bond lengths (1.970(5) Å and 1.974(5) Å) *versus* 1.984(3)–1.993(3) Å for Al–C(μ_4_-CH),^6*a*^ probably due to the steric hindrance. In the ^1^H NMR spectrum, the resonance of the [C*H*]^3−^ group is observed as a singlet at *δ* = 2.36 (benzene-*d*_6_) ppm, which is different to the proton signal at *δ* = 12.16 ppm in [(C_5_Me_5_)Sc(μ_2_-Br)]_3_(μ_3_-CH).^8^ The metal-bonded Y_2_*C*HAl_2_ methine carbon atom is shown at *δ* = 90.2 ppm based on a comprehensive analysis of the ^1^H, ^13^C{^1^H} and two-dimensional HMQC NMR spectra (Fig. S1–S3†). The bridged μ_2_-Me(Y–Me–Y) and AlMe_3_ are shown as two singlets at *δ* = −0.26 and 0.34 ppm, respectively.

**Scheme 2:**
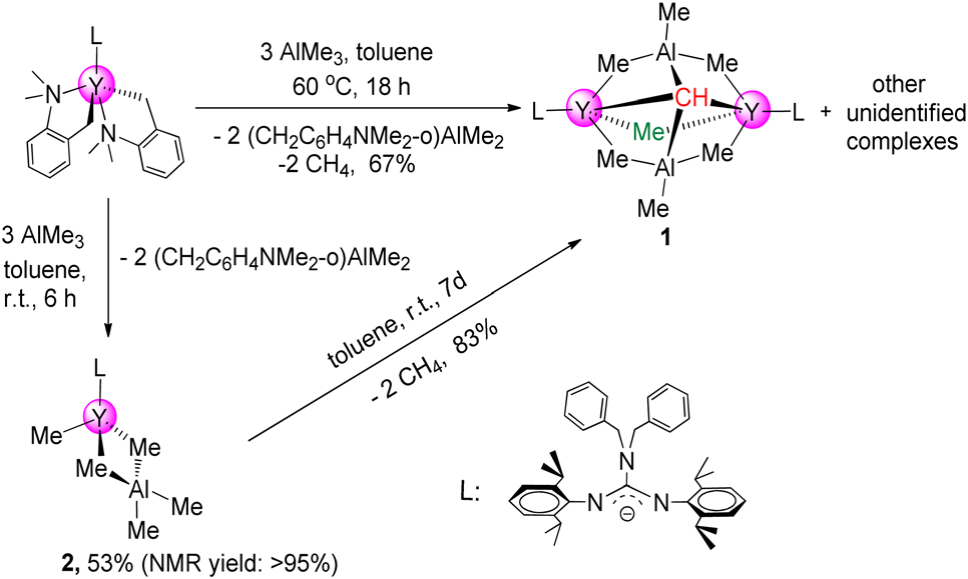
Synthesis of binuclear yttrium carbyne complex 1.

Complex 1 has good thermodynamic stability and multiple potential reactive sites (*e.g.* μ_2_-methyl and [CH]^3−^ groups) which makes this complex suitable for reactivity exploration. Complex 1 underwent a σ-bond metathesis reaction towards one equivalent of phenyl disulfide at room temperature, electron reduction of PhSSPh to form a (SPh)^−^ anion moiety and release PhSMe, affording the yttrium thiolate-bridged carbyne complex [(PhCH_2_)_2_NC(NC_6_H_3_^i^Pr_2_-2,6)_2_]_2_Y_2_(μ_2_-SPh)(AlMe_3_)_2_(μ_4_-CH) (3) in 86% isolated yield (Scheme 2). The molecular structure of 3 is depicted in ESI Fig. S26.†. Complex 3 is a dimer containing a μ_2_-SPh ligand, and the two Y–S bond lengths are 2.799(12) Å and 2.821(13) Å respectively, which are slightly shorter than those in [(C_5_Me_5_)_2_Y(μ-SPh)]_2_,^20^ ranging from 2.893(6) to 2.903(6) Å, probably due to the steric hindrance. In the ^1^H NMR spectrum, the distinct singlet at *δ* = 2.00 (benzene-*d*_6_) ppm is assigned to the [C*H*]^3−^ group, which is shifted to a high field compared to 1. The methyl protons in the AlMe_3_ unit split into two groups at *δ* = 0.89 and −0.50 ppm at room temperature, while they become one broad peak at *δ* = 0.51 ppm at 60 °C (ESI Fig. S8†).^19*b*^ Interestingly, the ^1^H NMR monitoring of this reaction reveals that the chemoselectivity of the reaction of 1 with PhSSPh is independent of the amount of PhSSPh used, and no further reaction was observed even with an excess of PhSSPh.

To develop a synthetic methodology of metal *N*-heterocycle complexes from nitriles,^21^ benzonitrile (2 equiv.) was added into the toluene solution of complex 1 at room temperature, obtaining a diinsertion product [(PhCH_2_)_2_NC(NC_6_H_3_^i^Pr_2_-2,6)_2_]_2_Y_2_[μ_3_-η^1^:η^2^:η^5^-HC(CPhN)_2_](AlMe_3_)(μ_2_-Me) (4) in excellent yield as red blocks (Scheme 2). The X-ray single crystal diffraction study shows the structure of complex 4 in which the tri-anionic amino-alkenyl [CH(CPhN)_2_]^3−^ unit acts as a pentadentate bridge ligand to connect two yttrium atoms in two bonding models (η^5^ and η^2^). In the NMR spectra, the [C*H*]^3−^ group appears as a singlet at *δ* = 7.13 ppm (^13^C: 96.5 ppm), which is shifted to a low field compared to 1 and 3. The average C–N bond length of 1.319(5) Å is longer than the normal C=N double bond distance (1.26 Å),^22^ while the C–C distance (av. 1.439(6) Å) is between the values observed for a C–C single bond and a C=C double bond, indicating that the three negative charges are delocalized partially in the [CH(CPhN)_2_]^3−^ unit. It is interesting that the nucleophilic addition has only taken place on the single [CH]^3−^ group, and the bridged μ_2_-Me(Y–Me–Y) group is inert even if an excess amount of benzonitrile was used under the same conditions, or even heated up to 60 °C for 12 h. The results are clearly different from those of rare-earth-metal methylidene complexes, in which only monoinsertion products [PhC(NC_6_H_3_^i^Pr_2_-2,6)_2_]_3_Ln_3_(μ_2_-Me)_3_(μ_3_-Me)[μ-η^1^:η^1^:η^3^-CH_2_C(Ph)N] (Ln = Y, Lu) were yielded.^17*c*^ Additionally, the present reaction is also different from that of the scandium carbyne complex with PhCN, which afforded the monoaddition product [(C_5_Me_5_)Sc(μ_2_-OMe)]_3_[μ-η^2^:η^2^:η^3^-CHC(Ph)N]^8^ possibly due to the steric hindrance and rigid structure.

**Scheme 3:**
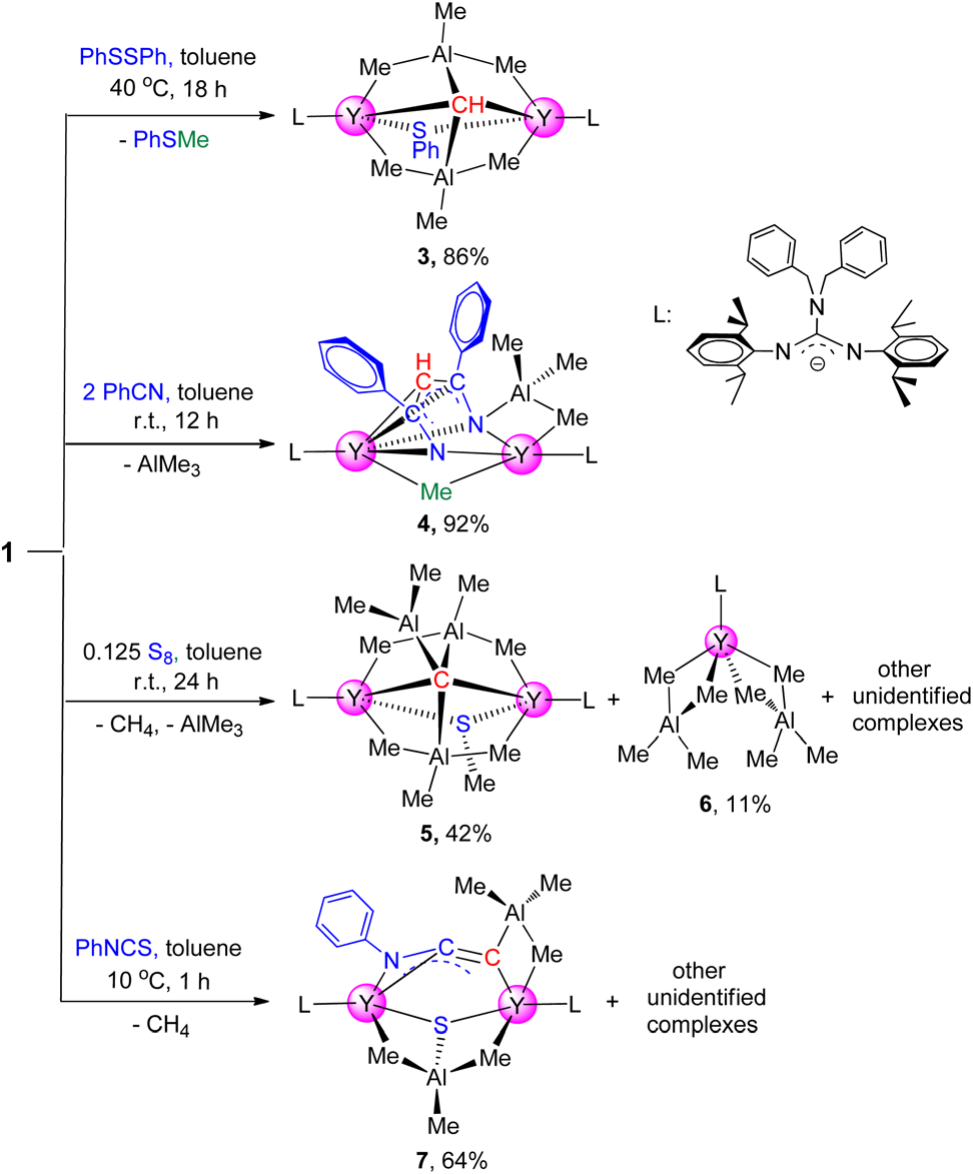
Reactions of complex 1 with benzonitrile, phenyl disulfide, sulfur and phenyl isothiocyanate.

Amazingly, the reaction of complex 1 with sulfur resulted in carbyne deprotonation to afford the rare carbide complex [(PhCH_2_)_2_NC(NC_6_H_3_^i^Pr_2_-2,6)_2_]_2_Y_2_(μ_2_-SMe)(AlMe_3_)_2_(μ_5_-C)(AlMe_2_) (5), along with the formation of the tetramethylaluminate complex [(PhCH_2_)_2_NC(NC_6_H_3_^i^Pr_2_-2,6)_2_]Y(AlMe_4_)_2_ (6) (Scheme 3). The single crystal structure of complex 5 reveals that the stabilization of the carbide unit (C^4−^) is achieved by the alkylaluminate ligands. The carbide carbon atom features a unique distorted trigonal bipyramidal geometry, in which three Al atoms lie in the equatorial plane and two Y atoms occupy the axial positions (ESI Fig. S28†). The bond length of Y–C7(*μ*_5_-C) (av. 2.434(3) Å) is shorter than the Y–C(μ_6_-C) in {[(TMTAC)Y][Y_2_(μ_2_-CH_3_)]}[{(μ_6_-C)[Al(μ_2_-CH_3_)_2_(CH_3_)]_3_}{(μ_3_-CH_2_)(μ_2_-CH_3_)Al(CH_3_)_2_}_2_] (2.696(6) Å),^23^ possibly due to higher coordination numbers of the carbide atoms in latter. The results suggest that the elemental sulfur initiates a bimolecular reaction where the [CH]^3−^ group was deprotonated by the methyl in the AlMe_3_ ligand to form a more stable carbide complex with the release of CH_4_.

Additionally, in order to expand the application of complex 1 as a nucleophile in the synthesis of rare-earth derivatives, the reaction of complex 1 with PhNCS was carried out. This reaction yields a yttrium sulfide complex [(PhCH_2_)_2_NC(NC_6_H_3_^i^Pr_2_-2,6)_2_]_2_Y_2_(μ_3_-η^1^:η^1^:η^2^-CCNPh)(AlMe_3_)_2_(μ_3_-S) (7) with a bridged ketenimine dianion unit (Scheme 3). The single crystal structure of complex 7 reveals that the N1–C1–C2 moiety displays a delocalized electronic framework, in which the C1–N1 (1.329(8) Å) bond length is intermediate between those of typical single and double bonds while the bond length of C1–C2 (1.221(8) Å) is slightly longer than the normal C≡C triple bond distance. And the average bond length of Y–S (2.660(17) Å) is longer than those in [(C_5_Me_5_)_2_Y]_2_(μ-S) (2.54(5) Å)^24^ probably due to the coordination of the AlMe_3_ unit. The mechanism of this reaction is proposed to begin with PhNCS molecule insertion into the Y–C(μ_4_-CH) bond, followed by isomerization to form a C=C double bond, and then C–S single bond cleavage takes place to give the final reductive coupling product 7*via* C–H bond activation (ESI Fig. S33†). This result reveals the unique reactivity of the [CH]^3−^ group towards PhNCS, which is in sharp contrast with that of the cubane-type lutetium methylidene complex with PhNCS, which gave the corresponding ethylene amido thiolate/methylidene product through addition of a Lu–methylidene bond with a less sterically demanding C=S bond followed by isomerization.^11*b*^ The present reaction is also different from previous rare-earth-metal alkylidene complexes in cases where the fragments formed after the cleavage of the C=S double bond in PhNCS participate in the construction of the free coupled organic molecules.^25^

**Scheme 4:**
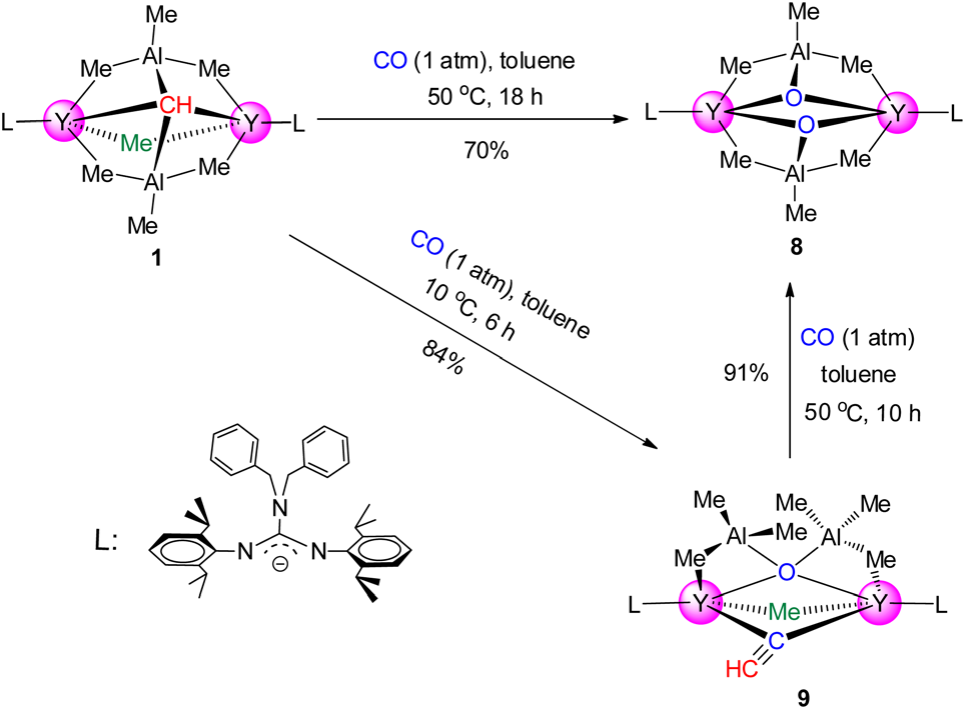
Reactions of complex 1 with CO.

Carbon monoxide (CO), important C1 feedstock, insertion into metal–carbon bonds and its subsequent transformations were thought to play important roles in organometallic chemistry and industrial catalytic processes (Fischer–Tropsch synthesis).^26^ However, the mechanistic details have not been fully understood at the molecular level; in consequence, the reaction of complex 1 with CO (1 atm) was examined at room temperature. Unfortunately, a complicated mixture is detected by using the ^1^H NMR spectrum (ESI Fig. S22†). It is interesting that when the mixture was further heated to 50 °C for 18 h, an unexpected dioxo yttrium complex {[(PhCH_2_)_2_NC(NC_6_H_3_^i^Pr_2_-2,6)_2_]Y(μ_3_-O)(AlMe_3_)}_2_ (8) was isolated in high yield as colorless crystals (Scheme 4). In the NMR spectra of 8, only one singlet at *δ* = −0.16 ppm (^13^C = −2.6 ppm) was ascribed to AlMe_3_ (ESI Fig. S18 and S19†). The X-ray single crystal diffraction study shows that two yttrium centers are linked by two oxygen atoms to form a distorted square in the dimeric system (ESI Fig. S31†), and the Y–O bond lengths (ranging from 2.122(3) to 2.207(2) Å) are comparable to those in (Tp^Me2^)_3_Y_3_(μ_3_-O)(μ_2_-O)(μ_2_-OCH_2_CH=CH_2_)(μ_2_-H) (from 2.127(2) to 2.268(19) Å).^27^ To date, the reaction of CO with rare-earth-metal alkylidene complexes generated only rare-earth-metal ketene complexes through nucleophilic addition of an alkylidene group to CO.^11*b*,15*f*^

**Fig. 2:**
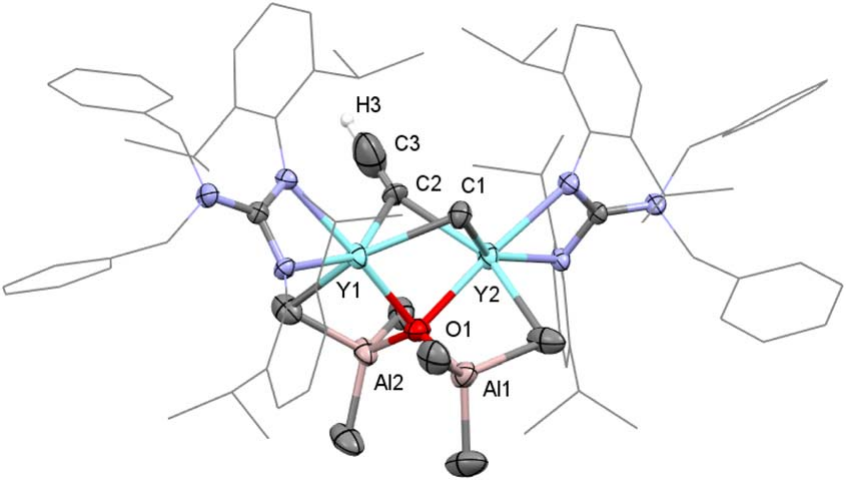
Molecular structure of complex 9 with thermal ellipsoids at 30% probability except for the 2,6-(^i^Pr)_2_C_6_H_3_ groups and benzyl groups in the guanidinate ligand. All of the hydrogen atoms (except for H3) are omitted for clarity. Selected bond distances (Å) and angles (°): Y(1)–C(1) 2.493(6), Y(1)–C(2) 2.537(6), Y(1)–O(1) 2.331(4), and C(2)–C(3) 1.065(12); Y(1)–C(1)–Y(2) 81.99(17), Y(1)–C(2)–Y(2) 84.13(18), Y(1)–O(1)–Y(2) 91.44(12), and Y(1)–C(2)–C(3) 118.1(7).

Although the CO deoxygenative coupling reaction induced by rare-earth-metal phosphinidene and polyhydrido complexes has been well documented,^27,28^ the well-defined rare-earth-metal alkyl complex induced deoxygenative coupling reaction of CO was unprecedented. In order to offer more insight into the formation process of complex 8, the same reaction was carried out in toluene at 10 °C for 6 h. To our delight, the yttrium mono-oxo complex [(PhCH_2_)_2_NC(NC_6_H_3_^i^Pr_2_-2,6)_2_]_2_Y_2_(μ_4_-O)(AlMe_3_)_2_(μ_2_-Me)(μ_2_-C≡CH) (9) was obtained in 84% isolated yield as colorless crystals (Scheme 4). According to single crystal X-ray diffraction, the C2–C3 bond length (1.065(12) Å) reveals a substantial C≡C triple bond character (Fig. 2). And the NMR spectra of 9 show a singlet at *δ* = 1.80 ppm (^13^C{^1^H} = 112.4 ppm), which is regarded as bridged ethynyl (Fig. S17 and S18†). It's worth noting that complex 9 could turn into complex 8 almost quantitatively in toluene at 50 °C for 10 h, indicating that complex 9 is an intermediate in the formation of complex 8. These reactions of CO may offer a convenient route to rare-earth-metal oxo complexes, a class of complexes that are of considerable current interest but still remain limited.^27–29^

### DFT calculations

**Fig. 3:**
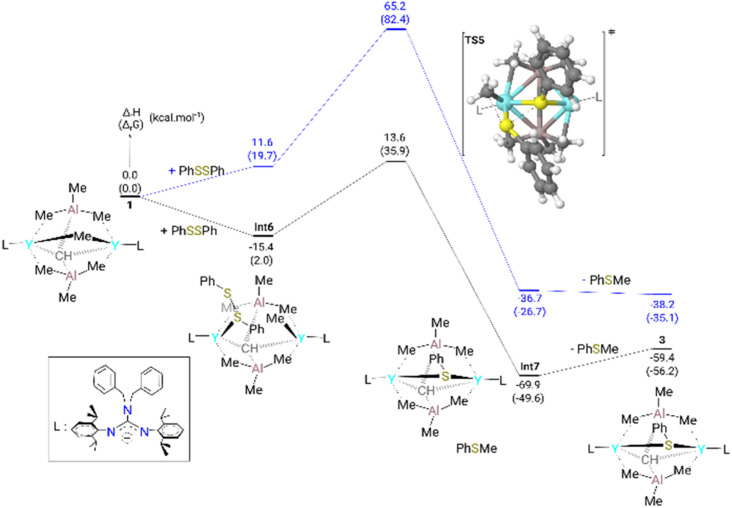
Computed (DFT) enthalpy pathway of complex 3 at room temperature with (black) and without (blue) dispersion correction. The Gibbs free energies are given between brackets.

The formation of complex 3 from complex 1 was investigated computationally at the DFT level (B3PW91 functional including dispersion). As can be seen from Fig. 3, the dispersion corrections play an important role to account for the reactivity due to the size of the substrate PhSSPh. Indeed, the coordination of the substrate is crucial in this case and implies the rearrangement of the μ_2_-Me(Y–Me–Y) to the terminal to allow the coordination of the substrate, which is only favored because of the dispersion forces (stabilization of 27.0 kcal mol^−1^). This is allowed to reach TS5 where the methyl groups attack the sulfur inducing S–S bond breaking with an accessible barrier of 29.0 kcal mol^−1^. This barrier is in line with the experimental observation (18 h at 40 °C). Following the intrinsic reaction coordinate, it yields complex 3 whose formation is thermodynamically favorable (−59.4 kcal mol^−1^). The reaction cannot occur on the carbyne side as moving the carbyne to the top position is too unstable.

**Fig. 4:**
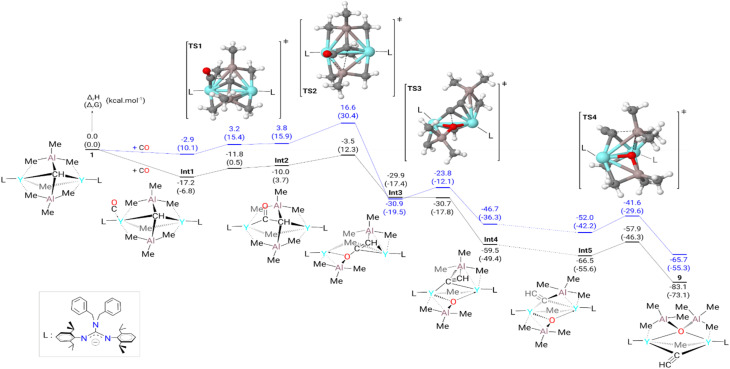
Computed (DFT) enthalpy pathway of complex 9 at room temperature with (black) and without (blue) dispersion correction. The Gibbs free energies are given between brackets.

In addition, the formation of complex 9 from complex 1 was also investigated computationally. The computed enthalpy profile (Fig. 4) begins by the coordination of CO to one of the yttrium centers. This coordination is slightly exothermic by 17.2 kcal mol^−1^. From this adduct (Int1), the migratory insertion of CO onto the Y–CH bond is observed *via*TS1. The associated barrier is very low (6.4 kcal mol^−1^) in line with a facile insertion. Following the intrinsic reaction coordinate, it yields the insertion product (Int2) whose formation is exothermic by 10.0 kcal mol^−1^ with respect to complex 1. This intermediate will undergo an O–Al bond formation which is concomitant with C–Al bond breaking *via*TS2. The associated barrier is 6.5 kcal mol^−1^ from Int2 (13.7 kcal mol^−1^ from complex Int1) and corresponds to the rate-determining step of the entire transformation. This barrier height fits nicely the experimental conditions. TS2 connects with Int3, whose formation is favorable (−29.9 kcal mol^−1^). Int3 undergoes easy C–O bond breaking (TS3) with an enthalpy barrier of 0.2 kcal mol^−1^. This reaction yields a vinylidyne complex (Int4), with an extra stabilization of 29.6 kcal mol^−1^ with respect to Int3. A rotamer of Int4 (Int5) was located on the Potential Energy Surface (PES) with an enthalpy gain of 7.0 kcal mol^−1^. In the final step, the system undergoes C–Al bond formation with concomitant O–Al bond formation *via*TS4. The associated barrier is 8.6 kcal mol^−1^ and allows the formation of complex 9. The latter is found to be the most stable species of the whole reaction profile (−83.1 kcal mol^−1^). Th addition of CO on the μ_2_-Me(Y–Me–Y) side was also computed and is higher in energy (ESI Fig. S34†).

## Conclusions

In summary, the first example of a well-defined binuclear carbyne complex 1 has been demonstrated. Complex 1 revealed diverse reactivity towards various substrates, such as nucleophilic addition with benzonitrile and phenyl isothiocyanate to form aza–allyl complex 4 and sulfide complex 7, σ-bond metathesis towards phenyl disulfide to give thiolate complex 3, and insertion of sulfide into the Y–C(μ_2_-Me) bond along with carbyne deprotonation to the afford carbide complex 5. Unexpectedly, the rare-earth-metal carbyne complex induced deoxygenative coupling reaction of CO was also presented for the first time. The results have afforded some evidence to suggest that unsaturated substrates are attacked preferentially by the methine rather than the methyl. The observed rich reactivity of this binuclear carbyne complex could provide new insight into the nature of rare-earth-metal carbyne complexes. We are currently probing the reactivity of complex 1 with other molecules.

## Data availability

The data that support the findings of this study are available in the ESI† of this article.

## Author contributions

Li, Meng designed the carbyne complex. Jiang, Wen and Kong, Feng performed the synthetic experiment and X-ray diffraction measurements. Rosal, Iker del and Maron, Laurent performed the DFT calculations. Zhang, Lixin and Wang, Kai reviewed/revised the manuscript. All authors contributed to the analysis of the manuscript.

## Conflicts of interest

The authors declare no conflict of interest.

## Supplementary Material

SC-014-D3SC03483F-s001

SC-014-D3SC03483F-s002
