# Supplementary material for: A binuclear guanidinate yttrium carbyne complex: unique reactivity toward unsaturated C–N, C–O and C–S bonds
Source: Chem Sci. 2023 Aug 3;14(34):9154–60. doi: 10.1039/d3sc03483f (PMC10466373; doi:10.1039/d3sc03483f)
Supplement: SC-014-D3SC03483F-s001 [file SC-014-D3SC03483F-s001.pdf]

Electronic Supplementary Material (ESI) for Chemical Science.
This journal is © The Royal Society of Chemistry 2023

Supporting Information

Binuclear Guanidinate Yttrium Carbyne Complex: Unique Reactivity toward Unsaturated C−N, C−O and C−S Bonds

Wen Jiang,[† 1] *Feng Kong,*[† 1] *Iker del Rosal,* [2]*Meng Li,*[1] *Kai Wang,*[1] *Laurent Maron** [2] *and Lixin Zhang** [1]

[1] Department of Chemistry, Shanghai Key Laboratory of Molecular Catalysis and Innovative Materials Fudan University, Shanghai, 200438 (P. R. China)

[2] LPCNO, CNRS, and INSA, Université Paul Sabatier, 135 Avenue de Rangueil, Toulouse 31077, France

[†] These authors contributed equally to this work.

E-mail: lixinzh@fudan.edu.cn and maron@irsamc.ups-tlse.fr

Tables of Contents

General procedure (page 2)

Experiment section (page 2-5)

NMR spectra of selected complexes (page 6-17)

Molecular structure of selected complexes (page 18-23)

Crystal data of selected complexes (page 24-26)

Proposed reaction pathway for formation of complex **7** (page 27)

Computational details (page 28-266)

References (page 267)

Materials and Methods. All manipulations were performed with rigorous exclusion of air and water, using Schlenk techniques or an MBraun glovebox (Unilab MBraun; <1 ppm O_2, <1 ppm H_2O). Toluene, THF, and hexane were purified using Grubbs-type columns (MBraun SPS-800, solvent purification system), then further dried over fresh Na chips and stored in a glovebox. Phenyl disulfide, sulfur and $AlMe_3$ (1 mol L^{-1} in hexane) were purchased from J&K Co., Ltd and used without purification. Benzonitrile and phenyl isothiocynate were obtained from J&K Co., Ltd and distilled from CaH_2, then further dried by 4 Å molecular sieves. Highly pure CO (99.999%) was purchased from Pujiang Gas and dried by passing through activated 4 Å molecular sieves. Benzene-d_6 was obtained from J&K Co., Ltd and Cambridge Isotope and dried by using sodium chips. ^{1}H and $^{13}C\{^{1}H\}$ NMR spectra of complexes were recorded using a JEOL ECA-400 NMR spectrometer (FT, 400 MHz for ^{1}H; 100 MHz for $^{13}C\{^{1}H\}$). The combustion method was used for the carbon, hydrogen and nitrogen analyses on an Elementar Vario EL III analyzer at Fudan Univerisity (China).

[$(PhCH_2)_2NC(NC_6H_3{}^{i}Pr_2$-2,6$)_2]_2Y_2(\mu_2$-Me$)(AlMe_3)_2(\mu_4$-CH) (1). ***Path A:*** A toluene solution of $AlMe_3$ (1.5 mL, 1M in hexane, 1.5 mmol) was added slowly to a stirred toluene solution (25 mL) of [$(PhCH_2)_2NC(NC_6H_3{}^{i}Pr_2$-2,6$)_2$]Y($CH_2C_6H_4NMe_2$-*o*$)_2$ (0.46 g, 0.5 mmol). The solution was left to stir 18 h at 60 ^{o}C. The toluene and volatile substances were removed under vacuum, and the oily yellow residue turned to white powder after washing twice with hexane (2 mL), which was collected by filtration and dried. Colourless crystalline **1** was harvested by recrystallization in toluene at ambient temperature, yield (0.24 g, 67%). ***Path B:*** Complex [$(PhCH_2)_2NC(NC_6H_3{}^{i}Pr_2$ -2,6$)_2$]Y($AlMe_4$)(Me) (0.37 g, 0.5 mmol) could turn into complex **1** when dissoved in toluene for 7 days, recrystallization in toluene at ambient temperature, yield (0.30 g, 83%). ^{1}H NMR (400 MHz, C_6D_6, 25 ^{o}C): δ (ppm) = 6.99-6.97 (m, 8H, Ar), 6.92 (br, 12H, Ar), 6.87-6.84 (m, 4H, Ar), 6.78 (br, 8H, Ar), 4.06 (s, 8H, CH_2Ph), 3.77 (m, 8H, *CH*Me_2), 2.36 (s, 1H, μ_4-CH), 1.42 (d, $^{3}J_{H-H}$ = 6 Hz, 24H, CH*Me_2*), 1.19 (d, $^{3}J_{H-H}$ = 5.6 Hz, 24H, CH*Me_2*), 0.34 (br, 18H, Al*Me_3*), 0.26 (s, 3H, μ_2-Me). $^{13}C\{^{1}H\}$ NMR (100 MHz, C_6D_6, 25 ^{o}C): δ (ppm) = 165.0 (s, N*C*N), 143.1 (s, Ar), 142.3 (s, Ar), 136.7 (s, Ar), 129.3 (s, Ar), 127.5 (s, Ar), 124.7 (s, Ar), 124.2 (s, Ar), 90.1 (s, μ_4-*C*H), 52.4 (s, *C*H_2Ph), 29.8 (s, μ_2-Me), 28.6 (s, *C*HMe_2), 27.0 (s, CH*Me_2*), 25.0 (s, CH*Me_2*), 2.6 (br, Al*Me_3*). Anal. Calcd for $C_{86}H_{118}Al_2N_6Y_2$(%): C, 70.38; H, 8.10; N, 5.73; Found C, 70.69; H, 8.23; N, 5.67.

[$(PhCH_2)_2NC(NC_6H_3{}^{i}Pr_2$-2,6$)_2$]Y($AlMe_4$)(Me) (2). An $AlMe_3$/toluene solution (1.5 mL, 1M in hexane, 1.5 mmol) was added slowly to a stirred toluene solution (10 mL) of [$(PhCH_2)_2NC(NC_6H_3{}^{i}Pr_2$-2,6$)_2$] Y($CH_2C_6H_4NMe_2$-*o*$)_2$ (0.46 g, 0.5 mmol). The solution was left to stir 6 h at ambient temperature. The toluene was removed under vacuum, and the oily yellow residue turned to white powder after washing twice with hexane (2 mL), which was collected by filtration and dried, recrystallization in toluene at -35 ^{o}C for one day, yield (0.19 g, 53%). ^{1}H NMR (400 MHz, C_6D_6, 25 ^{o}C): δ (ppm) = 7.08-7.05 (m, 4H, Ar), 7.05-6.95 (m, 8H, Ar), 6.83-6.82 (m, 4H, Ar), 3.99 (br, 4H, C*H_2*Ph), 3.82 (m, 4H, *CH*Me_2), 1.34 (d, 12H, $^{3}J_{H-H}$ = 5.6 Hz, CH*Me_2*), 1.14 (d, 12H, $^{3}J_{H-H}$ = 6 Hz, CH*Me_2*), 0.27 (s, 3H, *Me*), -0.14 (br, 12H, [Al*Me_4*$]^{-}$). $^{13}C\{^{1}H\}$ NMR (100 MHz, C_6D_6, 25 ^{o}C): δ (ppm) = 164.1 (s, N*C*N), 142.3 (s, Ar), 142.2 (s, Ar), 136.2 (s, Ar), 128.9 (s, Ar), 128.7 (s, Ar), 125.2 (s, Ar), 124.6 (s, Ar), 51.8 (s, *C*H_2Ph), 28.6 (s, *C*HMe_2), 27.0 (s, CH*Me_2*), 26.5 (s, *Me*), 23.8 (s, CH*Me_2*), 3.8 (s, [Al*Me_4*$]^{-}$). Anal. Calcd for $C_{44}H_{63}AlYN_3$(%): C, 70.47; H, 8.47; N, 5.60; Found C, 70.25; H, 8.15; N, 5.49.

[(PhCH$_2$)$_2$NC(NC$_6$H$_3$^{i}Pr$_2$-2,6)$_2$]$_2$Y$_2$(μ_2-SPh)(AlMe$_3$)$_2$(μ_4-CH) (3). A toluene solution (5 mL) of phenyl disulfide (0.06 g, 0.3 mmol) was added dropwise to a stirring toluene solution (15 mL) of complex **1** (0.44 g, 0.3 mmol). And the reaction mixture was left to stir for 18 hours at 40 ^{o}C. A colour change of the solution was observed from colourless to yellow. The toluene solvent was removed under vacuum and wash the reaction mixture with hexane for twice to obtain complex **3** as powder. Colourless crystalline **3** was harvested by recrystallization in toluene at ambient temperature, yield (0.40 g, 86%). ^{1}H NMR (400 MHz, C$_6$D$_6$, 25 ^{o}C): δ (ppm) = 7.06 (m, 8H, Ar), 6.98-6.94 (m, 4H, Ar), 6.91-6.90 (m, 12H, Ar), 6.79 (br, 9H, Ar), 6.65 (t, $^3J_{H-H}$ = 8 Hz, 2H, Ar), 5.91 (d, $^3J_{H-H}$ = 8 Hz, 2H, Ar), 4.13 (s, 8H, C*H$_2$*Ph), 3.83 (m, 8H, C*H*Me$_2$), 2.00 (s, 1H, μ_4-C*H*), 1.44 (d, $^3J_{H-H}$ = 8 Hz, 24H, CH*Me$_2$*), 1.18 (d, $^3J_{H-H}$ = 8 Hz, 24H, CH*Me$_2$*), 0.89 (br, 12H, (μ_2-*Me*)$_2$AlMe), -0.50 (br, 6H, (μ_2-Me)$_2$Al*Me*). ^{1}H NMR (500 MHz, C$_6$D$_6$, 60 ^{o}C): δ (ppm) = 7.05 (br, 4H, Ar), 7.03 (s, 4H, Ar), 6.96-6.93 (m, 4H, Ar), 6.91-6.89 (m, 12H, Ar), 6.78-6.74 (m, 9H, Ar), 6.62 (t, $^3J_{H-H}$ = 8 Hz, 2H, Ar), 5.96 (d, $^3J_{H-H}$ = 8 Hz, 2H, Ar), 4.14 (s, 8H, C*H$_2$*Ph), 3.81 (m, 8H, C*H*Me$_2$), 2.00 (s, 1H, μ_4-C*H*), 1.42 (d, $^3J_{H-H}$ = 8 Hz, 24H, CH*Me$_2$*), 1.18 (d, $^3J_{H-H}$ = 8 Hz, 24H, CH*Me$_2$*), 0.51 (br, 18H, Al*Me$_3$*). ^{13}C{^{1}H} NMR (100 MHz, C$_6$D$_6$, 25 ^{o}C): δ (ppm) = 165.6 (s, N*C*N), 143.2 (s, Ar), 142.8 (s, Ar), 137.0 (s, Ar), 136.5 (s, Ar), 131.7 (s, Ar), 129.6 (s, Ar), 129.3 (s, Ar), 128.1 (s, Ar), 127.5 (s, Ar), 125.0 (s, Ar), 124.5 (s, Ar), 124.1 (s, Ar), 91.4 (s, μ_4-*C*H), 52.4 (s, *C*H$_2$Ph), 28.7 (s, *C*HMe$_2$), 26.8 (s, CH*Me$_2$*), 25.5 (s, CH*Me$_2$*), 9.7 (s, Al*Me$_3$*). Anal. Calcd for C$_{91}$H$_{120}$Al$_2$N$_6$SY$_2$(%): C, 69.98; H, 7.74; N, 5.38; Found: C, 70.37; H, 7.35; N, 5.19.

[(PhCH$_2$)$_2$NC(NC$_6$H$_3$^{i}Pr$_2$-2,6)$_2$]$_2$Y$_2$[μ_3-η^5:η^2:η^1-HC(CPhN)$_2$](AlMe$_3$)(μ_2-Me) (4). A toluene solution (5 mL) of benzonitrile (70 µL 0.6 mmol) was added dropwise to a stirring toluene solution (15 mL) of complex **1** (0.44 g, 0.3 mmol). And the reaction mixture was left to stir for 12 hours at room temperature. The toluene solvent was removed under vacuum and wash the reaction mixture with hexane for three times to obtain complex **4** as red powder. Red crystalline **4** was harvested by recrystallization in toluene at ambient temperature, yield (0.44 g, 92%). ^{1}H NMR (400 MHz, C$_6$D$_6$, 25 ^{o}C): δ (ppm) = 8.15 (d, $^3J_{H-H}$ = 8 Hz, 2H, Ar), 7.41 (m, 2H, Ar), 7.31 (m, 1H, Ar), 7.24-7.22 (m, 2H, Ar), 7.13-6.96 (m, 19H, Ar and C*H*), 6.88-6.87 (m, 10H, Ar), 6.63-6.61 (m, 7H, Ar), 4.34 (s, 2H, C*H$_2$*Ph), 4.30 (s, 2H, C*H$_2$*Ph), 3.91 (s, 2H, C*H$_2$*Ph), 3.87 (s, 2H, C*H$_2$*Ph), 3.79-3.72 (m, 8H, C*H*Me$_2$), 1.27 (d, $^3J_{H-H}$ = 8 Hz, 12H, CH*Me$_2$*), 1.21 (d, $^3J_{H-H}$ = 8 Hz, 12H, CH*Me$_2$*), 1.13 (d, $^3J_{H-H}$ = 4 Hz, 12H, CH*Me$_2$*), 1.03 (d, $^3J_{H-H}$ = 8 Hz, 12H, CH*Me$_2$*), 0.65 (s, 3H, μ_2-*Me*), -0.46 (s, 9H, Al*Me$_3$*). ^{13}C{^{1}H} NMR (100 MHz, C$_6$D$_6$, 25 ^{o}C): δ (ppm) = 177.2 (s, HC(*C*PhN)$_2$), 170.3 (s, HC(*C*PhN)$_2$), 165.1 (s, N*C*N), 145.0 (s, Ar), 143.6 (s, Ar), 142.0 (s, Ar), 141.6 (s, Ar), 140.8 (s, Ar), 136.2 (s, Ar), 130.1 (s, Ar), 129.0 (s, Ar), 128.2 (s, Ar), 127.0 (s, Ar), 126.8 (s, Ar), 125.3 (s, Ar), 124.1 (s, Ar), 124.0 (s, Ar), 123.7 (s, Ar), 96.5 (s, H*C*(CPhN)$_2$), 52.4 (s, *C*H$_2$Ph), 29.8 (s, μ_2-*Me*), 28.1 (s, *C*HMe$_2$), 27.8 (s, *C*HMe$_2$), 26.5 (s, CH*Me$_2$*), 26.1 (s, CH*Me$_2$*), 24.0 (s, CH*Me$_2$*), 23.5 (s, CH*Me$_2$*), 0.3 (s, Al*Me$_3$*). Anal. Calcd for C$_{97}$H$_{116}$AlN$_8$Y$_2$(%): C, 72.87; H, 7.31; N, 7.01; Found: C, 72.47; H, 7.25; N, 7.11.

[(PhCH$_2$)$_2$NC(NC$_6$H$_3$^{i}Pr$_2$-2,6)$_2$]$_2$Y$_2$(AlMe$_3$)$_2$(μ_2-SMe)(μ_5-C)(AlMe$_2$) (5). A toluene solution (5 mL) of sulfur (0.01 g, 0.3 mmol) was added dropwise to a stirring toluene solution (15 mL) of complex **1** (0.44 g, 0.3 mmol). And the reaction mixture was left to stir for 24 hours at room temperature. The toluene solvent and volatile substances were removed under vacuum and wash the reaction mixture with hexane for three times to obtain complex **5** as white powder. Colourless crystalline **5** was harvested by recrystallization in toluene at ambient temperature, yield (0.19 g, 42%). ^{1}H

NMR (400 MHz, C_6D_6, 25 °C): δ (ppm) = 7.03 (m, 7H, Ar), 6.94-6.96 (d, $^3J_{H-H}$ = 8 Hz, 4H, Ar), 6.83-6.81 (m, 14H, Ar), 6.43-6.41 (m, 7H, Ar), 4.18(s, 8H, C*H*$_2$Ph), 3.82-3.74(m, 8H, C*H*Me$_2$), 1.46 (d, $^3J_{H-H}$ = 8 Hz, 24H, CH*Me*$_2$), 1.30 (d, $^3J_{H-H}$ = 4 Hz, 12H, CH*Me*$_2$), 1.20 (d, $^3J_{H-H}$ = 8 Hz, 12H, -CH*Me*$_2$), 0.81 (s, 12H, (μ_2-*Me*)$_2$AlMe), 0.80 (s, 3H, μ_2-S*Me*), -0.05 (s, 6H, (μ_2-Me)$_2$Al*Me*), -0.84 (s, 6H, -CAl*Me*$_2$). ^{13}C{^{1}H} NMR (100 MHz, C_6D_6, 25 °C): δ (ppm) = 167.8 (s, N*C*N), 144.4 (s, Ar), 143.4 (s, Ar), 143.0 (s, Ar), 141.8 (s, Ar), 136.2 (s, Ar), 129.4 (s, Ar), 127.1 (s, Ar), 125.2 (s, Ar), 125.0 (s, Ar), 124.6 (s, Ar), 124.3 (s, Ar), 53.0 (s, *C*H$_2$Ph), 28.6 (s, *C*HMe$_2$), 28.4 (s, CH*Me*$_2$), 28.3 (s, CH*Me*$_2$), 26.7 (s, *C*HMe$_2$), 24.9 (s, CH*Me*$_2$), 24.7 (s, CH*Me*$_2$), 10.8 (s, Al*Me*$_3$), 6.5 (s, μ_2-S*Me*), -0.8 (s, μ_4-CAl*Me*$_2$). Anal. Calcd for $C_{88}H_{123}Al_3N_6SY_2$(%): C, 67.94; H, 7.97; N, 5.40; Found: C, 67.57; H, 7.67; N, 5.41.

[(PhCH$_2$)$_2$NC(NC$_6$H$_3$^{i}Pr$_2$-2,6)$_2$]Y(AlMe$_4$)$_2$ (6). A toluene solution (5 mL) of sulfur (0.01 g, 0.3 mmol) was added dropwise to a stirring toluene solution (15 mL) of complex **1** (0.44 g, 0.3 mmol). And the reaction mixture was left to stir for 24 hours at room temperature. The toluene solvent and volatile substances were removed under vacuum and wash the reaction mixture with hexane for three times to obtain complex 5 as white powder. And then, the colorless crystal **6** was obtained from the residual solution at -35 °C for overnight (0.05 g, 11%). ^{1}H NMR (400 MHz, C_6D_6, 25 °C): δ (ppm) = 7.01-6.98 (m, 6H, Ar), 6.87-6.85 (m, 6H, Ar), 6.61-6.60 (m, 4H, Ar), 4.04 (s, 4H, C*H*$_2$C$_6$H$_5$), 3.66 (m, 4H, *CH*Me$_2$), 1.36 (d, 12H, $^3J_{H-H}$ = 8 Hz, CH*Me*$_2$), 1.10 (d, 12H, $^3J_{H-H}$ = 8 Hz, CH*Me*$_2$), 0.04 (s, 24H, [Al*Me*$_4$]$^-$). ^{13}C{^{1}H} NMR (100 MHz, C_6D_6, 25 °C): δ (ppm) = 167.0 ppm (s, N*C*N). 143.1 (s, Ar), 142.0 (s, Ar), 135.4 (s, Ar), 129.7 (s, Ar), 127.8 (s, Ar), 125.9 (s, Ar), 125.2 (s, Ar), 52.6 (s, *C*H$_2$Ph), 28.4 (s, *C*HMe$_2$), 27.0 (s, CH*Me*$_2$), 25.5 (s, CH*Me*$_2$), 2.5 (s, [Al*Me*$_4$]$^-$). Anal. Calcd for $C_{47}H_{72}Al_2N_3Y$ (%): C, 68.68; H, 8.83; N, 5.11; Found C, 68.77; H, 8.50; N, 5.42.

[(PhCH$_2$)$_2$NC(NC$_6$H$_3$^{i}Pr$_2$-2,6)$_2$]$_2$Y$_2$(μ_3-η^1:η^1:η^2-CCNPh)(AlMe$_3$)$_2$(μ_3-S) (7). A toluene solution (5 mL) of phenyl isothiocynate (35 µL, 0.3 mmol) was added dropwise to a stirring toluene solution (15 mL) of complex **1** (0.44 g, 0.3 mmol). And the reaction mixture was left to stir for 1 hour at 10 °C. Instant colour change from colourless to yellow was observed. The toluene solvent and volatile substances were removed under vacuum and wash the reaction mixture with hexane for three times to obtain complex **7** as white powder. Colourless crystalline **7** was harvested by recrystallization in toluene at ambient temperature, yield (0.30 g, 64%). ^{1}H NMR (400 MHz, C_6D_6, 25 °C): δ (ppm) = 7.60-7.55 (m, 2H, Ar), 7.26 (t, $^3J_{H-H}$ = 8 Hz, 2H, Ar), 7.13-7.02 (m, 14H, Ar), 6.89 (br s, 14H, Ar), 6.72-6.62 (m, 8H, Ar), 4.17-4.04(m, 8H, C*H*$_2$Ph), 3.88-3.74(m, 8H, C*H*Me$_2$), 1.45-0.78 (m, 48H, CH*Me*$_2$), 0.16 (s, 9H, SAl*Me*$_3$), -0.12 (br, 9H, CAl*Me*$_3$). ^{13}C{^{1}H} NMR (100 MHz, C_6D_6, 25 °C): δ (ppm) = 164.3 (s, N*C*N), 146.9 (s, Ar), 143.5 (s, Ar), 142.4 (s, Ar), 140.8 (s, Ar), 136.0 (s, C*C*NPh), 129.5 (s, Ar), 129.3 (s, Ar), 129.1 (s, Ar), 127.5 (s, Ar), 125.7 (s, Ar), 125.1 (s, Ar), 124.8 (s, Ar), 122.0 (s, Ar), 121.0 (s, Ar), 91.3 (d, *J* = 21 Hz, *C*CNPh), 52.2 (s, *C*H$_2$Ph), 28.7 (s, *C*HMe$_2$), 27.4 (s, CH*Me*$_2$), 25.3 (s, CH*Me*$_2$), 23.8 (s, CH*Me*$_2$), 21.5 (s, CH*Me*$_2$), 1.4 (s, SAl*Me*$_3$), -1.7 (s, CAl*Me*$_3$). Anal. Calcd for $C_{92}H_{119}Al_2N_7SY_2$(%): C, 69.64; H, 7.56; N, 6.18; Found: C, 70.01; H, 7.25; N, 6.09.

{[(PhCH$_2$)$_2$NC(NC$_6$H$_3$^{i}Pr$_2$-2,6)$_2$]Y(μ_3-O)(AlMe$_3$)}$_2$ (8). A toluene (15 mL) solution of complex **1** (0.44 g, 0.3 mmol) was placed in a tube with a Teflon stopcock and degassed by a freeze pump thaw cycle. The CO (1 atm) was introduced into the tube and the solution was left to stir for 18 hours at 50 °C. Colour change from colourless to dark brown was observed. The toluene solvent was removed under reduced pressure to saturation. Colorless crystals of **8** (0.37 g, 84%) were

harvested after the solution stood at ambient temperature after two days. ^{1}H NMR (400 MHz, C_6D_6, 25 °C): δ (ppm) = 7.08-7.02 (m, 12H, Ar), 6.91-6.89 (m, 12H, Ar), 6.72-6.70 (m, 12H, Ar), 3.98 (s, 8H, *CH$_2$*Ph), 3.73 (m, 8H, C*H*Me$_2$), 1.29 (d, $^{3}J_{H-H}$ = 8 Hz, 24H, CH*Me$_2$*), 1.14 (d, $^{3}J_{H-H}$ = 8 Hz, 24H, CH*Me$_2$*), -0.16 (s, 18H, Al*Me$_3$*). $^{13}C\{^{1}H\}$ NMR (100 MHz, C_6D_6, 25 °C): δ (ppm) = 165.5 (s, N*C*N), 143.5 (s, Ar), 142.6 (s, Ar), 136.4 (s, Ar), 129.2 (s, Ar), 127.4 (s, Ar), 124.7 (s, Ar), 124.4 (s, Ar), 124.4 (s, Ar), 51.9 (s, *C*H$_2$Ph), 28.5 (s, *C*HMe$_2$), 27.1 (s, CH*Me*$_2$), 23.5 (s, CH*Me*$_2$), -2.6 (s, Al*Me$_3$*). Anal. Calcd for $C_{84}H_{114}Al_2N_6O_2Y_2$(%): C, 68.56; H, 7.81; N, 5.71; Found: C, 68.83; H, 7.80; N, 5.71.

[(PhCH$_2$)$_2$NC(NC$_6$H$_3$^{i}Pr$_2$-2,6)$_2$]$_2$Y$_2$(μ_4-O)(AlMe$_3$)$_2$(μ_2-Me)(μ_2-C≡CH) (9). A toluene solution (15 mL) of complex **1** (0.44 g, 0.3 mmol) was placed in a tube with a Teflon stopcock and degassed by a freeze pump thaw cycle. The CO (1 atm) was introduced into the tube and the solution was left to stir for 6 hours at 10 °C. Colour change from colourless to yellow was observed. The toluene solvent was removed under reduced pressure to saturation. Colorless crystals of **9** were harvested after the solution stood at ambient temperature, yield (0.31 g, 70%). ^{1}H NMR (400 MHz, C_6D_6, 25 °C): δ (ppm) = 7.09-7.04 (m, 12H, Ar), 6.89 (br, 12H, Ar), 6.70 (br, 8H, Ar), 6.89 (br, 14H, Ar), 4.09 (br, 8H, C*H$_2$*Ph), 3.79 (m, 8H, C*H*Me$_2$), 1.80 (s, 1H, μ_2-C≡C*H*), 1.46-1.37(m, 24, CH*Me$_2$*), 1.14 (br, 24H, CH*Me$_2$*), 0.44 (br, 3H, μ_2-Me), -0.19(br, 18H, Al*Me$_3$*). $^{13}C\{^{1}H\}$ NMR (100 MHz, C_6D_6, 25 °C): δ (ppm) = 166.4 (s, N*C*N), 143.2 (s, Ar), 142.9 (s, Ar), 136.3 (s, Ar), 129.2 (s, Ar), 127.4 (s, Ar), 124.9 (s, Ar), 124.8 (s, Ar), 124.7 (s, Ar), 112.4 (s, μ_2-C≡*C*H), 52.1 (s, *C*H$_2$Ph), 35.2 (s, μ_2-Me), 28.5 (s, *C*HMe$_2$), 27.1 (br, CH*Me*$_2$), 25.0 (br, CH*Me*$_2$), 7.8 (s, Al*Me$_3$*). Anal. Calcd for $C_{87}H_{118}Al_2N_6OY_2$(%): C, 69.86; H, 7.95; N, 5.62; Found: C, 70.21; H, 8.25; N, 5.67.

NMR spectra of all complexes

The solid samples were dissolved in C_6D_6, and then transferred into a J-Young NMR tube in the glove-box. The ^1H NMR and $^{13}C\{^1H\}$ NMR spectra were recorded on a JEOL ECA-400 NMR spectrometer (FT, 400 MHz for ^1H; 100 MHz for $^{13}C\{^1H\}$) in C_6D_6 at room temperature (except for Fig. S7).

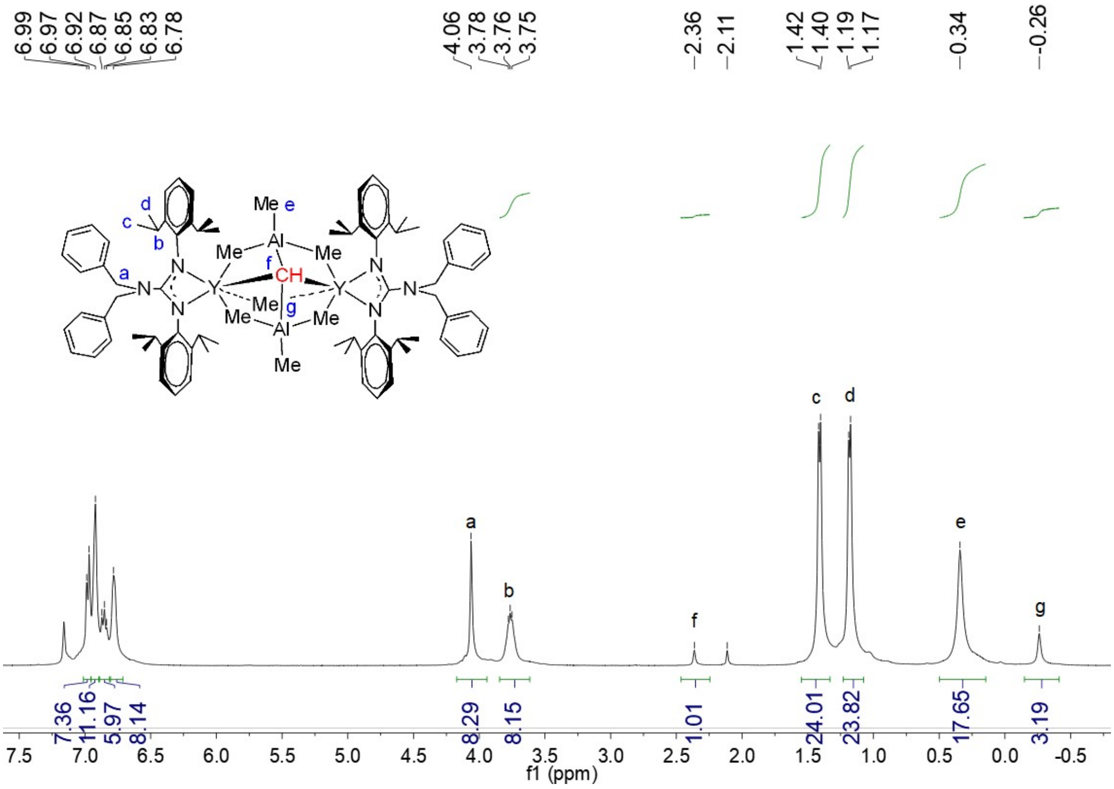

Fig. S1 ^1H NMR spectrum of **1** obtained in C_6D_6 at room temperature.

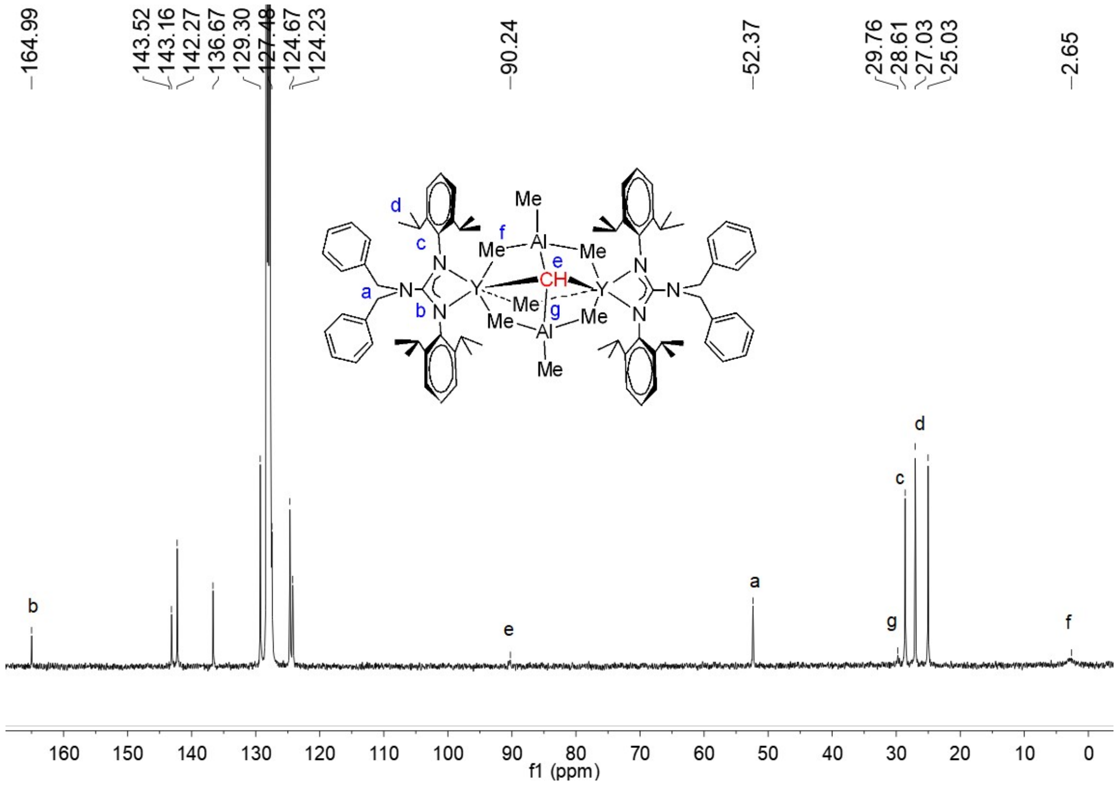

Fig. S2 $^{13}C\{^{1}H\}$ NMR spectrum of **1** obtained in C_6D_6 at room temperature.

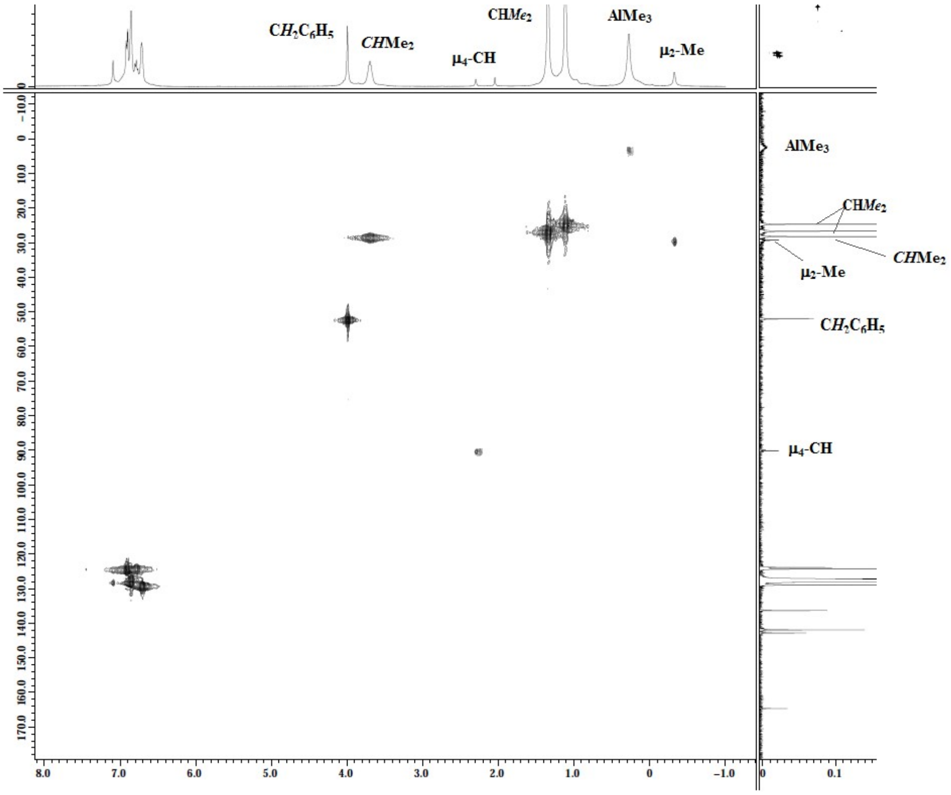

Fig. S3 Two-dimensional ^{1}H-^{13}C HMQC NMR spectrum of complex **1** (^{1}H NMR spectrum (400 MHz) on the top, $^{13}C\{^{1}H\}$ NMR spectrum (100 MHz) on the right).

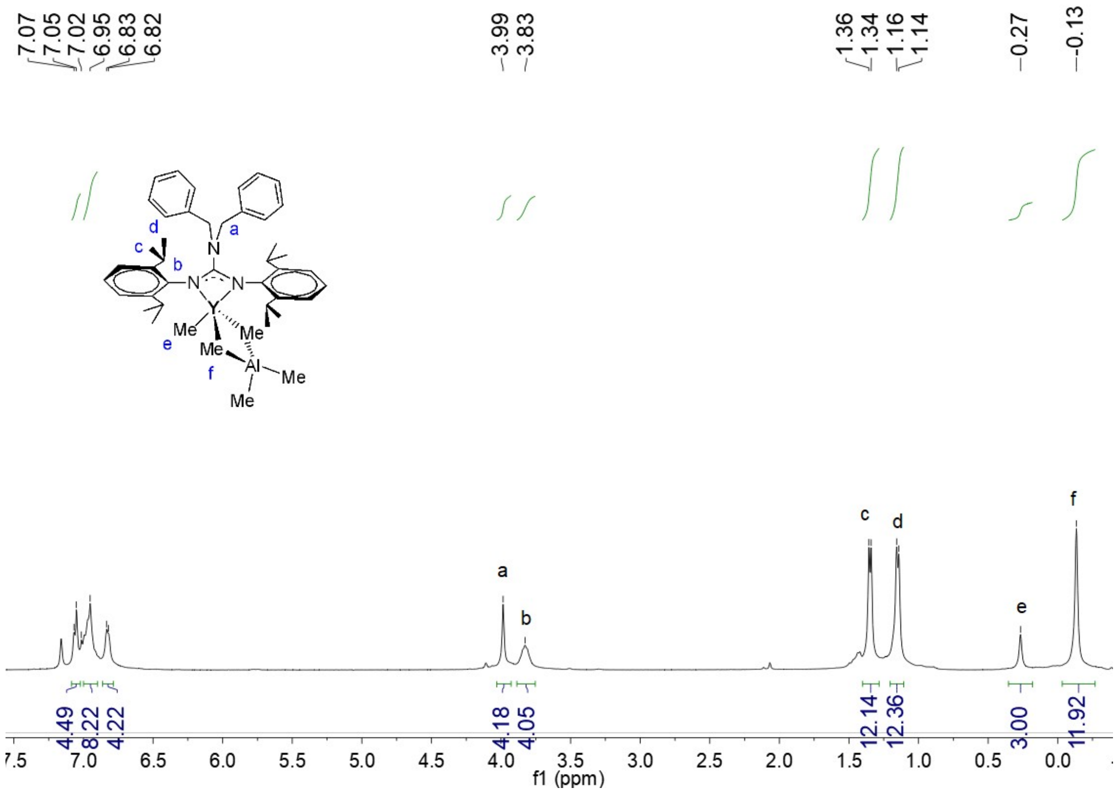

Fig. S4 ^{1}H NMR spectrum of **2** obtained in C_6D_6 at room temperature.

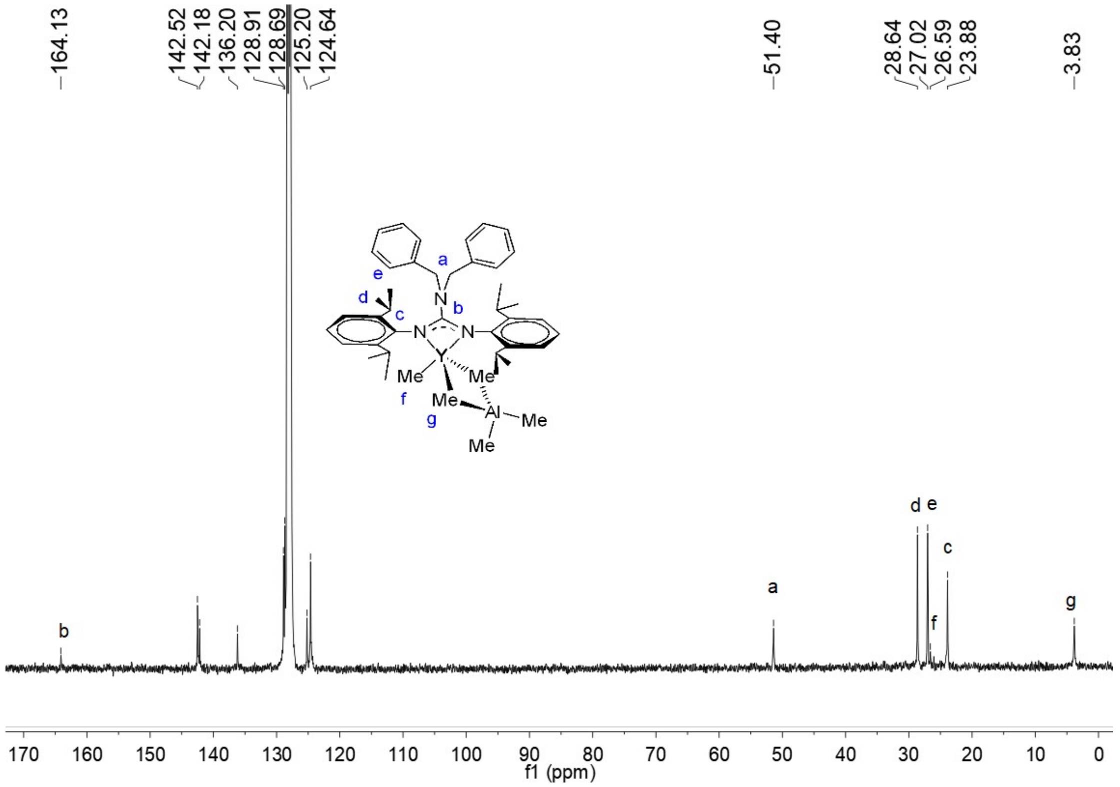

Fig. S5 $^{13}C\{^{1}H\}$ NMR spectrum of **2** obtained in C_6D_6 at room temperature.

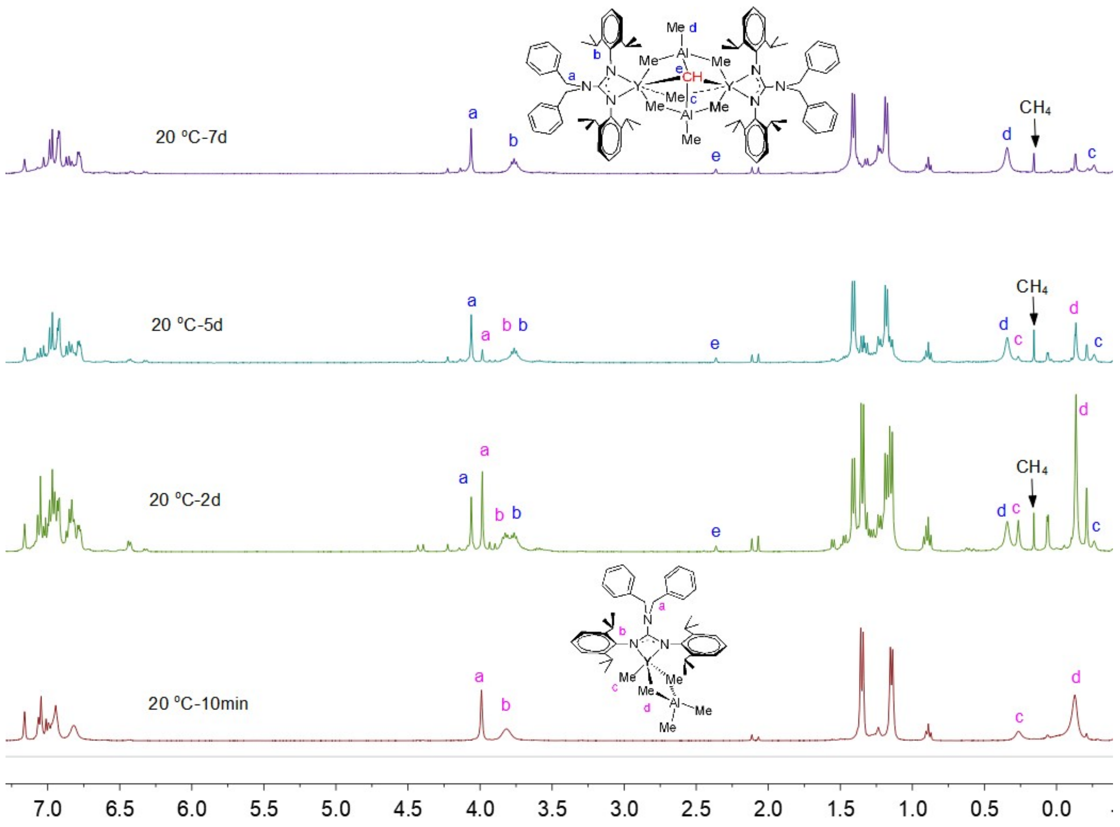

Fig. S6 The *in situ* ^{1}H NMR spectrum was carried out on conversion of complex **1** from complex **2** in C_6D_6 at room temperature.

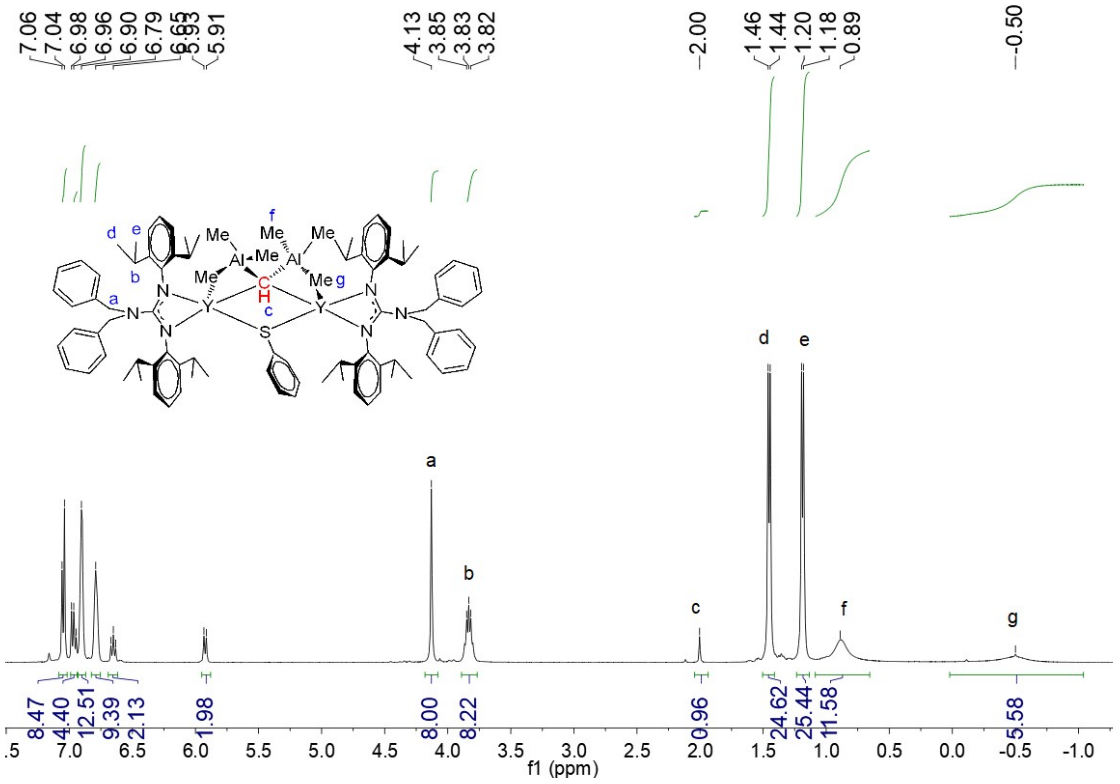

Fig. S7 ^{1}H NMR spectrum of **3** obtained in C_6D_6 at room temperature.

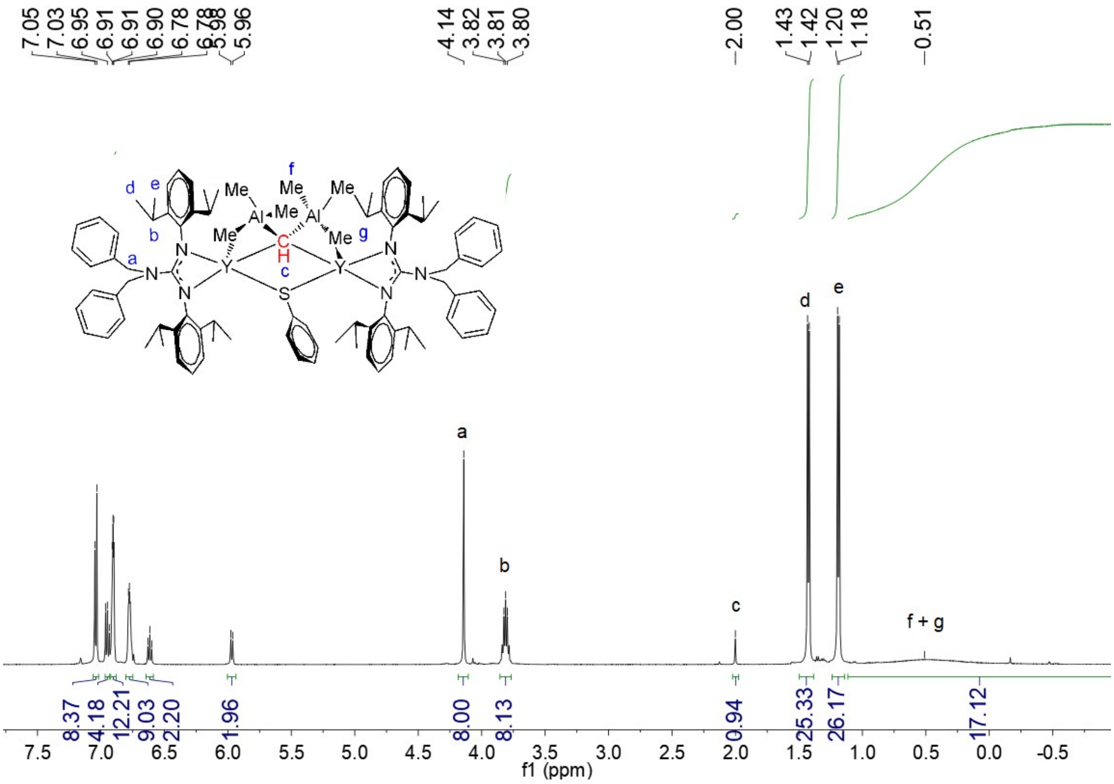

Fig. S8 ^{1}H NMR spectrum of **3** obtained in C_6D_6 at 60 ^{o}C.

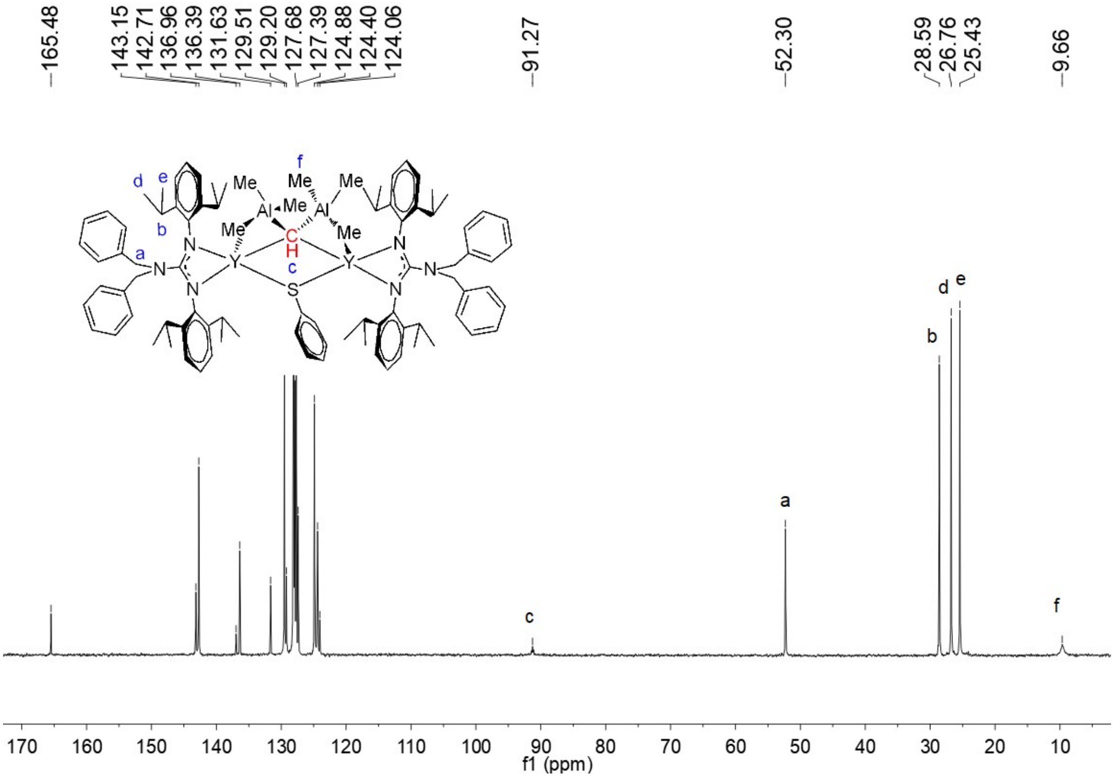

Fig. S9 $^{13}C\{^{1}H\}$ NMR spectrum of **3** obtained in C_6D_6 at room temperature.

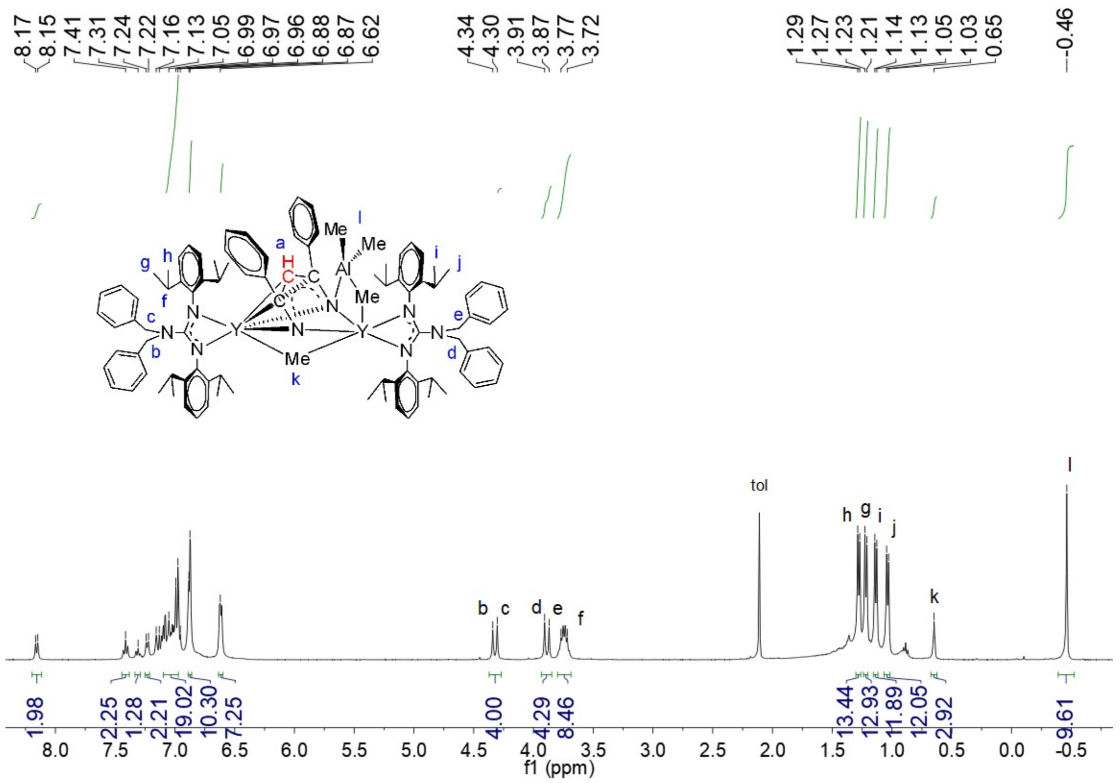

Fig. S10 ^{1}H NMR spectrum of **4** obtained in C_6D_6 at room temperature.

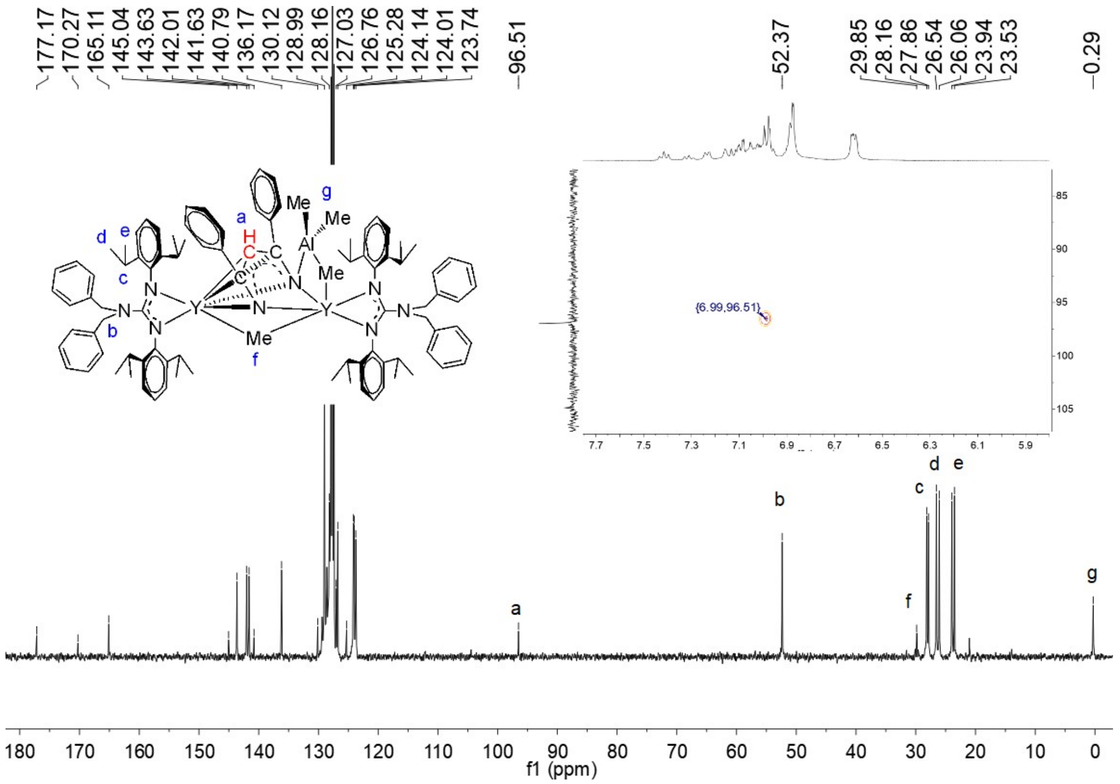

Fig. S11 $^{13}C\{^{1}H\}$ NMR spectrum of **4** obtained in C_6D_6 at room temperature.

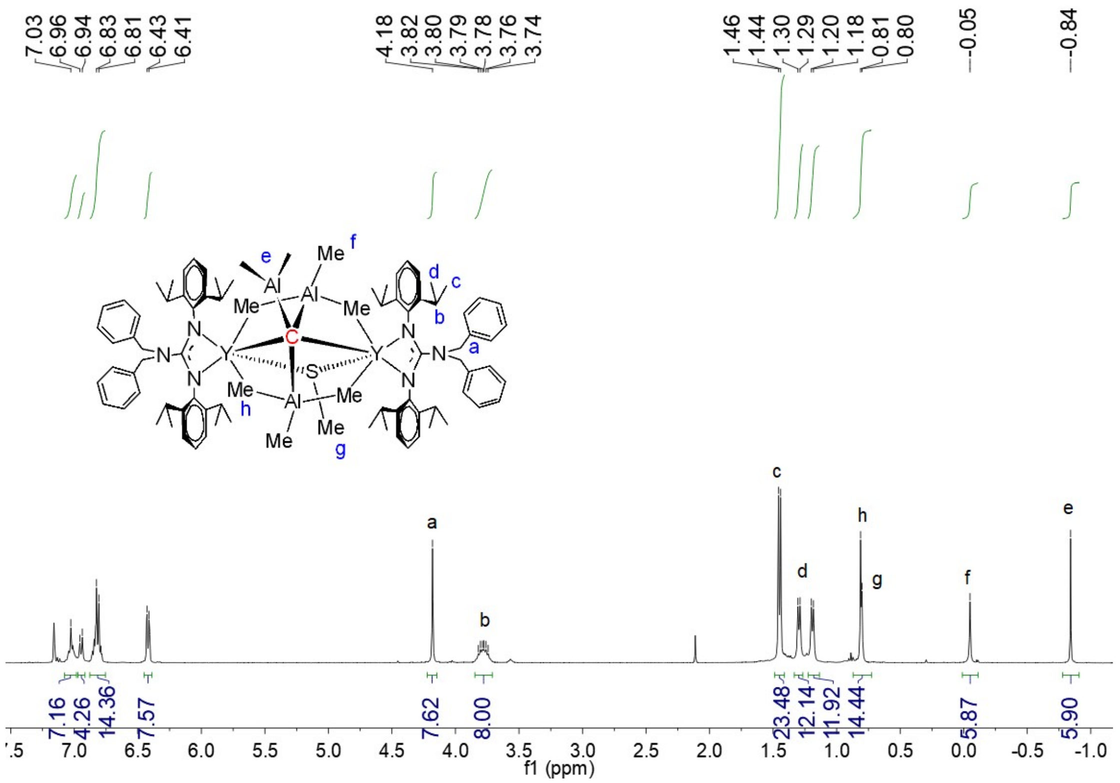

Fig. S12 ^{1}H NMR spectrum of **5** obtained in C_6D_6 at room temperature.

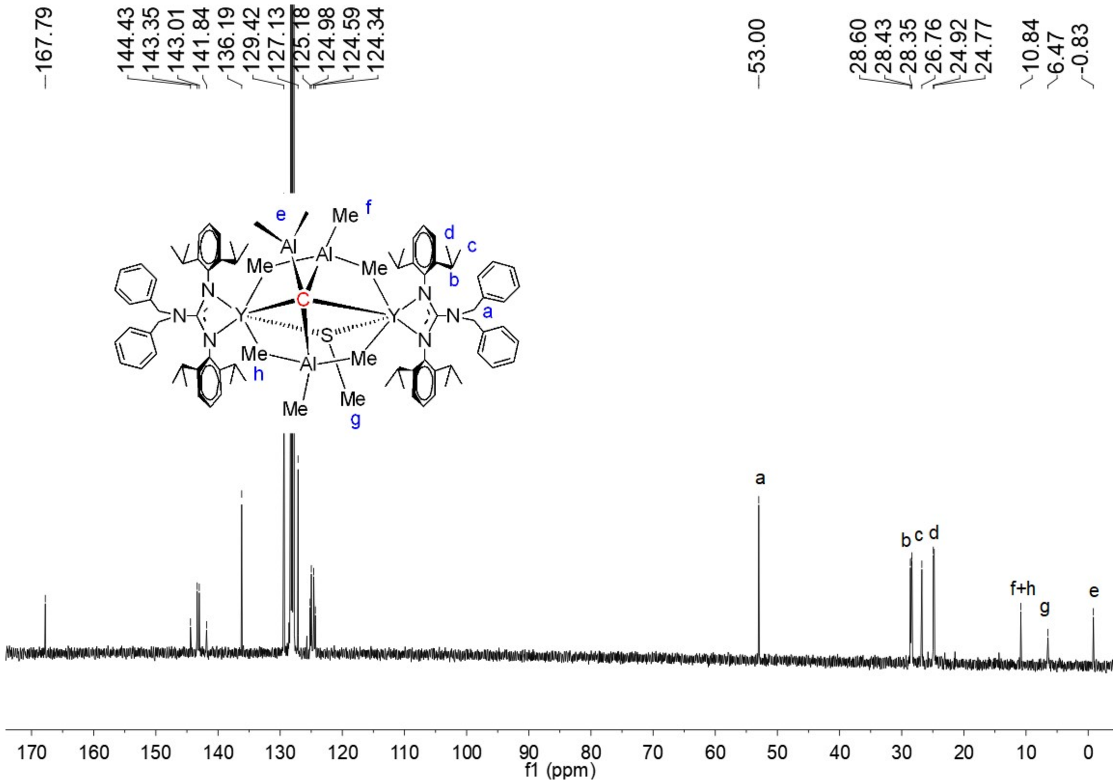

Fig. S13 $^{13}C\{^{1}H\}$ NMR spectrum of **5** obtained in C_6D_6 at room temperature.

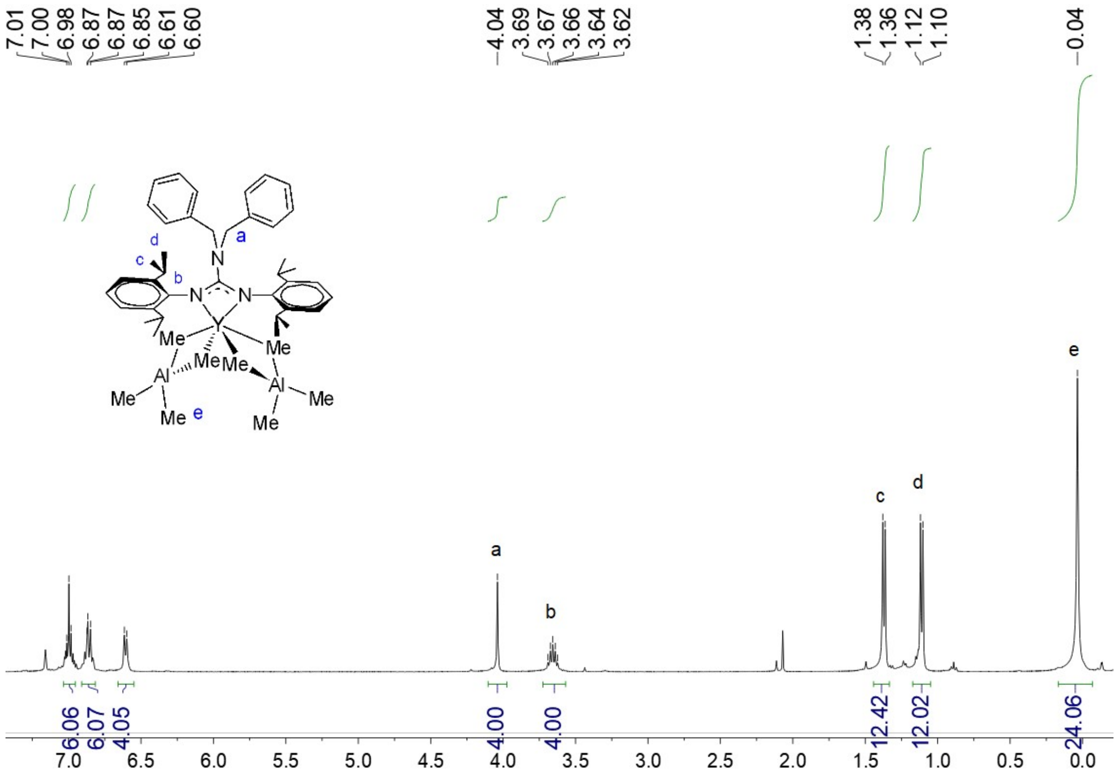

Fig. S14 ^{1}H NMR spectrum of **6** obtained in C_6D_6 at room temperature.

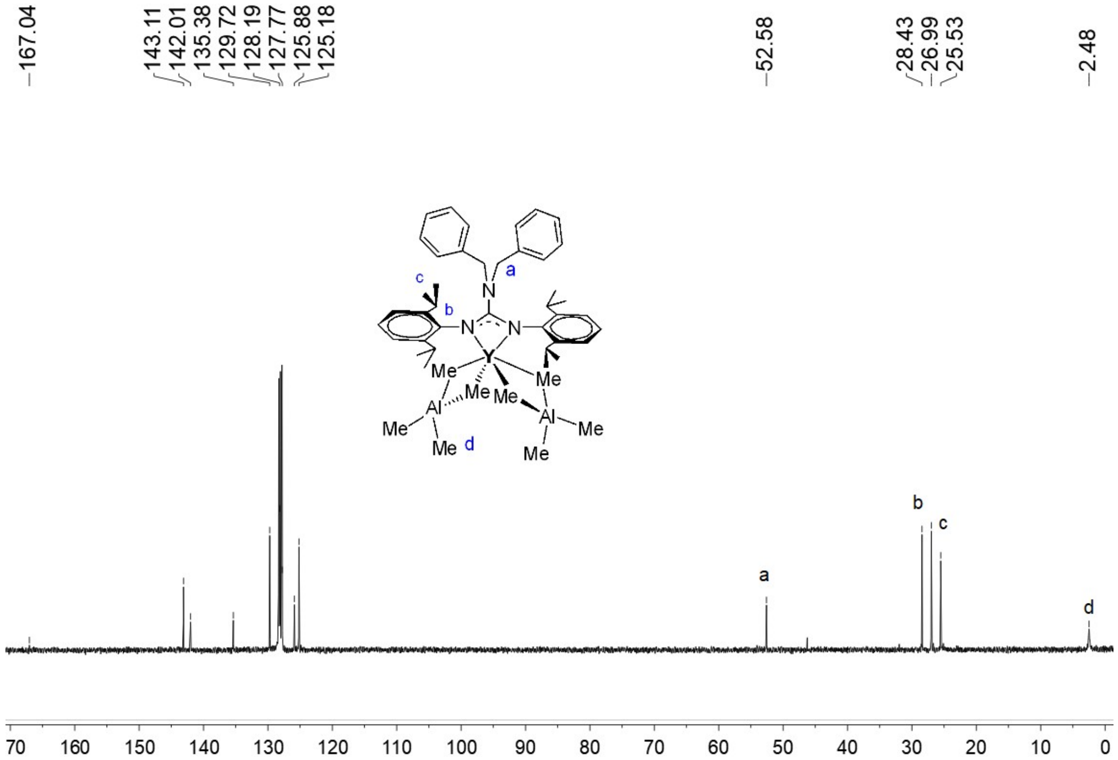

Fig. S15 $^{13}C\{^{1}H\}$ NMR spectrum of **6** obtained in C_6D_6 at room temperature.

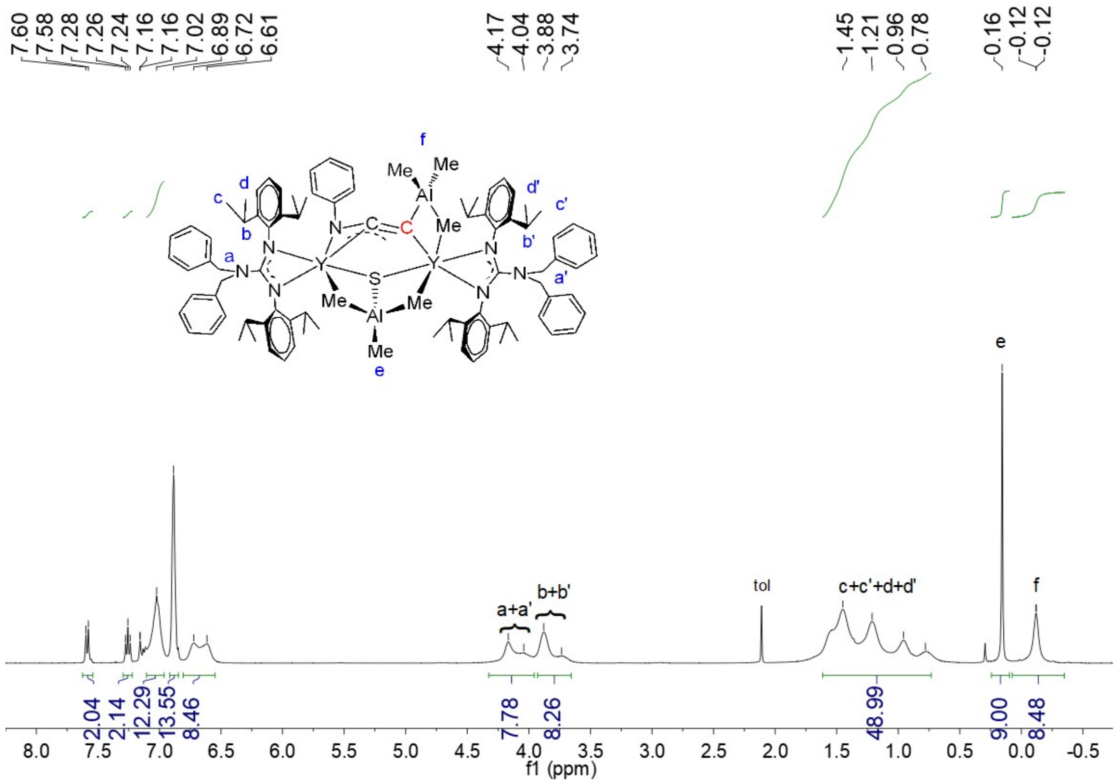

Fig. S16 ^{1}H NMR spectrum of **7** obtained in C_6D_6 at room temperature.

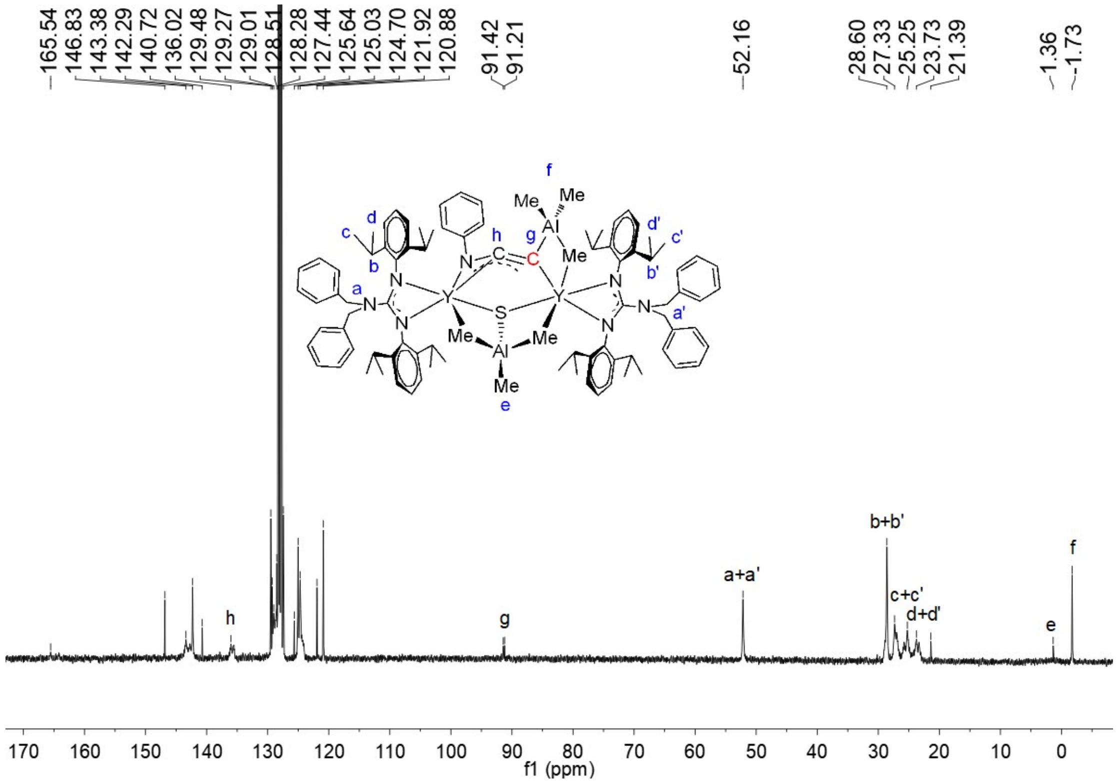

Fig. S17 $^{13}C\{^{1}H\}$ NMR spectrum of **7** obtained in C_6D_6 at room temperature.

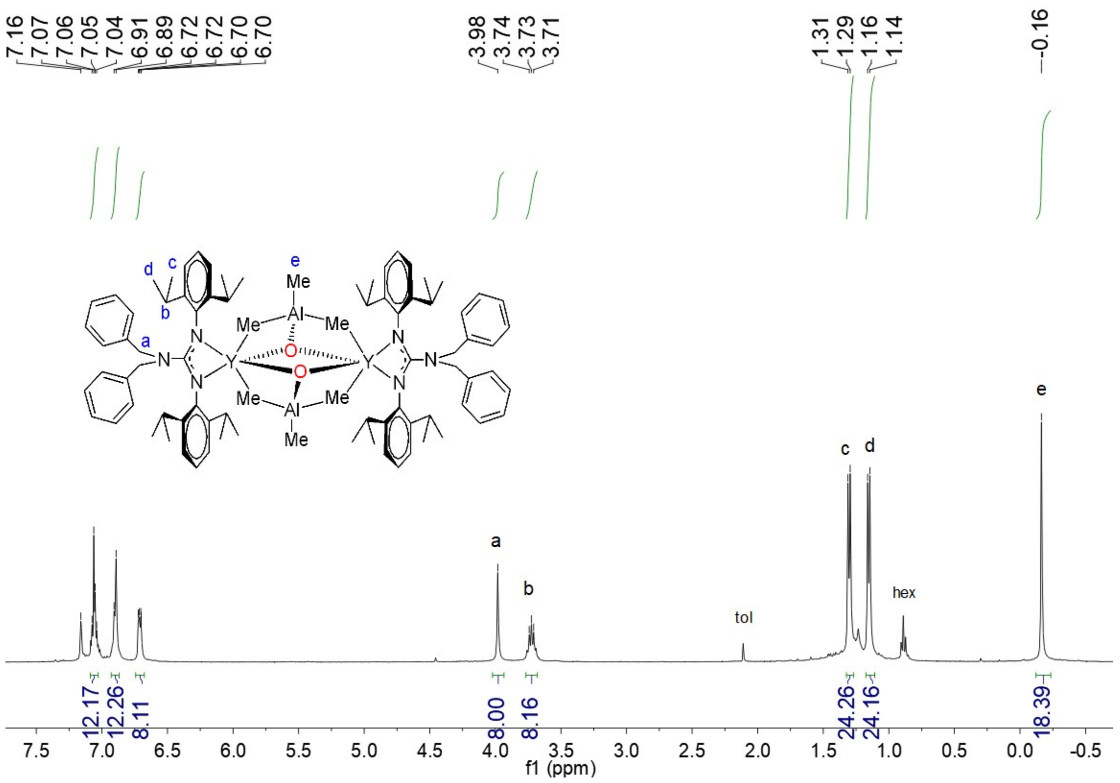

Fig. S18 ^{1}H NMR spectrum of **8** obtained in C_6D_6 at room temperature.

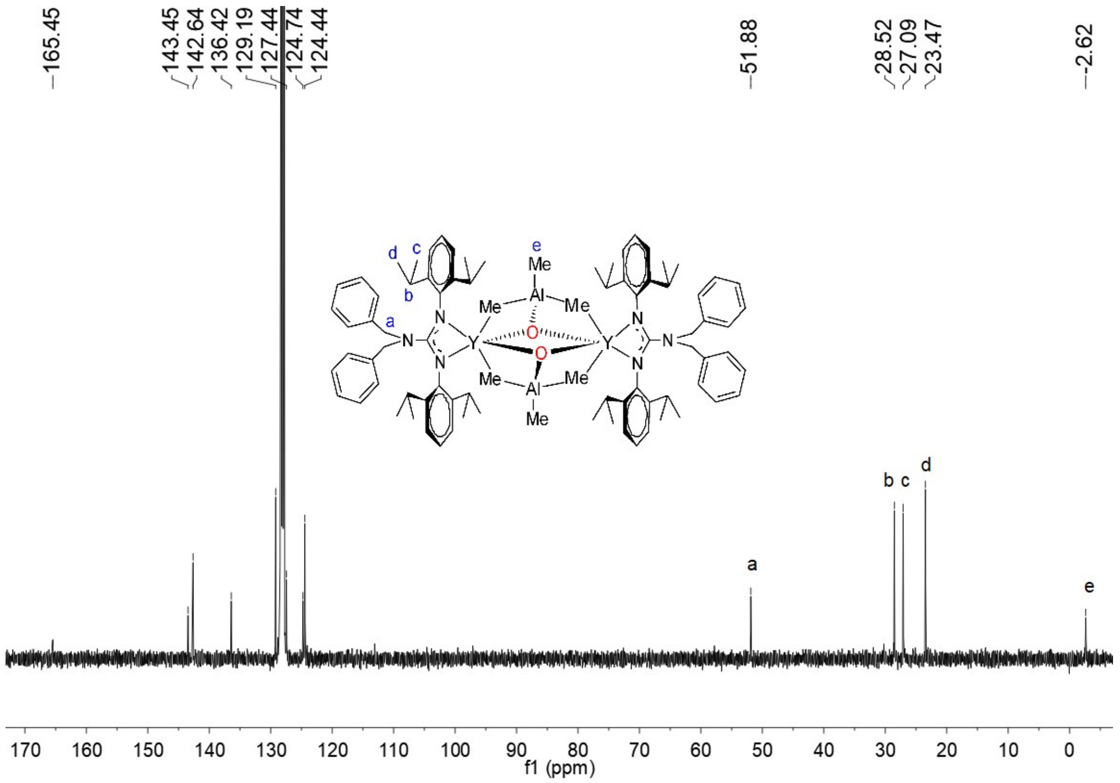

Fig. S19 $^{13}C\{^{1}H\}$ NMR spectrum of **8** obtained in C_6D_6 at room temperature.

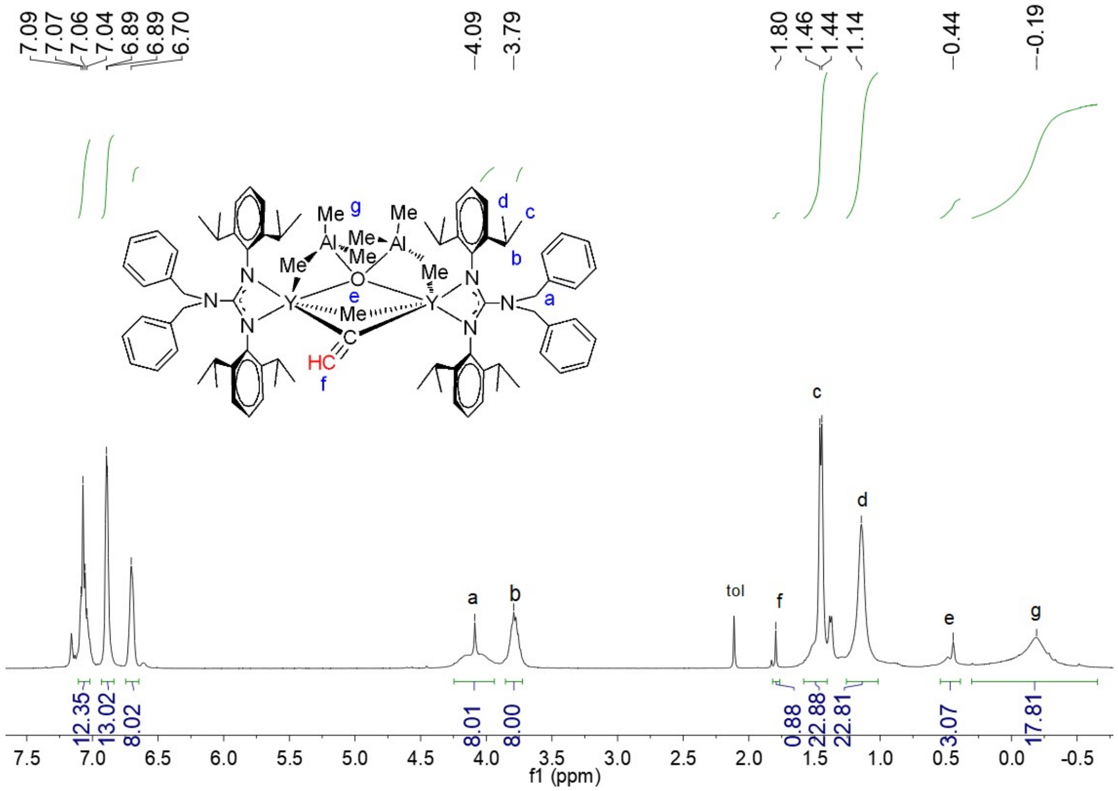

Fig. S20 ^{1}H NMR spectrum of **9** obtained in C_6D_6 at room temperature.

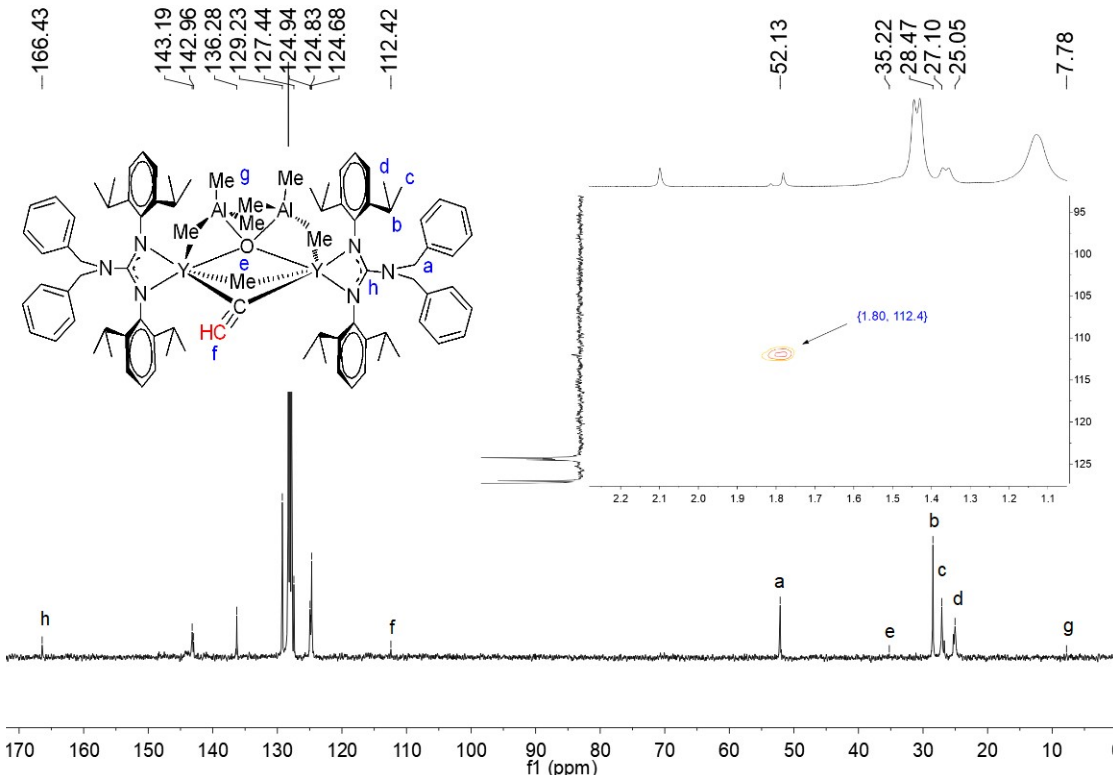

Fig. S21 $^{13}C\{^{1}H\}$ NMR spectrum of **9** obtained in C_6D_6 at room temperature.

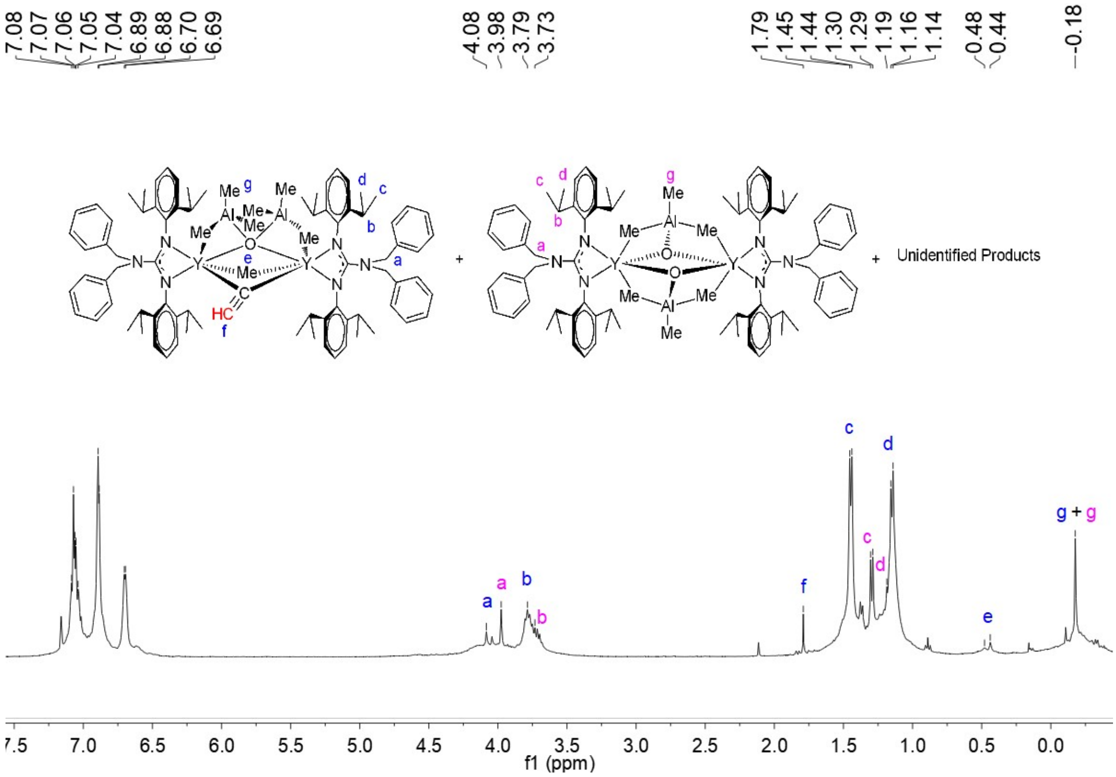

Fig. S22 The *in situ* ^{1}H NMR of complex **1** with CO obtained in C_6D_6 at room temperature.

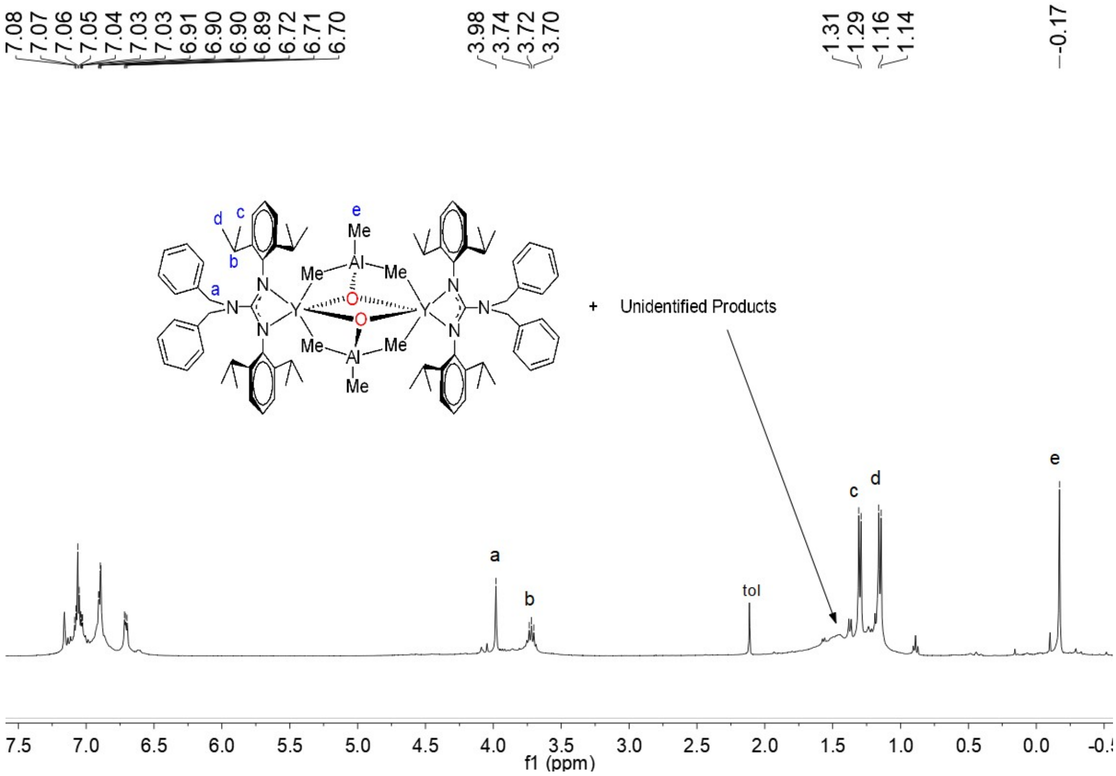

Fig. S23 The ^{1}H NMR of the reaction solution of **9** with CO (carried out in Young tube at 50 ^{o}C for 10 h) obtained in C_6D_6 at room temperature.

X-ray Crystallographic structure determinations

All crystals for X-ray analysis were obtained as described in the preparations. In the glovebox, the suitable crystals were sealed in dried immersion oil. Data collections were performed on a Bruker SMART APEX or Bruker SMART APEX II (at 173 K or 298 K) diffractometer with CCD area detector using graphite-monochromated Mo/Ga Kα radiation (λ = 0.71073 Å / λ = 1.34138 Å). The determination of crystal class and unit cell was carried out by using the SMART program package. The raw frame data were processed using SAINT[1] and SADABS[2] to yield the reflection data file. The structure was solved by using the SHELXTL program[3] Refinement was performed on F^2 anisotropically by the full-matrix least-squares method for all the non-hydrogen atoms. The analytical scattering factors for neutral atoms were used throughout the analysis. Except for the hydrogen atoms on bridging carbons, hydrogen atoms were placed at the calculated positions and included in the structure calculation without further refinement of the parameters. The hydrogen atoms on bridging carbons were located by difference Fourier syntheses and their coordinates and isotropic parameters were refined. The residual electron densities were of no chemical significance. Crystal data, data collection, and processing parameters: 2189939 (for **1**), 2189941 (for **3**), 2189946 (for **4**), 2189943 (for **5**), 2262169 (for **6**), 2189944 (for **7**), 2189945 (for **8**) and 2189942 (for **9**) contain the supplementary crystallographic data for this paper. These data can be obtained free of charge from The Cambridge Crystallographic Data Centre via https://www.ccdc.cam.ac.uk/ Community/Depositastructure/ CSDCommunications/

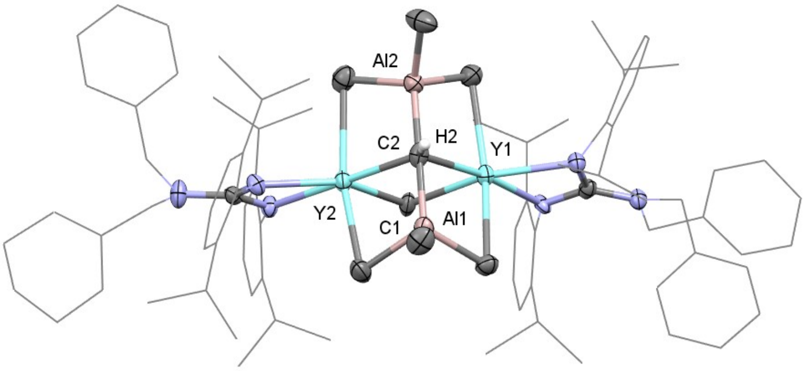

Fig. S24 Molecular structure of complex **1** with thermal ellipsoids at 30% probability except for the 2,6-(^{i}Pr)$_2$C$_6$H$_3$ groups and benzyl groups in the guanidinate ligand. All hydrogen atoms (except for H2) are omitted for clarity. Selected bond lengths (Å) and angles (deg): Y(1)−C(1) 2.528(6), Y(1)−C(2) 2.418(5), Y(2)−C(1) 2.543(5), Y(2)−C(2) 2.398(5), C(2)−Al(1) 1.974(5), C(2)−Al(2) 1.970(5), Y(1)−Al(1) 3.083(17), Y(1)−Al(2) 3.057(19); C(1)−Y(1)−C(2) 85.47(15), C(1)−Y(2)−C(2) 85.55(15), Y(1)−C(1)−Y(2) 91.29(17), Y(1)−C(2)−Y(2) 97.65(16).

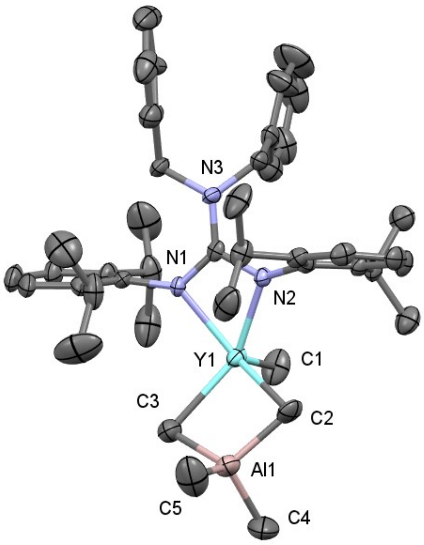

Fig. S25 Molecular structure of complex **2** with thermal ellipsoids at 30% probability. All hydrogen atoms are omitted for clarity.

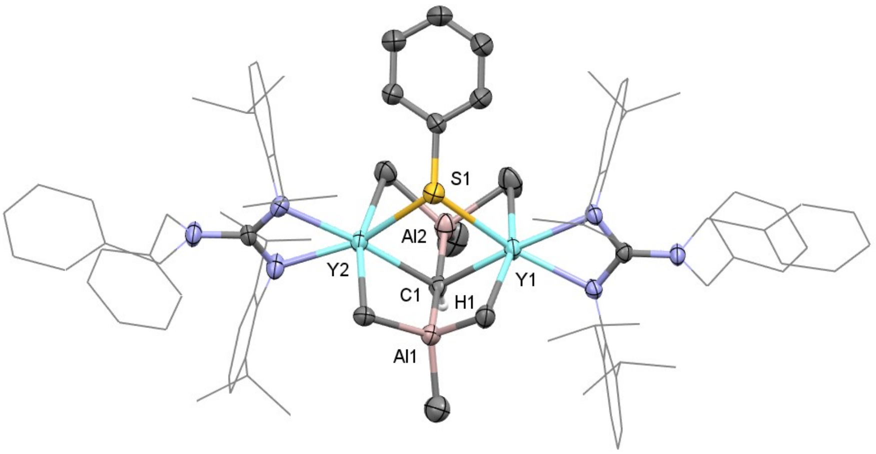

Fig. S26 Molecular structures of **3** with thermal ellipsoids at 30% probability except for the 2,6-(^{i}Pr)$_2$C$_6$H$_3$ groups and benzyl groups in the guanidinate ligand. All hydrogen atoms (except for H2) are omitted for clarity. Selected bond lengths (Å) and angles (deg): Y(1)−C(1) 2.451(4), Y(1)−S(1) 2.799(12), Y(2)−C(1) 2.469(4), Y(2)−S(1) 2.821(13), C(1)−Al(1) 1.984(5), C(1)−Al(2) 1.979(5), Y(1)−Al(1) 3.051(15), Y(1)−Al(2) 3.050(17); C(1)−Y(1)−S(1) 82.81(10), C(1)−Y(2)−S(1) 81.04(11), Y(1)−C(1)−Y(2) 106.19(17), Y(1)−S(1)−Y(2) 88.85(4).

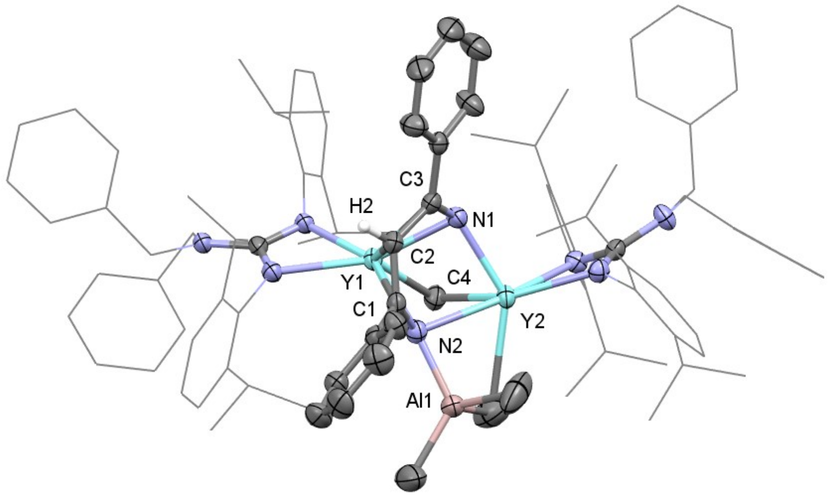

Fig. S27 Molecular structures of **4** with thermal ellipsoids at 30% probability except for the 2,6-(^{i}Pr)$_2$C$_6$H$_3$ groups and benzyl groups in the guanidinate ligand. All hydrogen atoms (except for H2) are omitted for clarity. Selected bond lengths (Å) and angles (deg): Y(1)−C(1) 2.724(4), Y(1)−C(2) 2.649(4), Y(1)−C(3) 2.665(4), Y(1)−C(4) 2.434(5), Y(1)−N(1) 2.315(3), Y(1)−N(2) 2.399(3), Y(2)−N(1) 2.241(4), Y(2)−N(2) 2.528(3), Y(2)−C(4) 2.555(5), N(1)−C(3) 1.304(5), C(2)−C(3) 1.447(6), C(1)−C(2) 1.431(6), C(1)−N(2) 1.334(5), N(2)−Al(1) 1.922(4); C(2)−Y(1)−C(4) 130.41(14), N(1)−Y(1)−N(2) 75.15(12), Y(1)−N(1)−Y(2) 89.86(13), Y(1)−N(2)−Y(2) 81.52(10), Y(1)−C(4)−Y(2) 80.31(15).

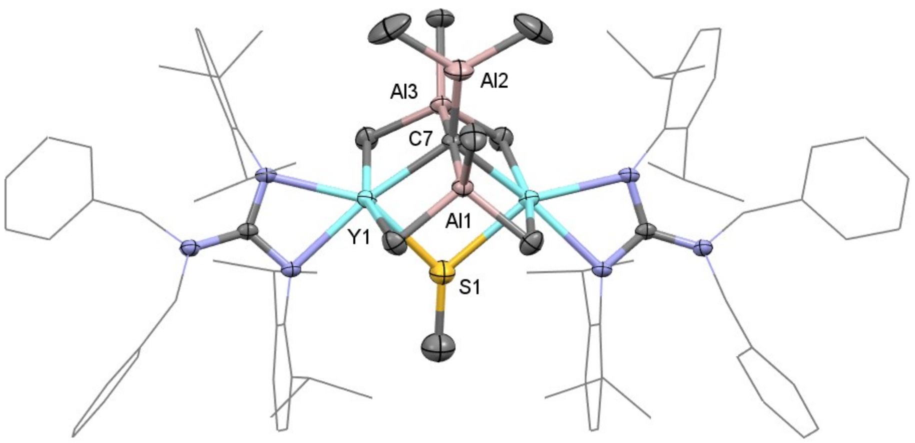

Fig. S28 Molecular structures of **5** with thermal ellipsoids at 30% probability except for the 2,6-(^{i}Pr)$_2$C$_6$H$_3$ groups and benzyl groups in the guanidinate ligand. All hydrogen atoms are omitted for clarity. Selected bond lengths (Å) and angles (deg): Y(1)–C(7) 2.434(3), Y(1)–S(1) 2.673(13), C(7)–Al(1) 1.984(6), C(7)–Al(2) 1.935(6), C(7)–Al(3) 1.963(6); C(7)–Y(1)–S(1) 84.07(10), Y(1)–C(7)–Y(1A) 101.87(19), Y(1)–S(1)–Y(1A) 89.97(6), Al(1)–C(7)–Al(2) 89.3(2), Al(2)–C(7)–Al(3) 98.4(2), Al(1)–C(7)–Al(3) 172.3(3).

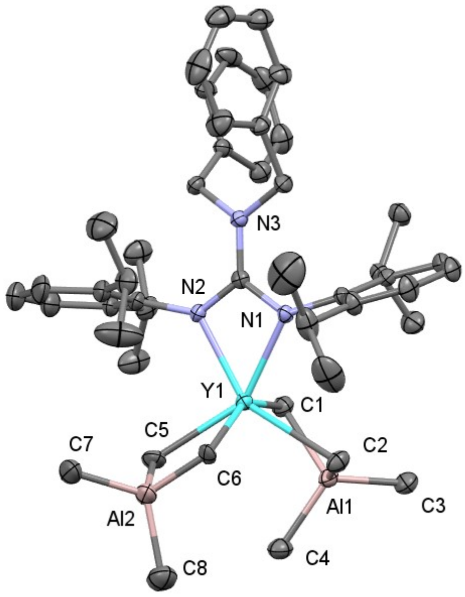

Fig. S29 Molecular structures of **6** with thermal ellipsoids at 30% probability. All hydrogen atoms are omitted for clarity. Selected bond lengths (Å) and angles (deg): Y(1)–C(1) 2.556(7), Y(1)–C(2) 2.567(7), Y(1)–C(5) 2.573(9), Y(1)–C(6) 2.268(5), Y(1)–N(1) 2.323(5), Y(1)–N(2) 2.296(4); C(1)–Y(1)–C(2) 79.9(2), C(1)–Y(1)–C(5) 87.9(3), C(1)–Y(1)–C(6) 145.5(2), N(1)–Y(1)–C(1) 100.4(2), N(2)–Y(1)–C(1) 95.2(2).

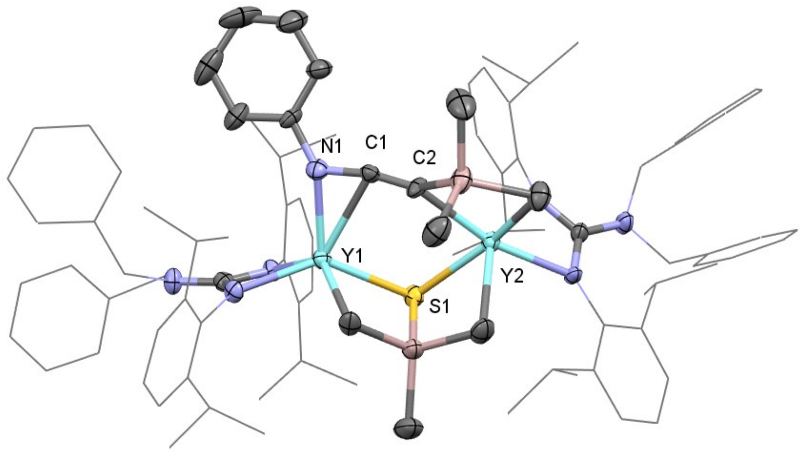

Fig. S30 Molecular structures of **7** with thermal ellipsoids at 30% probability except for the 2,6-(^{i}Pr)$_2$C$_6$H$_3$ groups and benzyl groups in the guanidinate ligand. All hydrogen atoms are omitted for clarity. Selected bond lengths (Å) and angles (deg): Y(1)−N(1) 2.323(5), Y(1)−C(1) 2.627(6), C(2)−C(1) 1.221(8), N(1)−C(1) 1.329(8), Y(2)−C(2) 2.499(6), Y(1)−S(1) 2.665(16), Y(2)−S(1) 2.654(17), C(2)−Al(2) 2.041(6), Al(1)−S(1) 2.286(3); C(1)−Y(1)−S(1) 93.18(14), C(2)−Y(2)−S(1) 87.83(15), Y(1)−S(1)−Y(2) 103.27(6), C(2)−C(1)−N(1) 167.6(6).

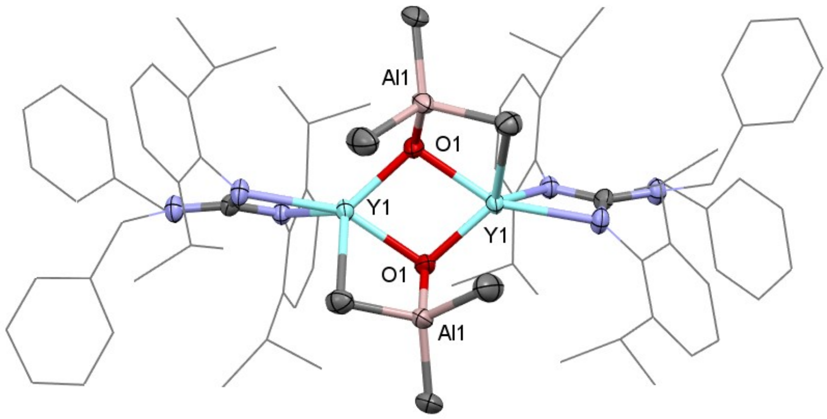

Fig. S31 Molecular structures of **8** with thermal ellipsoids at 30% probability except for the 2,6-(^{i}Pr)$_2$C$_6$H$_3$ groups and benzyl groups in the guanidinate ligand. All hydrogen atoms are omitted for clarity. Selected bond lengths (Å) and angles (deg): Y(1)−O(1) 2.207(2), Y(1A)−O(1) 2.122(3), O(1)−Al(1) 1.780(3), Y(1)−Y(1A) 3.389(8); O(1)−Y(1)−O(1A) 76.36(10), Y(1)−O(1)−Y(1A) 103.03(10), Y(1)−O(1)−Al(1) 103.39(12), Y(1A)−O(1)−Al(1) 114.16(14).

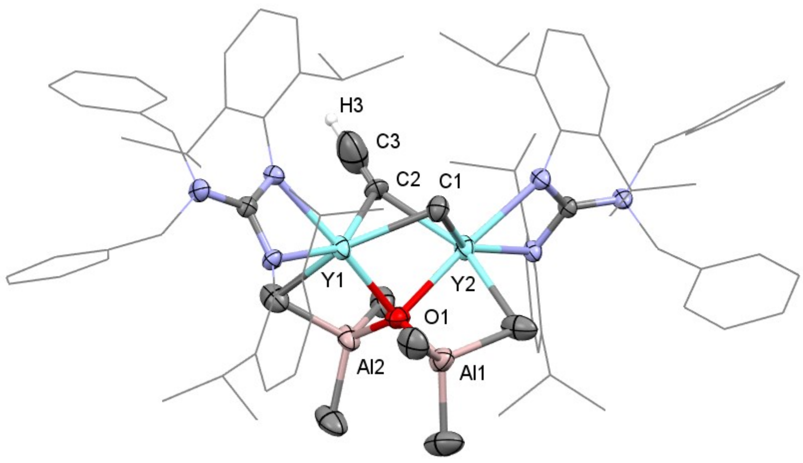

Fig. S32 Molecular structures of **9** with thermal ellipsoids at 30% probability except for the 2,6-(^{i}Pr)$_2$C$_6$H$_3$ groups and benzyl groups in the guanidinate ligand. All hydrogen atoms (except for H3) are omitted for clarity. Selected bond lengths (Å) and angles (deg): Y(1)−C(1) 2.493(6), Y(1)−C(2) 2.537(6), Y(1)−O(1) 2.331(4), Y(2)−O(1) 2.328(4), Y(2)−C(1) 2.591(5), Y(2)−C(2) 2.440(6), C(2)−C(3) 1.065(12); C(1)−Y(1)−C(2) 73.34(19), C(1)−Y(2)−C(2) 73.26(19), Y(1)−C(1)−Y(2) 81.99(17), Y(1)−C(2)−Y(2) 84.13(18), Y(1)−O(1)−Y(2) 91.44(12), Y(1)−C(2)−C(3) 118.1(7), Y(2)−C(2)−C(3) 155.1(7), Y(1)−O(1)−Al(1) 113.09(18), Y(1)−O(1)−Al(2) 99.56(16).

Table S1. Crystal data and refinement details of complexes **1, 3, 4**

	**1**	**3**	**4·tol**
Formula	$C_{86}H_{118}Al_2N_6Y_2$	$C_{91}H_{120}Al_2N_6SY_2$	$C_{104}H_{124}AlN_8Y_2$
Molecular weight	1467.64	1561.76	1690.90
Crystal system	Triclinic	Monoclinic	Triclinic
Space group	P-1	$P2_1/c$	P-1
a /(Å)	13.575(5)	18.210(3)	12.163(5)
b/ (Å)	18.655(7)	22.124(3)	18.782(6)
c/ (Å)	19.146(8)	26.122(4)	22.351(8)
V/ ($Å^3$)	4199(3)	10091(3)	4574.5(3)
Z	2	4	2
ρ_c /(mg. m^{-3})	1.161	1.028	1.228
μ(Mo-K_α)/(mm^{-1})	1.440	1.222	1.444
Limiting indices	-16<=h<=14, -20<=k<=22, -22<=l<=20	-20<=h<=21, -26<=k<=21, -30<=l<=31	-14<=h<=14 -22<=k<=22 -26<=l<=26
Collected reflections	17561	49629	145718
Unique	14597 [R(int) = 0.0445]	17884 [R(int) = 0.0606]	16178 [R(int) = 0.2260]
Parameters	865	962	1067
Goodness of fit on F^2	0.859	0.918	0.996
$R_1{}^a, wR_2{}^a[I > 2\sigma(I)]$	R_1 = 0.0537 wR_2 = 0.1115	R_1 = 0.0634 wR_2 = 0.1601	R_1 = 0.0565, wR_2 = 0.1303
R_1, wR_2indices (all data)	R_1 = 0.1301 wR_2 = 0.1341	R_1 = 0.1124 wR_2 = 0.1792	R_1 = 0.0987 wR_2 = 0.1529
Max/min residual density(e $Å^{-3}$)	1.16 and -0.84	0.95 and -1.84	0.58 and -1.70

[a] R1 = ∑|| F0|-|F_c|| (based on reflections with $F_o^2 > 2\sigma F^2$). $wR_2 = [\sum [w(F_o^2-F_c^2)^2]/ \sum[w(F_o^2)^2]]^{1/2}$; $w = 1/[\sigma^2(F_o^2) + (0.095P)^2]$; $P = [\max(F_o^2, 0) +2F_c^2]/3$ (also with $F_o^2 > 2\sigma F^2$).

Table S2. Crystal data and refinement details of complexes **5**, **6** and **7**

	**5**	**6**	**7**
Formula	$C_{88}H_{123}Al_3N_6SY_2$	$C_{47}H_{72}Al_2N_3Y$	$C_{92}H_{119}Al_2N_7SY_2$
Molecular weight	1555.74	821.98	1586.77
Crystal system	Monoclinic	Orthorhombic	Monoclinic
Space group	$P2_1/m$	$P2_12_12_1$	C2/c
a /(Å)	10.482(6)	11.072(3)	69.627(11)
b/ (Å)	36.766(2)	12.107(3)	13.874(2)
c/ (Å)	11.947(7)	36.431(9)	21.791(3)
V/ ($Å^3$)	4263.8(4)	4883.3(2)	20793(6)
Z	2	4	8
ρ_c /(mg. m^{-3})	1.212	1.118	1.014
$\mu(Mo\text{-}K_\alpha)/(mm^{-1})$	1.784	1.498	1.187
Limiting indices	-12<=h<=12<br>-43<=k<=43<br>-14<=l<=14	-21<=h<=13,<br>-14<=k<=14,<br>-43<=l<=42	-82<=h<=82<br>-16<=k<=16<br>-25<=l<=22
Collected reflections	77256	29693	59613
Unique	7663 [R(int) = 0.0963]	8569 [R(int) = 0.0565]	18380 [R(int) = 0.0764]
Parameters	499	528	974
Goodness of fit on F^2	1.066	1.055	1.011
$R_1{}^a, wR_2{}^a[I > 2\sigma(I)]$	R_1 = 0.0528<br>wR_2 = 0.1444	R_1 = 0.0465<br>wR_2 = 0.1273	R_1 = 0.0855<br>wR_2 = 0.2399
R_1, wR_2indices (all data)	R_1 = 0.0621<br>wR_2 = 0.1515	R_1 = 0.0529<br>wR_2 = 0.1318	R_1 = 0.1247<br>wR_2 = 0.2649
Max/min residual density(e $Å^{-3}$)	0.69 and -0.98	2.08 and -0.49	1.04 and -1.13

[a] R1 = ∑| | F0|-|F_c| | (based on reflections with $F_o{}^2 > 2\sigma F^2$). $wR_2 = [\sum [w(F_o{}^2-F_c{}^2)^2]/ \sum[w(F_o{}^2)^2]]^{1/2}$; $w = 1/[\sigma^2(F_o{}^2) + (0.095P)^2]$; $P = [\max(F_o{}^2, 0) +2F_c{}^2]/3$ (also with $F_o{}^2 > 2\sigma F^2$).

Table S3. Crystal data and refinement details of complexes **8** and **9**

	**8**	**9**
Formula	$C_{84}H_{114}Al_2N_6O_2Y_2$	$C_{87}H_{118}Al_2N_6OY_2$
Molecular weight	1471.58	1495.65
Crystal system	Monoclinic	Monoclinic
Space group	C2/c	C2/c
a /(Å)	35.057(6)	44.077(4)
b/ (Å)	11.675(2)	13.425(13)
c/ (Å)	20.542(4)	29.287(3)
V/ ($Å^3$)	8289(2)	17291(3)
Z	8	8
ρ_c /(mg. m^{-3})	1.179	1.149
μ(Mo-K_α)/(mm^{-1})	1.461	1.401
Limiting indices	-45<=h<=36<br>-14<=k<=14<br>-24<=l<=26	-44<=h<=55<br>-16<=k<=16<br>-36<=l<=36
Collected reflections	23273	54916
Unique	9016<br>[R(int) = 0.0433]	15258<br>[R(int=0.0802]
Parameters	452	927
Goodness of fit on F^2	0.988	0.930
$R_1{}^a$,$wR_2{}^a$[I >2σ(I)]	R_1 = 0.0561<br>wR_2 = 0.1468	R_1 = 0.0645<br>wR_2 = 0.1657
R_1, wR_2indices (all data)	R_1 = 0.1002<br>wR_2 = 0.1702	R_1 = 0.1240<br>wR_2 = 0.1903
Max/min residual density(e $Å^{-3}$)	0.79 and -0.48	0.86 and -0.56

[a] R1 = ∑| | F0|-|F_c| | (based on reflections with $F_o{}^2 > 2\sigma F^2$). wR_2 = [∑ [w($F_o{}^2$-$F_c{}^2$)^2]/ ∑[w($F_o{}^2$)^2]]$^{1/2}$; w = 1/[σ^2($F_o{}^2$) + (0.095P)^2]; P = [max($F_o{}^2$, 0) +2$F_c{}^2$]/3 (also with $F_o{}^2 > 2\sigma F^2$).

Proposed reaction pathway towards complex **7**.

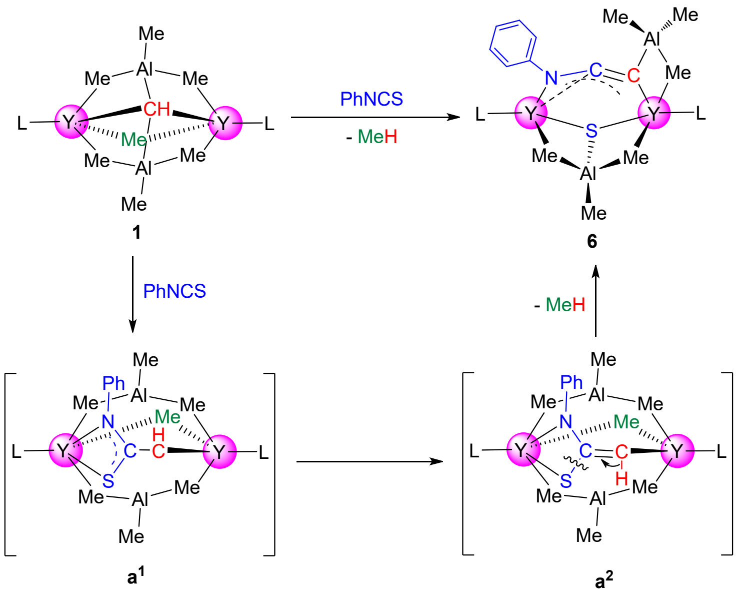

Fig. S33 Proposed reaction pathway for formation of complex **7.**

Computational Details.

All DFT calculations were performed with Gaussian 09.[4] Geometries were fully optimized in gas phase without symmetry constraints, employing the B3PW91 functional[5] and the Stuttgart effective core potential for Y[6] and Al[7] augmented with a polarization functions (ζf = 1.000 for Y and ζd = 0.325 for Al). For the other elements (N, H, O and C), Pople's double-ζ basis set 6-31G(d,p)[8] was used. Calculations of vibrational frequencies were systematically done in order to characterize the nature of stationary points. Dispersion corrections were accounted for using the Grimme's D3 scheme.

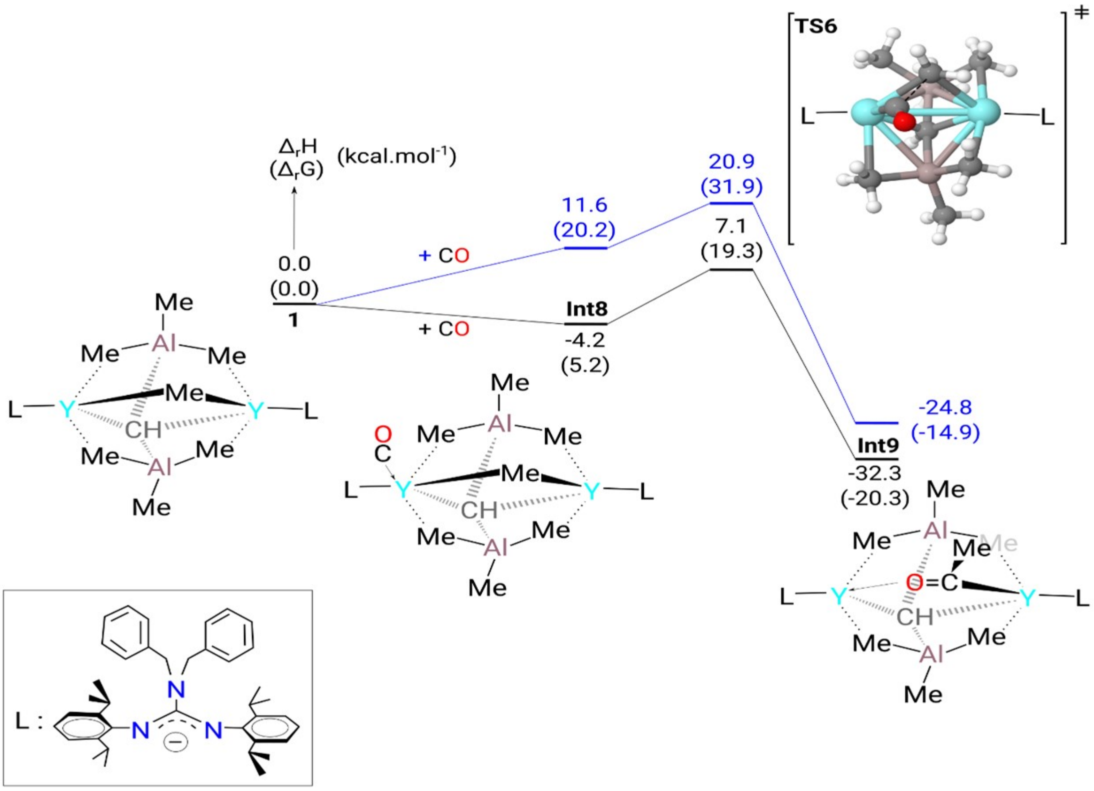

Fig. S34 Computed enthalpy (Gibbs free energy between bracket) for the alternative route (reaction of the methyl side) for the reaction of CO with complex **1**. The blue pathway does not include dispersion corrections while the black one does

Optimized geometries

2

CO without dispersion

C	-0.50648	-1.19461	1.60490
O	0.21272	-1.92291	2.10020

214

Complex 1 without dispersion

Y	0.85448	7.96768	5.30174
Y	2.73890	5.31418	3.66776
Al	-0.30168	5.56027	3.85205
Al	2.47180	5.74457	6.66055
N	0.97858	10.43627	5.29926
N	-0.67349	9.40030	6.41035
N	-0.48336	11.78197	6.71447
N	3.87426	3.34953	2.96973
N	4.44496	5.22557	1.88114
N	5.34950	3.10983	1.07522
C	2.68556	7.90178	3.53100
H	3.47491	7.62562	2.80867
H	2.02779	8.53060	2.90787
H	3.22472	8.61617	4.17892
C	1.00515	5.53462	5.34246
H	0.44424	4.79685	5.94750
C	-0.89927	7.52516	3.47007
H	-1.56800	8.02394	4.18705
H	-0.29270	8.29881	2.97276
H	-1.57934	7.19474	2.67352
C	0.67878	5.11209	2.06917
H	-0.24002	5.08604	1.46808
H	1.29747	5.85538	1.54380
H	1.12203	4.11790	1.89920
C	-1.97240	4.52769	4.07241
H	-2.63829	5.03287	4.78479
H	-1.80756	3.51565	4.45851
H	-2.53508	4.42558	3.13448
C	4.30774	5.66941	5.67067
H	4.88508	5.75619	6.60024
H	4.65208	6.52457	5.06914
H	4.70930	4.75002	5.21686

C	2.53341	7.71970	7.31473
H	1.68676	8.16523	7.85962
H	3.26137	7.50850	8.11002
H	3.02151	8.51610	6.73561
C	2.53134	4.48063	8.17799
H	2.73387	3.46105	7.82422
H	1.58870	4.43772	8.73592
H	3.32029	4.72673	8.90176
C	-0.06908	10.59228	6.13512
C	0.18395	13.06091	6.39890
H	0.40764	13.07259	5.33399
H	1.14254	13.12039	6.92479
C	-0.61905	14.29973	6.73804
C	-0.22861	15.11841	7.80341
H	0.62474	14.83073	8.41164
C	-0.92000	16.29355	8.09529
H	-0.59860	16.91539	8.92638
C	-2.01722	16.66879	7.32320
H	-2.55752	17.58424	7.54817
C	-2.41137	15.86680	6.25251
H	-3.25806	16.15715	5.63638
C	-1.71373	14.69702	5.96102
H	-2.02217	14.08678	5.11856
C	-1.20620	11.78374	7.98889
H	-1.77688	10.85962	8.07062
H	-1.94087	12.59390	7.95692
C	-0.35596	11.95194	9.23464
C	0.87599	11.31036	9.39024
H	1.27509	10.71301	8.57597
C	1.59453	11.42390	10.57981
H	2.55046	10.91719	10.68119
C	1.08825	12.17848	11.63689
H	1.64725	12.26542	12.56443
C	-0.14010	12.82205	11.49336

H	-0.54309	13.41673	12.30883
C	-0.85139	12.71245	10.30016
H	-1.80309	13.22745	10.19042
C	1.82576	11.39177	4.67383
C	3.06496	11.79690	5.23807
C	3.88171	12.68508	4.52983
H	4.82449	12.99698	4.97246
C	3.52590	13.17527	3.28146
H	4.17396	13.86990	2.75445
C	2.33415	12.74773	2.71171
H	2.05476	13.11014	1.72546
C	1.48458	11.85628	3.37137
C	3.57896	11.28606	6.57520
H	2.79940	10.64859	7.00411
C	3.87703	12.40797	7.58010
H	2.99529	13.01591	7.79777
H	4.66024	13.07881	7.21105
H	4.22723	11.98510	8.52775
C	4.84308	10.43212	6.38412
H	5.16231	9.99100	7.33503
H	5.67130	11.03998	6.00383
H	4.68442	9.62017	5.66861
C	0.21352	11.42352	2.65640
H	-0.27559	10.67535	3.28764
C	-0.76496	12.59094	2.46642
H	-1.03638	13.04940	3.42095
H	-1.68594	12.24892	1.98126
H	-0.32923	13.37480	1.83692
C	0.51252	10.76978	1.29935
H	1.23055	9.95029	1.39470
H	0.93475	11.49104	0.59099
H	-0.40642	10.37172	0.85476
C	-2.00661	9.13559	6.82464
C	-3.14554	9.50332	6.04891

C	-4.41002	9.05121	6.44054
H	-5.27355	9.33644	5.84532
C	-4.59655	8.24503	7.55446
H	-5.58908	7.89523	7.82431
C	-3.49202	7.90714	8.32362
H	-3.62548	7.29312	9.21061
C	-2.20792	8.34800	7.99466
C	-3.08667	10.40724	4.82591
H	-2.03556	10.56542	4.56680
C	-3.71203	11.77391	5.14281
H	-3.22722	12.25246	5.99595
H	-4.77609	11.66551	5.38145
H	-3.63481	12.44519	4.27974
C	-3.78697	9.81011	3.59491
H	-3.63216	10.45733	2.72448
H	-4.86840	9.72982	3.74780
H	-3.41083	8.81523	3.34805
C	-1.06988	7.98944	8.93509
H	-0.18808	8.53893	8.59102
C	-1.34707	8.43529	10.37838
H	-1.57318	9.50374	10.43603
H	-0.47028	8.24398	11.00643
H	-2.18905	7.88775	10.81610
C	-0.75261	6.48946	8.89385
H	-0.49914	6.15184	7.88306
H	-1.61340	5.89933	9.22869
H	0.09106	6.25298	9.55076
C	4.59872	3.88578	1.94566
C	5.05100	1.69028	0.86999
H	4.56826	1.29785	1.76338
H	6.00166	1.15570	0.77832
C	4.19248	1.35973	-0.33622
C	4.47630	0.20783	-1.07887
H	5.34929	-0.38867	-0.82389

C	3.66122	-0.17946	-2.14058
H	3.89898	-1.07819	-2.70340
C	2.54981	0.58855	-2.48446
H	1.91420	0.29199	-3.31417
C	2.26114	1.74147	-1.75615
H	1.39670	2.34668	-2.01547
C	3.07476	2.12132	-0.68928
H	2.83537	3.01694	-0.12321
C	6.13801	3.71786	-0.01498
H	6.56968	4.64577	0.35373
H	5.47906	3.98476	-0.84880
C	7.25606	2.84987	-0.55413
C	7.16899	2.31560	-1.84403
H	6.27061	2.48498	-2.43140
C	8.21585	1.56849	-2.38279
H	8.12797	1.16472	-3.38775
C	9.36910	1.34134	-1.63517
H	10.18642	0.75960	-2.05249
C	9.47186	1.87390	-0.35012
H	10.37195	1.71224	0.23685
C	8.42638	2.62558	0.18097
H	8.52051	3.04498	1.17741
C	4.05319	2.11750	3.65639
C	5.22049	1.82698	4.41959
C	5.25992	0.65870	5.18805
H	6.15480	0.44276	5.76609
C	4.19067	-0.22372	5.24613
H	4.24283	-1.11598	5.86378
C	3.05785	0.05196	4.49337
H	2.21850	-0.63814	4.51976
C	2.97180	1.19116	3.68946
C	6.46142	2.70608	4.43560
H	6.23252	3.62981	3.89445
C	7.62749	2.01441	3.71464

H	7.36431	1.74901	2.68884
H	7.91296	1.09339	4.23500
H	8.50874	2.66588	3.68845
C	6.89988	3.08644	5.85925
H	7.69886	3.83532	5.82274
H	7.29408	2.21879	6.39889
H	6.07603	3.49416	6.44926
C	1.71750	1.38011	2.85343
H	1.90751	2.22095	2.17840
C	1.40523	0.15696	1.97922
H	2.25271	-0.11425	1.34364
H	0.55146	0.36935	1.32670
H	1.14303	-0.71718	2.58517
C	0.51002	1.72834	3.73200
H	0.68908	2.62455	4.33597
H	0.28439	0.90963	4.42494
H	-0.38086	1.90442	3.12003
C	5.04627	6.19079	1.02826
C	4.44199	6.60830	-0.18762
C	5.05648	7.60744	-0.95005
H	4.59273	7.91397	-1.88438
C	6.23388	8.22052	-0.54582
H	6.69637	8.98878	-1.15911
C	6.79773	7.84844	0.66721
H	7.70632	8.33982	1.00640
C	6.22351	6.85952	1.47005
C	3.11910	6.05333	-0.69049
H	2.81327	5.25776	-0.00311
C	3.21116	5.45331	-2.10028
H	3.93023	4.63185	-2.15403
H	3.50948	6.20645	-2.83730
H	2.23690	5.06046	-2.41077
C	2.03606	7.14430	-0.66801
H	1.95436	7.61369	0.31680

H	1.05757	6.72847	-0.93313
H	2.26704	7.93837	-1.38672
C	6.88864	6.54744	2.80170
H	6.29285	5.77113	3.29085
C	8.31601	6.00931	2.63143
H	8.34141	5.11743	1.99921
H	8.74681	5.74693	3.60417
H	8.97263	6.75584	2.17086
C	6.89778	7.77929	3.71874
H	5.89407	8.19814	3.83560
H	7.53598	8.57262	3.31393
H	7.28099	7.51925	4.71166

216

Int1 without dispersion

C	2.28347	-5.83490	-4.48435
C	3.41601	-6.12687	-3.71720
C	4.34251	-7.04714	-4.21718
C	4.14329	-7.66416	-5.45168
C	3.01455	-7.36026	-6.21009
C	2.08502	-6.44183	-5.72264
C	3.60936	-5.53232	-2.33582
N	3.57110	-4.06628	-2.28304
C	4.51064	-3.44119	-3.22256
C	5.92975	-3.96754	-3.14403
C	6.57799	-4.40071	-4.30460
C	7.90924	-4.81417	-4.26778
C	8.61019	-4.81036	-3.06344
C	7.97276	-4.38386	-1.89851
C	6.64580	-3.96133	-1.94263
C	2.65784	-3.35459	-1.52143
N	1.89334	-3.91012	-0.54041
C	2.02034	-5.16004	0.13313
C	3.00591	-5.37606	1.13605
C	2.97993	-6.55731	1.88537

C	2.01354	-7.53235	1.68905
C	1.05958	-7.33216	0.70154
C	1.04556	-6.17812	-0.08623
C	4.09969	-4.37426	1.47222
C	5.49802	-4.98071	1.27804
C	-0.01092	-6.09210	-1.17841
C	0.05742	-7.28882	-2.14074
N	2.40049	-2.03205	-1.64827
Y	0.67132	-1.94608	0.11931
C	0.09306	-3.66793	1.94770
O	-0.30282	-4.32258	2.79608
C	2.83404	-1.10969	-2.64139
C	3.85304	-0.16598	-2.32081
C	4.21983	0.80201	-3.25940
C	3.60841	0.88232	-4.50275
C	2.59854	-0.01868	-4.80683
C	2.19033	-1.00790	-3.90538
C	4.58925	-0.16811	-0.98946
C	6.09772	-0.40377	-1.15803
C	1.03989	-1.90561	-4.33339
C	1.34626	-2.66842	-5.63017
C	-0.25064	-1.09180	-4.52213
C	4.36226	1.14745	-0.23048
C	2.18336	-0.88356	1.91500
Al	0.34924	-0.60942	2.88156
C	-1.13846	-1.29767	1.75005
Al	-2.40091	-1.83042	0.31051
C	-1.37541	-2.52882	-1.35696
Y	-1.48806	0.97705	1.00819
C	-3.27959	-0.12592	-0.49684
C	0.29137	0.56319	-0.68364
C	0.51797	-1.32624	4.71859
C	0.23225	1.45675	2.94877
N	-1.58958	3.39529	0.41680

C	-2.66217	3.64416	1.19512
N	-3.20154	4.89206	1.47363
C	-2.67311	6.11505	0.83511
C	-3.60252	7.31114	0.86810
C	-3.34672	8.37813	1.73678
C	-4.15756	9.51229	1.73801
C	-5.23980	9.59830	0.86469
C	-5.49841	8.54810	-0.01548
C	-4.68239	7.41917	-0.01606
N	-3.15918	2.50144	1.75079
C	-4.47735	2.22647	2.20022
C	-5.61947	2.30231	1.34858
C	-6.85346	1.84509	1.82369
C	-7.00614	1.30633	3.09332
C	-5.90105	1.25514	3.93088
C	-4.64861	1.71797	3.52024
C	-5.60225	2.89933	-0.05247
C	-6.36533	4.23198	-0.07147
C	-3.51529	1.68957	4.53307
C	-3.86716	2.46180	5.81307
C	-3.85982	-3.05565	0.83549
C	-0.82175	4.26146	-0.40947
C	0.33226	4.94798	0.05563
C	1.06754	5.73420	-0.83830
C	0.71471	5.85555	-2.17451
C	-0.38717	5.15045	-2.63941
C	-1.15227	4.34518	-1.79226
C	0.84989	4.84589	1.48233
C	0.99037	6.20594	2.18111
C	-2.32649	3.59135	-2.39784
C	-3.43429	4.53978	-2.87609
C	2.20726	4.12467	1.51217
C	-1.88523	2.67780	-3.55028
C	-6.19846	1.96625	-1.11820

C	-3.09756	0.25425	4.87615
C	3.97087	-3.86309	2.91712
C	-1.42850	-5.98982	-0.59687
C	-3.93314	5.13082	2.72026
C	-3.11259	5.66027	3.88230
C	-1.85331	5.14221	4.19824
C	-1.15704	5.59136	5.31935
C	-1.71315	6.56498	6.14782
C	-2.96900	7.08797	5.84402
C	-3.65873	6.64106	4.71825
H	1.09806	0.21440	-1.34587
H	-0.46011	0.91105	-1.41640
H	0.73220	1.49221	-0.27261
H	-1.64356	-1.86855	2.54895
H	-4.01125	0.39216	0.13979
H	-2.82398	0.62104	-1.16925
H	-3.89791	-0.71916	-1.18405
H	-2.28759	-2.80562	-1.90176
H	-0.89544	-1.78598	-2.01025
H	-0.77649	-3.45007	-1.39850
H	-4.53048	-2.57672	1.56100
H	-3.49287	-3.97502	1.30882
H	-4.48259	-3.36377	-0.01502
H	2.76177	-0.49464	2.76339
H	2.43990	-0.21040	1.08528
H	2.67228	-1.85196	1.72287
H	-0.65449	1.90673	3.42318
H	1.02083	1.58045	3.70282
H	0.55829	2.12462	2.13938
H	0.92602	-2.34499	4.72822
H	-0.44141	-1.37043	5.24729
H	1.19221	-0.71442	5.33366
H	-2.44788	5.88269	-0.20377
H	-1.72788	6.40331	1.30581

H	-2.50476	8.31795	2.42093
H	-3.93975	10.32966	2.42007
H	-5.87244	10.48170	0.86226
H	-6.33084	8.61196	-0.71104
H	-4.88307	6.61488	-0.71623
H	-4.42060	4.20553	3.02474
H	-4.73719	5.84167	2.50755
H	-1.41655	4.37526	3.56598
H	-0.17964	5.17517	5.54830
H	-1.17147	6.91377	7.02266
H	-3.41122	7.85003	6.48025
H	-4.63288	7.06180	4.47944
H	1.94371	6.26374	-0.47224
H	1.29741	6.47946	-2.84643
H	-0.66200	5.22194	-3.68890
H	0.13301	4.24694	2.05203
H	0.04098	6.74383	2.24249
H	1.70571	6.85131	1.66030
H	1.35614	6.07006	3.20441
H	2.53587	3.95332	2.54332
H	2.97680	4.72239	1.01143
H	2.16762	3.15882	1.00129
H	-2.74664	2.95984	-1.60858
H	-3.81033	5.16245	-2.05984
H	-4.27838	3.97362	-3.28513
H	-3.07079	5.21043	-3.66274
H	-1.06792	2.01680	-3.24902
H	-1.53345	3.25831	-4.41036
H	-2.72244	2.05927	-3.89298
H	-7.71974	1.90790	1.17018
H	-7.97408	0.94377	3.42790
H	-6.01074	0.85669	4.93629
H	-4.56262	3.09709	-0.33039
H	-5.95252	4.94538	0.64446

H	-7.41997	4.07634	0.18198
H	-6.33017	4.68407	-1.06932
H	-6.09289	2.41630	-2.11165
H	-7.26810	1.79845	-0.95325
H	-5.70553	0.99229	-1.13350
H	-2.66126	2.19566	4.07153
H	-4.16096	3.49264	5.59700
H	-3.00244	2.49413	6.48473
H	-4.68853	1.98386	6.35809
H	-2.78665	-0.30179	3.98574
H	-3.92668	-0.29650	5.33501
H	-2.26326	0.25306	5.58559
H	2.84422	-5.92886	-1.67445
H	4.57209	-5.87685	-1.94094
H	5.23101	-7.27737	-3.63450
H	4.87402	-8.37812	-5.82204
H	2.85805	-7.83655	-7.17403
H	1.19976	-6.20204	-6.30537
H	1.54928	-5.13062	-4.10334
H	4.52938	-2.37046	-3.03915
H	4.14844	-3.57791	-4.24861
H	6.03208	-4.41889	-5.24452
H	8.39612	-5.14534	-5.18111
H	9.64608	-5.13661	-3.03191
H	8.51071	-4.37410	-0.95437
H	6.15584	-3.62146	-1.03589
H	3.73386	-6.70828	2.65363
H	2.00656	-8.43552	2.29266
H	0.30445	-8.09493	0.53016
H	4.00021	-3.52093	0.79142
H	5.62781	-5.39852	0.27694
H	5.67802	-5.78770	1.99643
H	6.27388	-4.22343	1.43999
H	4.69430	-3.06353	3.11240

H	4.16877	-4.66870	3.63241
H	2.97115	-3.47815	3.13352
H	0.19533	-5.18542	-1.75860
H	1.05491	-7.42127	-2.56677
H	-0.64531	-7.14808	-2.96956
H	-0.21725	-8.22158	-1.63633
H	-1.54630	-5.13135	0.06855
H	-1.67650	-6.88743	-0.01969
H	-2.16982	-5.89532	-1.39808
H	2.10257	0.04859	-5.77186
H	3.90828	1.64094	-5.22024
H	5.00584	1.50866	-3.00523
H	0.86816	-2.63710	-3.53590
H	2.25796	-3.26493	-5.55285
H	1.46942	-1.98125	-6.47413
H	0.52039	-3.34404	-5.88082
H	-0.47996	-0.48226	-3.64430
H	-1.10320	-1.75265	-4.71442
H	-0.15974	-0.40973	-5.37474
H	4.18011	-0.98785	-0.38957
H	6.31637	-1.32713	-1.69964
H	6.58662	-0.46189	-0.17860
H	6.56747	0.41863	-1.70894
H	3.29871	1.38234	-0.13862
H	4.83417	1.98764	-0.75176
H	4.79376	1.09623	0.77522

216

TS1 without dispersion

C	2.48275	-5.91477	-4.58076
C	3.57324	-6.16432	-3.74070
C	4.56825	-7.04226	-4.18092
C	4.47772	-7.65712	-5.42932
C	3.39075	-7.39473	-6.26059
C	2.39223	-6.52003	-5.83232

C	3.64543	-5.56576	-2.35018
N	3.60308	-4.09751	-2.31560
C	4.59328	-3.47632	-3.20200
C	6.01304	-3.97251	-3.01547
C	6.76660	-4.36012	-4.12749
C	8.09929	-4.74586	-3.98832
C	8.69508	-4.76099	-2.72858
C	7.95162	-4.38023	-1.61175
C	6.62378	-3.98373	-1.75763
C	2.68590	-3.38324	-1.56563
N	1.89319	-3.91617	-0.60474
C	1.96733	-5.17837	0.05592
C	2.91762	-5.42749	1.08145
C	2.88372	-6.64447	1.77049
C	1.93849	-7.61975	1.49228
C	1.00238	-7.37392	0.49808
C	0.99534	-6.17956	-0.22613
C	3.96229	-4.41537	1.52262
C	5.38775	-4.98423	1.45908
C	-0.06669	-6.01795	-1.30176
C	-0.04217	-7.15614	-2.33334
N	2.45113	-2.04756	-1.69341
Y	0.73882	-1.90769	-0.01251
C	-0.34432	-3.04242	1.73685
O	-0.70754	-3.87430	2.51675
C	2.88722	-1.13192	-2.69155
C	3.90099	-0.18189	-2.37342
C	4.27141	0.77695	-3.32011
C	3.67009	0.83968	-4.56956
C	2.66606	-0.06867	-4.87215
C	2.25380	-1.04793	-3.96186
C	4.62630	-0.16816	-1.03616
C	6.13621	-0.40426	-1.18866
C	1.10885	-1.95468	-4.38466

C	1.42811	-2.73629	-5.66684
C	-0.18190	-1.14601	-4.59393
C	4.38996	1.15533	-0.29419
C	2.19215	-0.84734	1.86197
Al	0.41917	-0.65916	2.89849
C	-1.11971	-1.44070	1.80541
Al	-2.42195	-1.85149	0.28919
C	-1.40095	-2.44974	-1.39070
Y	-1.45971	0.95925	1.04685
C	-3.29289	-0.10065	-0.39180
C	0.27333	0.65716	-0.68707
C	0.60478	-1.37614	4.72239
C	0.19124	1.39850	2.99490
N	-1.59945	3.33394	0.43824
C	-2.65027	3.60364	1.24332
N	-3.17424	4.85768	1.51191
C	-2.64703	6.06873	0.85091
C	-3.56874	7.27034	0.88345
C	-3.25093	8.37697	1.67837
C	-4.05265	9.51781	1.67278
C	-5.18959	9.56853	0.86927
C	-5.51292	8.47591	0.06507
C	-4.70499	7.34144	0.06878
N	-3.12935	2.47218	1.83346
C	-4.43378	2.19711	2.32523
C	-5.59720	2.23324	1.49992
C	-6.80796	1.75352	2.01137
C	-6.91808	1.23492	3.29368
C	-5.79635	1.23695	4.11040
C	-4.56636	1.72556	3.66313
C	-5.62940	2.82058	0.09514
C	-6.40318	4.14761	0.09647
C	-3.41773	1.77653	4.65764
C	-3.78397	2.57406	5.91839

C	-3.88933	-3.05560	0.80792
C	-0.84572	4.17932	-0.42448
C	0.33241	4.85009	0.00018
C	1.06751	5.59537	-0.92811
C	0.68607	5.69573	-2.25818
C	-0.44923	5.01717	-2.67990
C	-1.21527	4.25105	-1.79779
C	0.86857	4.78225	1.42239
C	1.05347	6.16291	2.06916
C	-2.43736	3.53625	-2.35522
C	-3.52268	4.52546	-2.80352
C	2.20711	4.02795	1.46559
C	-2.07938	2.59653	-3.51551
C	-6.25534	1.88269	-0.94903
C	-2.93116	0.37477	5.04309
C	3.67368	-3.92533	2.95176
C	-1.46755	-5.91434	-0.68026
C	-3.88233	5.12155	2.76776
C	-3.03950	5.68644	3.89651
C	-1.77263	5.18093	4.20276
C	-1.05497	5.66646	5.29476
C	-1.59687	6.66444	6.10353
C	-2.85964	7.17563	5.80871
C	-3.57061	6.69262	4.71145
H	1.08150	0.30352	-1.34700
H	-0.46469	1.02933	-1.42059
H	0.72387	1.57850	-0.26922
H	-1.79415	-1.68989	2.63974
H	-4.01504	0.38849	0.27631
H	-2.85861	0.67057	-1.05030
H	-3.91957	-0.67697	-1.08589
H	-2.28836	-2.71993	-1.97826
H	-0.91362	-1.67923	-2.00788
H	-0.79667	-3.36749	-1.42846

H	-4.55775	-2.57151	1.53227
H	-3.52042	-3.97244	1.28116
H	-4.51055	-3.35683	-0.04596
H	2.82474	-0.45405	2.66918
H	2.39887	-0.17004	1.02047
H	2.66398	-1.81998	1.65154
H	-0.70947	1.82125	3.46643
H	0.96007	1.50372	3.77171
H	0.52993	2.09739	2.21701
H	0.85531	-2.44301	4.70454
H	-0.31679	-1.28308	5.30879
H	1.39774	-0.86637	5.28661
H	-2.43624	5.82307	-0.18796
H	-1.69439	6.35537	1.30705
H	-2.36680	8.34368	2.30936
H	-3.78592	10.36689	2.29613
H	-5.81596	10.45630	0.86241
H	-6.39031	8.51082	-0.57505
H	-4.95797	6.50254	-0.57107
H	-4.35719	4.20071	3.10414
H	-4.69416	5.82316	2.55429
H	-1.34597	4.39546	3.58620
H	-0.07211	5.25966	5.51722
H	-1.03857	7.04133	6.95592
H	-3.29069	7.95672	6.42923
H	-4.55000	7.10463	4.47911
H	1.96529	6.10980	-0.59476
H	1.27021	6.28654	-2.95810
H	-0.75025	5.07907	-3.72269
H	0.14233	4.22494	2.02213
H	0.12360	6.73604	2.10435
H	1.79232	6.76229	1.52683
H	1.41157	6.05434	3.09833
H	2.54890	3.89790	2.49858

H	2.98292	4.58094	0.92508
H	2.13617	3.04013	1.00251
H	-2.85469	2.92903	-1.54595
H	-3.83882	5.17703	-1.98458
H	-4.40572	3.99056	-3.17036
H	-3.16246	5.16800	-3.61470
H	-1.28422	1.89840	-3.24012
H	-1.73192	3.15515	-4.39146
H	-2.95642	2.01655	-3.82407
H	-7.69003	1.78196	1.37707
H	-7.86775	0.85110	3.65553
H	-5.87544	0.86277	5.12790
H	-4.60045	3.02498	-0.21584
H	-5.97683	4.86574	0.79952
H	-7.44846	3.98297	0.38102
H	-6.40111	4.59784	-0.90286
H	-6.17061	2.32522	-1.94779
H	-7.32198	1.72339	-0.75872
H	-5.77038	0.90504	-0.96952
H	-2.59539	2.30707	4.16673
H	-4.13407	3.58066	5.67391
H	-2.90876	2.67354	6.56932
H	-4.56893	2.07501	6.49679
H	-2.61146	-0.20091	4.16884
H	-3.72721	-0.19710	5.53287
H	-2.08600	0.43236	5.73711
H	2.82069	-5.95379	-1.75899
H	4.56662	-5.91086	-1.86673
H	5.42353	-7.24159	-3.54027
H	5.26017	-8.33830	-5.75284
H	3.31914	-7.87073	-7.23469
H	1.53731	-6.31444	-6.47078
H	1.69628	-5.24534	-4.24462
H	4.57956	-2.40130	-3.04478

H	4.30316	-3.64373	-4.24623
H	6.30201	-4.36528	-5.11044
H	8.66919	-5.04205	-4.86491
H	9.73146	-5.06745	-2.61676
H	8.40678	-4.38696	-0.62502
H	6.05058	-3.67838	-0.88795
H	3.61207	-6.82328	2.55717
H	1.92678	-8.55416	2.04638
H	0.25236	-8.12822	0.27509
H	3.91083	-3.55661	0.84254
H	5.61712	-5.40741	0.47790
H	5.52960	-5.77837	2.19980
H	6.12473	-4.20296	1.67981
H	4.36282	-3.12214	3.23799
H	3.80216	-4.74280	3.66953
H	2.64896	-3.56178	3.06053
H	0.15310	-5.08499	-1.83373
H	0.94307	-7.28158	-2.79003
H	-0.76221	-6.95650	-3.13548
H	-0.32226	-8.11262	-1.87897
H	-1.51678	-5.13809	0.08605
H	-1.74433	-6.85962	-0.20022
H	-2.22076	-5.69963	-1.44707
H	2.17844	-0.01486	-5.84227
H	3.97377	1.59044	-5.29374
H	5.05319	1.48918	-3.06832
H	0.93349	-2.67523	-3.57809
H	2.34559	-3.32153	-5.57374
H	1.54942	-2.06205	-6.52156
H	0.61013	-3.42409	-5.91027
H	-0.41404	-0.51873	-3.72899
H	-1.03349	-1.81097	-4.77591
H	-0.08871	-0.48065	-5.45942
H	4.21316	-0.98220	-0.43121

H	6.35997	-1.33999	-1.70631
H	6.61753	-0.44063	-0.20448
H	6.60863	0.40688	-1.75387
H	3.32535	1.39233	-0.21992
H	4.86948	1.98996	-0.81752
H	4.80787	1.11447	0.71767

216

Int2 without dispersion

C	2.59929	-5.94442	-4.69305
C	3.64041	-6.16495	-3.78467
C	4.68544	-7.01415	-4.16064
C	4.69321	-7.62761	-5.41295
C	3.65529	-7.39343	-6.31239
C	2.60676	-6.54843	-5.94845
C	3.60573	-5.56920	-2.39194
N	3.58261	-4.09953	-2.35789
C	4.62818	-3.48807	-3.18358
C	6.03215	-3.98786	-2.90775
C	6.87198	-4.32565	-3.97337
C	8.19162	-4.71430	-3.74732
C	8.68760	-4.78278	-2.44659
C	7.85758	-4.45121	-1.37603
C	6.54311	-4.05106	-1.60792
C	2.66857	-3.37357	-1.61533
N	1.84485	-3.89288	-0.67569
C	1.88091	-5.16863	-0.03768
C	2.80096	-5.45199	1.00513
C	2.74711	-6.69086	1.65242
C	1.80850	-7.65412	1.31506
C	0.89451	-7.37029	0.31042
C	0.90812	-6.15157	-0.37166
C	3.81008	-4.44092	1.52249
C	5.23564	-5.00568	1.60766
C	-0.13967	-5.93595	-1.45083

C	-0.14171	-7.04416	-2.51450
N	2.47295	-2.02964	-1.73469
Y	0.74000	-1.84039	-0.09649
C	-0.42572	-2.87671	1.68658
O	-0.72497	-3.80002	2.42316
C	2.92432	-1.12386	-2.73395
C	3.93722	-0.17511	-2.40882
C	4.32635	0.77335	-3.35844
C	3.74421	0.82727	-4.61745
C	2.74062	-0.07930	-4.92709
C	2.31058	-1.04866	-4.01451
C	4.64189	-0.15371	-1.06075
C	6.14854	-0.42392	-1.18542
C	1.16696	-1.95328	-4.44492
C	1.49958	-2.74595	-5.71673
C	-0.11680	-1.13944	-4.67648
C	4.42026	1.18551	-0.34297
C	2.18140	-0.77811	1.79526
Al	0.43255	-0.65764	2.86778
C	-1.14535	-1.44901	1.79832
Al	-2.43974	-1.83338	0.23995
C	-1.41797	-2.34265	-1.46181
Y	-1.48050	0.97631	1.05290
C	-3.33503	-0.06669	-0.35674
C	0.23442	0.73220	-0.71292
C	0.62772	-1.39286	4.67989
C	0.16744	1.39650	2.99338
N	-1.65938	3.32357	0.43303
C	-2.68910	3.60246	1.26416
N	-3.20691	4.85902	1.52743
C	-2.69180	6.06102	0.84170
C	-3.61215	7.26333	0.87944
C	-3.27054	8.38372	1.64454
C	-4.07164	9.52505	1.64154

C	-5.23182	9.56202	0.87123
C	-5.57991	8.45476	0.09793
C	-4.77284	7.31967	0.09868
N	-3.14554	2.47814	1.88319
C	-4.43311	2.20364	2.42020
C	-5.62123	2.22288	1.62990
C	-6.81331	1.74230	2.18252
C	-6.88233	1.23996	3.47405
C	-5.73731	1.26067	4.25733
C	-4.52382	1.75051	3.76801
C	-5.69888	2.79311	0.22025
C	-6.47336	4.11981	0.22827
C	-3.35050	1.82620	4.73148
C	-3.68659	2.65783	5.97887
C	-3.88595	-3.06227	0.74911
C	-0.93011	4.14926	-0.46960
C	0.27115	4.80850	-0.09549
C	0.99574	5.51166	-1.06405
C	0.57951	5.58274	-2.38558
C	-0.58593	4.92502	-2.75497
C	-1.34461	4.20231	-1.83090
C	0.83712	4.77996	1.31688
C	1.04533	6.18080	1.91182
C	-2.60984	3.52013	-2.33073
C	-3.66068	4.54028	-2.79372
C	2.17053	4.01734	1.36337
C	-2.32760	2.52149	-3.46246
C	-6.35687	1.84226	-0.79211
C	-2.85536	0.43546	5.14536
C	3.37535	-3.93116	2.90652
C	-1.53726	-5.81029	-0.82628
C	-3.88329	5.14058	2.79707
C	-3.01109	5.72298	3.89406
C	-1.73366	5.22692	4.17084

C	-0.98694	5.73113	5.23448
C	-1.50977	6.73840	6.04432
C	-2.78292	7.23998	5.77908
C	-3.52295	6.73857	4.70965
H	1.03560	0.36522	-1.37634
H	-0.50044	1.11352	-1.44383
H	0.69920	1.64903	-0.30361
H	-1.87205	-1.57607	2.62388
H	-4.03234	0.41396	0.34309
H	-2.91782	0.71351	-1.01548
H	-3.98841	-0.62761	-1.03854
H	-2.30236	-2.59976	-2.06001
H	-0.94174	-1.54170	-2.04909
H	-0.80537	-3.25290	-1.53420
H	-4.57473	-2.58261	1.45723
H	-3.49948	-3.96116	1.24126
H	-4.48716	-3.38936	-0.10943
H	2.81968	-0.38223	2.59716
H	2.35230	-0.07713	0.96514
H	2.67410	-1.73397	1.55894
H	-0.73284	1.81257	3.47054
H	0.93453	1.48967	3.77358
H	0.50799	2.10773	2.22706
H	0.82401	-2.47012	4.64975
H	-0.27768	-1.25809	5.28316
H	1.45476	-0.92349	5.22992
H	-2.50042	5.80335	-0.19806
H	-1.73073	6.35147	1.27729
H	-2.36890	8.36025	2.25080
H	-3.78649	10.38510	2.24124
H	-5.85771	10.45013	0.86653
H	-6.47650	8.47839	-0.51555
H	-5.04648	6.46817	-0.51554
H	-4.34899	4.22444	3.15852

H	-4.69993	5.83960	2.59364
H	-1.32118	4.43398	3.55415
H	0.00371	5.33152	5.43435
H	-0.92875	7.12984	6.87467
H	-3.19947	8.02800	6.40070
H	-4.51047	7.14327	4.49996
H	1.91296	6.01543	-0.76994
H	1.15788	6.13803	-3.11853
H	-0.91741	4.96921	-3.78943
H	0.11776	4.25026	1.94936
H	0.12298	6.76641	1.93551
H	1.78445	6.75099	1.33926
H	1.41413	6.10507	2.94011
H	2.53468	3.93168	2.39340
H	2.93810	4.53863	0.78140
H	2.08274	3.00982	0.94836
H	-3.03403	2.96306	-1.48990
H	-3.91114	5.25290	-2.00346
H	-4.58336	4.03384	-3.09817
H	-3.30056	5.11721	-3.65271
H	-1.57287	1.78423	-3.17563
H	-1.96111	3.02845	-4.36164
H	-3.24325	1.98619	-3.73763
H	-7.71343	1.75674	1.57373
H	-7.81824	0.85474	3.86873
H	-5.78415	0.90069	5.28192
H	-4.68013	2.99428	-0.12421
H	-6.02593	4.84809	0.90730
H	-7.50891	3.95835	0.54808
H	-6.50347	4.55619	-0.77675
H	-6.28919	2.26454	-1.80089
H	-7.42084	1.69875	-0.57606
H	-5.88376	0.85872	-0.80396
H	-2.54078	2.34366	4.20717

H	-4.03982	3.65867	5.71623
H	-2.79689	2.77211	6.60735
H	-4.45966	2.17576	6.58693
H	-2.56696	-0.17113	4.28131
H	-3.63604	-0.11689	5.68000
H	-1.98765	0.51200	5.80905
H	2.72692	-5.94283	-1.87464
H	4.47613	-5.93155	-1.83267
H	5.50198	-7.19329	-3.46588
H	5.51331	-8.28642	-5.68552
H	3.65965	-7.86896	-7.28935
H	1.78878	-6.36574	-6.64013
H	1.77374	-5.29902	-4.40786
H	4.60997	-2.41211	-3.03255
H	4.40061	-3.65844	-4.24279
H	6.48527	-4.29062	-4.98900
H	8.82937	-4.97204	-4.58864
H	9.71346	-5.09260	-2.26742
H	8.23426	-4.49984	-0.35788
H	5.90105	-3.78501	-0.77404
H	3.45172	-6.89655	2.45393
H	1.78187	-8.60741	1.83552
H	0.14549	-8.11233	0.04674
H	3.82483	-3.59288	0.82707
H	5.55213	-5.46776	0.66885
H	5.31617	-5.76614	2.39145
H	5.94777	-4.21110	1.85994
H	4.03307	-3.12709	3.25752
H	3.42108	-4.74312	3.64053
H	2.34497	-3.56762	2.89632
H	0.10809	-4.99490	-1.95571
H	0.84232	-7.18473	-2.97015
H	-0.85213	-6.80140	-3.31343
H	-0.45087	-8.00556	-2.09044

H	-1.55854	-5.06126	-0.03184
H	-1.84207	-6.76293	-0.37870
H	-2.28438	-5.54613	-1.58379
H	2.26761	-0.03167	-5.90478
H	4.06227	1.56996	-5.34381
H	5.10793	1.48412	-3.10175
H	0.97783	-2.66733	-3.63583
H	2.41633	-3.32965	-5.60798
H	1.63056	-2.07893	-6.57570
H	0.68418	-3.43563	-5.96363
H	-0.35206	-0.49948	-3.82168
H	-0.97110	-1.80108	-4.85775
H	-0.01127	-0.48564	-5.54937
H	4.20310	-0.94976	-0.45027
H	6.35982	-1.38051	-1.66912
H	6.61637	-0.43928	-0.19420
H	6.64396	0.35959	-1.76970
H	3.35987	1.44769	-0.29516
H	4.93020	2.00197	-0.86633
H	4.81541	1.14943	0.67816

216

TS2 without dispersion

C	6.11884	6.81316	1.32081
C	5.02596	5.97400	0.95162
C	4.48736	6.10892	-0.35732
C	5.06334	7.01987	-1.25001
C	6.14443	7.81298	-0.89778
C	6.65563	7.70401	0.38747
N	4.43779	5.12007	1.93232
C	4.63521	3.80164	2.18257
N	5.52684	2.97788	1.51595
C	6.44915	3.47815	0.48363
C	7.74585	2.70381	0.36060
C	8.09474	2.11985	-0.86107

C	9.31744	1.46794	-1.01990
C	10.20858	1.38316	0.04752
C	9.87146	1.96048	1.27171
C	8.65340	2.61892	1.42263
C	3.27584	5.33719	-0.85583
C	2.10648	6.29486	-1.14162
C	6.76351	6.77968	2.69796
C	6.71398	8.15805	3.37487
Y	2.59666	5.36685	3.51591
C	4.16112	6.54252	5.22250
Al	2.47720	6.31998	6.36636
C	2.78457	5.72561	8.21484
Y	0.37712	8.09984	4.42885
C	1.93140	8.32780	6.48317
Al	-0.58253	5.38796	3.14808
C	-2.19823	4.27466	3.32796
N	0.17283	10.48624	4.01159
C	0.92581	11.42204	3.24712
C	2.08286	12.06397	3.76308
C	2.82021	12.91496	2.93236
C	2.46338	13.14251	1.61114
C	1.35460	12.48461	1.09588
C	0.58638	11.61915	1.87861
C	2.59638	11.84934	5.17864
C	3.95381	11.12738	5.16455
C	-0.59397	10.92292	1.21859
C	-0.15262	10.07362	0.01742
N	-1.34649	9.46289	5.30666
C	-2.63331	9.09636	5.79377
C	-3.79735	9.11357	4.97089
C	-4.98741	8.56743	5.46279
C	-5.07222	7.99378	6.72334
C	-3.94745	8.00226	7.53548
C	-2.73856	8.55839	7.10888

C	-3.84721	9.73314	3.58172
C	-4.46511	8.79932	2.52792
C	-1.59253	8.60562	8.10734
C	-1.05076	7.20674	8.42927
C	2.27815	7.88992	2.79694
C	0.39281	5.70944	4.93101
C	-1.17909	7.26023	2.51788
N	3.81642	3.36552	3.17940
C	3.94611	2.22010	4.02183
C	4.97475	2.11893	4.99699
C	4.96929	1.04260	5.89032
C	3.98227	0.06849	5.86201
C	2.98420	0.16010	4.90298
C	2.94936	1.20524	3.97749
C	6.09718	3.13265	5.14588
C	6.05455	3.82542	6.51679
C	1.83820	1.19282	2.94146
C	0.47012	1.45553	3.58567
C	0.72455	4.73382	1.73282
C	-0.90243	10.65592	4.81561
N	-1.47558	11.86666	5.16152
C	-2.24557	12.01360	6.39932
C	-1.48473	12.56626	7.58986
C	-0.17812	12.17105	7.88897
C	0.46274	12.63660	9.03649
C	-0.19677	13.50279	9.90695
C	-1.50068	13.90323	9.61886
C	-2.13497	13.44188	8.46711
C	-0.99095	13.14240	4.59453
C	-1.97458	14.29127	4.67984
C	-1.73171	15.35907	5.55032
C	-2.59412	16.45359	5.59982
C	-3.71730	16.49568	4.77676
C	-3.96697	15.44137	3.89859

C	-3.09924	14.35302	3.84827
C	5.37318	1.51894	1.49315
C	4.85341	0.94437	0.18920
C	5.48631	-0.16484	-0.38192
C	4.97558	-0.76790	-1.53056
C	3.82475	-0.26244	-2.13231
C	3.18905	0.84643	-1.57525
C	3.69908	1.44229	-0.42356
C	2.72863	13.15433	5.97697
C	-1.68235	11.91439	0.78361
C	-4.63870	11.04903	3.60794
C	-1.99205	9.32385	9.40556
C	7.47239	2.48096	4.94047
C	1.79698	-0.11854	2.14256
C	3.57304	4.51349	-2.11675
C	8.21858	6.29032	2.64556
H	3.20277	7.54689	2.30103
H	1.66559	8.21479	1.94083
H	2.62854	8.83320	3.25839
H	-0.58278	5.67203	5.47103
H	-1.87915	7.85395	3.12420
H	-0.51810	7.95109	1.97465
H	-1.82309	6.85928	1.72352
H	-0.01746	4.51915	0.95145
H	1.40939	5.43419	1.23486
H	1.23856	3.77105	1.86724
H	-2.96538	4.79303	3.91832
H	-1.99807	3.32518	3.83604
H	-2.65251	4.03397	2.35674
H	4.74202	6.90171	6.08393
H	4.24699	7.34291	4.48113
H	4.74654	5.67726	4.87734
H	0.96273	8.66199	6.88634
H	2.61385	8.49709	7.32684

H	2.28125	9.07153	5.74968
H	3.12605	4.68562	8.23871
H	1.85467	5.75758	8.79451
H	3.52323	6.34363	8.74351
H	-0.74332	12.97571	3.54828
H	-0.06486	13.44435	5.09473
H	-0.85910	15.33163	6.19723
H	-2.38589	17.27312	6.28215
H	-4.39073	17.34756	4.81295
H	-4.83397	15.47043	3.24421
H	-3.29735	13.54286	3.15406
H	-2.66924	11.04634	6.66548
H	-3.09468	12.67189	6.19264
H	0.34151	11.49050	7.22095
H	1.47887	12.31786	9.25252
H	0.30207	13.86358	10.80206
H	-2.02368	14.58144	10.28774
H	-3.14778	13.76784	8.24157
H	3.70039	13.40956	3.33535
H	3.04834	13.81303	0.98797
H	1.07722	12.64125	0.05641
H	1.87626	11.20849	5.69795
H	1.77684	13.68361	6.06952
H	3.44596	13.83728	5.50986
H	3.08871	12.94521	6.98992
H	4.28155	10.89503	6.18398
H	4.72411	11.75386	4.70198
H	3.91590	10.19306	4.59693
H	-1.03248	10.24985	1.96136
H	-2.04236	12.51488	1.62322
H	-2.53955	11.38242	0.35599
H	-1.30874	12.60737	0.02153
H	0.64369	9.37425	0.28830
H	0.22928	10.69897	-0.79703

H	-0.99694	9.49775	-0.37749
H	-5.87061	8.58384	4.82991
H	-6.00463	7.55834	7.07134
H	-4.00680	7.57805	8.53435
H	-2.82248	9.96106	3.27038
H	-4.21680	11.76022	4.32037
H	-5.68011	10.86631	3.89569
H	-4.64643	11.51586	2.61615
H	-4.33280	9.22133	1.52557
H	-5.54204	8.67730	2.68532
H	-4.01662	7.80357	2.54127
H	-0.79213	9.19525	7.64822
H	-2.38902	10.32386	9.21142
H	-1.12029	9.43065	10.05983
H	-2.75089	8.76110	9.95986
H	-0.70681	6.67734	7.53598
H	-1.82434	6.58441	8.89229
H	-0.21085	7.26749	9.12943
H	4.71015	1.22213	2.30289
H	6.34678	1.06890	1.71135
H	6.39011	-0.55685	0.07840
H	5.48104	-1.63014	-1.95720
H	3.42505	-0.72912	-3.02837
H	2.28891	1.24503	-2.03521
H	3.18935	2.29782	0.01101
H	6.68999	4.51411	0.70865
H	5.95332	3.47481	-0.49417
H	7.39950	2.17342	-1.69492
H	9.56990	1.02268	-1.97845
H	11.16033	0.87306	-0.07290
H	10.56090	1.90414	2.10979
H	8.40125	3.07011	2.37641
H	5.75623	0.97839	6.63762
H	3.99128	-0.74946	6.57693

H	2.20749	-0.59895	4.86663
H	5.97140	3.89720	4.37166
H	7.51883	1.92750	3.99942
H	7.69744	1.77429	5.74666
H	8.26550	3.23798	4.94075
H	6.80405	4.62371	6.57180
H	6.27275	3.11371	7.32030
H	5.07263	4.25644	6.72473
H	2.05516	2.00073	2.23416
H	2.75803	-0.34595	1.67394
H	1.04442	-0.05398	1.34888
H	1.52465	-0.96602	2.78072
H	0.47124	2.35601	4.20467
H	0.19084	0.62495	4.24399
H	-0.30870	1.54832	2.82043
H	4.64482	7.11012	-2.24908
H	6.57679	8.50998	-1.61019
H	7.49762	8.32655	0.67939
H	2.96866	4.64668	-0.06412
H	4.36738	3.78095	-1.95497
H	3.87419	5.15620	-2.95075
H	2.67831	3.96652	-2.43346
H	1.88743	6.93739	-0.28379
H	1.19843	5.73717	-1.39646
H	2.34068	6.95225	-1.98592
H	6.19190	6.07706	3.30979
H	8.30783	5.30889	2.17372
H	8.63570	6.21970	3.65657
H	8.84898	6.98286	2.07669
H	5.70654	8.58246	3.35999
H	7.37457	8.87128	2.86939
H	7.04215	8.08786	4.41759
C	1.29331	4.71719	5.54012
O	0.96050	3.80124	6.30488

216

Int3 without dispersion

C	4.05858	0.05464	-2.68334
C	3.04514	-0.93681	-2.77825
C	2.27810	-1.03584	-3.96898
C	2.54006	-0.15503	-5.02144
C	3.52809	0.81855	-4.92962
C	4.27627	0.91414	-3.76429
N	2.74068	-1.70545	-1.62427
C	2.89924	-3.03144	-1.37348
N	3.61121	-3.90497	-2.16823
C	4.46261	-3.46577	-3.28379
C	5.86467	-4.03955	-3.29219
C	6.29950	-4.82337	-4.36531
C	7.61455	-5.28313	-4.42737
C	8.51431	-4.96616	-3.41188
C	8.09297	-4.18181	-2.33827
C	6.77976	-3.72014	-2.28374
C	1.14133	-2.03555	-4.10362
C	-0.19758	-1.37372	-3.75326
C	4.92752	0.20766	-1.44423
C	4.64761	1.53208	-0.71841
Y	1.29647	-1.25171	0.20898
C	2.65662	-0.45476	2.14215
Al	1.04161	-1.23250	3.30537
C	1.67036	-2.70470	4.44403
Y	-1.70652	0.74888	0.18247
C	0.23270	0.36153	4.14587
Al	-3.54915	-1.59073	-0.58071
C	-5.41644	-1.56988	0.05916
N	-1.61102	3.14527	0.22100
C	-0.65414	4.04250	-0.32711
C	0.44482	4.53665	0.42537
C	1.45079	5.25588	-0.22716

C	1.40123	5.51559	-1.59044
C	0.31077	5.06146	-2.31924
C	-0.71964	4.33277	-1.71886
C	0.58351	4.31323	1.92338
C	1.64129	3.24778	2.23599
C	-1.90242	3.92438	-2.58509
C	-1.49353	3.04235	-3.77362
N	-3.22262	2.14254	1.40100
C	-4.54274	1.89827	1.88200
C	-5.69981	2.18600	1.10254
C	-6.96319	1.85644	1.60396
C	-7.13033	1.24021	2.83503
C	-6.00288	0.93948	3.58420
C	-4.71500	1.25028	3.13853
C	-5.66478	2.81696	-0.28234
C	-6.20350	1.85265	-1.35204
C	-3.55921	0.89053	4.05828
C	-3.50330	-0.62135	4.31885
C	0.62158	0.95988	-0.90487
C	-2.23965	-1.58880	1.02885
C	-3.09766	0.11397	-1.77724
N	2.24101	-3.37538	-0.23740
C	2.39444	-4.52968	0.58176
C	3.57910	-4.77752	1.32893
C	3.63674	-5.90378	2.15686
C	2.56912	-6.78115	2.28125
C	1.40350	-6.51897	1.57547
C	1.28593	-5.40679	0.73784
C	4.79932	-3.86647	1.31628
C	5.00949	-3.19895	2.68575
C	-0.03758	-5.20322	0.01801
C	-1.20021	-5.09404	1.01407
C	-3.08391	-3.12190	-1.76238
C	-2.66464	3.33436	1.05215

N	-3.11748	4.56277	1.49665
C	-3.97163	4.71344	2.68148
C	-3.30229	5.33870	3.89108
C	-2.15520	4.78000	4.46598
C	-1.59911	5.32393	5.62229
C	-2.18547	6.43525	6.22732
C	-3.33323	6.99469	5.66987
C	-3.88371	6.44981	4.51040
C	-2.53641	5.83084	1.02659
C	-3.51240	6.97891	0.86454
C	-3.37052	8.13510	1.63878
C	-4.20459	9.23539	1.44191
C	-5.19786	9.19417	0.46568
C	-5.34391	8.05029	-0.31861
C	-4.50268	6.95738	-0.12314
C	3.53567	-5.36389	-2.01801
C	3.17681	-6.12633	-3.27799
C	3.95157	-7.22528	-3.66264
C	3.59878	-7.99847	-4.76775
C	2.46597	-7.67666	-5.51266
C	1.68589	-6.58280	-5.13921
C	2.03671	-5.81871	-4.02796
C	0.92107	5.60239	2.68730
C	-2.66468	5.15580	-3.10038
C	-6.47579	4.11934	-0.32943
C	-3.63808	1.64268	5.39557
C	6.08068	-4.61974	0.93074
C	-0.31379	-6.31671	-1.00319
C	1.05546	-2.68702	-5.48893
C	6.42479	0.09343	-1.76658
H	1.43531	0.61344	-1.56528
H	-0.02960	1.43550	-1.65970
H	1.05422	1.82468	-0.37836
H	-2.64457	-2.35055	1.71036

H	-3.43240	1.15435	-1.62375
H	-2.15887	0.14635	-2.35515
H	-3.82314	-0.26259	-2.50944
H	-3.52399	-2.99870	-2.76223
H	-2.00578	-3.26967	-1.89732
H	-3.48814	-4.06150	-1.36410
H	-5.66665	-0.72473	0.70957
H	-5.60226	-2.48362	0.64196
H	-6.14520	-1.57991	-0.76256
H	3.15322	-0.19041	3.08536
H	2.61918	0.51588	1.61767
H	3.42002	-1.07301	1.63732
H	-0.54333	0.08848	4.87009
H	0.99293	0.92945	4.69966
H	-0.22506	1.06380	3.43709
H	2.00120	-3.58020	3.87390
H	0.85114	-3.04176	5.09333
H	2.49481	-2.41180	5.10752
H	-2.05949	5.65280	0.06614
H	-1.74958	6.14748	1.72034
H	-2.60003	8.17280	2.40418
H	-4.07648	10.12479	2.05297
H	-5.84947	10.04978	0.31130
H	-6.10770	8.01195	-1.09057
H	-4.61104	6.07673	-0.74665
H	-4.36085	3.73630	2.95810
H	-4.83602	5.32629	2.40355
H	-1.70529	3.89880	4.01746
H	-0.71395	4.86957	6.05919
H	-1.75361	6.85704	7.13067
H	-3.80178	7.85746	6.13575
H	-4.77551	6.89424	4.07563
H	2.29562	5.62123	0.35061
H	2.19695	6.07418	-2.07528

H	0.25278	5.27825	-3.38298
H	-0.37787	3.94458	2.29372
H	0.23715	6.42029	2.44413
H	1.93709	5.94559	2.46485
H	0.86510	5.42925	3.76618
H	1.73697	3.09509	3.31592
H	2.62076	3.54597	1.84511
H	1.37882	2.28694	1.78832
H	-2.58618	3.34880	-1.95208
H	-2.96117	5.82446	-2.28780
H	-3.56881	4.85234	-3.64046
H	-2.04750	5.73966	-3.79237
H	-0.94889	2.14876	-3.45642
H	-0.84470	3.58788	-4.46759
H	-2.37836	2.71934	-4.33330
H	-7.83950	2.07651	1.00000
H	-8.12277	0.99076	3.19979
H	-6.11933	0.45194	4.54839
H	-4.62415	3.05422	-0.52946
H	-6.14478	4.83609	0.42496
H	-7.53941	3.92432	-0.15618
H	-6.39201	4.59157	-1.31515
H	-6.06276	2.27166	-2.35548
H	-7.27673	1.68160	-1.21451
H	-5.71385	0.87887	-1.30625
H	-2.63199	1.18722	3.55839
H	-3.68966	2.72558	5.25820
H	-2.75625	1.42322	6.00783
H	-4.52081	1.33801	5.96924
H	-3.44471	-1.18344	3.38505
H	-4.39237	-0.96210	4.86120
H	-2.63011	-0.87694	4.92782
H	2.79802	-5.59454	-1.25293
H	4.49776	-5.73648	-1.64789

H	4.84316	-7.47383	-3.09219
H	4.21321	-8.84960	-5.04860
H	2.19033	-8.27498	-6.37660
H	0.79677	-6.32801	-5.70935
H	1.41570	-4.97709	-3.73608
H	4.53761	-2.38244	-3.25427
H	3.97249	-3.72404	-4.22988
H	5.60009	-5.07779	-5.15729
H	7.93434	-5.89021	-5.26995
H	9.53936	-5.32346	-3.45776
H	8.78980	-3.92375	-1.54545
H	6.45780	-3.10152	-1.45145
H	4.54257	-6.08986	2.72790
H	2.64107	-7.65038	2.92899
H	0.55602	-7.19168	1.67575
H	4.63181	-3.07610	0.57520
H	5.99584	-5.11485	-0.03881
H	6.32535	-5.38444	1.67525
H	6.93185	-3.93108	0.88540
H	5.82452	-2.46698	2.63748
H	5.28022	-3.94478	3.44116
H	4.10867	-2.69506	3.03984
H	0.02518	-4.25783	-0.53216
H	0.48564	-6.40647	-1.74467
H	-1.24955	-6.11776	-1.53690
H	-0.41442	-7.28936	-0.50837
H	-1.01171	-4.30778	1.74876
H	-1.35206	-6.03708	1.55119
H	-2.12981	-4.86201	0.48572
H	1.95266	-0.22791	-5.93220
H	3.71173	1.49501	-5.75965
H	5.05100	1.67294	-3.68908
H	1.31294	-2.83216	-3.37221
H	2.00281	-3.14674	-5.78532

H	0.77527	-1.96400	-6.26222
H	0.28496	-3.46515	-5.49001
H	-0.17912	-0.95872	-2.74034
H	-1.02241	-2.09072	-3.81020
H	-0.41002	-0.54798	-4.44182
H	4.66763	-0.61225	-0.76535
H	6.65992	-0.83713	-2.29077
H	7.01608	0.12412	-0.84421
H	6.76376	0.92220	-2.39776
H	3.58881	1.64488	-0.46832
H	4.92157	2.38785	-1.34546
H	5.22953	1.59651	0.20794
C	-0.92275	-1.30190	1.15313
O	-0.02805	-1.97092	1.97099

216

TS3 without dispersion

C	0.99756	-5.48709	0.54415
C	2.12898	-4.62332	0.52863
C	3.22082	-4.90858	1.39473
C	3.18005	-6.05837	2.18957
C	2.09678	-6.92482	2.17213
C	1.01470	-6.62391	1.35769
N	2.08134	-3.44131	-0.26595
C	2.79943	-3.09380	-1.36763
N	3.58319	-3.96064	-2.10037
C	3.51971	-5.42065	-1.95918
C	3.25511	-6.17534	-3.24652
C	2.16913	-5.86022	-4.06929
C	1.89230	-6.61678	-5.20615
C	2.69547	-7.70844	-5.53398
C	3.77816	-8.03422	-4.71920
C	4.05673	-7.26929	-3.58735
C	4.43304	-4.00332	1.55264
C	5.75510	-4.74436	1.30751

C	-0.24437	-5.24730	-0.29958
C	-0.41141	-6.31856	-1.38743
Y	1.12226	-1.32520	0.12880
C	-1.17576	-1.62308	0.62560
C	-2.49029	-1.65027	0.74128
Al	-3.55200	-1.71862	-1.00309
C	-3.12188	0.25935	-1.69873
Y	-1.62345	0.88107	0.29726
C	0.53538	0.83034	-1.09480
N	-3.10187	2.22400	1.53762
C	-4.35068	1.87728	2.12572
C	-5.58874	2.06038	1.44568
C	-6.76444	1.58150	2.03192
C	-6.76472	0.92194	3.25252
C	-5.55827	0.74143	3.91268
C	-4.35053	1.20338	3.38108
C	-5.72924	2.75108	0.09620
C	-6.62967	3.99186	0.19487
C	-3.09579	1.00641	4.22046
C	-3.16166	1.83316	5.51479
C	-2.61784	3.43165	1.13924
N	-1.62107	3.26433	0.23394
C	-0.74158	4.20392	-0.37286
C	0.40563	4.69831	0.29975
C	1.29344	5.53133	-0.38776
C	1.08857	5.88192	-1.71562
C	-0.02543	5.38421	-2.37755
C	-0.94577	4.54920	-1.73746
C	0.74855	4.30813	1.72794
C	1.09506	5.50753	2.61930
C	-2.14090	4.05397	-2.53729
C	-3.00604	5.21221	-3.05627
N	-3.07437	4.64197	1.61499
C	-2.59742	5.93496	1.09654

C	-3.65917	7.00915	0.98279
C	-3.55089	8.18825	1.72659
C	-4.47663	9.21953	1.56965
C	-5.52880	9.08328	0.66662
C	-5.64562	7.91275	-0.08282
C	-4.71415	6.88884	0.07200
C	-3.88349	4.75842	2.83332
C	-3.20928	5.47261	3.98904
C	-3.89704	6.47898	4.67516
C	-3.33566	7.09535	5.79237
C	-2.06958	6.71789	6.23583
C	-1.37374	5.71725	5.55844
C	-1.94172	5.09832	4.44652
C	-6.29814	1.80548	-0.97371
C	-2.85317	-0.46837	4.57047
C	1.90913	3.30385	1.73265
C	-1.70530	3.16213	-3.70964
N	2.63556	-1.77437	-1.64561
C	3.02620	-0.98298	-2.75895
C	4.05225	-0.01281	-2.57645
C	4.34297	0.88417	-3.60726
C	3.65164	0.85779	-4.81096
C	2.65551	-0.09177	-4.99005
C	2.32460	-1.01680	-3.99379
C	4.87657	0.07562	-1.30030
C	6.36805	-0.18215	-1.56571
C	1.20547	-2.00335	-4.29655
C	1.47478	-2.79793	-5.58376
O	-0.43789	-0.41357	1.67673
Al	0.72796	-0.64236	3.10261
C	0.70513	0.89860	4.34393
C	0.55262	-2.47955	3.80988
C	2.56905	-0.51556	2.05630
C	4.70992	1.43149	-0.59922

C	-0.15406	-1.29918	-4.42067
C	4.51817	-3.51230	-3.14105
C	5.91399	-4.09265	-3.03433
C	6.45966	-4.80278	-4.10813
C	7.77040	-5.27612	-4.05955
C	8.55284	-5.05191	-2.92859
C	8.01780	-4.34791	-1.85001
C	6.71048	-3.86959	-1.90659
C	4.46092	-3.37739	2.95779
C	-1.50737	-5.18492	0.57123
C	-5.48589	-1.88134	-0.60386
C	-2.83079	-2.92585	-2.40042
H	1.58711	1.05831	-0.83486
H	0.59868	0.42314	-2.11409
H	0.15586	1.85506	-1.26100
H	-3.01512	-1.19623	1.59297
H	-3.51448	1.22744	-1.34173
H	-2.14738	0.44025	-2.18867
H	-3.77883	0.06147	-2.55683
H	-3.16669	-2.64034	-3.40651
H	-1.73541	-2.96795	-2.42573
H	-3.18562	-3.95185	-2.23572
H	-5.82775	-1.23172	0.21147
H	-5.70793	-2.91302	-0.29663
H	-6.12457	-1.67431	-1.47322
H	3.08825	-0.38500	3.01507
H	2.77833	0.42248	1.51883
H	3.17507	-1.30668	1.58239
H	0.09575	0.69290	5.23239
H	1.71441	1.13553	4.70736
H	0.30821	1.81544	3.89155
H	0.71471	-3.27188	3.06652
H	-0.44757	-2.64910	4.22889
H	1.26976	-2.66645	4.62118

H	-2.16923	5.77245	0.11099
H	-1.79032	6.30899	1.73718
H	-2.73572	8.29718	2.43715
H	-4.37430	10.12922	2.15509
H	-6.25135	9.88528	0.54331
H	-6.45849	7.80008	-0.79506
H	-4.80384	5.98431	-0.52073
H	-4.17735	3.76089	3.15425
H	-4.80783	5.29164	2.58599
H	-1.39986	4.30825	3.93469
H	-0.38943	5.41068	5.90115
H	-1.62809	7.19821	7.10460
H	-3.88680	7.87396	6.31283
H	-4.88038	6.78488	4.32639
H	2.17265	5.90572	0.12965
H	1.79264	6.53017	-2.22971
H	-0.18947	5.64827	-3.41916
H	-0.12577	3.80776	2.15810
H	0.29188	6.24879	2.64494
H	2.00529	6.01216	2.27895
H	1.27682	5.17405	3.64622
H	2.14441	2.97732	2.75020
H	2.80977	3.75260	1.29947
H	1.66182	2.42004	1.13865
H	-2.75725	3.44992	-1.86339
H	-3.31189	5.88787	-2.25273
H	-3.90970	4.82725	-3.54216
H	-2.46623	5.81142	-3.79785
H	-1.08153	2.32826	-3.37612
H	-1.12324	3.73262	-4.44215
H	-2.57899	2.75019	-4.22651
H	-7.70570	1.72117	1.50702
H	-7.69219	0.55380	3.68188
H	-5.54878	0.23457	4.87383

H	-4.73613	3.07649	-0.23325
H	-6.26549	4.71086	0.93192
H	-7.64899	3.71156	0.48091
H	-6.69438	4.49843	-0.77477
H	-6.28293	2.28841	-1.95773
H	-7.33914	1.54611	-0.75273
H	-5.73871	0.87110	-1.03596
H	-2.24023	1.36237	3.63678
H	-3.32217	2.89612	5.31844
H	-2.22723	1.73424	6.07756
H	-3.97683	1.48696	6.16017
H	-2.74584	-1.09349	3.68046
H	-3.67498	-0.87934	5.16667
H	-1.93853	-0.57224	5.16213
H	2.73620	-5.66811	-1.24685
H	4.46136	-5.78107	-1.52934
H	4.90904	-7.52077	-2.96075
H	4.41142	-8.88210	-4.96610
H	2.47786	-8.30124	-6.41809
H	1.04377	-6.35685	-5.83300
H	1.53445	-5.01654	-3.81473
H	4.58728	-2.42798	-3.10218
H	4.11183	-3.76889	-4.12583
H	5.84921	-4.98999	-4.98784
H	8.17742	-5.82409	-4.90502
H	9.57368	-5.42157	-2.88720
H	8.62103	-4.16610	-0.96468
H	6.30084	-3.31568	-1.06740
H	4.01762	-6.27182	2.84845
H	2.09005	-7.81432	2.79596
H	0.15342	-7.28649	1.35095
H	4.35403	-3.19585	0.81504
H	5.78568	-5.22317	0.32631
H	5.91730	-5.52167	2.06157

H	6.60108	-4.05052	1.37392
H	5.25908	-2.62984	3.03578
H	4.65120	-4.14558	3.71545
H	3.51129	-2.90117	3.21054
H	-0.12929	-4.27798	-0.79536
H	0.46448	-6.38900	-2.03808
H	-1.28090	-6.09110	-2.01357
H	-0.57140	-7.30756	-0.94294
H	-1.40480	-4.45356	1.37586
H	-1.72757	-6.15919	1.02229
H	-2.37096	-4.89634	-0.03494
H	2.10957	-0.11407	-5.92964
H	3.88682	1.56687	-5.59974
H	5.12986	1.61978	-3.46078
H	1.14222	-2.71157	-3.46246
H	2.43618	-3.31714	-5.55846
H	1.47519	-2.14229	-6.46090
H	0.68980	-3.54541	-5.74113
H	-0.44017	-0.79670	-3.49374
H	-0.94117	-2.02057	-4.65968
H	-0.13072	-0.54615	-5.21611
H	4.51377	-0.70502	-0.62248
H	6.53835	-1.13552	-2.07238
H	6.92802	-0.19326	-0.62343
H	6.79945	0.60528	-2.19360
H	3.65996	1.66916	-0.40790
H	5.11741	2.24451	-1.21038
H	5.24222	1.43859	0.35851

216

Int4 without dispersion

C	0.83340	-5.56030	0.47344
C	1.96534	-4.69601	0.51228
C	3.03823	-5.01708	1.38979
C	2.97489	-6.19310	2.14441

C	1.89358	-7.05823	2.07141
C	0.83172	-6.72785	1.24180
N	1.92919	-3.49244	-0.24953
C	2.67353	-3.12929	-1.32678
N	3.49007	-3.98273	-2.04680
C	3.47269	-5.44108	-1.87654
C	3.43885	-6.24897	-3.15746
C	2.48781	-6.01318	-4.15423
C	2.42216	-6.82414	-5.28567
C	3.30256	-7.89558	-5.43107
C	4.25030	-8.14506	-4.43992
C	4.31977	-7.32353	-3.31628
C	4.25659	-4.13073	1.60538
C	5.58090	-4.88551	1.41860
C	-0.38237	-5.29000	-0.40018
C	-0.40134	-6.18935	-1.64496
Y	0.99785	-1.32061	0.17524
C	-1.57663	-1.80275	0.25406
C	-2.49610	-2.22672	0.96934
Al	-3.44313	-1.68144	-1.15898
C	-3.12213	0.24638	-1.70737
Y	-1.49408	0.86490	0.43166
C	0.43100	0.69443	-1.25928
N	-3.09308	2.19988	1.57463
C	-4.32095	1.85991	2.19903
C	-5.57817	2.00982	1.54779
C	-6.72924	1.51616	2.17014
C	-6.68581	0.87314	3.39944
C	-5.46054	0.73188	4.03610
C	-4.27875	1.21635	3.46878
C	-5.75673	2.68306	0.19460
C	-6.66634	3.91658	0.30075
C	-2.99137	1.05709	4.26307
C	-3.08075	1.73711	5.63770

C	-2.59963	3.40031	1.17411
N	-1.59488	3.22666	0.28060
C	-0.69437	4.15097	-0.31784
C	0.44787	4.63493	0.37086
C	1.36484	5.43840	-0.31370
C	1.18921	5.77472	-1.64964
C	0.07449	5.29456	-2.32350
C	-0.87153	4.48450	-1.68903
C	0.74310	4.27756	1.81823
C	1.09891	5.49892	2.67637
C	-2.06796	4.00774	-2.49829
C	-2.93463	5.18194	-2.97862
N	-3.05541	4.61792	1.63827
C	-2.56566	5.90269	1.11707
C	-3.62713	6.97296	0.96879
C	-3.49916	8.18817	1.64832
C	-4.42496	9.21403	1.45881
C	-5.49790	9.03526	0.58823
C	-5.63559	7.82724	-0.09548
C	-4.70403	6.80866	0.09108
C	-3.85612	4.75162	2.86079
C	-3.16012	5.44554	4.01649
C	-3.78634	6.51773	4.66028
C	-3.20609	7.12087	5.77540
C	-1.98329	6.66148	6.26035
C	-1.34873	5.59404	5.62581
C	-1.93440	4.99094	4.51454
C	-6.33853	1.72052	-0.85293
C	-2.61136	-0.41919	4.43373
C	1.86958	3.23842	1.89662
C	-1.64062	3.15255	-3.70039
N	2.51457	-1.80811	-1.60430
C	2.87749	-1.05248	-2.75469
C	3.86432	-0.03403	-2.61873

C	4.12373	0.82467	-3.68981
C	3.44328	0.71410	-4.89389
C	2.49314	-0.28718	-5.03165
C	2.19421	-1.17788	-3.99441
C	4.69361	0.14679	-1.35597
C	6.18636	-0.11054	-1.61688
C	1.12133	-2.22277	-4.27064
C	1.40782	-3.00962	-5.55992
O	-0.00438	0.13433	1.66162
Al	1.03794	-0.50956	2.98861
C	1.24400	0.58470	4.62058
C	0.47795	-2.42587	3.21610
C	2.83030	-0.56986	1.88657
C	4.51738	1.54402	-0.74321
C	-0.27201	-1.58600	-4.38039
C	4.48452	-3.50296	-3.01663
C	5.88584	-4.04932	-2.83241
C	6.51157	-4.73607	-3.87760
C	7.83141	-5.16916	-3.75969
C	8.54385	-4.92698	-2.58685
C	7.92995	-4.24433	-1.53717
C	6.61336	-3.80588	-1.66292
C	4.23129	-3.51339	3.01308
C	-1.69573	-5.45889	0.37577
C	-5.19648	-1.92537	-0.25543
C	-3.03428	-3.01868	-2.54966
H	1.52406	0.82809	-1.15520
H	0.30658	0.37521	-2.30068
H	0.11220	1.75531	-1.26472
H	-3.23881	-2.63081	1.62730
H	-3.51481	1.09175	-1.11996
H	-2.13033	0.50294	-2.10904
H	-3.76297	0.27044	-2.60112
H	-3.36282	-2.68124	-3.54169

H	-1.97184	-3.26879	-2.63154
H	-3.57370	-3.95151	-2.34028
H	-5.39445	-1.25045	0.58588
H	-5.32954	-2.95429	0.10446
H	-5.99745	-1.75614	-0.98933
H	3.45632	-0.54848	2.78904
H	3.06060	0.37077	1.36693
H	3.29057	-1.38725	1.30821
H	1.22803	-0.03430	5.52703
H	2.18529	1.14817	4.63889
H	0.43302	1.31721	4.72056
H	0.50668	-3.11896	2.35991
H	-0.55350	-2.46630	3.58948
H	1.10893	-2.90560	3.97685
H	-2.11481	5.73037	0.14289
H	-1.77300	6.28453	1.77138
H	-2.66839	8.32942	2.33487
H	-4.30708	10.15223	1.99425
H	-6.22106	9.83247	0.43993
H	-6.46587	7.68063	-0.78103
H	-4.81081	5.87345	-0.44910
H	-4.17621	3.76235	3.18088
H	-4.76583	5.31119	2.61710
H	-1.43919	4.15198	4.03395
H	-0.39885	5.22401	6.00163
H	-1.52768	7.12984	7.12839
H	-3.70924	7.95183	6.26251
H	-4.73703	6.88446	4.28114
H	2.24223	5.80298	0.21361
H	1.91588	6.40044	-2.16040
H	-0.06801	5.55093	-3.37025
H	-0.15803	3.82043	2.23979
H	0.32534	6.27103	2.63918
H	2.04161	5.95475	2.35585

H	1.22486	5.20177	3.72237
H	2.07215	2.96469	2.93696
H	2.79338	3.63632	1.46215
H	1.60255	2.32594	1.35849
H	-2.67899	3.38291	-1.83862
H	-3.24716	5.82824	-2.15377
H	-3.83525	4.81347	-3.48311
H	-2.39064	5.80797	-3.69468
H	-1.00682	2.31462	-3.39826
H	-1.07381	3.74663	-4.42606
H	-2.51943	2.75114	-4.21737
H	-7.68647	1.63119	1.66821
H	-7.59497	0.49184	3.85587
H	-5.41608	0.24109	5.00499
H	-4.77326	3.01283	-0.15834
H	-6.29119	4.64488	1.02311
H	-7.67659	3.63065	0.61267
H	-6.75636	4.41415	-0.67158
H	-6.35754	2.19541	-1.84066
H	-7.36844	1.44416	-0.60216
H	-5.76206	0.79681	-0.92433
H	-2.19074	1.54538	3.69692
H	-3.34857	2.79369	5.55345
H	-2.11726	1.67329	6.15503
H	-3.82823	1.25240	6.27559
H	-2.47731	-0.91346	3.46739
H	-3.38495	-0.96558	4.98551
H	-1.67394	-0.51441	4.99080
H	2.59937	-5.70717	-1.28599
H	4.34722	-5.75295	-1.29440
H	5.07123	-7.51362	-2.55397
H	4.94248	-8.97631	-4.54371
H	3.24965	-8.53120	-6.31068
H	1.67840	-6.62138	-6.05155

H	1.79643	-5.18458	-4.04161
H	4.52854	-2.41882	-2.95503
H	4.14726	-3.74511	-4.03071
H	5.95620	-4.93704	-4.79013
H	8.30049	-5.69975	-4.58382
H	9.57205	-5.26496	-2.49118
H	8.47918	-4.04694	-0.62058
H	6.14422	-3.26409	-0.84691
H	3.79675	-6.43105	2.81438
H	1.87281	-7.96988	2.66210
H	-0.02578	-7.39274	1.18735
H	4.22267	-3.31683	0.87212
H	5.65771	-5.36023	0.43791
H	5.70409	-5.66575	2.17693
H	6.42743	-4.19814	1.52700
H	5.06568	-2.81515	3.14601
H	4.32540	-4.29510	3.77505
H	3.29923	-2.97878	3.20206
H	-0.32352	-4.25002	-0.73825
H	0.49476	-6.06541	-2.25787
H	-1.27196	-5.96167	-2.26978
H	-0.45903	-7.24592	-1.35914
H	-1.67421	-4.91016	1.32133
H	-1.89845	-6.51093	0.60499
H	-2.53521	-5.09074	-0.22229
H	1.95705	-0.37878	-5.97253
H	3.65254	1.39501	-5.71417
H	4.87930	1.59747	-3.57415
H	1.10422	-2.92901	-3.43311
H	2.41199	-3.44170	-5.56980
H	1.31522	-2.37003	-6.44386
H	0.68278	-3.82257	-5.67886
H	-0.58702	-1.12974	-3.43937
H	-1.01957	-2.33765	-4.65134

H	-0.28131	-0.80827	-5.15211
H	4.34737	-0.59202	-0.62669
H	6.36725	-1.09844	-2.04869
H	6.75446	-0.04165	-0.68188
H	6.60048	0.63151	-2.30857
H	3.46732	1.78245	-0.55253
H	4.90865	2.32063	-1.40988
H	5.06240	1.61795	0.20435

216

Int5 without dispersion

C	-2.08264	1.15990	-7.82846
C	-3.21740	0.95119	-7.03508
C	-4.47119	0.95058	-7.65417
C	-4.59156	1.15100	-9.02888
C	-3.45554	1.36519	-9.80601
C	-2.19909	1.37082	-9.20018
C	-3.11263	0.65214	-5.55358
N	-2.51008	1.73036	-4.74707
C	-2.98075	3.06669	-5.12599
C	-4.47706	3.28466	-5.00108
C	-5.14263	4.03806	-5.97422
C	-6.49925	4.33094	-5.84823
C	-7.21656	3.86200	-4.74886
C	-6.56601	3.10103	-3.77861
C	-5.20679	2.81728	-3.90410
C	-1.96260	1.49059	-3.49842
N	-1.94437	0.29267	-2.85921
C	-2.32645	-1.00636	-3.29912
C	-1.36761	-1.84281	-3.94108
C	-1.71989	-3.14758	-4.29769
C	-2.97703	-3.66645	-4.02045
C	-3.89800	-2.86473	-3.36203
C	-3.60180	-1.54896	-2.99035
C	0.03867	-1.37410	-4.28524

C	0.26558	-1.29563	-5.80208
C	-4.67363	-0.77606	-2.23955
C	-5.99064	-0.66820	-3.02127
N	-1.41602	2.47013	-2.72749
Y	-1.19262	1.19178	-0.74939
C	1.66784	1.42014	-0.66977
C	1.55569	0.36008	-1.29061
C	-0.86004	3.72636	-3.10739
C	-1.44698	4.92697	-2.61773
C	-0.79767	6.14693	-2.82589
C	0.39535	6.22571	-3.53013
C	0.94333	5.06079	-4.04844
C	0.34885	3.81027	-3.85180
C	-2.78334	4.94482	-1.89439
C	-2.61250	5.24911	-0.40205
C	1.02210	2.60781	-4.49228
C	0.89987	2.68400	-6.02160
C	2.49931	2.46637	-4.09921
C	-3.77759	5.93260	-2.52255
C	-3.44546	1.83067	0.24956
Al	-2.31136	1.88985	2.05393
C	-2.42184	3.68742	2.87756
Y	0.89942	0.03784	1.60138
C	-0.70831	-1.17460	-0.05204
Al	2.40490	2.95236	0.48264
C	1.13506	4.44216	0.71402
O	-0.66928	1.44582	1.39072
C	-3.13833	0.43856	3.11854
C	2.66930	1.92405	2.25952
N	1.05942	-1.05330	3.67510
C	1.99795	-1.98021	3.31007
N	2.69454	-2.72716	4.24174
C	2.90547	-2.24073	5.61523
C	4.29368	-1.68657	5.87453

C	5.15449	-2.32764	6.77079
C	6.41309	-1.79827	7.05680
C	6.82890	-0.61685	6.44718
C	5.97855	0.03270	5.55152
C	4.72263	-0.49914	5.26896
N	2.16809	-2.01726	1.97388
C	3.01454	-2.81240	1.14963
C	2.47049	-3.96820	0.51716
C	3.24638	-4.66729	-0.41176
C	4.53179	-4.26077	-0.74478
C	5.06475	-3.14386	-0.11698
C	4.34031	-2.41572	0.83285
C	1.09521	-4.52525	0.85891
C	1.20804	-5.83019	1.66432
C	5.02121	-1.22295	1.48180
C	5.20586	-0.07726	0.47835
C	4.15237	3.43032	-0.32312
C	0.24032	-1.08229	4.84318
C	0.24433	0.02741	5.73153
C	-0.66641	0.06678	6.79215
C	-1.56078	-0.96644	7.02132
C	-1.54222	-2.06726	6.17447
C	-0.66766	-2.15233	5.08761
C	1.22559	1.18144	5.60622
C	2.04392	1.39123	6.88988
C	-0.78383	-3.36130	4.17051
C	-1.81339	-3.10321	3.06231
C	0.51584	2.48648	5.21926
C	-1.14751	-4.65752	4.90892
C	0.22236	-4.77090	-0.37934
C	6.37657	-1.58415	2.10703
C	1.11557	-2.27732	-3.66462
C	-4.94165	-1.41100	-0.86515
C	3.62846	-3.78911	3.86089

C	3.65404	-4.97478	4.80328
C	4.87891	-5.57165	5.11841
C	4.93246	-6.71357	5.91622
C	3.75846	-7.26926	6.42182
C	2.53259	-6.67747	6.11954
C	2.48215	-5.54202	5.31338
H	1.52920	-0.53400	-1.87872
H	-3.42675	4.11396	2.74864
H	-1.71081	4.42725	2.49387
H	-2.25796	3.62001	3.96122
H	-3.38030	-0.46014	2.53702
H	-4.08140	0.78833	3.56233
H	-2.50774	0.11656	3.95741
H	1.63269	5.28858	1.20773
H	0.26920	4.17565	1.32838
H	0.76611	4.82307	-0.24714
H	4.77938	2.58475	-0.62415
H	4.74113	4.03603	0.38081
H	3.99988	4.05400	-1.21337
H	2.72065	-3.07215	6.30192
H	2.15796	-1.48345	5.83505
H	4.83455	-3.25017	7.24891
H	7.06707	-2.31004	7.75774
H	7.80712	-0.20028	6.67095
H	6.29030	0.96110	5.08031
H	4.05973	0.02122	4.58262
H	4.64427	-3.38251	3.78618
H	3.36257	-4.14569	2.86781
H	5.79933	-5.13582	4.73678
H	5.89359	-7.16427	6.14851
H	3.79835	-8.15540	7.04911
H	1.61143	-7.10182	6.50987
H	1.52704	-5.08201	5.08141
H	-0.66292	0.92695	7.45645

H	-2.26224	-0.91960	7.84949
H	-2.24207	-2.87770	6.35295
H	1.92950	0.92202	4.80943
H	1.41075	1.71822	7.72159
H	2.80231	2.16597	6.73219
H	2.56005	0.47881	7.20204
H	-0.04713	2.39141	4.28628
H	1.23631	3.30330	5.09809
H	-0.19742	2.78621	5.99496
H	0.18774	-3.52317	3.69448
H	-2.80806	-2.95014	3.49407
H	-1.86635	-3.95144	2.37023
H	-1.56912	-2.20887	2.48641
H	-0.54177	-4.80399	5.80852
H	-1.00079	-5.51922	4.24956
H	-2.19911	-4.66854	5.21466
H	2.83070	-5.55421	-0.88276
H	5.11442	-4.81377	-1.47639
H	6.07480	-2.82716	-0.36370
H	0.58877	-3.78444	1.48540
H	1.71598	-6.60614	1.08059
H	0.21196	-6.20849	1.92046
H	1.76672	-5.70272	2.59505
H	0.11412	-3.87245	-0.98995
H	-0.78127	-5.08929	-0.07724
H	0.63559	-5.56193	-1.01460
H	4.37195	-0.87333	2.29181
H	5.87833	-0.37919	-0.33263
H	5.64237	0.80212	0.96299
H	4.25998	0.22603	0.02188
H	6.29650	-2.41302	2.81548
H	6.78489	-0.72463	2.64743
H	7.10729	-1.86943	1.34263
H	-4.11640	0.42234	-5.17822

H	-2.51568	-0.24786	-5.42547
H	-5.36271	0.79534	-7.05222
H	-5.57543	1.14453	-9.48994
H	-3.54681	1.52501	-10.87681
H	-1.30615	1.53347	-9.79770
H	-1.10027	1.16316	-7.36760
H	-2.68459	3.25034	-6.16310
H	-2.45810	3.80780	-4.52466
H	-4.59146	4.39547	-6.84094
H	-6.99730	4.91957	-6.61397
H	-8.27519	4.08521	-4.64974
H	-7.11644	2.72927	-2.91867
H	-4.70739	2.22854	-3.13969
H	-0.98727	-3.77096	-4.80422
H	-3.23180	-4.68316	-4.30598
H	-4.87915	-3.26863	-3.12550
H	0.15560	-0.36641	-3.87312
H	0.18812	-2.28698	-6.26233
H	1.26523	-0.90547	-6.02291
H	-0.46521	-0.65045	-6.29428
H	0.97295	-2.41814	-2.58872
H	2.11578	-1.86150	-3.83444
H	1.10563	-3.27649	-4.11256
H	-4.29687	0.23924	-2.07893
H	-5.85602	-0.18276	-3.99162
H	-6.72108	-0.08112	-2.45407
H	-6.43194	-1.65460	-3.19962
H	-5.36233	-2.41651	-0.97552
H	-5.65755	-0.81308	-0.29045
H	-4.02524	-1.50833	-0.27534
H	1.86818	5.11749	-4.61654
H	0.88715	7.18256	-3.68109
H	-1.24597	7.05465	-2.43101
H	0.49926	1.70937	-4.15036

H	-0.13952	2.80939	-6.33431
H	1.29983	1.77896	-6.49322
H	1.46578	3.53703	-6.41259
H	3.09902	3.29458	-4.49165
H	2.91534	1.54239	-4.51789
H	2.63279	2.43909	-3.01634
H	-3.21522	3.94372	-1.99605
H	-1.93199	4.54819	0.08797
H	-3.57417	5.20309	0.11984
H	-2.19776	6.25251	-0.25489
H	-3.45540	6.97083	-2.38771
H	-4.75819	5.83040	-2.04570
H	-3.90879	5.75663	-3.59373
H	3.41491	2.63443	2.64563
H	3.20265	0.96205	2.34877
H	-0.72749	-1.52228	-1.09506
H	-0.16763	-1.98893	0.45650
H	-1.74480	-1.28857	0.31561
H	1.87641	1.97530	3.01876
H	-4.25615	2.30066	0.82371
H	-3.42943	2.44513	-0.66558
H	-3.85590	0.84624	-0.01713

216

TS4 without dispersion

C	3.23556	3.57613	-1.80463
C	2.02504	3.23678	-1.19114
C	0.90138	3.03297	-1.99767
C	0.98552	3.15570	-3.38420
C	2.19594	3.49702	-3.98414
C	3.32149	3.71047	-3.18846
C	1.92660	3.00814	0.30295
N	2.45878	4.10348	1.12547
C	1.96035	5.42246	0.71637
C	0.44990	5.55881	0.72674

C	-0.21266	6.05149	-0.40185
C	-1.59124	6.26072	-0.38752
C	-2.32986	5.97029	0.75750
C	-1.68070	5.47153	1.88691
C	-0.30242	5.26922	1.87064
C	3.05071	3.90066	2.35690
N	3.15091	2.71822	3.00696
C	2.72945	1.41352	2.62943
C	3.66579	0.53689	2.01080
C	3.28314	-0.77371	1.71227
C	2.01693	-1.25213	2.02304
C	1.11729	-0.40599	2.65595
C	1.44482	0.91687	2.97275
C	5.07273	0.97530	1.63171
C	5.28500	0.97106	0.11030
C	0.40058	1.74984	3.69895
C	-0.91061	1.87164	2.90991
N	3.58594	4.91449	3.09626
Y	4.08087	3.66659	5.02815
C	6.56948	3.41817	5.23871
C	6.95225	2.26304	5.01706
C	4.11102	6.16916	2.66961
C	3.53301	7.36796	3.17313
C	4.16224	8.59171	2.92937
C	5.32961	8.67256	2.18371
C	5.86461	7.50819	1.64976
C	5.28166	6.25575	1.86732
C	2.22185	7.38217	3.94258
C	2.42180	7.81317	5.40025
C	5.95678	5.04854	1.23379
C	6.17229	5.24905	-0.27458
C	7.29711	4.72713	1.90867
C	1.16979	8.27899	3.27176
C	1.88884	4.27483	6.35605

Al	3.26565	4.57844	7.88659
C	2.85802	6.23800	8.86765
Y	5.68856	2.44312	7.77523
C	3.98132	1.39076	6.21035
Al	6.85936	5.20334	6.61324
C	6.09200	6.99938	6.97906
O	4.82629	4.36302	6.98961
C	3.12811	3.00589	9.15576
C	7.51480	4.18701	8.31350
N	6.05896	1.28976	9.82312
C	6.99637	0.41639	9.35911
N	7.73274	-0.39567	10.20438
C	7.99497	-0.01047	11.60022
C	9.38999	0.53282	11.84941
C	10.26357	-0.14094	12.70860
C	11.52821	0.37474	12.99258
C	11.93834	1.57484	12.41660
C	11.07557	2.25704	11.55789
C	9.81285	1.73952	11.27849
N	7.12091	0.48534	8.01584
C	7.92667	-0.25731	7.10558
C	7.33256	-1.34720	6.40315
C	8.04533	-1.96867	5.37322
C	9.32025	-1.55212	5.01255
C	9.91053	-0.51460	5.72062
C	9.25135	0.13479	6.77086
C	5.97374	-1.92563	6.77603
C	6.12961	-3.28141	7.48506
C	10.01157	1.22352	7.51175
C	10.22652	2.46477	6.63655
C	8.51896	5.63646	5.56379
C	5.30667	1.20536	11.03011
C	5.36493	2.27778	11.96320
C	4.47717	2.30422	13.04259

C	3.54602	1.29641	13.24340
C	3.51990	0.22608	12.35932
C	4.38052	0.14881	11.25949
C	6.38632	3.40083	11.86359
C	7.25955	3.49314	13.12505
C	4.24381	-1.06177	10.34437
C	3.07192	-0.91315	9.36300
C	5.72661	4.76055	11.59206
C	4.07354	-2.37228	11.13047
C	5.03389	-2.09611	5.57478
C	11.37128	0.73207	8.03362
C	6.14214	0.09979	2.29811
C	0.10629	1.17114	5.09261
C	8.66810	-1.41079	9.71145
C	8.76191	-2.65421	10.57185
C	10.01640	-3.20961	10.84303
C	10.13060	-4.39700	11.56474
C	8.98868	-5.04174	12.03668
C	7.73323	-4.49298	11.77706
C	7.62304	-3.31162	11.04681
H	7.35540	1.29455	4.79626
H	1.90307	6.13227	9.40254
H	2.77687	7.13175	8.24090
H	3.61813	6.45324	9.62907
H	3.57208	2.00022	9.12207
H	2.05469	2.78174	9.04792
H	3.26185	3.33396	10.19259
H	6.90172	7.70531	7.21099
H	5.35897	7.04377	7.79114
H	5.60135	7.40109	6.08053
H	9.12253	4.80688	5.18123
H	9.17586	6.26544	6.18495
H	8.24114	6.25598	4.69936
H	7.83954	-0.89108	12.23097

H	7.25396	0.72352	11.90496
H	9.94917	-1.07972	13.15784
H	12.19181	-0.16306	13.66440
H	12.92187	1.98023	12.63755
H	11.38262	3.19965	11.11262
H	9.14214	2.28687	10.62137
H	9.67075	-0.97793	9.62066
H	8.36307	-1.70875	8.71031
H	10.91224	-2.70484	10.48935
H	11.11411	-4.81393	11.76410
H	9.07609	-5.96378	12.60462
H	6.83618	-4.98661	12.14124
H	6.64589	-2.88521	10.84742
H	4.52360	3.13478	13.74236
H	2.85767	1.33607	14.08299
H	2.80079	-0.57168	12.52172
H	7.04355	3.16470	11.02076
H	6.66945	3.79078	13.99872
H	8.04576	4.24357	12.98840
H	7.74518	2.54195	13.35964
H	5.12165	4.75323	10.68109
H	6.48334	5.54616	11.48892
H	5.06308	5.04514	12.41637
H	5.16256	-1.15021	9.75733
H	2.12919	-0.78364	9.90616
H	2.97959	-1.80593	8.73396
H	3.19313	-0.05244	8.70210
H	4.79362	-2.45588	11.94986
H	4.20107	-3.23293	10.46495
H	3.07174	-2.45518	11.56516
H	7.58866	-2.80145	4.84512
H	9.85292	-2.04100	4.20158
H	10.91667	-0.19822	5.45842
H	5.50362	-1.23085	7.47952

H	6.59692	-4.01620	6.81976
H	5.14941	-3.67519	7.77690
H	6.74624	-3.21653	8.38480
H	4.90826	-1.17093	5.00903
H	4.04242	-2.41447	5.91415
H	5.39949	-2.86275	4.88298
H	9.40951	1.51189	8.37984
H	10.85195	2.22339	5.76921
H	10.73554	3.25366	7.20093
H	9.28743	2.87602	6.26291
H	11.28565	-0.17993	8.63038
H	11.83234	1.49921	8.66306
H	12.06266	0.52314	7.21021
H	0.87487	2.82991	0.55718
H	2.46727	2.09659	0.54710
H	-0.04916	2.78039	-1.53430
H	0.10149	2.99094	-3.99422
H	2.26240	3.59788	-5.06388
H	4.27031	3.97795	-3.64571
H	4.11339	3.74334	-1.18942
H	2.32708	5.63186	-0.29311
H	2.39622	6.17831	1.36507
H	0.35798	6.27082	-1.30094
H	-2.08749	6.64658	-1.27392
H	-3.40431	6.13097	0.77139
H	-2.24851	5.24433	2.78508
H	0.19669	4.88813	2.75772
H	3.99665	-1.43300	1.22429
H	1.73763	-2.27392	1.78145
H	0.13047	-0.78071	2.91643
H	5.20739	1.99955	1.99357
H	5.20713	-0.04454	-0.29375
H	6.28299	1.34894	-0.13856
H	4.55021	1.58676	-0.41424

H	5.99969	0.04149	3.37955
H	7.14269	0.50095	2.10244
H	6.11903	-0.92484	1.91090
H	0.81222	2.75699	3.82723
H	-0.76000	2.32301	1.92615
H	-1.62683	2.49567	3.45534
H	-1.37706	0.89177	2.76137
H	-0.36703	0.18614	5.01404
H	-0.57684	1.82383	5.64758
H	1.01875	1.04496	5.68189
H	6.77198	7.56754	1.05461
H	5.81202	9.63074	2.01259
H	3.72120	9.49990	3.33184
H	5.29925	4.18329	1.36160
H	5.25705	5.57366	-0.77792
H	6.51525	4.31777	-0.73958
H	6.93944	6.00536	-0.47153
H	8.00217	5.55658	1.78587
H	7.75082	3.83538	1.46008
H	7.18555	4.54728	2.97962
H	1.83460	6.35720	3.93536
H	3.16372	7.19654	5.91355
H	1.48068	7.75019	5.95768
H	2.77335	8.84932	5.45597
H	1.46984	9.33222	3.29581
H	0.21262	8.19854	3.79847
H	0.99895	8.00323	2.22762
H	8.31564	4.92807	8.44029
H	8.06936	3.23534	8.34840
H	3.74910	1.07748	5.17684
H	4.57046	0.52083	6.55069
H	3.02814	1.31952	6.75226
H	6.92536	4.27838	9.23796
H	1.02120	4.51519	6.98690

H	1.78781	4.98584	5.52127
H	1.64530	3.26924	5.97976

216

Complex9 without dispersion

C	-2.04799	1.31592	-7.73209
C	-3.20726	0.86665	-7.09198
C	-4.31826	0.53630	-7.87443
C	-4.26993	0.64231	-9.26373
C	-3.11000	1.09325	-9.89126
C	-1.99916	1.43266	-9.11958
C	-3.26373	0.65659	-5.59255
N	-2.67401	1.74407	-4.80612
C	-3.23608	3.06152	-5.14211
C	-4.73943	3.16258	-4.96691
C	-5.55758	3.44573	-6.06482
C	-6.93419	3.60661	-5.90846
C	-7.51255	3.48341	-4.64717
C	-6.70685	3.19779	-3.54444
C	-5.33249	3.03798	-3.70458
C	-1.99453	1.53635	-3.61948
N	-1.91690	0.37133	-2.93830
C	-2.36649	-0.93593	-3.28281
C	-1.45829	-1.82226	-3.93083
C	-1.84632	-3.14299	-4.17039
C	-3.09155	-3.62007	-3.78261
C	-3.97188	-2.75616	-3.14746
C	-3.64122	-1.42105	-2.88910
C	-0.08940	-1.38120	-4.43068
C	-0.01749	-1.40895	-5.96607
C	-4.68367	-0.56273	-2.18924
C	-6.00025	-0.48243	-2.97603
N	-1.34387	2.54086	-2.96584
Y	-0.87850	1.32358	-0.98987
C	1.31950	0.29217	-0.87217

C	2.30914	-0.42549	-1.02616
C	-0.80746	3.73855	-3.52197
C	-1.25790	4.99368	-3.03022
C	-0.62298	6.16834	-3.44349
C	0.42895	6.14367	-4.34615
C	0.84826	4.92032	-4.85269
C	0.25793	3.71371	-4.46495
C	-2.42979	5.11597	-2.07180
C	-1.96781	5.55820	-0.67864
C	0.83354	2.42403	-5.03106
C	1.25664	2.54930	-6.50269
C	2.02885	1.93903	-4.19851
C	-3.52051	6.06393	-2.59223
C	-3.12322	1.97882	0.24224
Al	-1.88829	2.24337	1.88432
C	-2.33071	3.97896	2.71206
Y	0.78864	0.01604	1.64664
C	-1.16382	-0.89291	0.36000
Al	1.28200	3.16303	1.21315
C	1.08327	4.73744	2.38984
O	-0.21690	2.06479	1.10098
C	-2.12103	0.74418	3.18121
C	2.61744	1.85057	2.09807
N	1.05409	-1.09688	3.73422
C	2.03637	-1.96011	3.34822
N	2.68448	-2.81080	4.22551
C	2.76513	-2.53657	5.66570
C	4.11595	-2.04180	6.14668
C	4.73111	-2.65617	7.24209
C	5.92922	-2.16451	7.75926
C	6.53516	-1.05153	7.18024
C	5.93436	-0.43464	6.08303
C	4.73498	-0.92606	5.57222
N	2.28761	-1.84671	2.02494

C	3.18385	-2.56049	1.18122
C	2.69088	-3.68212	0.45407
C	3.50421	-4.29475	-0.50230
C	4.78995	-3.83952	-0.76519
C	5.27604	-2.75733	-0.04433
C	4.50580	-2.10497	0.92639
C	1.31098	-4.27641	0.69751
C	1.39729	-5.73555	1.17277
C	5.14549	-0.92652	1.64625
C	5.50448	0.21084	0.67511
C	1.85663	3.62838	-0.63565
C	0.24437	-1.14821	4.90718
C	0.32133	-0.08630	5.84976
C	-0.59522	-0.02985	6.90268
C	-1.57386	-1.00020	7.06615
C	-1.62457	-2.05724	6.16816
C	-0.73723	-2.15894	5.09169
C	1.38833	0.99292	5.77164
C	2.17751	1.13292	7.08168
C	-0.90330	-3.34991	4.15946
C	-2.18094	-3.23939	3.31405
C	0.78971	2.34738	5.37318
C	-0.91495	-4.67827	4.93115
C	0.41625	-4.19452	-0.54572
C	6.40683	-1.33915	2.42030
C	1.04855	-2.22980	-3.85108
C	-4.97135	-1.07927	-0.77041
C	3.66595	-3.81259	3.78481
C	3.68824	-5.07857	4.61666
C	4.89125	-5.52633	5.17055
C	4.95008	-6.72975	5.87325
C	3.80027	-7.49850	6.04073
C	2.59350	-7.05894	5.49633
C	2.54156	-5.86162	4.78654

H	3.16659	-1.04868	-1.17695
H	-3.41747	4.04182	2.87090
H	-2.03762	4.87010	2.15001
H	-1.86993	4.06401	3.70400
H	-1.48907	-0.15468	3.18714
H	-3.13642	0.34579	3.03511
H	-2.08295	1.12199	4.21009
H	2.07448	5.18142	2.56593
H	0.63749	4.55255	3.37203
H	0.48092	5.51883	1.90923
H	2.59010	2.92814	-1.05036
H	2.33667	4.61712	-0.59942
H	1.06809	3.74370	-1.39564
H	2.51352	-3.45584	6.20402
H	2.00248	-1.80860	5.92984
H	4.26601	-3.52983	7.69229
H	6.38989	-2.65483	8.61265
H	7.46877	-0.66597	7.58042
H	6.39743	0.43656	5.62765
H	4.26981	-0.43117	4.72382
H	4.67162	-3.37705	3.79441
H	3.44941	-4.08221	2.75396
H	5.78914	-4.92474	5.05315
H	5.89512	-7.06248	6.29393
H	3.84297	-8.43430	6.59110
H	1.69147	-7.65237	5.61913
H	1.60240	-5.52536	4.36070
H	-0.53636	0.79254	7.61088
H	-2.28323	-0.93831	7.88672
H	-2.38522	-2.82286	6.29655
H	2.09444	0.68730	4.99191
H	1.54680	1.51092	7.89352
H	3.00088	1.84349	6.95132
H	2.60641	0.18057	7.40492

H	0.25016	2.29439	4.42348
H	1.57150	3.10762	5.27435
H	0.07688	2.69469	6.12951
H	-0.04997	-3.36904	3.47487
H	-3.06968	-3.23198	3.95475
H	-2.26709	-4.09257	2.63146
H	-2.19814	-2.32546	2.71731
H	-0.06539	-4.75878	5.61446
H	-0.88552	-5.52539	4.23632
H	-1.82738	-4.78648	5.52716
H	3.11725	-5.14892	-1.05185
H	5.40718	-4.32608	-1.51527
H	6.28505	-2.40210	-0.23804
H	0.83709	-3.68921	1.49063
H	1.80024	-6.38423	0.38707
H	0.40040	-6.11286	1.42691
H	2.03738	-5.85175	2.05135
H	0.31107	-3.16861	-0.90311
H	-0.58531	-4.57894	-0.32388
H	0.82461	-4.79327	-1.36729
H	4.41635	-0.54253	2.36725
H	6.28424	-0.10520	-0.02702
H	5.88971	1.07603	1.22557
H	4.64484	0.54280	0.08671
H	6.20322	-2.11528	3.16172
H	6.82717	-0.47760	2.94957
H	7.18027	-1.71854	1.74379
H	-4.31081	0.50943	-5.30228
H	-2.74219	-0.26830	-5.35544
H	-5.23066	0.19607	-7.39027
H	-5.14293	0.37917	-9.85496
H	-3.07240	1.18225	-10.97342
H	-1.09010	1.78690	-9.59821
H	-1.18322	1.58234	-7.13343

H	-2.98319	3.28948	-6.18199
H	-2.74664	3.81592	-4.53262
H	-5.11042	3.53929	-7.05127
H	-7.55342	3.82848	-6.77347
H	-8.58421	3.61125	-4.52161
H	-7.14913	3.10625	-2.55602
H	-4.71096	2.82373	-2.83887
H	-1.15365	-3.81142	-4.67536
H	-3.37260	-4.65185	-3.97506
H	-4.94799	-3.12423	-2.84156
H	0.06951	-0.35184	-4.09667
H	-0.13860	-2.42965	-6.34596
H	0.95757	-1.04495	-6.30905
H	-0.78914	-0.79297	-6.43513
H	1.05854	-2.19252	-2.76094
H	2.01507	-1.85757	-4.20864
H	0.96727	-3.27837	-4.15852
H	-4.28042	0.45240	-2.11216
H	-5.85881	-0.06712	-3.97683
H	-6.71752	0.15816	-2.45253
H	-6.45911	-1.47098	-3.08506
H	-5.44376	-2.06735	-0.80390
H	-5.65471	-0.40370	-0.24384
H	-4.05813	-1.17632	-0.17768
H	1.67035	4.89928	-5.56162
H	0.91516	7.06448	-4.65603
H	-0.96848	7.12066	-3.04998
H	0.05637	1.65547	-4.98139
H	0.49051	3.03822	-7.11240
H	1.45325	1.55759	-6.92344
H	2.18092	3.12697	-6.61047
H	2.82848	2.68815	-4.20858
H	2.43231	1.00558	-4.60805
H	1.76191	1.75621	-3.15558

H -2.87506 4.11890 -1.98518
H -1.21615 4.88137 -0.26103
H -2.81101 5.59836 0.01818
H -1.51536 6.55543 -0.71694
H -3.16117 7.09683 -2.65299
H -4.38150 6.05730 -1.91489
H -3.87534 5.77400 -3.58535
H 3.40924 2.61336 2.12696
H 3.12154 1.02263 1.57768
H -1.43454 -1.19311 -0.66816
H -0.67228 -1.82287 0.70593
H -2.10508 -0.84093 0.92013
H 2.49220 1.56945 3.15426
H -4.02890 2.05774 0.86141
H -3.22162 2.80872 -0.47200
H -3.30843 1.03974 -0.30239
2
CO
C -0.30574 -3.96313 1.59426
O -0.69199 -4.69365 2.37526
214
Complex 1
C 4.03742 -0.78235 -2.64631
C 2.88480 -1.52655 -3.00777
C 2.47032 -1.54437 -4.35916
C 3.23716 -0.86656 -5.31237
C 4.38158 -0.16294 -4.96652
C 4.76628 -0.11772 -3.63209
N 2.15983 -2.12462 -1.94808
C 1.97952 -3.42073 -1.65606
N 2.49690 -4.49018 -2.35320
C 3.66807 -4.34604 -3.20336
C 4.53635 -5.58220 -3.25657
C 5.31348 -5.79977 -4.39707

C	6.17512	-6.89128	-4.47528
C	6.25834	-7.79193	-3.41508
C	5.47556	-7.59091	-2.27962
C	4.62212	-6.49374	-2.19826
C	1.19027	-2.20635	-4.81659
C	0.16490	-1.14550	-5.23654
C	4.47984	-0.66369	-1.20216
C	4.15541	0.73147	-0.65594
Y	0.44453	-1.39701	-0.44987
C	1.82319	-0.87997	1.62802
Al	0.01796	-1.46811	2.48493
C	0.26914	-2.93949	3.76181
Y	-1.81008	0.65199	1.36148
C	-0.31860	0.34363	3.45315
Al	-2.52951	-1.58304	-0.52198
C	-3.86010	-3.00126	-0.81780
N	-1.93798	3.01932	1.73040
C	-1.23426	4.13029	1.22467
C	-0.00601	4.54510	1.78897
C	0.70804	5.58072	1.18247
C	0.24423	6.20950	0.03431
C	-0.95037	5.78164	-0.53192
C	-1.69230	4.74324	0.03265
C	0.57172	3.88960	3.02543
C	1.87399	3.14784	2.69902
C	-2.97603	4.30750	-0.64422
C	-2.74424	3.86601	-2.09250
N	-3.57081	1.67675	2.45203
C	-4.86957	1.21518	2.69967
C	-6.03137	1.83916	2.17612
C	-7.28392	1.29694	2.47708
C	-7.42193	0.13961	3.22934
C	-6.27642	-0.52303	3.65612
C	-5.00432	-0.02116	3.38832

C	-5.98508	3.02817	1.23577
C	-6.50076	2.62802	-0.15240
C	-3.79236	-0.79931	3.85900
C	-3.93022	-2.31162	3.68812
C	-0.04844	1.18019	-0.35706
C	-1.32425	-1.70142	1.04611
C	-3.57865	0.21530	-0.48038
N	1.17497	-3.61981	-0.57988
C	1.39900	-4.73280	0.26128
C	2.69303	-4.97456	0.79901
C	2.92947	-6.15727	1.50128
C	1.91106	-7.06928	1.75269
C	0.62165	-6.76121	1.34261
C	0.34021	-5.60539	0.60755
C	3.77482	-3.90707	0.82482
C	3.93727	-3.42089	2.27371
C	-1.10030	-5.33003	0.23025
C	-1.95745	-5.17225	1.49168
C	-1.39433	-1.31113	-2.23224
C	-3.06929	2.93355	2.45296
N	-3.62270	3.94936	3.20064
C	-4.33358	3.60088	4.42912
C	-3.43119	3.43310	5.62782
C	-2.10446	3.02478	5.48880
C	-1.29328	2.84022	6.60584
C	-1.80405	3.06130	7.88277
C	-3.12901	3.47230	8.03149
C	-3.93388	3.66132	6.91134
C	-2.95518	5.26219	3.23289
C	-3.76554	6.30595	3.95106
C	-3.43530	6.68072	5.25588
C	-4.19648	7.63031	5.93377
C	-5.29687	8.21632	5.31144
C	-5.62676	7.85656	4.00487

C	-4.86193	6.91004	3.32918
C	1.65851	-5.67977	-2.55278
C	0.53853	-5.52925	-3.55227
C	0.54550	-6.26912	-4.73605
C	-0.52101	-6.19164	-5.63155
C	-1.60321	-5.35795	-5.35751
C	-1.61947	-4.61159	-4.17813
C	-0.56181	-4.70821	-3.28048
C	0.80277	4.88032	4.17076
C	-4.03557	5.41131	-0.57317
C	-6.76205	4.22927	1.77919
C	-3.41088	-0.42388	5.29242
C	5.13682	-4.32590	0.27135
C	-1.68712	-6.42468	-0.66685
C	1.39480	-3.19246	-5.96883
C	5.96053	-0.99620	-1.00658
H	0.76798	1.02948	-1.09032
H	-0.80695	1.70151	-0.96419
H	0.36767	1.96739	0.29246
H	-1.87281	-2.52584	1.52834
H	-4.39827	0.25596	0.24956
H	-3.21806	1.24806	-0.62718
H	-4.06591	-0.00071	-1.44032
H	-2.25170	-1.33786	-2.91585
H	-0.94796	-0.32210	-2.41878
H	-0.72193	-2.06884	-2.65995
H	-4.46633	-3.19937	0.07434
H	-3.39299	-3.94914	-1.10801
H	-4.55955	-2.72948	-1.61962
H	2.34326	-0.76141	2.58577
H	1.87545	0.12131	1.17550
H	2.48992	-1.54699	1.06421
H	-1.26645	0.67066	3.90666
H	0.28601	0.06195	4.32457

H	0.18912	1.23063	3.05515
H	0.58094	-3.85366	3.24179
H	-0.64415	-3.18275	4.31882
H	1.04247	-2.71100	4.50719
H	-2.77789	5.58197	2.20744
H	-1.97714	5.17228	3.71476
H	-2.58794	6.21011	5.74600
H	-3.93069	7.90968	6.94912
H	-5.89249	8.95538	5.83942
H	-6.47727	8.31714	3.51065
H	-5.11764	6.63155	2.31176
H	-4.90211	2.68601	4.26666
H	-5.06568	4.38587	4.63293
H	-1.70037	2.85159	4.49884
H	-0.26403	2.52036	6.47236
H	-1.17509	2.91862	8.75648
H	-3.53367	3.65417	9.02309
H	-4.96005	4.00051	7.02965
H	1.64856	5.89975	1.62394
H	0.81091	7.01731	-0.41902
H	-1.31442	6.25773	-1.43865
H	-0.15969	3.15272	3.36473
H	-0.11570	5.40385	4.44940
H	1.54534	5.63732	3.89801
H	1.17059	4.35769	5.05958
H	2.24687	2.61007	3.57750
H	2.65179	3.84817	2.37691
H	1.73406	2.42356	1.89182
H	-3.35752	3.43977	-0.09922
H	-4.27196	5.66276	0.46422
H	-4.96184	5.09735	-1.06339
H	-3.68579	6.32416	-1.06648
H	-1.97869	3.08679	-2.15070
H	-2.41361	4.70019	-2.72015

H	-3.67044	3.47017	-2.52238
H	-8.17179	1.78893	2.08904
H	-8.40635	-0.25997	3.45355
H	-6.37132	-1.45464	4.20503
H	-4.94687	3.33692	1.11927
H	-6.37769	4.54678	2.75066
H	-7.82547	3.99607	1.89705
H	-6.68704	5.07652	1.08951
H	-6.45610	3.47832	-0.84076
H	-7.54176	2.29176	-0.10682
H	-5.90302	1.81510	-0.57219
H	-2.94166	-0.51935	3.22670
H	-3.24727	0.65201	5.38994
H	-2.49636	-0.94174	5.60088
H	-4.21452	-0.70553	5.98153
H	-4.23142	-2.56765	2.66852
H	-4.66199	-2.73852	4.38139
H	-2.96942	-2.79442	3.88804
H	1.25378	-5.98834	-1.58715
H	2.31677	-6.48374	-2.88044
H	1.39285	-6.91487	-4.95446
H	-0.50237	-6.77734	-6.54616
H	-2.43399	-5.29541	-6.05442
H	-2.46451	-3.97101	-3.94357
H	-0.59197	-4.16871	-2.34076
H	4.25951	-3.51665	-2.80587
H	3.40079	-4.05885	-4.22648
H	5.24175	-5.10434	-5.23036
H	6.77432	-7.04294	-5.36858
H	6.92304	-8.64868	-3.47670
H	5.52542	-8.29154	-1.45099
H	4.01036	-6.34811	-1.31297
H	3.92499	-6.34426	1.89597
H	2.11313	-7.98617	2.29866

H	-0.19333	-7.43704	1.58620
H	3.42756	-3.06404	0.22452
H	5.11635	-4.47234	-0.80829
H	5.48894	-5.25840	0.72455
H	5.88056	-3.55430	0.49090
H	4.57631	-2.53141	2.31330
H	4.40263	-4.19850	2.88859
H	2.97178	-3.17909	2.72258
H	-1.12280	-4.37565	-0.30421
H	-1.09364	-6.57907	-1.57011
H	-2.70445	-6.16278	-0.97610
H	-1.73989	-7.37892	-0.13128
H	-1.51514	-4.44752	2.17734
H	-2.04827	-6.12417	2.02643
H	-2.96533	-4.83683	1.22997
H	2.91620	-0.88582	-6.35010
H	4.96087	0.35478	-5.72555
H	5.65031	0.44568	-3.34687
H	0.77938	-2.76648	-3.97508
H	2.09154	-3.99232	-5.70998
H	1.77740	-2.68709	-6.86192
H	0.44301	-3.66168	-6.22798
H	0.03377	-0.38412	-4.46480
H	-0.80733	-1.61068	-5.43017
H	0.48893	-0.63898	-6.15245
H	3.90029	-1.38921	-0.62846
H	6.20313	-1.98029	-1.41711
H	6.21100	-0.99980	0.05951
H	6.61043	-0.26078	-1.49259
H	3.09607	0.97532	-0.78426
H	4.72981	1.49968	-1.18537
H	4.39188	0.79943	0.41113

216

Int1

C	-2.56059	6.64737	4.55972
C	-2.44983	5.48817	3.78910
C	-1.43764	4.57456	4.09558
C	-0.54944	4.81486	5.13882
C	-0.66595	5.97687	5.90106
C	-1.67756	6.89042	5.61107
C	-3.42062	5.21980	2.66234
N	-2.76474	4.83336	1.40888
C	-1.95137	5.91071	0.82538
C	-2.66710	7.23418	0.77531
C	-2.29785	8.26446	1.64375
C	-2.95176	9.49442	1.60670
C	-3.98352	9.70807	0.69517
C	-4.34746	8.69125	-0.18734
C	-3.68861	7.46577	-0.15029
C	-2.36462	3.52153	1.25054
N	-3.02565	2.49273	1.83905
C	-4.35758	2.43108	2.28413
C	-5.45995	2.68749	1.43098
C	-6.75248	2.48722	1.92207
C	-6.98389	2.01746	3.20883
C	-5.89915	1.74677	4.03455
C	-4.59072	1.95164	3.59806
C	-5.29677	3.17324	0.00515
C	-6.00908	2.26586	-1.00387
C	-3.43469	1.64674	4.52613
C	-3.23599	0.13454	4.67445
Y	-1.61517	0.75736	1.21225
N	-1.28938	3.09439	0.57908
C	-0.37943	3.75911	-0.26912
C	0.81861	4.34022	0.20981
C	1.71923	4.88933	-0.70536
C	1.47881	4.85853	-2.07274
C	0.31747	4.25729	-2.54250

C	-0.61599	3.70219	-1.66659
C	1.17081	4.37287	1.68257
C	2.32207	3.40620	1.98266
C	-1.89117	3.09912	-2.21960
C	-1.66643	2.25691	-3.47706
Y	0.33320	-2.05751	0.03426
C	-1.84619	-2.58654	-1.21443
Al	-2.65901	-1.99172	0.59325
C	-3.97149	-3.30699	1.23882
C	2.20247	-3.26935	-1.78118
N	1.84033	-1.97226	-1.85949
C	1.83123	-1.12815	-2.98911
C	2.53486	0.10229	-2.93438
C	2.35004	1.04545	-3.94655
C	1.51723	0.79214	-5.02814
C	0.85639	-0.42845	-5.10015
C	0.98473	-1.39156	-4.09729
C	3.52017	0.40518	-1.82507
C	3.12095	1.63382	-1.00769
C	0.22592	-2.69523	-4.23183
C	-1.23204	-2.50211	-4.65971
N	3.13555	-3.84364	-2.61420
C	4.13245	-3.00620	-3.27632
C	5.39477	-2.81631	-2.47109
C	6.64312	-2.92258	-3.08743
C	7.81259	-2.67607	-2.37088
C	7.74559	-2.32459	-1.02410
C	6.50296	-2.22519	-0.39946
C	5.33825	-2.47382	-1.11851
C	3.57259	-5.23369	-2.41176
C	4.29912	-5.80302	-3.60157
C	3.61837	-6.10016	-4.78648
C	4.29786	-6.62351	-5.88248
C	5.66909	-6.86677	-5.80403

C	6.35290	-6.58830	-4.62274
C	5.67003	-6.05899	-3.52931
N	1.56200	-3.89921	-0.77625
C	1.68380	-5.21535	-0.27249
C	2.58143	-5.51450	0.77673
C	2.57275	-6.79381	1.33744
C	1.70316	-7.77843	0.88750
C	0.81825	-7.47898	-0.14106
C	0.78710	-6.21291	-0.72676
C	3.53417	-4.47953	1.33675
C	3.13179	-4.07891	2.76011
C	-0.20665	-5.94376	-1.83814
C	-1.64884	-6.08468	-1.33784
C	4.99161	-4.94750	1.30423
C	0.02942	-6.84827	-3.05214
C	-0.30459	-3.96095	1.59193
C	-1.21595	-1.52255	1.87319
Al	0.37681	-0.83691	2.83275
C	0.10878	1.17726	3.18126
C	2.05026	-0.92802	1.59818
O	-0.69314	-4.69583	2.37759
C	4.93579	0.59720	-2.38423
C	0.94104	-3.63267	-5.21006
C	0.74192	-1.66287	4.58232
C	-3.68920	-0.27553	0.05028
C	-0.28722	0.31374	-0.77138
C	-5.80089	4.61244	-0.13511
C	-3.57126	2.33171	5.88698
C	1.53422	5.77248	2.19114
C	-2.93228	4.19442	-2.47498
H	0.37399	-0.06692	-1.55750
H	-1.21951	0.50056	-1.33013
H	0.14016	1.31307	-0.59797
H	-1.62618	-2.07778	2.73295

H	-4.33555	0.17381	0.81805
H	-3.36364	0.53872	-0.61946
H	-4.38061	-0.84411	-0.58601
H	-2.80308	-2.85383	-1.67839
H	-1.43332	-1.81155	-1.87572
H	-1.24874	-3.49861	-1.35204
H	-4.48312	-2.94635	2.14041
H	-3.50150	-4.26208	1.50317
H	-4.74977	-3.52689	0.49661
H	2.72455	-0.48859	2.34403
H	2.12325	-0.24847	0.73683
H	2.56257	-1.86418	1.32624
H	-0.63027	1.28807	3.98600
H	1.08149	1.34781	3.65838
H	0.01359	2.05458	2.52471
H	1.71809	-1.37532	4.99369
H	0.72304	-2.75827	4.53754
H	-0.01566	-1.36694	5.31956
H	-1.66714	5.61923	-0.18321
H	-1.02964	6.02892	1.40141
H	-1.50110	8.09238	2.36169
H	-2.65453	10.28495	2.28986
H	-4.49664	10.66492	0.66572
H	-5.14114	8.85553	-0.91048
H	-3.96563	6.68094	-0.84558
H	-4.12566	4.44239	2.95259
H	-4.00987	6.11796	2.46332
H	-1.34956	3.66182	3.51916
H	0.23028	4.08992	5.35463
H	0.02585	6.16824	6.71614
H	-1.77587	7.79868	6.19892
H	-3.33743	7.37000	4.32346
H	2.63425	5.34230	-0.33375
H	2.19392	5.29070	-2.76675

H	0.13047	4.21438	-3.61105
H	0.28903	4.04259	2.23610
H	0.77385	6.51613	1.93875
H	2.48336	6.11725	1.76761
H	1.63786	5.75999	3.28032
H	2.53332	3.37781	3.05727
H	3.23318	3.72624	1.46631
H	2.09475	2.39186	1.64921
H	-2.29722	2.44477	-1.44036
H	-3.17490	4.72769	-1.55270
H	-3.86060	3.76773	-2.86897
H	-2.55573	4.92411	-3.20000
H	-0.85166	1.54151	-3.34313
H	-1.42345	2.88028	-4.34428
H	-2.57866	1.70357	-3.72556
H	-7.59764	2.69276	1.27073
H	-7.99864	1.86079	3.56259
H	-6.06839	1.37390	5.04099
H	-4.23084	3.15841	-0.23585
H	-5.27830	5.28472	0.54726
H	-6.87219	4.66910	0.08635
H	-5.65413	4.97663	-1.15771
H	-5.80044	2.60057	-2.02570
H	-7.09526	2.29098	-0.86764
H	-5.68139	1.22896	-0.90776
H	-2.54227	2.05921	4.05056
H	-3.69971	3.41134	5.76942
H	-2.67080	2.16332	6.48694
H	-4.42407	1.94464	6.45485
H	-3.07867	-0.35613	3.70634
H	-4.11472	-0.33219	5.13267
H	-2.36537	-0.09001	5.30010
H	2.69683	-5.84281	-2.20474
H	4.22283	-5.29969	-1.53271

H	6.20581	-5.82579	-2.61383
H	7.41970	-6.77978	-4.55136
H	6.19975	-7.27658	-6.65851
H	3.75597	-6.84688	-6.79697
H	2.55199	-5.91222	-4.85060
H	3.68713	-2.03819	-3.50225
H	4.37721	-3.46607	-4.23690
H	6.69544	-3.20876	-4.13463
H	8.77654	-2.76265	-2.86420
H	8.65562	-2.13094	-0.46388
H	6.43912	-1.94806	0.64876
H	4.37300	-2.37842	-0.63391
H	3.26043	-7.01876	2.14811
H	1.71154	-8.76720	1.33636
H	0.13147	-8.24242	-0.49670
H	3.45598	-3.59445	0.69938
H	5.31821	-5.18464	0.28827
H	5.13719	-5.83909	1.92254
H	5.65318	-4.16554	1.68845
H	3.78669	-3.29037	3.14545
H	3.19653	-4.93570	3.43884
H	2.10571	-3.70686	2.80210
H	-0.05248	-4.91117	-2.16418
H	1.05224	-6.76059	-3.42796
H	-0.65578	-6.58538	-3.86513
H	-0.13918	-7.90116	-2.80241
H	-1.83627	-5.45933	-0.46117
H	-1.86348	-7.12023	-1.05353
H	-2.36053	-5.79664	-2.11883
H	0.20718	-0.63378	-5.94652
H	1.38325	1.53626	-5.80772
H	2.87279	1.99546	-3.88045
H	0.22010	-3.17518	-3.24907
H	1.97680	-3.79191	-4.90825

H	0.94186	-3.20549	-6.21901
H	0.43901	-4.60576	-5.25465
H	-1.73524	-1.74566	-4.05285
H	-1.78214	-3.44331	-4.55705
H	-1.30555	-2.19397	-5.70798
H	3.52730	-0.46264	-1.15998
H	5.24734	-0.24128	-3.01041
H	5.65960	0.68646	-1.56789
H	4.99397	1.50922	-2.98848
H	2.12573	1.53122	-0.57383
H	3.10201	2.53764	-1.62315
H	3.83381	1.79807	-0.19325

216

TS1

C	-2.67056	6.50546	4.66870
C	-2.51166	5.37340	3.86686
C	-1.49558	4.46735	4.18102
C	-0.65054	4.68734	5.26388
C	-0.81572	5.82144	6.05834
C	-1.83153	6.72768	5.75983
C	-3.43824	5.12472	2.69960
N	-2.73475	4.75411	1.46731
C	-1.89260	5.83812	0.93615
C	-2.59179	7.17102	0.90958
C	-2.22657	8.17269	1.81234
C	-2.86675	9.41034	1.79805
C	-3.88093	9.65997	0.87606
C	-4.24128	8.67149	-0.03956
C	-3.59588	7.43842	-0.02557
C	-2.34422	3.44281	1.29376
N	-3.02667	2.40612	1.84147
C	-4.37251	2.35717	2.24954
C	-5.44434	2.63370	1.36541
C	-6.75398	2.44892	1.81577

C	-7.03095	1.97857	3.09309
C	-5.97577	1.69096	3.95083
C	-4.65234	1.87830	3.55395
C	-5.23252	3.12670	-0.05115
C	-5.90631	2.21978	-1.08667
C	-3.52697	1.54599	4.50844
C	-3.34771	0.02804	4.61535
Y	-1.57882	0.68924	1.23184
N	-1.25214	3.01841	0.64347
C	-0.32740	3.70037	-0.17743
C	0.86969	4.25270	0.33538
C	1.78474	4.82939	-0.54740
C	1.55664	4.85589	-1.91708
C	0.39625	4.28230	-2.42135
C	-0.55000	3.69816	-1.57787
C	1.20346	4.22317	1.81194
C	2.31782	3.20679	2.08506
C	-1.82069	3.12586	-2.17261
C	-1.57640	2.31127	-3.44498
Y	0.41391	-2.06896	0.01296
C	-1.82526	-2.64671	-1.15296
Al	-2.64918	-2.08691	0.63445
C	-3.95563	-3.37403	1.32441
C	2.22696	-3.36062	-1.74367
N	1.87067	-2.05534	-1.84363
C	1.83546	-1.23264	-2.98827
C	2.53066	0.00346	-2.96166
C	2.33101	0.92444	-3.99129
C	1.49216	0.64214	-5.06152
C	0.83897	-0.58414	-5.10410
C	0.98168	-1.52602	-4.08297
C	3.51880	0.33519	-1.86226
C	3.10511	1.57053	-1.06079
C	0.22759	-2.83634	-4.18015

C	-1.23535	-2.65662	-4.59734
N	3.15475	-3.94757	-2.56887
C	4.11727	-3.12520	-3.29438
C	5.40622	-2.88699	-2.54747
C	6.62477	-2.93046	-3.22844
C	7.81609	-2.62864	-2.57237
C	7.80179	-2.28631	-1.22140
C	6.59044	-2.25319	-0.53235
C	5.40265	-2.55498	-1.19169
C	3.59667	-5.33485	-2.34792
C	4.29141	-5.92430	-3.54628
C	3.56847	-6.28856	-4.68644
C	4.21647	-6.82275	-5.79625
C	5.59867	-7.00913	-5.77566
C	6.32522	-6.66409	-4.63813
C	5.67345	-6.12479	-3.53080
N	1.58288	-3.95877	-0.72891
C	1.72412	-5.25552	-0.18227
C	2.65494	-5.51769	0.84477
C	2.71220	-6.79784	1.39979
C	1.86799	-7.81338	0.97036
C	0.93254	-7.54113	-0.02014
C	0.83875	-6.27535	-0.59939
C	3.55594	-4.43709	1.40222
C	3.10853	-4.05558	2.81784
C	-0.22136	-6.02169	-1.65008
C	-1.62052	-6.11904	-1.03049
C	5.03530	-4.82753	1.38644
C	-0.08831	-6.95574	-2.85598
C	-0.40192	-3.33672	1.79049
C	-1.16957	-1.67753	1.95858
Al	0.42719	-0.87185	2.90691
C	0.00966	1.10686	3.27402
C	2.05191	-0.88188	1.64037

O	-0.71543	-4.15279	2.59761
C	4.92826	0.53876	-2.43218
C	0.93258	-3.79694	-5.14337
C	0.81770	-1.66885	4.65613
C	-3.66996	-0.35244	0.15565
C	-0.31411	0.29449	-0.78993
C	-5.73523	4.56569	-0.20019
C	-3.69456	2.19198	5.88410
C	1.59868	5.59185	2.37608
C	-2.83809	4.24275	-2.42852
H	0.34426	-0.09473	-1.57544
H	-1.26046	0.44528	-1.33671
H	0.09343	1.30721	-0.65333
H	-1.72172	-1.95142	2.86729
H	-4.30966	0.08950	0.93197
H	-3.36770	0.46252	-0.52365
H	-4.36583	-0.93279	-0.46576
H	-2.75698	-2.94552	-1.64824
H	-1.41522	-1.86752	-1.81100
H	-1.20380	-3.54755	-1.24930
H	-4.59437	-2.92292	2.09484
H	-3.44989	-4.22907	1.78663
H	-4.62113	-3.76632	0.54501
H	2.74139	-0.38916	2.33760
H	2.08092	-0.23506	0.75047
H	2.57557	-1.81963	1.39880
H	-0.76181	1.16053	4.05231
H	0.95599	1.29541	3.79549
H	-0.08916	2.00164	2.64299
H	1.83823	-1.45602	4.99878
H	0.69796	-2.75691	4.63873
H	0.13440	-1.28459	5.42473
H	-1.58767	5.57403	-0.07375
H	-0.98374	5.92647	1.53817

H	-1.44507	7.97223	2.53955
H	-2.57298	10.17845	2.50770
H	-4.38345	10.62278	0.86457
H	-5.02153	8.86390	-0.77029
H	-3.87065	6.67556	-0.74584
H	-4.15628	4.34655	2.95201
H	-4.01681	6.02738	2.49084
H	-1.37133	3.57553	3.57933
H	0.13279	3.96829	5.48621
H	-0.15817	5.99710	6.90464
H	-1.96684	7.61469	6.37218
H	-3.44911	7.22393	4.42556
H	2.69949	5.25960	-0.14946
H	2.28137	5.31022	-2.58641
H	0.21885	4.28485	-3.49242
H	0.30387	3.90309	2.34218
H	0.85799	6.36200	2.14445
H	2.56056	5.92896	1.97606
H	1.69036	5.53598	3.46500
H	2.52471	3.13454	3.15846
H	3.24165	3.50999	1.58161
H	2.05463	2.21303	1.71613
H	-2.25364	2.45824	-1.41969
H	-3.09569	4.75897	-1.50081
H	-3.76225	3.83986	-2.85573
H	-2.43246	4.98248	-3.12710
H	-0.77444	1.58196	-3.30994
H	-1.30567	2.95287	-4.29032
H	-2.48916	1.77601	-3.72844
H	-7.57591	2.66851	1.13983
H	-8.05800	1.83472	3.41522
H	-6.18056	1.31798	4.95047
H	-4.15903	3.11681	-0.25548
H	-5.23664	5.23396	0.50414

H	-6.81312	4.61998	-0.01350
H	-5.55548	4.93712	-1.21465
H	-5.66214	2.55524	-2.10029
H	-6.99662	2.24190	-0.98856
H	-5.58123	1.18291	-0.97906
H	-2.62002	1.96546	4.06812
H	-3.80732	3.27599	5.79436
H	-2.81312	1.99559	6.50342
H	-4.56654	1.79866	6.41730
H	-3.22227	-0.43421	3.62924
H	-4.22787	-0.43849	5.07051
H	-2.47259	-0.23238	5.22047
H	2.72582	-5.93822	-2.10537
H	4.26829	-5.38123	-1.48367
H	6.24090	-5.84081	-2.64939
H	7.40097	-6.81213	-4.61178
H	6.10503	-7.42757	-6.64067
H	3.64202	-7.09965	-6.67556
H	2.49311	-6.14543	-4.70361
H	3.65426	-2.17017	-3.53933
H	4.33298	-3.61633	-4.24657
H	6.63622	-3.21010	-4.27875
H	8.75626	-2.66599	-3.11527
H	8.72910	-2.05080	-0.70747
H	6.56889	-1.98914	0.52081
H	4.46091	-2.51573	-0.65558
H	3.42841	-6.99807	2.19197
H	1.92887	-8.80320	1.41299
H	0.25508	-8.32473	-0.34882
H	3.43706	-3.55877	0.75980
H	5.38748	-5.04664	0.37479
H	5.21988	-5.70996	2.00733
H	5.65018	-4.01297	1.78199
H	3.68377	-3.20416	3.19794

H	3.25259	-4.89497	3.50615
H	2.04834	-3.79686	2.83959
H	-0.07351	-5.00060	-2.01356
H	0.90829	-6.89322	-3.30245
H	-0.82386	-6.69671	-3.62533
H	-0.25721	-8.00055	-2.57477
H	-1.71127	-5.47417	-0.15235
H	-1.82842	-7.14410	-0.70565
H	-2.39096	-5.83276	-1.75494
H	0.18500	-0.81087	-5.94127
H	1.34803	1.36869	-5.85576
H	2.84833	1.87892	-3.94996
H	0.23252	-3.29470	-3.18698
H	1.96917	-3.95859	-4.84601
H	0.92872	-3.39113	-6.16112
H	0.42411	-4.76739	-5.16346
H	-1.73455	-1.88792	-4.00252
H	-1.78122	-3.59690	-4.46812
H	-1.32044	-2.37216	-5.65144
H	3.54345	-0.52450	-1.18627
H	5.24768	-0.30699	-3.04431
H	5.65477	0.65278	-1.62138
H	4.97077	1.44071	-3.05272
H	2.11162	1.46439	-0.62287
H	3.07469	2.46468	-1.68976
H	3.81722	1.75656	-0.25032

216

Int2

C	3.16697	0.24593	-2.39872
C	2.28837	-0.83375	-2.66848
C	1.54943	-0.84583	-3.87523
C	1.72473	0.20016	-4.78578
C	2.59617	1.25042	-4.53327
C	3.30564	1.26750	-3.33919

N	2.11334	-1.80076	-1.65097
C	2.45900	-3.09828	-1.64751
N	3.21708	-3.72506	-2.60251
C	4.10040	-3.02869	-3.51515
C	5.55357	-3.42478	-3.37211
C	6.46957	-2.95934	-4.32122
C	7.82078	-3.27273	-4.21609
C	8.27429	-4.06980	-3.16432
C	7.36631	-4.54107	-2.22049
C	6.01354	-4.21591	-2.31919
C	0.58717	-1.95721	-4.23688
C	-0.86498	-1.46822	-4.30132
C	4.01801	0.28007	-1.14598
C	3.96956	1.63925	-0.44520
Y	0.57511	-2.03840	0.14641
C	1.46476	0.35248	1.51321
Al	0.44912	-0.50464	3.09628
C	1.57037	-1.16607	4.54648
Y	-1.48883	0.56046	1.25179
C	-0.75156	1.02117	3.75414
Al	-2.38332	-2.41641	0.94906
C	-3.47071	-3.81542	1.78252
N	-1.11724	2.89533	0.84874
C	-0.01238	3.63889	0.37577
C	0.95644	4.18029	1.25336
C	2.12245	4.72979	0.71349
C	2.33876	4.77588	-0.65738
C	1.36321	4.28527	-1.51669
C	0.18320	3.72177	-1.02838
C	0.74564	4.25070	2.75318
C	1.71847	3.35023	3.51869
C	-0.89969	3.30494	-2.00648
C	-0.36487	2.52613	-3.20864
N	-3.14577	2.18220	1.41901

C	-4.54966	2.08416	1.37657
C	-5.28807	2.36422	0.20003
C	-6.64742	2.04271	0.17365
C	-7.28790	1.46263	1.26263
C	-6.56325	1.21467	2.42081
C	-5.20479	1.52237	2.49916
C	-4.65137	2.94871	-1.04996
C	-4.26147	1.85977	-2.05652
C	-4.45594	1.25936	3.78913
C	-4.32002	-0.23804	4.08308
C	-0.55323	0.04896	-0.89371
C	-0.92205	-1.77562	2.24990
C	-3.60864	-0.89069	0.39338
N	1.94049	-3.77694	-0.59636
C	2.66935	-4.78899	0.07258
C	3.95308	-4.50289	0.61101
C	4.67137	-5.52751	1.22754
C	4.13143	-6.80172	1.37847
C	2.84181	-7.04811	0.93190
C	2.09699	-6.06239	0.27810
C	4.46213	-3.07222	0.66379
C	3.68883	-2.32287	1.75505
C	0.69006	-6.38030	-0.17677
C	-0.24840	-6.53936	1.02423
C	-1.53433	-3.18641	-0.76521
C	-2.36540	3.25988	1.16831
N	-2.84134	4.54656	1.28859
C	-3.84588	4.84392	2.32269
C	-3.19535	5.26618	3.61818
C	-2.48413	4.33497	4.38082
C	-1.84402	4.71557	5.55550
C	-1.90628	6.04219	5.98404
C	-2.61131	6.97837	5.23062
C	-3.25181	6.59126	4.05382

C	-1.99253	5.69708	0.99266
C	-2.77903	6.90386	0.55336
C	-2.43235	8.16973	1.02885
C	-3.12107	9.30158	0.59625
C	-4.17215	9.17646	-0.31031
C	-4.52821	7.91382	-0.78345
C	-3.83331	6.78714	-0.35453
C	3.03624	-5.15037	-2.89205
C	1.90974	-5.44782	-3.85120
C	2.17113	-6.01024	-5.10152
C	1.12779	-6.35204	-5.96235
C	-0.19298	-6.13543	-5.57767
C	-0.46459	-5.56279	-4.33375
C	0.57929	-5.22050	-3.48218
C	0.87542	5.68526	3.28337
C	-1.68109	4.53310	-2.49253
C	-5.55935	3.97468	-1.73843
C	-5.08561	1.99763	4.97394
C	5.95842	-2.92542	0.93231
C	0.62668	-7.60597	-1.09211
C	0.96766	-2.61306	-5.56740
C	5.46531	-0.11861	-1.46130
H	-1.54346	0.21606	-1.34806
H	-0.06947	1.03770	-0.84139
H	0.00773	-0.42874	-1.70404
H	-1.56962	-1.91988	3.13469
H	-4.42396	-0.76321	1.11302
H	-3.48221	0.09321	-0.07387
H	-4.03934	-1.47011	-0.43756
H	-2.50192	-3.48842	-1.18666
H	-1.08102	-2.62479	-1.60004
H	-0.98645	-4.12899	-0.63848
H	-4.23742	-4.22277	1.11128
H	-3.98835	-3.44544	2.67646

H	-2.83484	-4.64796	2.10464
H	2.33912	0.61405	2.12570
H	1.11108	1.32360	1.14141
H	1.94212	-0.10001	0.63221
H	-1.63718	0.63137	4.26951
H	-0.07416	1.36696	4.54383
H	-1.02937	1.95816	3.25232
H	2.27180	-0.40129	4.90410
H	2.14638	-2.04786	4.25348
H	0.94803	-1.46845	5.39767
H	-1.29130	5.42390	0.20680
H	-1.39903	5.96035	1.87284
H	-1.62447	8.26545	1.74992
H	-2.84163	10.28080	0.97436
H	-4.71399	10.05709	-0.64246
H	-5.34918	7.80510	-1.48653
H	-4.11482	5.80208	-0.70663
H	-4.47486	3.96945	2.47089
H	-4.48866	5.64309	1.94617
H	-2.43650	3.30303	4.05164
H	-1.29872	3.97647	6.13542
H	-1.40805	6.34313	6.90099
H	-2.66210	8.01304	5.55743
H	-3.79184	7.32369	3.45989
H	2.87171	5.13712	1.38662
H	3.25687	5.19922	-1.05369
H	1.51779	4.33646	-2.59002
H	-0.27365	3.91200	2.95895
H	0.28495	6.39748	2.70238
H	1.91630	6.02428	3.25327
H	0.53342	5.73443	4.32088
H	1.52813	3.40718	4.59582
H	2.75363	3.66275	3.34366
H	1.63003	2.30985	3.21038

H	-1.59564	2.65890	-1.46448
H	-2.16303	5.07177	-1.67457
H	-2.46102	4.23553	-3.20146
H	-1.01397	5.23637	-3.00244
H	0.29349	1.71013	-2.91119
H	0.19716	3.17238	-3.89073
H	-1.19642	2.09938	-3.77910
H	-7.21722	2.23873	-0.72938
H	-8.34340	1.21319	1.20841
H	-7.05726	0.77420	3.28273
H	-3.73902	3.47064	-0.75471
H	-5.97111	4.69301	-1.02357
H	-6.40099	3.49310	-2.24670
H	-4.99748	4.52353	-2.50125
H	-3.86881	2.30750	-2.97530
H	-5.12884	1.24419	-2.31700
H	-3.48990	1.19594	-1.66212
H	-3.45031	1.66222	3.64978
H	-5.16707	3.06994	4.77480
H	-4.47343	1.86881	5.87278
H	-6.08844	1.61814	5.19649
H	-3.78997	-0.76452	3.28346
H	-5.30182	-0.71255	4.18378
H	-3.77007	-0.40493	5.01567
H	2.88591	-5.69199	-1.95797
H	3.97765	-5.51368	-3.31112
H	3.20131	-6.19259	-5.39871
H	1.34837	-6.79110	-6.93103
H	-1.00707	-6.40860	-6.24270
H	-1.49075	-5.38701	-4.02373
H	0.36880	-4.78357	-2.51308
H	4.00759	-1.95550	-3.35817
H	3.77722	-3.21030	-4.54861
H	6.11792	-2.34241	-5.14550

H	8.52101	-2.89925	-4.95793
H	9.32765	-4.32202	-3.08500
H	7.70562	-5.16584	-1.39894
H	5.31440	-4.57689	-1.57117
H	5.66028	-5.32089	1.62290
H	4.70461	-7.58460	1.86662
H	2.39728	-8.02774	1.08371
H	4.26040	-2.58430	-0.29459
H	6.56476	-3.44150	0.18524
H	6.22826	-3.30372	1.92383
H	6.23156	-1.86677	0.90645
H	3.88625	-1.24723	1.72420
H	3.97771	-2.69484	2.74307
H	2.60650	-2.50293	1.69738
H	0.35296	-5.51530	-0.75125
H	1.29303	-7.50817	-1.95337
H	-0.39094	-7.74321	-1.47270
H	0.90294	-8.51816	-0.55239
H	-0.18684	-5.67935	1.69454
H	0.01591	-7.43102	1.60419
H	-1.28514	-6.65626	0.68770
H	1.15556	0.18990	-5.71094
H	2.71355	2.05294	-5.25557
H	3.98656	2.08714	-3.12991
H	0.64912	-2.72306	-3.46049
H	1.99147	-2.99042	-5.55097
H	0.88568	-1.90026	-6.39453
H	0.30754	-3.45635	-5.78064
H	-1.18250	-0.99625	-3.36896
H	-1.53549	-2.30914	-4.50863
H	-0.99350	-0.73110	-5.10138
H	3.61477	-0.47375	-0.46452
H	5.53790	-1.14132	-1.84089
H	6.08644	-0.05315	-0.56211

H	5.89511	0.54954	-2.21558
H	2.94443	1.98621	-0.30220
H	4.49805	2.40798	-1.01842
H	4.45212	1.57828	0.53611
C	0.07007	-3.00626	2.22800
O	0.05561	-3.85780	3.10266

216

TS2

C	4.86601	7.22560	0.92021
C	3.98891	6.12107	0.76625
C	3.22318	5.99888	-0.41672
C	3.34697	6.97857	-1.40626
C	4.21429	8.05287	-1.26662
C	4.97112	8.16383	-0.10694
N	3.83759	5.24777	1.86930
C	4.24618	3.97920	2.01506
N	5.02150	3.27258	1.13081
C	5.83189	3.89045	0.10126
C	7.29960	3.53446	0.18544
C	8.13917	3.92555	-0.86283
C	9.50130	3.64677	-0.82294
C	10.04257	2.95820	0.26331
C	9.21106	2.56086	1.30627
C	7.84749	2.85237	1.27194
C	2.29558	4.83136	-0.67585
C	0.82924	5.26643	-0.77199
C	5.74028	7.38100	2.14754
C	5.66453	8.79068	2.74173
Y	2.28912	5.10376	3.68584
C	3.10544	7.53438	4.65435
Al	2.54801	6.59394	6.42218
C	4.00226	6.51880	7.73550
Y	0.32391	7.65469	4.74828
C	1.10234	7.74830	7.31620

Al	-0.68332	4.62791	4.23501
C	-1.78424	3.18104	4.97854
N	0.76080	9.99419	4.53709
C	1.88778	10.79497	4.23070
C	2.78709	11.24212	5.22471
C	3.94513	11.91875	4.82972
C	4.22733	12.16843	3.49399
C	3.33404	11.73613	2.52188
C	2.16904	11.04851	2.86323
C	2.55247	11.02782	6.70681
C	3.65717	10.16010	7.31883
C	1.21107	10.64360	1.76045
C	1.90739	9.84714	0.65400
N	-1.30220	9.28518	5.00796
C	-2.70582	9.24633	4.88087
C	-3.36091	9.63526	3.68560
C	-4.73270	9.39810	3.57109
C	-5.46077	8.78908	4.58733
C	-4.81404	8.42321	5.75987
C	-3.44781	8.65043	5.92831
C	-2.61213	10.22822	2.50318
C	-2.10020	9.13436	1.55936
C	-2.78200	8.26052	7.23122
C	-2.66700	6.74127	7.38220
C	0.99861	6.99964	2.50064
C	0.68291	5.28287	5.60711
C	-1.90319	6.17154	3.75394
N	3.77179	3.40317	3.14763
C	4.52858	2.47684	3.89956
C	5.84331	2.79806	4.33386
C	6.56821	1.83810	5.04046
C	6.01103	0.60756	5.37843
C	4.69624	0.33920	5.02844
C	3.94130	1.25500	4.29067

C	6.39108	4.20780	4.17921
C	5.67612	5.12297	5.17742
C	2.50685	0.92559	3.94146
C	1.62178	0.93156	5.19223
C	0.26108	3.82785	2.58991
C	-0.48714	10.35915	4.86008
N	-0.94033	11.64126	5.06151
C	-1.96046	11.88650	6.08945
C	-1.35130	12.16119	7.44277
C	-0.71398	11.13605	8.14889
C	-0.16809	11.36977	9.40598
C	-0.23938	12.64367	9.97074
C	-0.85560	13.67657	9.26823
C	-1.41083	13.43464	8.01152
C	-0.10736	12.80965	4.78334
C	-0.92217	14.02441	4.42608
C	-0.66727	15.24798	5.04676
C	-1.39254	16.38534	4.69370
C	-2.38629	16.30596	3.72044
C	-2.64822	15.08481	3.09865
C	-1.91804	13.95394	3.44925
C	4.86496	1.82042	0.99130
C	3.74928	1.41493	0.06065
C	4.02356	0.78980	-1.15678
C	2.98669	0.36191	-1.98633
C	1.66150	0.55718	-1.60427
C	1.37731	1.19283	-0.39419
C	2.41487	1.61705	0.42784
C	2.46608	12.34773	7.48262
C	0.49679	11.86468	1.16722
C	-3.45368	11.23153	1.70743
C	-3.46861	8.89022	8.44555
C	7.89860	4.33876	4.39098
C	2.37609	-0.39580	3.17950

C	2.69016	4.07603	-1.94842
C	7.19410	7.00441	1.83581
H	0.08703	6.66404	1.99491
H	0.98078	8.10285	2.44284
H	1.82020	6.78113	1.80446
H	-0.07014	5.19618	6.41286
H	-2.80125	6.07439	4.37408
H	-1.79609	7.25475	3.61801
H	-2.19309	5.84623	2.74302
H	-0.66176	3.82147	1.99191
H	0.99459	4.17903	1.84705
H	0.48389	2.77470	2.79706
H	-2.46437	2.74254	4.23624
H	-2.40587	3.53930	5.80890
H	-1.16152	2.37010	5.37260
H	3.98624	7.90031	5.19972
H	2.65483	8.46496	4.28632
H	3.57164	7.10391	3.75656
H	0.91769	7.02262	8.12267
H	1.61185	8.60052	7.77178
H	0.08881	8.11010	7.08214
H	4.92797	6.99463	7.39239
H	4.22235	5.47067	7.96057
H	3.71175	7.00464	8.67469
H	0.55653	12.57270	3.95527
H	0.53097	13.04338	5.64030
H	0.09922	15.30558	5.81539
H	-1.18516	17.33191	5.18450
H	-2.95593	17.18986	3.44903
H	-3.42183	15.01322	2.33936
H	-2.12656	12.99995	2.97990
H	-2.62880	11.02995	6.13834
H	-2.56059	12.74019	5.76671
H	-0.64992	10.14706	7.70952

H	0.31490	10.55826	9.94280
H	0.18697	12.82966	10.95214
H	-0.90924	14.67207	9.69962
H	-1.89530	14.23875	7.46385
H	4.63935	12.25913	5.59306
H	5.13439	12.69524	3.21303
H	3.54287	11.93160	1.47404
H	1.59771	10.50709	6.81833
H	1.59587	12.94306	7.19797
H	3.36194	12.95752	7.32718
H	2.37560	12.14868	8.55339
H	3.44653	9.94774	8.37213
H	4.62612	10.66800	7.27184
H	3.76032	9.20631	6.80126
H	0.44629	10.00775	2.21393
H	-0.07514	12.41616	1.91800
H	-0.19698	11.55586	0.37805
H	1.21739	12.56115	0.72540
H	2.50640	9.02658	1.05273
H	2.57829	10.48177	0.06623
H	1.17137	9.42142	-0.03517
H	-5.24301	9.68238	2.65653
H	-6.52417	8.60632	4.46439
H	-5.37636	7.95536	6.56337
H	-1.74346	10.76797	2.88836
H	-3.95733	11.94896	2.36203
H	-4.22155	10.72999	1.10937
H	-2.81663	11.78423	1.00943
H	-1.60232	9.57552	0.68967
H	-2.92942	8.51357	1.20433
H	-1.38051	8.47628	2.04752
H	-1.76811	8.66412	7.18966
H	-3.52836	9.97778	8.34581
H	-2.90605	8.66689	9.35796

H	-4.48516	8.50578	8.57942
H	-2.12861	6.29271	6.54217
H	-3.65602	6.27173	7.41293
H	-2.13629	6.47832	8.30319
H	4.71402	1.37833	1.97591
H	5.81380	1.42968	0.61650
H	5.05775	0.62620	-1.45098
H	3.21544	-0.12651	-2.92914
H	0.85332	0.21819	-2.24582
H	0.34803	1.35305	-0.08622
H	2.19778	2.09905	1.37354
H	5.72644	4.97207	0.16102
H	5.45136	3.60492	-0.88817
H	7.71850	4.45694	-1.71393
H	10.14132	3.96210	-1.64211
H	11.10442	2.73228	0.29281
H	9.62020	2.02052	2.15541
H	7.20747	2.54998	2.09500
H	7.57921	2.06578	5.36091
H	6.59265	-0.12113	5.93551
H	4.24046	-0.60163	5.32443
H	6.17594	4.56690	3.16792
H	8.47207	3.68893	3.72740
H	8.17731	4.11284	5.42559
H	8.20698	5.36856	4.19095
H	5.93172	6.17461	5.01165
H	5.95764	4.85920	6.20059
H	4.58705	5.00968	5.13629
H	2.15965	1.72696	3.28514
H	3.00365	-0.41107	2.28421
H	1.33908	-0.55265	2.86386
H	2.66194	-1.24623	3.80775
H	1.69745	1.87559	5.73744
H	1.91814	0.13199	5.88038

H	0.57376	0.76586	4.91944
H	2.75186	6.88687	-2.31059
H	4.29775	8.79793	-2.05237
H	5.65349	9.00044	0.01172
H	2.38962	4.13463	0.16066
H	3.72412	3.72969	-1.90878
H	2.58342	4.71314	-2.83261
H	2.05309	3.19929	-2.08265
H	0.51232	5.82324	0.11138
H	0.18288	4.38906	-0.88127
H	0.67038	5.91030	-1.64396
H	5.37308	6.67125	2.89313
H	7.29236	5.96172	1.52189
H	7.82433	7.14680	2.71972
H	7.59459	7.63383	1.03362
H	4.63348	9.12185	2.88392
H	6.15806	9.52640	2.09802
H	6.16934	8.82202	3.71320
C	1.91584	4.60928	6.02572
O	2.05745	3.92876	7.05822

216

Int3

C	2.98532	0.03356	-2.79211
C	2.06109	-1.02943	-2.95594
C	1.29625	-1.12135	-4.13917
C	1.50679	-0.18172	-5.15255
C	2.44805	0.83001	-5.02164
C	3.17674	0.93143	-3.84251
N	1.85576	-1.90316	-1.86494
C	2.31307	-3.14893	-1.67707
N	3.15961	-3.83890	-2.50868
C	3.84527	-3.22013	-3.63487
C	5.25280	-3.73057	-3.80860
C	5.78598	-3.87676	-5.09044

C	7.10613	-4.28824	-5.26485
C	7.90349	-4.56739	-4.15641
C	7.37282	-4.43363	-2.87381
C	6.05604	-4.01751	-2.70270
C	0.23549	-2.18378	-4.34691
C	-1.15862	-1.54540	-4.41329
C	3.79814	0.18325	-1.52129
C	3.86305	1.63148	-1.02911
Y	0.10366	-2.16092	-0.28716
C	1.67917	1.02920	1.51290
Al	1.24970	0.17136	3.32430
C	2.73536	0.42016	4.57677
Y	-0.95669	0.74168	1.39300
C	-0.58989	0.88964	3.94645
Al	-2.74042	-2.66045	0.64354
C	-3.80470	-4.08179	1.50051
N	-0.84162	3.07155	1.07675
C	0.23236	3.95506	0.81868
C	0.98954	4.54966	1.85362
C	2.17162	5.21680	1.52195
C	2.59383	5.33971	0.20377
C	1.80224	4.82086	-0.81405
C	0.62159	4.13216	-0.53177
C	0.53644	4.53659	3.29961
C	1.42801	3.66421	4.18235
C	-0.26100	3.66581	-1.67378
C	0.49439	2.83708	-2.71252
N	-2.83608	2.14173	1.42235
C	-4.23290	1.98569	1.27673
C	-4.90714	2.33975	0.08036
C	-6.26240	2.02602	-0.03915
C	-6.96033	1.37662	0.97228
C	-6.29604	1.04111	2.14306
C	-4.94446	1.34105	2.31528

C	-4.19929	2.96956	-1.10714
C	-3.69795	1.89466	-2.07641
C	-4.26300	0.95077	3.60806
C	-4.05881	-0.56507	3.68360
C	-0.71676	0.07216	-0.97777
C	-1.21451	-2.18616	1.93162
C	-3.81343	-1.09829	0.04261
N	1.79458	-3.71821	-0.55881
C	2.46088	-4.66408	0.25000
C	3.74178	-4.39605	0.79651
C	4.33126	-5.35367	1.62334
C	3.68281	-6.54073	1.94816
C	2.41457	-6.78018	1.44001
C	1.79476	-5.86349	0.58893
C	4.41647	-3.05124	0.60208
C	3.76979	-2.01414	1.52476
C	0.41262	-6.15730	0.05389
C	-0.64931	-5.92831	1.13167
C	-1.92167	-3.39266	-1.16720
C	-2.14243	3.30110	1.31615
N	-2.73491	4.53321	1.46541
C	-3.85332	4.70619	2.40372
C	-3.37780	5.08503	3.78573
C	-2.71116	4.14448	4.57672
C	-2.25388	4.47593	5.84769
C	-2.45862	5.76295	6.34713
C	-3.12146	6.70826	5.56725
C	-3.57846	6.36957	4.29375
C	-1.98601	5.76946	1.24754
C	-2.85374	6.90486	0.77423
C	-2.80021	8.14513	1.41213
C	-3.57424	9.21051	0.95387
C	-4.41496	9.04170	-0.14437
C	-4.47157	7.80483	-0.78719

C	-3.69153	6.74729	-0.33092
C	3.06623	-5.30093	-2.60797
C	2.18572	-5.74850	-3.74753
C	2.74289	-6.15393	-4.96293
C	1.92704	-6.58674	-6.00782
C	0.54447	-6.63082	-5.84198
C	-0.02073	-6.22215	-4.63400
C	0.79537	-5.77383	-3.60054
C	0.48069	5.94855	3.89991
C	-0.95579	4.85794	-2.34455
C	-5.08194	3.96547	-1.86717
C	-4.99975	1.47365	4.84337
C	5.92822	-3.07450	0.83568
C	0.28926	-7.55126	-0.56389
C	0.48878	-3.01658	-5.60605
C	5.21913	-0.36365	-1.70639
H	-1.55027	-0.46644	-1.45658
H	-0.99589	1.12627	-1.11163
H	0.15204	-0.00949	-1.64959
H	-1.59665	-2.56903	2.89242
H	-4.77770	-1.04786	0.56096
H	-3.37824	-0.09921	0.15739
H	-4.03199	-1.18789	-1.03055
H	-2.90468	-3.84972	-1.34172
H	-1.77539	-2.76924	-2.06251
H	-1.23196	-4.24729	-1.23561
H	-4.47265	-4.59212	0.79354
H	-4.45180	-3.63902	2.26994
H	-3.20423	-4.85231	1.99579
H	2.04695	2.04535	1.68787
H	1.22451	1.06598	0.50813
H	2.56191	0.40588	1.32565
H	-0.30940	0.90293	5.00727
H	-0.92813	1.91611	3.74681

H	-1.45806	0.21272	3.92511
H	3.53509	1.04148	4.15680
H	3.17409	-0.55579	4.81809
H	2.43338	0.88234	5.52393
H	-1.21261	5.58207	0.50639
H	-1.48211	6.06885	2.16974
H	-2.15436	8.27194	2.27719
H	-3.52465	10.17072	1.45934
H	-5.02313	9.86909	-0.49797
H	-5.12215	7.66377	-1.64565
H	-3.73439	5.78289	-0.82081
H	-4.43574	3.78894	2.44005
H	-4.50547	5.48504	2.00104
H	-2.56030	3.14164	4.19271
H	-1.74198	3.72930	6.44791
H	-2.10456	6.02551	7.33975
H	-3.28454	7.71164	5.95016
H	-4.09403	7.10688	3.68448
H	2.76657	5.65731	2.31726
H	3.51945	5.85747	-0.02986
H	2.10378	4.95074	-1.84961
H	-0.47527	4.12197	3.32323
H	-0.03449	6.66082	3.25102
H	1.48858	6.34011	4.07394
H	-0.04285	5.92635	4.85940
H	1.03575	3.61882	5.20359
H	2.44591	4.06593	4.22750
H	1.49961	2.64690	3.80287
H	-1.04535	3.04034	-1.23852
H	-1.54122	5.44659	-1.63339
H	-1.63336	4.51165	-3.13265
H	-0.22214	5.53022	-2.80234
H	1.03182	2.00337	-2.25893
H	1.23026	3.44149	-3.25238

H	-0.19535	2.42230	-3.45414
H	-6.78310	2.28181	-0.95619
H	-8.01172	1.13614	0.84563
H	-6.83172	0.53503	2.94134
H	-3.32950	3.51768	-0.73617
H	-5.57328	4.67327	-1.19373
H	-5.86137	3.45377	-2.44081
H	-4.47909	4.52871	-2.58763
H	-3.12906	2.34559	-2.89724
H	-4.54257	1.34363	-2.50361
H	-3.05915	1.17116	-1.57429
H	-3.27521	1.41690	3.59547
H	-5.14838	2.55583	4.78808
H	-4.42185	1.25816	5.74836
H	-5.98131	1.00235	4.95886
H	-3.47995	-0.93202	2.83038
H	-5.01856	-1.09184	3.66578
H	-3.53102	-0.84927	4.60052
H	2.69985	-5.69870	-1.66606
H	4.07782	-5.69068	-2.74525
H	3.82330	-6.13354	-5.08413
H	2.37288	-6.89976	-6.94764
H	-0.09090	-6.98014	-6.65060
H	-1.09805	-6.25128	-4.49705
H	0.35549	-5.45140	-2.66312
H	3.88233	-2.14501	-3.47550
H	3.28245	-3.37539	-4.56320
H	5.16095	-3.66671	-5.95528
H	7.50961	-4.39783	-6.26745
H	8.93043	-4.89390	-4.29136
H	7.98465	-4.65584	-2.00409
H	5.62966	-3.93167	-1.70941
H	5.31193	-5.15833	2.04368
H	4.16107	-7.26460	2.60140

H	1.89363	-7.69766	1.69893
H	4.25009	-2.72366	-0.42829
H	6.42039	-3.89049	0.29781
H	6.16614	-3.18584	1.89875
H	6.37156	-2.13255	0.50170
H	4.23405	-1.03166	1.38534
H	3.88955	-2.30347	2.57318
H	2.69687	-1.92509	1.34275
H	0.23782	-5.42801	-0.74227
H	1.04758	-7.71544	-1.33519
H	-0.69576	-7.67449	-1.02668
H	0.39745	-8.33828	0.18953
H	-0.59309	-4.91501	1.54326
H	-0.51116	-6.62595	1.96492
H	-1.65560	-6.07377	0.72721
H	0.91975	-0.24934	-6.06430
H	2.60331	1.54262	-5.82636
H	3.90023	1.73275	-3.72720
H	0.26369	-2.86685	-3.48986
H	1.42489	-3.57612	-5.54327
H	0.52890	-2.38177	-6.49704
H	-0.31385	-3.74372	-5.74865
H	-1.35262	-0.90606	-3.54810
H	-1.93637	-2.31503	-4.46295
H	-1.25297	-0.91843	-5.30641
H	3.29932	-0.41860	-0.75551
H	5.23071	-1.42461	-1.96654
H	5.79803	-0.24105	-0.78512
H	5.73957	0.17721	-2.50450
H	2.87299	2.07930	-0.92655
H	4.44820	2.26297	-1.70629
H	4.34744	1.67362	-0.04867
C	0.04534	-1.64468	1.97103
O	0.79689	-1.54069	3.07430

216
TS3

C	0.67654	-5.32988	0.34424
C	1.86680	-4.56073	0.35822
C	2.94136	-4.93260	1.19662
C	2.82754	-6.10236	1.95263
C	1.68501	-6.88800	1.90980
C	0.61551	-6.49047	1.11579
N	1.87194	-3.36474	-0.39336
C	2.66286	-2.97886	-1.40910
N	3.46559	-3.80896	-2.14505
C	3.45701	-5.26094	-1.96938
C	3.63256	-6.00506	-3.26740
C	2.79160	-5.75329	-4.35418
C	2.94253	-6.45544	-5.54523
C	3.93569	-7.42852	-5.66223
C	4.77237	-7.69318	-4.58050
C	4.62201	-6.98153	-3.39069
C	4.17744	-4.07403	1.38232
C	5.48246	-4.85568	1.20917
C	-0.50508	-4.92599	-0.51709
C	-0.33259	-5.44135	-1.95031
Y	0.97409	-1.27776	0.04514
C	-1.28563	-1.66019	0.64242
C	-2.59808	-1.70837	0.82606
Al	-3.91566	-1.73763	-0.71091
C	-3.43432	0.13099	-1.63398
Y	-1.78902	0.76476	0.18052
C	0.24946	0.78373	-1.25522
N	-2.96729	2.19951	1.52814
C	-4.22793	1.90124	2.09234
C	-5.44190	2.17313	1.41075
C	-6.64709	1.75300	1.97704
C	-6.68507	1.03897	3.16697

C	-5.49296	0.74878	3.81621
C	-4.26236	1.17146	3.31026
C	-5.49770	2.88798	0.07562
C	-6.11913	4.27892	0.22421
C	-3.01665	0.90770	4.13430
C	-3.07705	1.69560	5.44980
C	-2.49598	3.35872	0.99767
N	-1.62680	3.11004	0.00667
C	-0.67834	3.91088	-0.66104
C	0.46085	4.43361	-0.00988
C	1.44086	5.06961	-0.77429
C	1.31778	5.19186	-2.15314
C	0.19711	4.66928	-2.78869
C	-0.80484	4.02188	-2.06599
C	0.63721	4.27393	1.48370
C	1.40479	5.41943	2.14146
C	-2.02105	3.44442	-2.75884
C	-3.22694	4.37903	-2.61776
N	-2.87658	4.59798	1.44152
C	-2.28942	5.82094	0.87499
C	-3.20746	7.01344	0.93002
C	-2.97435	8.03893	1.84976
C	-3.81386	9.14976	1.90463
C	-4.89487	9.24942	1.03123
C	-5.12075	8.24258	0.09316
C	-4.27757	7.13654	0.04018
C	-3.44697	4.75808	2.78084
C	-2.40660	5.02040	3.84450
C	-2.46333	6.16972	4.63433
C	-1.51208	6.39700	5.62880
C	-0.49466	5.47089	5.84626
C	-0.43771	4.31206	5.07132
C	-1.38727	4.09231	4.07912
C	-6.27267	2.07924	-0.97252

C	-2.78963	-0.57515	4.44118
C	1.28598	2.93103	1.81892
C	-1.78476	3.05644	-4.21617
N	2.56463	-1.64623	-1.63645
C	2.74338	-0.95392	-2.84955
C	3.57151	0.19425	-2.84876
C	3.65156	0.97925	-4.00094
C	2.93892	0.65316	-5.14623
C	2.11440	-0.46765	-5.13816
C	1.98823	-1.27387	-4.00633
C	4.35494	0.60627	-1.61868
C	5.85053	0.75696	-1.91566
C	0.97070	-2.39767	-3.98803
C	0.72593	-3.02900	-5.36005
O	-0.55799	-0.43974	1.60696
Al	0.64850	-0.76076	2.97825
C	0.61050	0.58920	4.41355
C	0.46672	-2.68496	3.37107
C	2.45299	-0.46752	1.90234
C	3.79806	1.89920	-1.01190
C	-0.35995	-1.91181	-3.40580
C	4.61461	-3.29212	-2.89175
C	5.91544	-3.41712	-2.13920
C	6.86277	-4.37935	-2.49355
C	8.07659	-4.46317	-1.81174
C	8.36003	-3.57304	-0.77842
C	7.41846	-2.60875	-0.41867
C	6.20087	-2.54165	-1.08748
C	4.14519	-3.42059	2.77139
C	-1.84814	-5.36064	0.06754
C	-5.71431	-1.53169	0.07715
C	-3.63338	-3.04414	-2.16577
H	0.59039	1.76155	-0.88477
H	1.09292	0.43528	-1.86793

H	-0.47447	1.02788	-2.05193
H	-3.03917	-1.20518	1.69950
H	-3.71138	1.15924	-1.34530
H	-2.53159	0.17647	-2.26711
H	-4.22614	-0.11052	-2.35386
H	-3.86495	-2.62750	-3.15488
H	-2.59893	-3.39915	-2.19952
H	-4.27121	-3.92801	-2.03879
H	-5.75748	-0.71737	0.81174
H	-6.00419	-2.44981	0.60645
H	-6.49868	-1.33794	-0.66640
H	3.00586	-0.32102	2.83810
H	2.58368	0.48433	1.36313
H	3.06976	-1.22287	1.38464
H	0.31734	0.14391	5.37173
H	1.59332	1.05374	4.56335
H	-0.10022	1.39518	4.20656
H	0.66261	-3.36125	2.52771
H	-0.56373	-2.89266	3.68832
H	1.12744	-3.01211	4.18395
H	-2.02329	5.61923	-0.15984
H	-1.36253	6.06504	1.40689
H	-2.12962	7.95887	2.52805
H	-3.62122	9.93803	2.62673
H	-5.55209	10.11308	1.07272
H	-5.95034	8.32262	-0.60332
H	-4.44478	6.36255	-0.70028
H	-4.02170	3.86539	3.02671
H	-4.15422	5.59055	2.74142
H	-1.35897	3.17899	3.49414
H	0.33304	3.56775	5.24807
H	0.24523	5.64483	6.62205
H	-1.56824	7.29729	6.23401
H	-3.25966	6.89021	4.46783

H	2.31830	5.47316	-0.27929
H	2.09243	5.68853	-2.72992
H	0.10638	4.75532	-3.86645
H	-0.35620	4.26470	1.93390
H	1.00461	6.39384	1.84316
H	2.46926	5.39989	1.88402
H	1.32526	5.33932	3.22904
H	1.38785	2.80412	2.89948
H	2.28045	2.86136	1.36774
H	0.69065	2.09309	1.44443
H	-2.28288	2.52806	-2.22210
H	-3.45714	4.55219	-1.56350
H	-4.11592	3.94371	-3.08688
H	-3.02729	5.34754	-3.08905
H	-0.90023	2.41971	-4.31916
H	-1.64898	3.93292	-4.85915
H	-2.65026	2.50564	-4.59815
H	-7.57467	1.96734	1.45429
H	-7.63288	0.70536	3.57835
H	-5.51308	0.19307	4.74937
H	-4.47430	3.01281	-0.29111
H	-5.55314	4.90181	0.91853
H	-7.14564	4.20084	0.59795
H	-6.15671	4.79082	-0.74343
H	-6.14177	2.52263	-1.96562
H	-7.34543	2.07566	-0.75421
H	-5.94236	1.04020	-1.00462
H	-2.16178	1.26163	3.55214
H	-3.30196	2.75097	5.28323
H	-2.12028	1.63404	5.97811
H	-3.85330	1.28654	6.10585
H	-2.63021	-1.16573	3.53776
H	-3.63940	-1.00831	4.97882
H	-1.90423	-0.69429	5.07227

H	2.50769	-5.54715	-1.52151
H	4.23979	-5.56519	-1.26655
H	5.28035	-7.18031	-2.54875
H	5.54676	-8.45046	-4.66278
H	4.05501	-7.97749	-6.59166
H	2.28204	-6.24552	-6.38171
H	2.02274	-4.99543	-4.26258
H	4.43314	-2.25016	-3.14374
H	4.67278	-3.84191	-3.83456
H	6.64663	-5.06062	-3.31215
H	8.80493	-5.21747	-2.09569
H	9.30984	-3.62969	-0.25477
H	7.63231	-1.91008	0.38525
H	5.46193	-1.80053	-0.80223
H	3.64849	-6.39197	2.60217
H	1.61892	-7.79393	2.50524
H	-0.28831	-7.08977	1.10135
H	4.16208	-3.27756	0.63174
H	5.60848	-5.23131	0.19079
H	5.52956	-5.70739	1.89464
H	6.33930	-4.21244	1.42105
H	4.96051	-2.69621	2.87861
H	4.26217	-4.17919	3.55281
H	3.19686	-2.91137	2.95128
H	-0.52114	-3.83166	-0.56139
H	0.56668	-5.02638	-2.41319
H	-1.19028	-5.16038	-2.57010
H	-0.24524	-6.53341	-1.96423
H	-1.94231	-5.04137	1.10912
H	-1.98525	-6.44680	0.02215
H	-2.66308	-4.90507	-0.49784
H	1.54122	-0.70865	-6.02753
H	3.01517	1.27015	-6.03669
H	4.28403	1.86264	-3.99490

H	1.34632	-3.18599	-3.33146
H	1.66407	-3.30519	-5.85074
H	0.18116	-2.35252	-6.02678
H	0.11253	-3.92939	-5.25109
H	-0.26753	-1.56973	-2.37027
H	-1.10513	-2.70893	-3.41815
H	-0.74826	-1.06550	-3.98254
H	4.22424	-0.19007	-0.87814
H	6.26137	-0.14069	-2.38456
H	6.40812	0.94219	-0.99101
H	6.03728	1.60221	-2.58651
H	2.73810	1.80777	-0.76784
H	3.88853	2.73547	-1.71279
H	4.34261	2.16299	-0.09826

216

Int4

C	0.64888	-5.58960	0.16547
C	1.76681	-4.73264	0.32580
C	2.79393	-5.07907	1.23302
C	2.68336	-6.27530	1.94717
C	1.59954	-7.12637	1.78496
C	0.59151	-6.77790	0.89409
N	1.73355	-3.49604	-0.35891
C	2.50514	-3.04058	-1.35675
N	3.41475	-3.77850	-2.07305
C	3.56356	-5.22099	-1.90417
C	3.92285	-5.92151	-3.18850
C	3.19235	-5.69332	-4.35696
C	3.50936	-6.36414	-5.53343
C	4.56269	-7.27886	-5.55474
C	5.29472	-7.51445	-4.39315
C	4.97738	-6.83519	-3.21745
C	3.99523	-4.19309	1.49728
C	5.32572	-4.94458	1.39089

C	-0.44934	-5.26332	-0.82744
C	-0.12654	-5.85962	-2.20322
Y	0.77375	-1.38870	0.13530
C	-1.75165	-1.98536	0.21908
C	-2.54573	-2.69928	0.84357
Al	-3.95821	-1.62700	-0.75524
C	-3.40251	0.12127	-1.61692
Y	-1.71489	0.68354	0.37546
C	0.08119	0.49419	-1.37076
N	-3.00232	2.19809	1.55048
C	-4.24984	1.99316	2.16184
C	-5.46821	2.33804	1.52693
C	-6.67281	2.01625	2.15454
C	-6.70542	1.33550	3.36501
C	-5.50824	0.96123	3.96238
C	-4.27730	1.27672	3.38554
C	-5.51605	3.00670	0.16825
C	-6.13393	4.40375	0.25624
C	-3.00441	0.81902	4.06962
C	-2.92650	1.29431	5.52277
C	-2.45166	3.32469	1.03729
N	-1.54577	3.02624	0.09431
C	-0.57170	3.80600	-0.56513
C	0.56011	4.33546	0.09468
C	1.53998	4.97917	-0.66309
C	1.42978	5.09940	-2.04313
C	0.32115	4.56488	-2.68872
C	-0.68399	3.91374	-1.97292
C	0.73384	4.19296	1.59010
C	1.35823	5.42060	2.25506
C	-1.89607	3.34039	-2.67799
C	-3.08161	4.30803	-2.60246
N	-2.79849	4.58638	1.45709
C	-2.15448	5.77696	0.88699

C	-3.03015	7.00164	0.91368
C	-2.75972	8.04193	1.80603
C	-3.56055	9.18213	1.83321
C	-4.64040	9.29553	0.96007
C	-4.90455	8.27174	0.05056
C	-4.09981	7.13651	0.02510
C	-3.35902	4.78853	2.79608
C	-2.31275	4.97559	3.87026
C	-2.28018	6.14104	4.63820
C	-1.32848	6.30520	5.64440
C	-0.39864	5.29915	5.89620
C	-0.42800	4.12667	5.14058
C	-1.37846	3.96922	4.13663
C	-6.28076	2.15025	-0.84842
C	-2.84872	-0.70227	3.98931
C	1.53293	2.93313	1.92806
C	-1.62408	2.89203	-4.11187
N	2.30157	-1.72257	-1.59838
C	2.38392	-1.05911	-2.84001
C	3.10264	0.15897	-2.90986
C	3.03212	0.93099	-4.07076
C	2.27756	0.52620	-5.16238
C	1.58440	-0.67786	-5.09657
C	1.61518	-1.48107	-3.95499
C	3.96612	0.64684	-1.76247
C	5.44422	0.68036	-2.17166
C	0.75563	-2.73143	-3.90423
C	0.58342	-3.40338	-5.26976
O	-0.20751	0.04253	1.61546
Al	0.87546	-0.62768	2.87774
C	0.97179	0.35854	4.57402
C	0.34088	-2.57034	2.92367
C	2.64598	-0.56951	1.74828
C	3.54084	2.02359	-1.23997

C	-0.62568	-2.44772	-3.30644
C	4.57252	-3.12536	-2.68899
C	5.79074	-3.13138	-1.79861
C	6.87350	-3.96896	-2.07215
C	8.01117	-3.94424	-1.26541
C	8.07869	-3.07275	-0.18106
C	6.99710	-2.23852	0.10465
C	5.85932	-2.27617	-0.69392
C	3.88858	-3.54011	2.88019
C	-1.83303	-5.72461	-0.37095
C	-5.27214	-1.34993	0.69263
C	-4.24401	-3.02493	-2.09911
H	-0.28141	1.52926	-1.46467
H	1.16309	0.68364	-1.30644
H	-0.05602	0.07204	-2.37194
H	-3.16570	-3.33817	1.43807
H	-3.64592	1.09832	-1.17235
H	-2.41823	0.16506	-2.10446
H	-4.10499	0.06622	-2.45985
H	-5.03895	-2.74040	-2.80164
H	-3.34775	-3.22710	-2.69458
H	-4.55384	-3.97204	-1.64087
H	-4.95332	-0.64593	1.47066
H	-5.54762	-2.28525	1.19648
H	-6.20039	-0.93634	0.27666
H	3.31375	-0.48387	2.61537
H	2.79453	0.36130	1.18161
H	3.10978	-1.38596	1.17130
H	0.97974	-0.30248	5.44898
H	1.85872	0.99970	4.64226
H	0.09378	1.00917	4.66863
H	0.46078	-3.25192	2.06627
H	-0.72097	-2.62395	3.19130
H	0.90077	-3.06875	3.72567

H	-1.88201	5.55530	-0.14201
H	-1.22764	5.99300	1.42895
H	-1.91625	7.95004	2.48468
H	-3.33922	9.98204	2.53402
H	-5.26751	10.18209	0.98003
H	-5.73423	8.36115	-0.64476
H	-4.29656	6.34741	-0.69211
H	-4.00039	3.94409	3.04325
H	-4.00193	5.67118	2.75060
H	-1.40370	3.05237	3.55812
H	0.28160	3.32824	5.33584
H	0.34243	5.42395	6.68035
H	-1.31543	7.21916	6.23147
H	-3.00902	6.92390	4.44571
H	2.41045	5.38799	-0.15951
H	2.20598	5.60234	-2.61261
H	0.23857	4.64766	-3.76747
H	-0.25842	4.06551	2.02449
H	0.86425	6.34804	1.94752
H	2.42252	5.51523	2.01410
H	1.27117	5.33528	3.34170
H	1.60387	2.80380	3.01265
H	2.54828	3.00174	1.52376
H	1.05571	2.04040	1.51837
H	-2.19290	2.45227	-2.11508
H	-3.33804	4.51960	-1.56085
H	-3.96733	3.87885	-3.08394
H	-2.84660	5.25705	-3.09666
H	-0.75355	2.23034	-4.15938
H	-1.44748	3.73919	-4.78376
H	-2.49085	2.34747	-4.50085
H	-7.60684	2.28790	1.66999
H	-7.65449	1.08723	3.83070
H	-5.52540	0.41051	4.89879

H	-4.49049	3.11343	-0.19647
H	-5.57593	5.04458	0.94135
H	-7.16877	4.34860	0.61073
H	-6.14687	4.88438	-0.72799
H	-6.19334	2.57935	-1.85262
H	-7.34603	2.09884	-0.60038
H	-5.89972	1.12743	-0.87307
H	-2.16363	1.25454	3.52285
H	-3.05561	2.37706	5.59409
H	-1.95318	1.03910	5.95400
H	-3.69629	0.81849	6.13987
H	-2.81671	-1.05093	2.95297
H	-3.68383	-1.21039	4.48424
H	-1.91987	-1.01699	4.47468
H	2.62553	-5.62438	-1.52845
H	4.32359	-5.44226	-1.14836
H	5.55510	-7.00982	-2.31317
H	6.11827	-8.22273	-4.40222
H	4.81238	-7.80254	-6.47284
H	2.93333	-6.17337	-6.43450
H	2.38391	-4.97228	-4.33976
H	4.30156	-2.10570	-2.95330
H	4.79096	-3.64744	-3.62370
H	6.82299	-4.63903	-2.92661
H	8.84749	-4.60014	-1.48993
H	8.96823	-3.04451	0.44154
H	7.03998	-1.55775	0.95011
H	5.01842	-1.62685	-0.47310
H	3.46609	-6.54003	2.65211
H	1.53794	-8.05172	2.35032
H	-0.25812	-7.44055	0.76300
H	4.00320	-3.39813	0.74673
H	5.49274	-5.35831	0.39353
H	5.37895	-5.76740	2.11059

H	6.15550	-4.26605	1.59898
H	4.71895	-2.84376	3.04018
H	3.92678	-4.30061	3.66783
H	2.95226	-2.99294	2.98968
H	-0.48097	-4.17514	-0.93491
H	0.80118	-5.45453	-2.61317
H	-0.92863	-5.64807	-2.91833
H	-0.01282	-6.94697	-2.13393
H	-2.02479	-5.43523	0.66592
H	-1.94490	-6.81224	-0.44044
H	-2.60318	-5.27528	-1.00400
H	0.98968	-0.99174	-5.94837
H	2.22904	1.13864	-6.05796
H	3.57836	1.86925	-4.11269
H	1.24986	-3.45251	-3.25019
H	1.53865	-3.51073	-5.79249
H	-0.09006	-2.83378	-5.91866
H	0.14138	-4.39713	-5.14440
H	-0.57573	-2.08089	-2.27905
H	-1.23132	-3.35768	-3.29691
H	-1.15451	-1.68900	-3.89374
H	3.84773	-0.07269	-0.94663
H	5.78116	-0.28167	-2.56521
H	6.07742	0.93062	-1.31374
H	5.61382	1.43654	-2.94587
H	2.50410	2.04352	-0.89804
H	3.63225	2.79131	-2.01444
H	4.17832	2.32231	-0.40056

216

Int5

C	-1.87621	0.79060	-7.76269
C	-2.95963	0.72680	-6.88002
C	-4.24626	0.95860	-7.37057
C	-4.45040	1.25313	-8.71718

C	-3.36520	1.32380	-9.58742
C	-2.07639	1.09081	-9.10668
C	-2.75365	0.42804	-5.41922
N	-2.10252	1.54243	-4.70403
C	-2.57385	2.85769	-5.13540
C	-4.03368	3.10580	-4.84405
C	-4.77616	3.92760	-5.69557
C	-6.10470	4.23130	-5.40999
C	-6.71363	3.70524	-4.27126
C	-5.98612	2.86823	-3.42758
C	-4.65683	2.56983	-3.71567
C	-1.70283	1.38379	-3.39548
N	-1.75820	0.24035	-2.68730
C	-2.02883	-1.08195	-3.09083
C	-0.98573	-1.87819	-3.62754
C	-1.25092	-3.20609	-3.96754
C	-2.50162	-3.77292	-3.75477
C	-3.50208	-3.00442	-3.17419
C	-3.28802	-1.66831	-2.82966
C	0.40382	-1.31709	-3.85193
C	0.62295	-0.90713	-5.31062
C	-4.40784	-0.89860	-2.16076
C	-5.66212	-0.80111	-3.03423
N	-1.27978	2.41543	-2.62934
Y	-1.27460	1.29718	-0.61325
C	1.58473	1.22271	-0.65749
C	1.46436	0.03965	-0.98436
C	-0.69450	3.64463	-2.99474
C	-1.30783	4.84107	-2.55062
C	-0.63726	6.05294	-2.71776
C	0.60923	6.10708	-3.32928
C	1.18191	4.93507	-3.80881
C	0.55299	3.69694	-3.66024
C	-2.67727	4.82334	-1.90545

C	-2.58866	5.08841	-0.40188
C	1.19781	2.46919	-4.26985
C	1.09280	2.54162	-5.79908
C	2.65243	2.27713	-3.83637
C	-3.65152	5.79495	-2.57561
C	-3.53578	1.82228	0.36133
Al	-2.36537	2.03582	2.12734
C	-2.57050	3.84930	2.87425
Y	0.71899	0.19942	1.74603
C	-1.00060	-0.99968	0.26697
Al	2.26170	2.88174	0.31992
C	0.94953	4.34025	0.41743
O	-0.71227	1.72696	1.44824
C	-2.94515	0.51591	3.25717
C	2.54579	2.03214	2.19234
N	0.87794	-1.02955	3.67917
C	1.76905	-1.94674	3.20933
N	2.47601	-2.76566	4.05668
C	2.85666	-2.27817	5.39029
C	4.18914	-1.57002	5.37155
C	5.33745	-2.18268	5.87696
C	6.56490	-1.52092	5.84884
C	6.65486	-0.23678	5.31624
C	5.51374	0.38048	4.80246
C	4.29317	-0.28572	4.82818
N	1.87893	-1.89591	1.87921
C	2.65583	-2.64842	0.97394
C	2.03522	-3.72920	0.29662
C	2.73972	-4.38872	-0.71220
C	4.02396	-3.99697	-1.07244
C	4.63082	-2.94962	-0.39056
C	3.97682	-2.27294	0.64235
C	0.65975	-4.23123	0.69308
C	0.75627	-5.55290	1.46638

C	4.70620	-1.16894	1.37932
C	4.91026	0.05361	0.48228
C	4.00183	3.34747	-0.49632
C	0.14124	-1.11668	4.87997
C	0.13668	-0.00237	5.75149
C	-0.67677	-0.01885	6.88602
C	-1.46666	-1.11818	7.18659
C	-1.45419	-2.21525	6.33191
C	-0.67632	-2.23876	5.17358
C	0.99497	1.21430	5.48310
C	1.90202	1.56179	6.66624
C	-0.82076	-3.40237	4.21011
C	-1.86913	-3.06022	3.14746
C	0.14315	2.41594	5.06420
C	-1.18375	-4.72447	4.89154
C	-0.28521	-4.38914	-0.50085
C	6.05279	-1.63051	1.94442
C	1.50781	-2.28201	-3.41064
C	-4.75341	-1.51552	-0.79968
C	3.37553	-3.79483	3.54554
C	3.47169	-4.98548	4.46240
C	4.71385	-5.55747	4.74110
C	4.80617	-6.68829	5.55130
C	3.65498	-7.25262	6.09708
C	2.41111	-6.68240	5.82596
C	2.32243	-5.55736	5.01241
H	1.43965	-0.98280	-1.29863
H	-3.54985	4.28984	2.64537
H	-1.81104	4.54993	2.50883
H	-2.48626	3.82600	3.96832
H	-2.17807	0.17590	3.96598
H	-3.24546	-0.36210	2.67171
H	-3.81259	0.79722	3.86968
H	1.44512	5.26503	0.74309

H	0.14193	4.13157	1.12504
H	0.50571	4.56827	-0.55922
H	4.56788	2.50689	-0.90962
H	4.64954	3.84407	0.23989
H	3.84304	4.06610	-1.31002
H	2.89701	-3.14158	6.05856
H	2.07681	-1.62002	5.76569
H	5.26500	-3.18370	6.29436
H	7.45045	-2.00809	6.24689
H	7.60933	0.28119	5.29922
H	5.57296	1.38069	4.38289
H	3.40522	0.19711	4.43549
H	4.37640	-3.38165	3.38222
H	3.01140	-4.12478	2.57514
H	5.61250	-5.10979	4.32384
H	5.77873	-7.12400	5.76158
H	3.72642	-8.12990	6.73325
H	1.50834	-7.11430	6.24855
H	1.36132	-5.10031	4.80880
H	-0.68236	0.84571	7.54392
H	-2.09263	-1.12203	8.07391
H	-2.08598	-3.06715	6.56018
H	1.64711	0.95779	4.64412
H	1.32174	1.88250	7.53761
H	2.57757	2.38106	6.39829
H	2.51301	0.70468	6.96364
H	-0.44530	2.20340	4.16734
H	0.76822	3.29025	4.85309
H	-0.56474	2.68869	5.85341
H	0.13528	-3.55157	3.70154
H	-2.85136	-2.92847	3.61263
H	-1.94377	-3.85278	2.39491
H	-1.62244	-2.12876	2.63963
H	-0.55452	-4.92266	5.76463

H	-1.06732	-5.55316	4.18619
H	-2.22649	-4.73184	5.22555
H	2.26881	-5.22210	-1.22598
H	4.55157	-4.51152	-1.87011
H	5.64101	-2.65178	-0.65754
H	0.23040	-3.48878	1.37024
H	1.20745	-6.33612	0.84760
H	-0.24154	-5.89298	1.76394
H	1.36019	-5.46073	2.37269
H	-0.36583	-3.47235	-1.08727
H	-1.29183	-4.64690	-0.15644
H	0.04460	-5.18759	-1.17400
H	4.08298	-0.87222	2.22883
H	5.59104	-0.18182	-0.34335
H	5.34311	0.88508	1.04720
H	3.97175	0.39421	0.04400
H	5.94646	-2.47518	2.62976
H	6.52235	-0.81642	2.50168
H	6.73772	-1.93125	1.14494
H	-3.72169	0.20481	-4.96006
H	-2.13918	-0.46294	-5.31483
H	-5.08920	0.92618	-6.68624
H	-5.45687	1.43360	-9.08332
H	-3.52113	1.55760	-10.63657
H	-1.22624	1.14049	-9.78091
H	-0.87166	0.61425	-7.39184
H	-2.39643	2.94496	-6.20928
H	-1.96913	3.62925	-4.66116
H	-4.30702	4.32621	-6.59155
H	-6.66739	4.87457	-6.08043
H	-7.74991	3.94045	-4.04737
H	-6.45078	2.44482	-2.54201
H	-4.10129	1.91283	-3.05555
H	-0.45680	-3.80979	-4.39675

H	-2.68893	-4.80818	-4.02374
H	-4.47456	-3.44948	-2.98108
H	0.47930	-0.40919	-3.24583
H	0.42843	-1.74689	-5.98660
H	1.65491	-0.57604	-5.46488
H	-0.03262	-0.08028	-5.58671
H	1.33130	-2.69679	-2.41390
H	2.47760	-1.77398	-3.40193
H	1.59810	-3.13383	-4.09269
H	-4.04285	0.11878	-1.98913
H	-5.46010	-0.29878	-3.98379
H	-6.44291	-0.23266	-2.51863
H	-6.06544	-1.79391	-3.25878
H	-5.17265	-2.51976	-0.92250
H	-5.49528	-0.90658	-0.27201
H	-3.86650	-1.60806	-0.16700
H	2.14573	4.97735	-4.30840
H	1.12590	7.05557	-3.44171
H	-1.10084	6.96689	-2.35750
H	0.64164	1.59279	-3.92833
H	0.04975	2.57805	-6.12368
H	1.56675	1.67322	-6.26696
H	1.59443	3.43934	-6.17570
H	3.29686	3.07957	-4.21000
H	3.04127	1.33293	-4.23374
H	2.74353	2.25013	-2.74980
H	-3.07981	3.81716	-2.05406
H	-1.92316	4.38309	0.10186
H	-3.57324	5.01477	0.07018
H	-2.18767	6.08790	-0.20376
H	-3.35619	6.83744	-2.41573
H	-4.65566	5.66529	-2.15966
H	-3.71210	5.61774	-3.65217
H	3.34191	2.73283	2.47745

H	3.02985	1.05604	2.36827
H	-1.10768	-1.40261	-0.74853
H	-0.46613	-1.80809	0.78196
H	-2.00832	-1.00776	0.71446
H	1.77289	2.21113	2.95384
H	-4.39822	2.19367	0.93018
H	-3.56600	2.45224	-0.54468
H	-3.83987	0.80474	0.07327

216

TS4

C	3.25750	2.96440	-1.81314
C	2.07004	3.06605	-1.08156
C	0.88884	3.39839	-1.74721
C	0.89203	3.62954	-3.12175
C	2.08032	3.53379	-3.84248
C	3.26443	3.19961	-3.18463
C	2.06871	2.83102	0.40490
N	2.76120	3.90425	1.13399
C	2.38614	5.24089	0.68050
C	0.93301	5.56722	0.92505
C	0.23367	6.34986	0.00386
C	-1.08548	6.72540	0.24734
C	-1.72628	6.30971	1.41322
C	-1.04159	5.51076	2.32752
C	0.27785	5.14043	2.08257
C	3.19129	3.73106	2.42548
N	3.13936	2.59356	3.13610
C	2.70013	1.29560	2.79979
C	3.56322	0.42067	2.09260
C	3.19708	-0.91428	1.92865
C	1.99616	-1.40159	2.43419
C	1.13976	-0.53372	3.09652
C	1.46813	0.81167	3.28879
C	4.87541	0.92359	1.52701

C	5.20077	0.34878	0.14723
C	0.47465	1.71346	3.98933
C	-0.79158	1.90543	3.14756
N	3.67960	4.75410	3.17006
Y	3.99598	3.63670	5.12834
C	6.48818	3.47184	5.29316
C	7.02460	2.37965	5.08213
C	4.39042	5.91520	2.79771
C	3.86113	7.17375	3.17171
C	4.63303	8.31939	2.97485
C	5.89861	8.24375	2.40585
C	6.40670	7.00566	2.02808
C	5.68181	5.82824	2.22282
C	2.47568	7.28643	3.77375
C	2.54355	7.62221	5.26544
C	6.27560	4.49937	1.80060
C	6.07499	4.28170	0.29619
C	7.75274	4.35294	2.16921
C	1.59479	8.29128	3.02725
C	1.78982	4.28433	6.34475
Al	3.11601	4.59977	7.90983
C	2.74255	6.28166	8.84574
Y	5.56558	2.50362	7.83987
C	3.93316	1.40855	6.26479
Al	6.69370	5.24331	6.65989
C	5.84573	7.02163	6.82197
O	4.69310	4.38703	7.06607
C	2.92036	2.98030	9.11752
C	7.29385	4.30300	8.42835
N	5.92128	1.28049	9.79044
C	6.86923	0.45870	9.27169
N	7.62691	-0.36690	10.06563
C	7.97228	0.04147	11.43378
C	9.27771	0.79777	11.48740

C	10.42867	0.20671	12.01156
C	11.63101	0.91219	12.05687
C	11.69273	2.21903	11.57837
C	10.54894	2.81540	11.04536
C	9.35342	2.10617	10.99890
N	6.96995	0.58789	7.94223
C	7.80006	-0.04763	6.99253
C	7.21556	-1.04640	6.17200
C	7.92475	-1.51420	5.06440
C	9.19243	-1.02924	4.76048
C	9.78298	-0.09747	5.60586
C	9.11714	0.39452	6.73210
C	5.87240	-1.66151	6.51297
C	6.05860	-3.01764	7.20697
C	9.82629	1.38710	7.62756
C	9.97685	2.74855	6.94666
C	8.39186	5.69508	5.69289
C	5.20002	1.06559	10.98565
C	5.16289	2.09911	11.95070
C	4.34047	1.96437	13.07055
C	3.57345	0.82479	13.26503
C	3.62444	-0.19631	12.32220
C	4.41577	-0.10063	11.17601
C	6.01480	3.34149	11.80532
C	6.92686	3.55891	13.01632
C	4.33333	-1.19343	10.12531
C	3.26811	-0.84169	9.08235
C	5.16640	4.58588	11.52829
C	4.04575	-2.58042	10.70733
C	4.94721	-1.81297	5.30298
C	11.20092	0.88009	8.07826
C	6.00730	0.64608	2.51559
C	0.11345	1.19215	5.38429
C	8.57622	-1.31952	9.49975

C	8.70376	-2.56849	10.33156
C	9.96419	-3.08793	10.63010
C	10.08760	-4.26571	11.36596
C	8.94897	-4.93037	11.81674
C	7.68649	-4.41350	11.52507
C	7.56753	-3.24115	10.78577
H	7.55416	1.47264	4.87315
H	1.80954	6.20521	9.42118
H	2.64203	7.14281	8.17715
H	3.53741	6.52369	9.56180
H	3.64326	2.21144	9.42853
H	2.08083	2.40202	8.70857
H	2.58548	3.37860	10.08375
H	6.60701	7.77679	7.05976
H	5.03349	7.10704	7.54903
H	5.44186	7.31062	5.84097
H	9.03292	4.88202	5.33947
H	9.00529	6.34708	6.33414
H	8.12142	6.30131	4.81660
H	8.03594	-0.86332	12.04328
H	7.16349	0.64284	11.84074
H	10.37843	-0.81270	12.38549
H	12.51880	0.44080	12.46868
H	12.62723	2.77106	11.61761
H	10.58659	3.83259	10.66642
H	8.46557	2.57331	10.58646
H	9.56142	-0.85460	9.39550
H	8.24631	-1.58926	8.49864
H	10.85218	-2.56161	10.28887
H	11.07404	-4.65980	11.59298
H	9.04423	-5.84458	12.39519
H	6.79321	-4.92409	11.87368
H	6.59116	-2.82572	10.56728
H	4.31156	2.76586	13.80370

H	2.94006	0.72911	14.14203
H	3.01756	-1.08319	12.47260
H	6.65558	3.17706	10.93606
H	6.34850	3.78414	13.91856
H	7.60297	4.40114	12.83587
H	7.53930	2.67617	13.22021
H	4.55943	4.46884	10.62675
H	5.80055	5.46939	11.39776
H	4.47761	4.78477	12.35637
H	5.29636	-1.24873	9.61049
H	2.28030	-0.78487	9.55195
H	3.23083	-1.59320	8.28641
H	3.46636	0.12527	8.62107
H	4.68444	-2.80353	11.56714
H	4.21007	-3.34837	9.94488
H	3.00484	-2.67142	11.03474
H	7.47386	-2.27174	4.43026
H	9.72328	-1.39020	3.88455
H	10.78447	0.26301	5.38923
H	5.39148	-0.99616	7.23489
H	6.57464	-3.72443	6.54812
H	5.08592	-3.44842	7.46850
H	6.64418	-2.93559	8.12610
H	4.85210	-0.88387	4.73938
H	3.94446	-2.10914	5.62639
H	5.30702	-2.58591	4.61530
H	9.21381	1.51970	8.52382
H	10.62734	2.67107	6.06846
H	10.42403	3.47662	7.63136
H	9.02013	3.14788	6.61105
H	11.15450	-0.12607	8.50329
H	11.61222	1.54637	8.84043
H	11.90578	0.85080	7.24071
H	1.03499	2.74483	0.76006

H	2.55930	1.88662	0.61854
H	-0.03262	3.49264	-1.17958
H	-0.03349	3.88891	-3.62764
H	2.08510	3.71659	-4.91310
H	4.19337	3.11892	-3.74175
H	4.17851	2.70845	-1.29968
H	2.60433	5.30931	-0.38791
H	3.01976	5.97768	1.17240
H	0.72934	6.66260	-0.91173
H	-1.61503	7.33793	-0.47675
H	-2.75503	6.60018	1.60469
H	-1.53459	5.17330	3.23437
H	0.80297	4.51676	2.79892
H	3.86264	-1.58444	1.39328
H	1.72742	-2.44529	2.30090
H	0.18987	-0.90319	3.47354
H	4.79247	2.00892	1.41816
H	5.43566	-0.71951	0.19639
H	6.07965	0.85073	-0.27055
H	4.36911	0.47578	-0.55233
H	5.75123	1.03371	3.50296
H	6.94246	1.11749	2.19588
H	6.18009	-0.43019	2.61669
H	0.94945	2.69327	4.10194
H	-0.56441	2.34157	2.17144
H	-1.49560	2.57244	3.65621
H	-1.29722	0.94888	2.97857
H	-0.43699	0.24756	5.32181
H	-0.52146	1.91041	5.91355
H	1.00780	1.00964	5.98623
H	7.39922	6.94767	1.59196
H	6.49005	9.14320	2.26276
H	4.23380	9.28418	3.27475
H	5.73329	3.71219	2.33549

H	5.02041	4.34954	0.02304
H	6.44818	3.29675	-0.00730
H	6.62398	5.03911	-0.27417
H	8.38576	5.02404	1.57895
H	8.08638	3.33011	1.96375
H	7.92115	4.55973	3.22642
H	2.00897	6.30275	3.66931
H	3.15545	6.90437	5.81660
H	1.54252	7.62802	5.71029
H	2.99485	8.60685	5.42659
H	1.96195	9.31608	3.14844
H	0.57133	8.25497	3.41386
H	1.55281	8.06907	1.95805
H	8.09498	5.03986	8.56592
H	7.83749	3.35199	8.55080
H	3.70396	1.10170	5.23064
H	4.53587	0.55357	6.60039
H	2.98088	1.32534	6.80469
H	6.63820	4.44603	9.30044
H	0.90891	4.61732	6.90985
H	1.74201	4.90905	5.43714
H	1.53643	3.25240	6.06480

216

Complex9

C	-2.03747	0.55401	-7.82583
C	-3.14062	0.65725	-6.97249
C	-4.38749	0.98000	-7.51142
C	-4.53254	1.19716	-8.88049
C	-3.42778	1.09793	-9.72329
C	-2.17855	0.77542	-9.19241
C	-2.98721	0.43924	-5.49171
N	-2.21433	1.51375	-4.84953
C	-2.62170	2.84525	-5.29688
C	-4.02808	3.21464	-4.89083

C	-4.79506	4.03363	-5.72293
C	-6.06689	4.44935	-5.33489
C	-6.59411	4.03933	-4.11129
C	-5.84309	3.20618	-3.28401
C	-4.57101	2.79662	-3.67355
C	-1.74799	1.35901	-3.56417
N	-1.84728	0.25358	-2.81119
C	-2.27662	-1.06106	-3.10699
C	-1.41694	-1.95326	-3.79780
C	-1.74318	-3.30841	-3.85151
C	-2.90713	-3.80019	-3.27299
C	-3.78451	-2.91084	-2.66907
C	-3.49721	-1.54585	-2.58270
C	-0.17787	-1.46809	-4.52578
C	-0.10610	-2.01831	-5.95521
C	-4.53860	-0.62445	-1.98272
C	-5.77803	-0.53281	-2.88119
N	-1.17313	2.37768	-2.87624
Y	-0.91702	1.31777	-0.85862
C	1.27958	0.32426	-0.61678
C	2.28221	-0.27043	-1.00784
C	-0.49362	3.50343	-3.38438
C	-0.95613	4.79011	-3.02374
C	-0.22842	5.90994	-3.42470
C	0.93438	5.77916	-4.17704
C	1.39324	4.51004	-4.50982
C	0.71137	3.35961	-4.10787
C	-2.20541	4.94361	-2.18421
C	-1.83240	5.21165	-0.72280
C	1.29081	1.99735	-4.42716
C	1.16603	1.68888	-5.92154
C	2.73935	1.85745	-3.95045
C	-3.16908	6.00525	-2.71463
C	-3.23174	1.97835	0.22198

Al	-2.12418	2.19117	1.94044
C	-2.53352	3.91604	2.78437
Y	0.53169	0.05259	1.77454
C	-1.30671	-0.87105	0.38618
Al	1.06381	3.12905	1.30528
C	0.87010	4.73589	2.41869
O	-0.43005	2.06029	1.24499
C	-2.36023	0.61768	3.16032
C	2.37590	1.88678	2.30173
N	0.86482	-1.12358	3.74764
C	1.85233	-1.92792	3.27869
N	2.54107	-2.79376	4.09355
C	2.80990	-2.41973	5.48679
C	4.09533	-1.64135	5.63177
C	5.18441	-2.17549	6.32227
C	6.36353	-1.44361	6.46369
C	6.46284	-0.16630	5.91715
C	5.38101	0.37300	5.22019
C	4.21047	-0.36334	5.07559
N	2.05462	-1.74280	1.96886
C	2.91967	-2.38573	1.05951
C	2.36352	-3.39268	0.23183
C	3.13390	-3.92979	-0.79903
C	4.43491	-3.49465	-1.02766
C	4.98025	-2.52115	-0.19906
C	4.24837	-1.95465	0.84911
C	0.95930	-3.91486	0.46121
C	0.98377	-5.33609	1.03581
C	4.89719	-0.86811	1.68224
C	5.15009	0.39199	0.84377
C	1.56098	3.50379	-0.58520
C	0.09454	-1.31345	4.91516
C	0.02989	-0.25546	5.85138
C	-0.84450	-0.34950	6.93464

C	-1.63610	-1.47454	7.12034
C	-1.55325	-2.52196	6.20926
C	-0.70658	-2.46704	5.10033
C	0.90600	0.96885	5.70230
C	1.73580	1.24388	6.95890
C	-0.74667	-3.59012	4.08076
C	-1.84619	-3.32107	3.04866
C	0.10002	2.20230	5.29013
C	-0.94777	-4.97116	4.71041
C	0.10269	-3.85272	-0.80509
C	6.20776	-1.32734	2.32944
C	1.10119	-1.80600	-3.76112
C	-4.94990	-1.03992	-0.56664
C	3.52024	-3.73279	3.54841
C	3.65500	-4.96808	4.39794
C	4.90315	-5.36840	4.87597
C	5.02823	-6.52502	5.64505
C	3.90233	-7.28774	5.94728
C	2.65138	-6.89304	5.47124
C	2.53213	-5.74224	4.69926
H	3.14502	-0.80425	-1.34724
H	-3.60895	3.99530	2.99718
H	-2.26033	4.78256	2.17397
H	-2.01442	4.02709	3.74371
H	-1.58179	-0.13813	3.34810
H	-3.22449	0.03089	2.82019
H	-2.60106	0.98498	4.16559
H	1.84097	5.23232	2.55551
H	0.46340	4.53995	3.41595
H	0.20749	5.46910	1.94298
H	2.63471	3.29787	-0.69371
H	1.40328	4.56104	-0.83074
H	1.11615	2.94930	-1.42254
H	2.85781	-3.33924	6.07487

H	1.97058	-1.84274	5.86883
H	5.10476	-3.17149	6.74987
H	7.20330	-1.87153	7.00384
H	7.37864	0.40639	6.03097
H	5.44793	1.36665	4.78645
H	3.37346	0.05872	4.52996
H	4.49860	-3.25267	3.44617
H	3.20354	-4.02105	2.54840
H	5.77823	-4.76448	4.65041
H	6.00503	-6.82633	6.01252
H	3.99780	-8.18558	6.55085
H	1.76860	-7.48332	5.70011
H	1.56237	-5.42478	4.33427
H	-0.89784	0.47154	7.64437
H	-2.31264	-1.53845	7.96752
H	-2.17757	-3.39801	6.35379
H	1.60332	0.74679	4.89156
H	1.10282	1.52837	7.80607
H	2.43657	2.06563	6.77803
H	2.31764	0.36499	7.25068
H	-0.41238	2.05072	4.33604
H	0.75117	3.07533	5.18495
H	-0.66991	2.43785	6.03249
H	0.21083	-3.60885	3.55397
H	-2.83106	-3.34237	3.52766
H	-1.83318	-4.07220	2.25133
H	-1.73045	-2.33994	2.58879
H	-0.25921	-5.14064	5.54315
H	-0.78789	-5.75319	3.96054
H	-1.96741	-5.09779	5.08855
H	2.70417	-4.69852	-1.43511
H	5.02124	-3.91650	-1.83876
H	5.99836	-2.18307	-0.37184
H	0.49803	-3.26932	1.21403

H	1.44377	-6.03681	0.33053
H	-0.03462	-5.68436	1.23956
H	1.55121	-5.38984	1.96935
H	0.11011	-2.85287	-1.24138
H	-0.93704	-4.11406	-0.58524
H	0.46184	-4.55138	-1.56818
H	4.20463	-0.61109	2.48794
H	5.88313	0.19003	0.05466
H	5.54919	1.19470	1.47289
H	4.23590	0.75270	0.36682
H	6.05937	-2.17112	3.00805
H	6.64198	-0.51447	2.91686
H	6.94161	-1.62752	1.57437
H	-3.97901	0.36864	-5.03212
H	-2.48631	-0.50787	-5.31613
H	-5.24308	1.07867	-6.84916
H	-5.50788	1.44904	-9.28674
H	-3.53831	1.26978	-10.79003
H	-1.31398	0.69325	-9.84481
H	-1.06561	0.30667	-7.41164
H	-2.53224	2.87378	-6.38463
H	-1.92252	3.58544	-4.91291
H	-4.39063	4.34282	-6.68359
H	-6.64935	5.08897	-5.99190
H	-7.58596	4.36136	-3.80811
H	-6.24459	2.87283	-2.33146
H	-3.99697	2.14733	-3.02193
H	-1.07043	-3.99137	-4.36185
H	-3.13839	-4.86033	-3.31637
H	-4.72016	-3.27806	-2.25626
H	-0.24178	-0.37942	-4.59783
H	0.09963	-3.09351	-5.96163
H	0.70539	-1.53211	-6.50661
H	-1.03754	-1.85785	-6.50587

H	1.08340	-1.37885	-2.75850
H	1.97980	-1.41209	-4.28415
H	1.22160	-2.89035	-3.66584
H	-4.09467	0.37323	-1.93488
H	-5.52818	-0.17484	-3.88302
H	-6.51188	0.15888	-2.45483
H	-6.25639	-1.51227	-2.98644
H	-5.48443	-1.99559	-0.57642
H	-5.61859	-0.29282	-0.12648
H	-4.08256	-1.15465	0.08701
H	2.31530	4.40596	-5.07524
H	1.48638	6.66051	-4.49039
H	-0.58025	6.89909	-3.14578
H	0.70833	1.25365	-3.87608
H	0.12515	1.76315	-6.24519
H	1.52901	0.67937	-6.14323
H	1.75693	2.39526	-6.51502
H	3.40773	2.54568	-4.47855
H	3.09764	0.83935	-4.13575
H	2.81850	2.05569	-2.87925
H	-2.72927	3.98481	-2.22986
H	-1.17524	4.43384	-0.31881
H	-2.72336	5.25897	-0.08944
H	-1.28911	6.15763	-0.62573
H	-2.74523	7.01312	-2.64916
H	-4.09394	5.99709	-2.12924
H	-3.43324	5.81024	-3.75751
H	3.23924	2.53535	2.09592
H	2.79966	0.89531	2.07832
H	-1.63096	-1.13057	-0.63377
H	-0.75566	-1.78767	0.65283
H	-2.21600	-0.87760	0.99587
H	2.22886	1.94794	3.38735
H	-4.20610	2.13561	0.70454

H	-3.19772	2.76408	-0.54812
H	-3.37387	1.01123	-0.27798

24

PhSSPh without dispersion

S	3.46152	7.43772	1.70859
S	3.97296	8.39967	3.49930
C	2.84192	8.74987	0.66737
C	5.59211	9.08422	3.18307
C	6.73383	8.27594	3.24050
C	7.99150	8.83681	3.03708
C	8.11724	10.20465	2.78833
C	6.98214	11.01202	2.73847
C	5.71863	10.45484	2.93119
H	6.62776	7.21453	3.44331
H	8.87617	8.20755	3.08076
H	9.10091	10.64035	2.63623
H	7.07803	12.07706	2.54598
H	4.82791	11.07418	2.89021
C	1.49959	9.13998	0.74164
C	1.01790	10.13280	-0.10757
C	1.86895	10.73437	-1.03563
C	3.20487	10.34331	-1.11413
C	3.69447	9.35271	-0.26442
H	0.84394	8.66308	1.46387
H	-0.02436	10.43405	-0.04856
H	1.48890	11.50592	-1.69957
H	3.86800	10.80922	-1.83783
H	4.73276	9.04025	-0.31816

16

PhSMe without dispersion

S	5.33066	11.04892	4.27685
C	6.94088	10.61100	3.67596
C	7.65107	9.66066	4.42596
C	8.92113	9.25939	4.02913

C	9.50682	9.79451	2.88061
C	8.80392	10.73688	2.13588
C	7.52859	11.14752	2.52564
H	7.20271	9.23685	5.32088
H	9.45620	8.52220	4.62173
H	10.49914	9.47916	2.57211
H	9.24626	11.16310	1.23923
H	7.00422	11.88407	1.92663
C	4.72683	12.23432	3.05129
H	4.65486	11.78561	2.05715
H	5.34719	13.13365	3.01623
H	3.72425	12.51449	3.38207

238

int6 without dispersion

C	3.43488	-1.92423	-3.70712
C	2.23296	-2.68525	-3.67339
C	1.43239	-2.77160	-4.84521
C	1.84917	-2.09834	-5.99800
C	3.02149	-1.35377	-6.03232
C	3.80479	-1.27506	-4.88720
N	1.80804	-3.18689	-2.42093
C	1.62093	-4.44399	-1.96237
N	2.07003	-5.58340	-2.59394
C	3.05310	-5.55185	-3.68533
C	4.35233	-6.27971	-3.40350
C	4.74818	-7.34770	-4.21484
C	5.98014	-7.97277	-4.02433
C	6.83562	-7.53629	-3.01513
C	6.45227	-6.47100	-2.20048
C	5.22124	-5.84849	-2.39515
C	0.12671	-3.55330	-4.90301
C	-1.09549	-2.62456	-4.97113
C	4.32652	-1.78331	-2.48230
C	4.20884	-0.37970	-1.86821

Y	0.74715	-2.10126	-0.55820
C	2.09188	-1.38376	1.42489
Al	0.12297	-1.53298	2.28439
C	0.02494	-3.20122	3.34383
Y	-1.23854	1.19015	1.60161
C	0.03713	0.04627	3.60216
Al	-1.70177	-0.43826	-0.99724
C	-3.21579	-1.31866	-1.92026
N	-1.66818	3.42049	2.53227
C	-0.87182	4.59277	2.59550
C	0.08469	4.80043	3.62336
C	0.87572	5.95386	3.60072
C	0.77141	6.89587	2.58711
C	-0.12394	6.66449	1.55128
C	-0.93538	5.52764	1.52394
C	0.34104	3.79356	4.73368
C	1.75015	3.19230	4.59971
C	-1.85112	5.33295	0.32564
C	-1.04558	5.21583	-0.97699
N	-3.25561	1.86561	2.74879
C	-4.55597	1.30018	2.69956
C	-5.53919	1.72259	1.75855
C	-6.74816	1.02529	1.66881
C	-7.02175	-0.08222	2.45896
C	-6.07100	-0.49007	3.38427
C	-4.85578	0.18317	3.53259
C	-5.36284	2.92274	0.84172
C	-5.58988	2.57106	-0.63749
C	-3.90559	-0.29515	4.61711
C	-3.31060	-1.66546	4.27362
C	0.89173	2.06881	0.87860
C	-1.07883	-1.27566	0.70672
C	-2.09679	1.57624	-0.80291
N	0.93630	-4.41642	-0.78414

C	0.68846	-5.45716	0.15524
C	1.69700	-5.92829	1.03735
C	1.36592	-6.90094	1.98634
C	0.07768	-7.40490	2.10008
C	-0.91193	-6.92123	1.25447
C	-0.63801	-5.95300	0.28494
C	3.12562	-5.40325	1.03640
C	3.44859	-4.67461	2.35155
C	-1.78086	-5.48098	-0.60094
C	-2.87989	-4.79730	0.22391
C	0.00740	-0.28775	-2.21807
C	-2.87411	3.13800	3.05649
N	-3.64314	3.98894	3.83993
C	-4.66218	3.47711	4.75779
C	-4.24455	3.36644	6.21242
C	-3.00290	2.84759	6.59072
C	-2.67971	2.68132	7.93662
C	-3.59698	3.02796	8.92758
C	-4.83819	3.54654	8.56219
C	-5.15451	3.71812	7.21577
C	-3.20562	5.36256	4.15038
C	-4.32480	6.31901	4.50762
C	-4.40387	6.85324	5.79780
C	-5.38554	7.78718	6.12780
C	-6.30901	8.19905	5.16949
C	-6.23902	7.67653	3.87804
C	-5.25176	6.75001	3.55119
C	1.68425	-6.94307	-2.19608
C	1.22160	-7.83149	-3.33311
C	1.80228	-9.09119	-3.50999
C	1.33735	-9.96133	-4.49552
C	0.28573	-9.57838	-5.32546
C	-0.29942	-8.32328	-5.15969
C	0.16311	-7.45997	-4.16887

C	0.17122	4.39091	6.13733
C	-2.89367	6.45255	0.19734
C	-6.30909	4.06220	1.24588
C	-4.57328	-0.34644	5.99927
C	4.15719	-6.51781	0.81251
C	-2.37693	-6.62676	-1.43220
C	0.09385	-4.52866	-6.08939
C	5.79805	-2.10676	-2.77673
H	1.16820	1.74238	-0.13855
H	0.88387	3.16804	0.84079
H	1.74888	1.80326	1.51659
H	-1.99460	-1.82651	0.99623
H	-2.92378	1.94175	-0.17384
H	-1.25709	2.28379	-0.72157
H	-2.47735	1.72967	-1.82031
H	-0.51651	0.36336	-2.93073
H	0.80123	0.36841	-1.82083
H	0.48634	-1.01472	-2.89042
H	-4.11666	-1.28911	-1.29248
H	-3.02301	-2.37715	-2.13640
H	-3.47792	-0.84044	-2.87346
H	2.52603	-1.21358	2.41844
H	2.33696	-0.45396	0.88193
H	2.74042	-2.18886	1.03187
H	-0.93398	0.21069	4.09750
H	0.65849	-0.38824	4.39594
H	0.51006	1.02081	3.41334
H	-0.95000	-3.29870	3.83608
H	0.78133	-3.22348	4.14093
H	0.16258	-4.11036	2.74736
H	-2.67838	5.75746	3.28493
H	-2.48707	5.35017	4.97824
H	-3.68992	6.53159	6.55148
H	-5.42753	8.19137	7.13566

H	-7.07511	8.92624	5.42433
H	-6.94865	7.99800	3.12037
H	-5.19774	6.35695	2.54125
H	-4.99176	2.50023	4.40831
H	-5.53617	4.13372	4.69625
H	-2.28542	2.56740	5.82480
H	-1.70903	2.27740	8.21112
H	-3.34509	2.89677	9.97631
H	-5.55972	3.82505	9.32570
H	-6.11959	4.13473	6.93661
H	1.59905	6.11123	4.39705
H	1.39209	7.78764	2.59425
H	-0.19171	7.38120	0.73627
H	-0.38430	2.98068	4.61743
H	-0.83570	4.78282	6.30277
H	0.87999	5.20817	6.30899
H	0.36094	3.62952	6.90220
H	1.89761	2.38341	5.32457
H	2.51697	3.95190	4.78922
H	1.92352	2.79755	3.59512
H	-2.38452	4.38940	0.47481
H	-3.49057	6.56102	1.10709
H	-3.57672	6.24951	-0.63536
H	-2.41706	7.42004	0.00338
H	-0.28468	4.43353	-0.91015
H	-0.53084	6.15472	-1.21042
H	-1.70702	4.98269	-1.81907
H	-7.49216	1.35877	0.94973
H	-7.96424	-0.61369	2.36022
H	-6.27886	-1.34596	4.02146
H	-4.33556	3.28565	0.95072
H	-6.17527	4.34096	2.29310
H	-7.35510	3.76410	1.11239
H	-6.13679	4.94974	0.62585

H	-5.31377	3.41674	-1.27731
H	-6.64367	2.34613	-0.83474
H	-5.00642	1.70096	-0.94821
H	-3.08940	0.43338	4.66996
H	-5.01943	0.61305	6.27318
H	-3.83437	-0.60160	6.76667
H	-5.36019	-1.10785	6.03713
H	-2.80856	-1.65721	3.30155
H	-4.09366	-2.43109	4.22854
H	-2.58372	-1.97642	5.03178
H	0.88504	-6.87813	-1.46192
H	2.53140	-7.42886	-1.69818
H	2.62895	-9.39104	-2.87062
H	1.80032	-10.93703	-4.61578
H	-0.07752	-10.25305	-6.09566
H	-1.12261	-8.01797	-5.79987
H	-0.30062	-6.48645	-4.04095
H	3.27882	-4.51574	-3.92364
H	2.59382	-5.98910	-4.57857
H	4.08304	-7.69190	-5.00277
H	6.27031	-8.80029	-4.66607
H	7.79713	-8.01950	-2.86546
H	7.11661	-6.11986	-1.41560
H	4.93255	-5.01525	-1.76011
H	2.13777	-7.26100	2.66148
H	-0.15496	-8.15871	2.84704
H	-1.92550	-7.30264	1.34679
H	3.21926	-4.68168	0.21562
H	4.00215	-7.04544	-0.13104
H	4.11835	-7.25622	1.62032
H	5.17114	-6.10368	0.79904
H	4.43793	-4.20517	2.29997
H	3.46121	-5.37808	3.19122
H	2.70904	-3.90597	2.58382

H	-1.37195	-4.73943	-1.29608
H	-1.62121	-7.13595	-2.03736
H	-3.15107	-6.24470	-2.10730
H	-2.84652	-7.38094	-0.79086
H	-2.47279	-3.97521	0.81705
H	-3.35711	-5.50520	0.91084
H	-3.65828	-4.39190	-0.43117
H	1.23455	-2.15707	-6.89245
H	3.32442	-0.84568	-6.94379
H	4.72287	-0.69422	-4.90731
H	0.04545	-4.14010	-3.98174
H	0.94222	-5.21825	-6.08095
H	0.10791	-3.99506	-7.04548
H	-0.82607	-5.12270	-6.06879
H	-1.16478	-1.97166	-4.09882
H	-2.01989	-3.21045	-5.02235
H	-1.05337	-1.98990	-5.86352
H	3.97104	-2.50903	-1.74081
H	5.91223	-3.09859	-3.22355
H	6.38322	-2.08034	-1.85061
H	6.24469	-1.37711	-3.46041
H	3.17139	-0.11596	-1.63267
H	4.57123	0.38447	-2.56412
H	4.79550	-0.30270	-0.94616
S	4.92466	3.13347	-4.79656
S	5.84369	4.41635	-3.48013
C	3.24934	3.74234	-4.98713
C	6.65617	5.65538	-4.48866
C	7.65833	6.39253	-3.84714
C	8.31434	7.41114	-4.53309
C	7.98832	7.69184	-5.85952
C	6.99643	6.94755	-6.49509
C	6.32379	5.93337	-5.81491
H	7.92784	6.16667	-2.81857

H	9.09154	7.97917	-4.02926
H	8.50693	8.48192	-6.39459
H	6.73634	7.15643	-7.52923
H	5.55328	5.35470	-6.31553
C	2.87364	5.06886	-4.77204
C	1.55305	5.45382	-4.99692
C	0.61144	4.52969	-5.44673
C	0.99398	3.20594	-5.66008
C	2.30691	2.80452	-5.42371
H	3.60233	5.79195	-4.41848
H	1.26340	6.48633	-4.82223
H	-0.41483	4.83777	-5.62397
H	0.26680	2.47399	-6.00062
H	2.59191	1.76553	-5.56649

238

TS5 without dispersion

C	6.97363	5.39853	0.16423
C	5.59231	5.09968	-0.01816
C	5.02822	5.26082	-1.31252
C	5.85319	5.64888	-2.37434
C	7.20559	5.90492	-2.20325
C	7.74938	5.78842	-0.93060
N	4.81208	4.77354	1.12577
C	4.53268	3.58741	1.70725
N	4.84313	2.34005	1.18821
C	5.65174	2.20860	-0.04390
C	6.29371	0.85303	-0.26069
C	5.76806	-0.03255	-1.20856
C	6.38816	-1.25337	-1.47027
C	7.54955	-1.60867	-0.78735
C	8.09195	-0.73032	0.14984
C	7.47155	0.49090	0.40375
C	3.55240	5.06393	-1.62224
C	2.91409	6.37356	-2.11544

C	7.65876	5.30811	1.51872
C	8.15200	6.68048	1.99050
Y	3.59644	6.10809	2.77790
C	5.27144	6.67096	4.62386
Al	3.49632	7.06002	5.67131
C	3.66053	5.88618	7.25903
Y	1.67585	9.37826	4.73122
C	3.62629	9.02028	6.35590
Al	0.70530	7.10288	2.88748
C	-1.03305	6.15765	2.85011
N	0.63056	11.38588	5.70910
C	0.94181	12.77522	5.65058
C	1.92416	13.36225	6.49161
C	2.27239	14.70511	6.30599
C	1.69047	15.48522	5.31762
C	0.74325	14.90793	4.48308
C	0.36309	13.57013	4.61708
C	2.66284	12.59632	7.57919
C	4.15210	12.44303	7.23178
C	-0.68973	13.03927	3.65217
C	-0.27838	13.19719	2.17998
N	-0.20378	9.37155	6.20827
C	-1.26109	8.43619	6.40773
C	-2.51577	8.58255	5.75268
C	-3.51542	7.62412	5.95426
C	-3.31561	6.52309	6.76832
C	-2.08138	6.36352	7.38570
C	-1.04201	7.28460	7.22468
C	-2.86234	9.73184	4.81748
C	-3.12218	9.24226	3.38486
C	0.28464	7.02103	7.93197
C	0.38731	5.61930	8.54227
C	2.77347	11.60996	3.35551
C	1.95835	6.93541	4.41815

C	0.37913	9.12703	2.48506
N	3.85671	3.76056	2.88372
C	3.75099	2.82717	3.95381
C	4.89793	2.32217	4.63323
C	4.73269	1.39371	5.66511
C	3.47934	0.96149	6.07154
C	2.36070	1.50365	5.45645
C	2.46308	2.44131	4.42392
C	6.32535	2.74162	4.32037
C	6.99195	3.41721	5.53014
C	1.16235	2.99056	3.85919
C	0.25682	3.51616	4.98145
C	1.63564	6.43795	1.14668
C	-0.26105	10.71319	6.46834
N	-1.10022	11.28512	7.41283
C	-1.77960	10.51353	8.45590
C	-1.35473	10.79782	9.88327
C	-0.05080	11.15552	10.23092
C	0.30518	11.35349	11.56513
C	-0.63854	11.18231	12.57575
C	-1.94155	10.81428	12.24066
C	-2.29520	10.63125	10.90658
C	-1.18928	12.75449	7.57321
C	-2.45515	13.28731	8.21690
C	-2.40434	13.83833	9.50231
C	-3.53205	14.41805	10.08036
C	-4.73632	14.45429	9.38075
C	-4.80025	13.91731	8.09572
C	-3.66717	13.34753	7.51902
C	4.07784	1.14179	1.53818
C	3.02827	0.70681	0.53365
C	2.75169	-0.65793	0.38970
C	1.73959	-1.09686	-0.46018
C	0.99098	-0.17491	-1.19161

C	1.26140	1.18552	-1.06023
C	2.27018	1.62198	-0.20071
C	2.54337	13.26465	8.95812
C	-2.05128	13.71949	3.86397
C	-4.08956	10.50966	5.31195
C	0.60803	8.04827	9.02209
C	7.17772	1.55034	3.86419
C	0.40809	1.94926	3.02050
C	3.30382	3.96828	-2.67007
C	8.82246	4.30676	1.51551
H	3.08409	11.84050	2.34264
H	1.68977	11.43288	3.38103
H	3.07248	12.36093	4.08423
H	1.32356	6.25850	5.02105
H	-0.25406	9.80732	3.07991
H	1.21292	9.70667	2.06842
H	-0.25837	8.91629	1.61661
H	0.78519	6.73125	0.51773
H	2.48855	6.89923	0.62396
H	1.69569	5.34667	1.00357
H	-1.60287	6.22910	3.78342
H	-0.90335	5.09116	2.62733
H	-1.67561	6.55691	2.05255
H	5.89483	6.92490	5.49014
H	5.70379	7.27743	3.81382
H	5.52676	5.61355	4.44040
H	2.86086	9.33782	7.08581
H	4.50535	8.83108	6.98549
H	3.94569	9.89340	5.76264
H	3.29383	6.35630	8.17883
H	4.71594	5.63872	7.43754
H	3.13200	4.93273	7.14623
H	-1.09522	13.20525	6.58852
H	-0.33851	13.11545	8.15970

H	-1.47141	13.80633	10.05802
H	-3.46765	14.83947	11.07971
H	-5.61733	14.90495	9.82957
H	-5.73021	13.95261	7.53462
H	-3.72505	12.95191	6.51122
H	-1.63279	9.45408	8.25840
H	-2.85818	10.69100	8.37859
H	0.69376	11.27762	9.45130
H	1.32410	11.63861	11.81292
H	-0.36194	11.33435	13.61536
H	-2.68751	10.67917	13.01933
H	-3.31835	10.36309	10.65268
H	3.02656	15.14603	6.95265
H	1.97573	16.52632	5.19501
H	0.28687	15.50914	3.70074
H	2.22198	11.59461	7.64112
H	1.50527	13.43307	9.25676
H	3.04975	14.23561	8.97135
H	3.01971	12.64213	9.72402
H	4.65657	11.80529	7.96627
H	4.65111	13.41878	7.23962
H	4.30650	12.00637	6.24210
H	-0.80936	11.97075	3.85744
H	-2.40720	13.62350	4.89233
H	-2.80692	13.28273	3.20142
H	-1.99337	14.79020	3.63877
H	0.70077	12.75472	1.98162
H	-0.22649	14.25308	1.89221
H	-1.01472	12.71565	1.52682
H	-4.47047	7.74696	5.44989
H	-4.10403	5.79011	6.91542
H	-1.92554	5.49316	8.01173
H	-2.01480	10.42247	4.78835
H	-3.97834	10.82422	6.35134

H	-4.99569	9.89731	5.24981
H	-4.25768	11.39890	4.69392
H	-3.28202	10.09286	2.71184
H	-4.01974	8.61556	3.34217
H	-2.29159	8.64766	3.00367
H	1.06854	7.10205	7.16775
H	0.66942	9.05742	8.61507
H	1.57252	7.81476	9.48704
H	-0.15083	8.04206	9.81185
H	0.19019	4.82830	7.81330
H	-0.31013	5.49524	9.37929
H	1.39416	5.46240	8.93557
H	3.59238	1.30035	2.49937
H	4.78328	0.31780	1.68735
H	3.34099	-1.38270	0.94690
H	1.53996	-2.16075	-0.55681
H	0.20389	-0.51448	-1.85905
H	0.68337	1.91247	-1.62407
H	2.46794	2.68469	-0.09604
H	6.43905	2.95835	-0.01203
H	5.03641	2.43596	-0.92020
H	4.86244	0.23717	-1.74444
H	5.96284	-1.92512	-2.21087
H	8.03521	-2.55910	-0.99094
H	9.00634	-0.99021	0.67629
H	7.91331	1.17468	1.12113
H	5.61562	1.00531	6.16630
H	3.37532	0.22984	6.86793
H	1.37308	1.19251	5.78477
H	6.29866	3.46779	3.50288
H	6.72264	1.03604	3.01556
H	7.29684	0.81881	4.67111
H	8.18122	1.88042	3.57286
H	7.97675	3.81197	5.25500

H	7.14075	2.70265	6.34702
H	6.38908	4.23977	5.92192
H	1.41012	3.83202	3.20256
H	0.99837	1.60074	2.17012
H	-0.52447	2.37425	2.63213
H	0.14640	1.07522	3.62814
H	0.79628	4.20958	5.63142
H	-0.12149	2.69899	5.60512
H	-0.60919	4.03896	4.56928
H	5.41438	5.75891	-3.36272
H	7.82536	6.19731	-3.04663
H	8.80453	6.00177	-0.77826
H	3.05622	4.77372	-0.69084
H	3.67614	2.99029	-2.35530
H	3.78539	4.21663	-3.62204
H	2.23048	3.86598	-2.86458
H	3.12981	7.21343	-1.45129
H	1.82674	6.26500	-2.19451
H	3.29268	6.64032	-3.10843
H	6.91582	4.95801	2.24072
H	8.50653	3.31462	1.18166
H	9.24625	4.20854	2.52102
H	9.62948	4.63356	0.85033
H	7.34188	7.41455	2.02786
H	8.92470	7.07607	1.32239
H	8.58604	6.61110	2.99308
S	4.22745	8.80924	2.16332
S	5.18014	10.99365	3.61069
C	4.28138	9.33580	0.47584
C	6.79839	10.37280	3.26787
C	7.53621	9.76017	4.29682
C	8.90416	9.54717	4.15080
C	9.54773	9.91248	2.96758
C	8.81803	10.49463	1.92812

C	7.45443	10.72548	2.07322
H	7.03096	9.48891	5.21851
H	9.47019	9.09880	4.96303
H	10.61619	9.74895	2.85685
H	9.31907	10.78601	1.00882
H	6.88774	11.20608	1.28229
C	3.28778	10.17031	-0.06441
C	3.38375	10.62546	-1.37656
C	4.47082	10.26157	-2.17199
C	5.45779	9.42808	-1.64695
C	5.36783	8.96549	-0.33627
H	2.44082	10.46194	0.54594
H	2.60488	11.26876	-1.77745
H	4.54402	10.62062	-3.19474
H	6.30076	9.12249	-2.25973
H	6.13256	8.30637	0.06029

238

int7 without dispersion

C	3.52179	-1.46594	-2.99663
C	2.35335	-2.21376	-3.32018
C	1.82516	-2.12184	-4.63644
C	2.47903	-1.32290	-5.58056
C	3.62475	-0.60549	-5.26969
C	4.13107	-0.68216	-3.97945
N	1.68663	-2.92812	-2.28626
C	1.61648	-4.25304	-2.02711
N	2.35039	-5.23110	-2.67232
C	3.46720	-4.93178	-3.57748
C	4.72922	-5.72981	-3.31950
C	5.31941	-6.45811	-4.35711
C	6.52564	-7.13033	-4.16276
C	7.15602	-7.09001	-2.92074
C	6.57538	-6.36864	-1.87790
C	5.37469	-5.69123	-2.07941

C	0.55664	-2.83114	-5.08715
C	-0.52297	-1.82317	-5.51398
C	4.16504	-1.49751	-1.61818
C	4.12868	-0.11538	-0.94942
Y	0.01893	-2.27073	-0.65193
C	1.65145	-1.82444	1.29091
Al	-0.15593	-1.60280	2.32437
C	-0.12490	-2.75655	3.93374
Y	-1.83392	0.72840	1.20328
C	0.05732	0.36246	2.95812
Al	-2.97025	-1.55404	-0.44082
C	-4.74424	-2.41266	-0.23750
N	-1.91708	3.13886	1.66818
C	-1.22450	4.29503	1.20526
C	0.04857	4.67617	1.70694
C	0.68397	5.80628	1.18028
C	0.11784	6.56140	0.16306
C	-1.09790	6.15428	-0.36920
C	-1.77305	5.03174	0.11639
C	0.79736	3.89283	2.77197
C	2.05104	3.23849	2.16694
C	-3.08143	4.65407	-0.55967
C	-2.87512	4.33738	-2.04680
N	-3.42487	1.74765	2.59253
C	-4.71384	1.34187	3.03447
C	-5.91445	1.94330	2.55274
C	-7.14925	1.48265	3.01969
C	-7.25391	0.44131	3.92885
C	-6.09031	-0.18104	4.35477
C	-4.82759	0.22724	3.91566
C	-5.95990	3.06435	1.52544
C	-6.65505	2.61300	0.23030
C	-3.63521	-0.56445	4.42970
C	-3.82111	-2.06972	4.18721

C	3.82861	6.61121	-1.79380
C	-1.66376	-1.73074	1.04167
C	-3.49281	0.46602	-0.70876
N	0.71368	-4.50457	-1.03257
C	0.47734	-5.68927	-0.27334
C	1.35561	-6.13340	0.75383
C	0.97543	-7.21472	1.55698
C	-0.23210	-7.87474	1.38491
C	-1.07876	-7.45864	0.36739
C	-0.74709	-6.39025	-0.46883
C	2.70917	-5.50617	1.04969
C	2.75302	-4.90401	2.46282
C	-1.72506	-6.03185	-1.57479
C	-3.01022	-5.43817	-0.98768
C	-2.06174	-2.13674	-2.22577
C	-2.89805	3.01157	2.58781
N	-3.30530	3.98630	3.48471
C	-3.91089	3.62471	4.76827
C	-2.96525	3.57096	5.95315
C	-1.62923	3.18139	5.83101
C	-0.81140	3.07432	6.95639
C	-1.32144	3.34939	8.22344
C	-2.65442	3.73805	8.35680
C	-3.46500	3.85307	7.23018
C	-2.71675	5.34299	3.45800
C	-3.51054	6.40162	4.19511
C	-3.05547	6.89375	5.42350
C	-3.73673	7.91584	6.08282
C	-4.88828	8.46364	5.52166
C	-5.34750	7.98935	4.29322
C	-4.65971	6.97236	3.63515
C	2.03251	-6.66261	-2.60050
C	1.76368	-7.33125	-3.93450
C	2.50305	-8.45718	-4.31072

C	2.21488	-9.14139	-5.49088
C	1.18229	-8.70323	-6.31741
C	0.43911	-7.58061	-5.95362
C	0.72695	-6.90345	-4.77027
C	1.19634	4.74160	3.98776
C	-4.14600	5.74755	-0.39852
C	-6.66489	4.30960	2.08025
C	-3.36188	-0.29036	5.91552
C	3.84870	-6.52715	0.90793
C	-2.06180	-7.22882	-2.47637
C	0.81668	-3.79901	-6.25043
C	5.61267	-2.00990	-1.66832
H	3.84601	6.42029	-2.87000
H	2.90479	6.20177	-1.37849
H	3.85295	7.68742	-1.60302
H	-2.26628	-2.43300	1.64976
H	-4.22346	0.86001	0.01481
H	-2.86459	1.30778	-1.04741
H	-4.10231	0.24502	-1.59492
H	-2.93142	-1.92336	-2.86170
H	-1.25107	-1.60475	-2.74966
H	-1.89571	-3.21532	-2.37204
H	-5.39479	-1.77890	0.38012
H	-4.71246	-3.39480	0.24636
H	-5.25949	-2.54825	-1.19826
H	2.24082	-1.75339	2.21420
H	2.02078	-0.99364	0.67440
H	2.01717	-2.76550	0.84966
H	-0.70731	0.85186	3.58423
H	0.84574	0.11328	3.68098
H	0.52854	1.12440	2.32255
H	-0.73289	-2.33936	4.74584
H	0.89250	-2.85580	4.33602
H	-0.49411	-3.77239	3.74707

H	-2.61616	5.64948	2.41898
H	-1.70680	5.31709	3.88115
H	-2.15892	6.47045	5.86802
H	-3.36449	8.28413	7.03489
H	-5.42036	9.26124	6.03290
H	-6.23644	8.41973	3.84003
H	-5.01555	6.62356	2.67099
H	-4.39991	2.65723	4.66962
H	-4.70467	4.34688	4.98404
H	-1.22401	2.96102	4.84800
H	0.22592	2.77268	6.84022
H	-0.68534	3.26385	9.09998
H	-3.06218	3.95963	9.33947
H	-4.49994	4.16936	7.33995
H	1.64921	6.09923	1.58542
H	0.61954	7.44821	-0.21465
H	-1.53896	6.71880	-1.18684
H	0.13618	3.09512	3.12226
H	0.33256	5.20425	4.47354
H	1.88958	5.54262	3.71016
H	1.70266	4.11839	4.73303
H	2.54837	2.59674	2.90307
H	2.77246	4.00022	1.85082
H	1.80788	2.63147	1.28908
H	-3.45077	3.74452	-0.07778
H	-4.33869	5.97332	0.65371
H	-5.09127	5.43801	-0.85738
H	-3.83431	6.67944	-0.88308
H	-2.10907	3.57265	-2.19432
H	-2.56287	5.22688	-2.60528
H	-3.80915	3.97864	-2.49417
H	-8.05575	1.95441	2.64949
H	-8.22499	0.10871	4.28459
H	-6.15831	-1.01450	5.04808

H	-4.93521	3.34404	1.27124
H	-6.20030	4.65329	3.00701
H	-7.72000	4.10655	2.29321
H	-6.63250	5.12754	1.35252
H	-6.63426	3.41567	-0.51541
H	-7.70489	2.36018	0.41378
H	-6.17627	1.73308	-0.20672
H	-2.74854	-0.24259	3.87235
H	-3.16814	0.76855	6.10465
H	-2.49055	-0.85980	6.25795
H	-4.21835	-0.59087	6.52991
H	-4.06448	-2.27883	3.14175
H	-4.62819	-2.47731	4.80538
H	-2.90913	-2.61559	4.44255
H	1.16242	-6.79728	-1.96341
H	2.86312	-7.18619	-2.11399
H	3.31401	-8.79865	-3.67243
H	2.79983	-10.01535	-5.76455
H	0.95551	-9.23338	-7.23829
H	-0.37095	-7.23452	-6.58979
H	0.13846	-6.03509	-4.48765
H	3.70573	-3.87437	-3.49793
H	3.15232	-5.10707	-4.61271
H	4.82660	-6.50062	-5.32517
H	6.96984	-7.68887	-4.98229
H	8.09444	-7.61524	-2.76586
H	7.06128	-6.32788	-0.90682
H	4.93135	-5.12310	-1.26745
H	1.64725	-7.54258	2.34596
H	-0.50541	-8.70543	2.02954
H	-2.02123	-7.97694	0.21180
H	2.87894	-4.70273	0.32340
H	3.87192	-6.98927	-0.08138
H	3.74847	-7.32918	1.64667

H	4.81818	-6.04652	1.08142
H	3.68459	-4.34744	2.61682
H	2.71274	-5.69353	3.22124
H	1.91376	-4.23187	2.64750
H	-1.24304	-5.26963	-2.19767
H	-1.16588	-7.69091	-2.89975
H	-2.70094	-6.90789	-3.30675
H	-2.60919	-8.00260	-1.92754
H	-2.79786	-4.58747	-0.33468
H	-3.54158	-6.18406	-0.38569
H	-3.69060	-5.10071	-1.77704
H	2.06936	-1.25693	-6.58527
H	4.11268	0.00999	-6.02033
H	5.02516	-0.11948	-3.72364
H	0.16954	-3.40570	-4.23754
H	1.55627	-4.56226	-6.00003
H	1.17909	-3.26094	-7.13298
H	-0.10891	-4.31027	-6.53848
H	-0.67892	-1.04732	-4.76176
H	-1.47723	-2.33093	-5.69482
H	-0.23603	-1.31735	-6.44268
H	3.58339	-2.18890	-0.99970
H	5.68983	-2.98671	-2.15316
H	6.01938	-2.10088	-0.65446
H	6.26079	-1.31838	-2.21798
H	3.11044	0.28041	-0.89680
H	4.73786	0.60621	-1.50560
H	4.53109	-0.17028	0.06864
S	0.20008	0.58296	-0.74870
S	5.17216	5.75178	-0.94131
C	0.09275	1.48884	-2.28415
C	6.63672	6.45420	-1.65312
C	7.86183	5.96237	-1.17630
C	9.06021	6.45582	-1.67819

C	9.06228	7.44584	-2.66227
C	7.84847	7.93456	-3.13630
C	6.63943	7.44647	-2.63927
H	7.87081	5.19093	-0.41056
H	9.99917	6.06313	-1.29726
H	9.99981	7.82989	-3.05326
H	7.83319	8.70543	-3.90237
H	5.70837	7.84566	-3.02675
C	-0.93617	1.29458	-3.21385
C	-0.95333	2.01658	-4.40774
C	0.04892	2.94272	-4.68778
C	1.07131	3.14410	-3.76097
C	1.09598	2.42578	-2.56783
H	-1.72761	0.58470	-3.00095
H	-1.75897	1.85149	-5.11829
H	0.03391	3.50206	-5.61882
H	1.86079	3.86173	-3.96690
H	1.89580	2.58231	-1.85137

222

Complex 3 without dispersion

C	6.94062	6.21827	0.90250
C	5.68706	5.68615	0.48009
C	5.25161	5.94099	-0.85019
C	6.09640	6.64188	-1.71981
C	7.33731	7.11745	-1.32108
C	7.74083	6.91092	-0.00882
N	4.87630	5.04039	1.45376
C	4.74484	3.73699	1.78586
N	5.30634	2.66874	1.10484
C	6.26384	2.89294	-0.00080
C	7.14607	1.71327	-0.35531
C	6.83213	0.89961	-1.45057
C	7.67005	-0.14766	-1.82915
C	8.84447	-0.39480	-1.12086

C	9.18013	0.41962	-0.04040
C	8.34031	1.46711	0.33242
C	3.89157	5.52549	-1.39618
C	3.05759	6.75185	-1.80479
C	7.45553	6.06877	2.32659
C	7.62682	7.43310	3.01141
Y	3.35540	5.85133	3.18758
C	4.72030	5.98341	5.34460
Al	2.79591	6.16643	6.14908
C	2.70703	4.66078	7.43220
Y	1.29884	8.66231	5.10042
C	2.92319	8.03853	7.05888
Al	0.40368	6.63121	3.03125
C	-1.32226	5.70434	2.75374
N	0.85794	11.04278	5.53906
C	1.51272	12.24738	5.16540
C	2.74118	12.63216	5.76536
C	3.41307	13.76318	5.28801
C	2.91695	14.52316	4.23879
C	1.72370	14.13791	3.64088
C	1.01419	13.01336	4.07063
C	3.38723	11.86432	6.90783
C	4.74618	11.27886	6.49107
C	-0.27666	12.66925	3.34095
C	-0.02143	12.30119	1.87208
N	-0.47311	9.47608	6.43429
C	-1.70875	8.86733	6.80833
C	-2.88361	9.01793	6.02137
C	-4.02920	8.28541	6.34924
C	-4.05580	7.40265	7.41915
C	-2.91665	7.26871	8.20011
C	-1.75160	7.99130	7.92942
C	-2.98043	9.95841	4.83268
C	-3.37215	9.22651	3.54068

C	-0.57474	7.81395	8.87262
C	0.03962	6.42096	8.71296
S	3.71962	8.67301	3.54999
C	1.45488	6.25995	4.67784
C	0.12549	8.69546	2.82700
N	3.94474	3.58436	2.88178
C	3.88019	2.46981	3.76230
C	5.01944	1.98652	4.47166
C	4.87771	0.88702	5.32409
C	3.65569	0.26293	5.52577
C	2.53661	0.77203	4.88332
C	2.61420	1.86937	4.02098
C	6.41139	2.59630	4.37665
C	6.93007	3.05385	5.75101
C	1.31403	2.35222	3.39552
C	0.25103	2.62808	4.46911
C	1.58041	6.27380	1.36033
C	-0.15530	10.80636	6.39663
N	-0.78745	11.75263	7.18665
C	-1.48190	11.38297	8.42373
C	-0.70645	11.64052	9.70142
C	0.64776	11.31493	9.82389
C	1.31561	11.48785	11.03520
C	0.63646	11.98519	12.14661
C	-0.71324	12.31495	12.03543
C	-1.37512	12.14938	10.82011
C	-0.41357	13.17805	7.14037
C	-1.48826	14.12999	7.62362
C	-1.22386	14.98746	8.69582
C	-2.16899	15.92350	9.11449
C	-3.40108	16.00882	8.47019
C	-3.67807	15.15750	7.40056
C	-2.72716	14.23120	6.97877
C	4.71576	1.32984	1.14933

C	3.81753	0.94965	-0.01211
C	3.78933	-0.38336	-0.43962
C	2.91342	-0.79527	-1.44111
C	2.05350	0.12480	-2.04048
C	2.07593	1.45470	-1.62560
C	2.94944	1.86156	-0.61675
C	3.55921	12.73071	8.16403
C	-1.30212	13.81186	3.40610
C	-3.97511	11.09345	5.11460
C	-0.94785	8.06022	10.34165
C	7.41820	1.61394	3.76404
C	0.77035	1.35768	2.35992
C	3.99049	4.58366	-2.60653
C	8.76903	5.27616	2.38620
C	3.86065	9.72634	2.11865
H	0.76599	5.49532	5.08354
H	-0.52728	9.33053	3.44706
H	0.98559	9.30400	2.50733
H	-0.46686	8.57413	1.91103
H	0.78108	6.53204	0.65376
H	2.40789	6.92899	1.04247
H	1.84017	5.23862	1.08414
H	-1.93612	5.66491	3.66200
H	-1.17907	4.66886	2.42008
H	-1.92817	6.19719	1.98093
H	5.17493	6.01984	6.34346
H	5.25132	6.77352	4.79302
H	5.04246	5.00280	4.96151
H	2.06918	8.48292	7.59692
H	3.57392	7.69577	7.87459
H	3.50621	8.84724	6.59287
H	3.24068	3.79797	7.01202
H	1.69383	4.31870	7.66668
H	3.19294	4.89641	8.38910

H	-0.16244	13.43560	6.11466
H	0.49004	13.34771	7.73792
H	-0.26928	14.91747	9.21082
H	-1.94206	16.58217	9.94836
H	-4.14060	16.73487	8.79650
H	-4.63426	15.21922	6.88791
H	-2.94762	13.57836	6.14053
H	-1.75267	10.33082	8.37569
H	-2.42360	11.93890	8.46501
H	1.18392	10.92112	8.96477
H	2.36862	11.23087	11.11090
H	1.15670	12.11738	13.09120
H	-1.25119	12.70956	12.89322
H	-2.42441	12.42208	10.73508
H	4.35154	14.05084	5.75471
H	3.45411	15.40013	3.88859
H	1.32824	14.72189	2.81354
H	2.72377	11.03103	7.16483
H	2.60793	13.13664	8.51891
H	4.23191	13.57444	7.97563
H	3.99582	12.14036	8.97729
H	5.17073	10.67818	7.30358
H	5.46288	12.07570	6.26302
H	4.66524	10.64579	5.60198
H	-0.70806	11.79412	3.83548
H	-1.51103	14.13060	4.43056
H	-2.24779	13.50186	2.94778
H	-0.94920	14.69204	2.85721
H	0.71307	11.49987	1.77288
H	0.35437	13.16473	1.31145
H	-0.95148	11.97637	1.39214
H	-4.92070	8.40656	5.73908
H	-4.95345	6.83400	7.64561
H	-2.92974	6.59279	9.05103

H	-1.99498	10.40863	4.68011
H	-3.73978	11.60245	6.05243
H	-4.99703	10.70710	5.19774
H	-3.96617	11.83098	4.30408
H	-3.32887	9.90950	2.68480
H	-4.39550	8.84047	3.59896
H	-2.71243	8.37946	3.33881
H	0.17875	8.55818	8.59327
H	-1.40556	9.04131	10.49222
H	-0.05159	8.01200	10.96922
H	-1.64640	7.30176	10.71144
H	0.30138	6.21794	7.67130
H	-0.66854	5.64506	9.02623
H	0.94395	6.31343	9.32043
H	4.14517	1.22806	2.07111
H	5.53018	0.60137	1.21473
H	4.46538	-1.10348	0.01580
H	2.90671	-1.83481	-1.75787
H	1.37176	-0.19256	-2.82456
H	1.40771	2.17910	-2.08321
H	2.95275	2.89685	-0.29009
H	6.90436	3.72828	0.27498
H	5.72428	3.19511	-0.90315
H	5.92109	1.08783	-2.01134
H	7.40654	-0.76644	-2.68258
H	9.50093	-1.20866	-1.41636
H	10.10328	0.24813	0.50650
H	8.62234	2.10850	1.16148
H	5.75399	0.51669	5.84985
H	3.57360	-0.59476	6.18753
H	1.56939	0.30753	5.05400
H	6.36029	3.47561	3.72664
H	7.10354	1.27755	2.77497
H	7.53457	0.72780	4.39762

H	8.40517	2.08137	3.67146
H	7.87293	3.60088	5.63884
H	7.12588	2.19674	6.40392
H	6.21788	3.70224	6.26568
H	1.52136	3.29354	2.87442
H	1.48630	1.17235	1.55592
H	-0.15273	1.74006	1.90937
H	0.53591	0.39526	2.82930
H	0.63946	3.28078	5.25492
H	-0.08674	1.69976	4.94302
H	-0.62744	3.10682	4.02689
H	5.76460	6.82002	-2.73961
H	7.97477	7.65333	-2.01881
H	8.70032	7.30031	0.32187
H	3.35804	5.00706	-0.59332
H	4.49958	3.64525	-2.37552
H	4.52882	5.05979	-3.43299
H	2.98923	4.33071	-2.97178
H	3.00438	7.50032	-1.01187
H	2.03586	6.45103	-2.06146
H	3.48789	7.23837	-2.68744
H	6.70529	5.50530	2.88946
H	8.66634	4.28772	1.92862
H	9.08595	5.13597	3.42546
H	9.57696	5.79914	1.86260
H	6.69658	8.00928	3.00678
H	8.39299	8.03612	2.51131
H	7.93916	7.30046	4.05348
C	3.54182	11.08803	2.20592
C	3.72277	11.92438	1.10518
C	4.22552	11.42292	-0.09375
C	4.55569	10.07162	-0.18070
C	4.37951	9.22843	0.91533
H	3.16504	11.49999	3.13650

H	3.46872	12.97633	1.19851
H	4.36799	12.07924	-0.94767
H	4.96372	9.66106	-1.09978
H	4.67546	8.18655	0.83649

24

PhSSPh

S	2.46859	1.17389	-1.71047
S	3.72027	2.71701	-1.02356
C	2.05071	1.78803	-3.32652
C	5.10319	2.53047	-2.12630
C	6.11583	1.60330	-1.85868
C	7.17161	1.45741	-2.75264
C	7.22115	2.23445	-3.91155
C	6.21281	3.15793	-4.17787
C	5.15021	3.30661	-3.28872
H	6.06199	1.00114	-0.95733
H	7.95888	0.73842	-2.54547
H	8.04778	2.11779	-4.60645
H	6.24716	3.75917	-5.08160
H	4.34970	4.01064	-3.48998
C	1.01055	2.70722	-3.49939
C	0.72415	3.19412	-4.77062
C	1.47062	2.76551	-5.86992
C	2.50607	1.84972	-5.69780
C	2.80128	1.36110	-4.42658
H	0.44201	3.03686	-2.63567
H	-0.08367	3.90735	-4.90614
H	1.24303	3.14766	-6.86086
H	3.09116	1.51959	-6.55109
H	3.61476	0.65963	-4.27441

16

PhSMe

C	2.07141	4.95586	-0.83474
H	1.89336	4.08821	-1.47506

H	1.17768	5.58318	-0.83702
H	2.91208	5.54056	-1.21674
S	2.32770	4.46984	0.88635
C	3.78250	3.46414	0.80655
C	4.24104	2.92839	2.01933
C	5.37563	2.12767	2.04735
C	6.07323	1.84636	0.87184
C	5.61934	2.37755	-0.33118
C	4.48122	3.18250	-0.37124
H	3.70355	3.14293	2.93896
H	5.71722	1.72067	2.99483
H	6.95979	1.22035	0.89617
H	6.15168	2.16772	-1.25476
H	4.14960	3.58316	-1.32227

238

int6

C	4.64432	-1.91673	-3.53992
C	3.25779	-2.20050	-3.65196
C	2.62973	-2.07047	-4.91174
C	3.37446	-1.57713	-5.99066
C	4.70455	-1.20562	-5.85701
C	5.33416	-1.39614	-4.63192
N	2.55797	-2.45000	-2.44379
C	2.02870	-3.57576	-1.96169
N	2.14374	-4.82557	-2.51812
C	3.23249	-5.16132	-3.41635
C	3.80066	-6.54294	-3.17910
C	4.50876	-7.16090	-4.21313
C	5.10053	-8.40702	-4.02401
C	4.97751	-9.05947	-2.79813
C	4.26191	-8.45523	-1.76660
C	3.67853	-7.20439	-1.95334
C	1.19656	-2.47453	-5.17463
C	0.34106	-1.32587	-5.71603

C	5.40718	-2.22317	-2.26757
C	5.80232	-0.96474	-1.49683
Y	1.25867	-1.08976	-0.89979
C	2.74927	-0.42100	1.02973
Al	0.95496	-0.81766	2.05704
C	1.07936	-2.34098	3.29502
Y	-0.91881	1.46358	1.34448
C	0.77311	0.89398	3.20832
Al	-1.67878	-0.51832	-0.84849
C	-3.29593	-1.62380	-1.04605
N	-1.92726	3.54722	2.02143
C	-1.54142	4.89118	1.86998
C	-0.61212	5.49315	2.74637
C	-0.22861	6.81820	2.52724
C	-0.72632	7.55119	1.45700
C	-1.59634	6.93634	0.56403
C	-1.99885	5.61222	0.74050
C	0.03059	4.71736	3.87641
C	1.50369	4.44549	3.54500
C	-2.90344	4.96566	-0.28751
C	-2.24360	4.95387	-1.66995
N	-2.93242	1.63776	2.55716
C	-4.00328	0.73239	2.60422
C	-5.18070	0.90681	1.83324
C	-6.18145	-0.06704	1.88536
C	-6.03801	-1.21762	2.64432
C	-4.86190	-1.41018	3.36224
C	-3.83718	-0.46387	3.36019
C	-5.39963	2.08833	0.90858
C	-5.62265	1.62588	-0.53754
C	-2.56547	-0.71589	4.15619
C	-2.48359	-2.11580	4.76208
C	0.86396	2.57024	0.15773
C	-0.45923	-0.86067	0.67185

C	-2.18433	1.46668	-0.92097
N	1.30019	-3.38113	-0.82612
C	1.30589	-4.37797	0.18555
C	2.53866	-4.79626	0.75230
C	2.55020	-5.86781	1.64756
C	1.37526	-6.49253	2.04580
C	0.16415	-5.99691	1.58441
C	0.10132	-4.94090	0.67032
C	3.83337	-4.02567	0.55695
C	4.26132	-3.38879	1.88634
C	-1.26587	-4.41540	0.28593
C	-1.99789	-3.91052	1.53625
C	-0.54939	-0.57908	-2.59021
C	-2.95060	2.98832	2.68160
N	-3.88283	3.65941	3.44344
C	-4.64351	2.96753	4.47764
C	-4.17589	3.23851	5.88559
C	-2.84069	3.51381	6.17820
C	-2.43652	3.75916	7.48886
C	-3.36615	3.72326	8.52598
C	-4.70203	3.43938	8.24259
C	-5.10199	3.20486	6.93075
C	-3.83638	5.12822	3.56504
C	-5.09666	5.72706	4.13392
C	-5.13702	6.16072	5.46181
C	-6.29844	6.71654	5.99257
C	-7.43605	6.84842	5.19919
C	-7.40069	6.43667	3.86745
C	-6.23584	5.88690	3.33967
C	0.99231	-5.73498	-2.49832
C	-0.13331	-5.37208	-3.43662
C	-0.47806	-6.22657	-4.48517
C	-1.56377	-5.93919	-5.31251
C	-2.31197	-4.78242	-5.10504

C	-1.97543	-3.91963	-4.06061
C	-0.90087	-4.21908	-3.23080
C	-0.08479	5.42425	5.22957
C	-4.28651	5.61816	-0.34380
C	-6.57502	2.95497	1.36552
C	-2.36231	0.31573	5.26840
C	4.98658	-4.85753	-0.00434
C	-2.13142	-5.46020	-0.42506
C	1.11892	-3.65086	-6.15383
C	6.63943	-3.08736	-2.55875
H	1.12982	1.92286	-0.69946
H	0.58236	3.54105	-0.27670
H	1.80542	2.73342	0.69844
H	-1.09112	-1.63176	1.13999
H	-2.91484	1.88263	-0.21433
H	-1.39316	2.21461	-1.07462
H	-2.72706	1.44998	-1.87301
H	-1.43851	-0.47596	-3.22314
H	0.06007	0.29238	-2.87050
H	-0.06395	-1.47347	-3.00840
H	-3.92380	-1.55705	-0.14847
H	-3.08721	-2.68517	-1.21984
H	-3.91323	-1.27818	-1.88683
H	3.31894	-0.23308	1.94774
H	2.83352	0.53488	0.48428
H	3.37677	-1.16484	0.51064
H	-0.13488	1.03393	3.81830
H	1.53414	0.62383	3.95081
H	1.10313	1.87513	2.83893
H	1.18253	-3.30675	2.79015
H	0.16745	-2.39554	3.90091
H	1.91470	-2.24513	4.00121
H	-3.66037	5.54495	2.57585
H	-2.98864	5.43145	4.18825

H	-4.25660	6.04482	6.08664
H	-6.31400	7.04128	7.02881
H	-8.34377	7.27826	5.61306
H	-8.27801	6.55025	3.23708
H	-6.20861	5.57776	2.30049
H	-4.60573	1.89669	4.28352
H	-5.69583	3.25781	4.39612
H	-2.11529	3.54414	5.37249
H	-1.39451	3.98233	7.69701
H	-3.05309	3.91784	9.54760
H	-5.43551	3.41445	9.04353
H	-6.14888	3.01529	6.70725
H	0.47984	7.28252	3.20810
H	-0.42462	8.58411	1.30989
H	-1.96876	7.49253	-0.29268
H	-0.48178	3.75068	3.94443
H	-1.12258	5.65255	5.48682
H	0.47234	6.36674	5.23269
H	0.33311	4.79615	6.02382
H	1.96066	3.79055	4.29473
H	2.06913	5.38370	3.52107
H	1.60289	3.97789	2.56219
H	-3.03727	3.92675	0.01771
H	-4.78326	5.57261	0.62887
H	-4.92617	5.10690	-1.07143
H	-4.22153	6.67247	-0.63405
H	-1.24722	4.50627	-1.61559
H	-2.13325	5.96830	-2.06919
H	-2.84513	4.37306	-2.37753
H	-7.08467	0.07770	1.29855
H	-6.82444	-1.96619	2.66804
H	-4.74356	-2.32047	3.93808
H	-4.50202	2.71193	0.92620
H	-6.42567	3.32573	2.38004

H	-7.50919	2.38329	1.35070
H	-6.70238	3.81508	0.69915
H	-5.60131	2.48237	-1.22052
H	-6.59713	1.13862	-0.64744
H	-4.86008	0.91091	-0.85055
H	-1.72486	-0.62191	3.45200
H	-2.29221	1.32277	4.86264
H	-1.44303	0.10510	5.82566
H	-3.19780	0.29496	5.97612
H	-2.58570	-2.90314	4.01218
H	-3.25765	-2.26222	5.52408
H	-1.51641	-2.24618	5.25542
H	0.61781	-5.80456	-1.47527
H	1.36741	-6.72626	-2.75579
H	0.10568	-7.12915	-4.65032
H	-1.82108	-6.61716	-6.12121
H	-3.15843	-4.55747	-5.74741
H	-2.56084	-3.02448	-3.87369
H	-0.66569	-3.57258	-2.39317
H	4.02452	-4.42594	-3.26590
H	2.93444	-5.07249	-4.46790
H	4.59703	-6.65858	-5.17406
H	5.65064	-8.87241	-4.83692
H	5.43163	-10.03500	-2.65082
H	4.15436	-8.95649	-0.80895
H	3.12697	-6.73704	-1.14308
H	3.49882	-6.19273	2.06669
H	1.40173	-7.32675	2.74073
H	-0.76394	-6.43667	1.93809
H	3.63304	-3.21172	-0.14373
H	4.81714	-5.16019	-1.03774
H	5.14392	-5.76808	0.58260
H	5.91552	-4.28103	0.02979
H	5.11872	-2.72461	1.72947

H	4.55846	-4.15636	2.60854
H	3.44936	-2.81213	2.32998
H	-1.11258	-3.56048	-0.37742
H	-1.65751	-5.85371	-1.32561
H	-3.08968	-5.02010	-0.71977
H	-2.34473	-6.30362	0.24086
H	-1.34688	-3.27789	2.14191
H	-2.31701	-4.74865	2.16607
H	-2.88857	-3.33731	1.26297
H	2.88440	-1.47430	-6.95525
H	5.25244	-0.80093	-6.70348
H	6.38367	-1.14159	-4.52023
H	0.76398	-2.81074	-4.23108
H	1.66067	-4.52583	-5.79094
H	1.53958	-3.37559	-7.12714
H	0.07755	-3.94680	-6.30035
H	0.39522	-0.43810	-5.08678
H	-0.70549	-1.64207	-5.77683
H	0.66053	-1.03934	-6.72406
H	4.73804	-2.80226	-1.63063
H	6.36947	-4.01295	-3.07729
H	7.14399	-3.35870	-1.62672
H	7.36626	-2.55546	-3.18091
H	4.92805	-0.36825	-1.22611
H	6.46508	-0.32857	-2.09014
H	6.32353	-1.22915	-0.57015
S	2.83759	0.90966	-2.64227
S	3.82798	2.37859	-1.56040
C	1.89224	1.82017	-3.85279
C	5.45843	2.28526	-2.27986
C	6.53986	2.45988	-1.41223
C	7.83987	2.39552	-1.90741
C	8.06439	2.13510	-3.25810
C	6.98010	1.96241	-4.11826

C	5.67706	2.05078	-3.63843
H	6.36275	2.61680	-0.35223
H	8.67816	2.52424	-1.22919
H	9.07888	2.06529	-3.63859
H	7.14638	1.76170	-5.17262
H	4.83815	1.91001	-4.30979
C	0.89478	2.72290	-3.47444
C	0.15558	3.36903	-4.45965
C	0.40721	3.11394	-5.80904
C	1.40846	2.21801	-6.17594
C	2.16032	1.56847	-5.19857
H	0.71267	2.91862	-2.42359
H	-0.62102	4.06960	-4.16891
H	-0.17644	3.61835	-6.57342
H	1.60778	2.01914	-7.22467
H	2.92865	0.85155	-5.46921
238			
TS5			
C	6.87796	5.57993	0.32970
C	5.52240	5.22547	0.11996
C	4.96398	5.33563	-1.17343
C	5.78417	5.74239	-2.22883
C	7.12180	6.06310	-2.03532
C	7.65539	5.99171	-0.75339
N	4.75207	4.88213	1.24935
C	4.52316	3.70568	1.84680
N	4.88850	2.46421	1.36617
C	5.65038	2.39163	0.10850
C	6.20206	1.02630	-0.20590
C	5.64399	0.26881	-1.23926
C	6.15473	-0.98739	-1.55931
C	7.23942	-1.49998	-0.85090
C	7.82084	-0.74097	0.16401
C	7.30987	0.51563	0.47651

C	3.50179	5.05953	-1.45787
C	2.81754	6.32398	-1.99201
C	7.50830	5.49482	1.70424
C	8.00443	6.85668	2.18283
Y	3.50460	6.13870	2.85291
C	5.23057	6.62276	4.60209
Al	3.48333	6.96042	5.69103
C	3.67760	5.59733	7.10806
Y	1.93782	9.40835	4.48045
C	3.53479	8.84916	6.52352
Al	0.67033	7.03165	2.91828
C	-1.00415	5.99918	2.89191
N	0.81086	11.26649	5.45509
C	1.04513	12.65721	5.37570
C	2.00587	13.28545	6.19852
C	2.26902	14.64645	6.02059
C	1.60659	15.39316	5.05570
C	0.67835	14.76603	4.23253
C	0.39697	13.40570	4.35943
C	2.77821	12.52730	7.25950
C	4.25146	12.38575	6.86123
C	-0.61066	12.77046	3.41944
C	-0.39322	13.16310	1.95519
N	0.06790	9.24451	6.00291
C	-0.94303	8.27085	6.18058
C	-2.18780	8.37595	5.50592
C	-3.12959	7.35118	5.62891
C	-2.87555	6.21815	6.38136
C	-1.66290	6.11808	7.05038
C	-0.69158	7.12002	6.98655
C	-2.59274	9.56320	4.65283
C	-2.99355	9.14341	3.23336
C	0.57970	6.95420	7.80910
C	0.66236	5.62328	8.55631

C	2.56763	11.55812	2.83784
C	1.95914	6.92656	4.41446
C	0.29903	9.00354	2.45692
N	3.81277	3.85621	2.99578
C	3.78272	2.98357	4.10502
C	4.96835	2.52789	4.73927
C	4.87007	1.70758	5.86483
C	3.64332	1.33821	6.39611
C	2.48724	1.81958	5.79957
C	2.52826	2.64543	4.67432
C	6.36376	2.88614	4.26932
C	7.16963	3.60469	5.35845
C	1.20605	3.13963	4.12234
C	0.39985	3.85023	5.21152
C	1.62078	6.42914	1.17457
C	-0.05877	10.57611	6.22021
N	-0.92849	11.13912	7.13191
C	-1.56242	10.35441	8.18744
C	-1.07616	10.67276	9.57881
C	0.23517	11.07447	9.83128
C	0.65174	11.36982	11.12693
C	-0.24022	11.25460	12.19138
C	-1.54895	10.83809	11.94942
C	-1.96235	10.55506	10.65144
C	-0.99210	12.60335	7.33302
C	-2.21159	13.08730	8.07899
C	-2.08795	13.55160	9.39165
C	-3.19485	14.02967	10.08723
C	-4.44739	14.04986	9.47797
C	-4.58018	13.60786	8.16262
C	-3.46796	13.14040	7.46778
C	3.94312	1.35773	1.56709
C	2.78217	1.39769	0.59921
C	2.51551	0.32532	-0.25371

C	1.44137	0.37279	-1.14261
C	0.61762	1.49531	-1.18574
C	0.86698	2.56623	-0.32755
C	1.93872	2.51348	0.55612
C	2.68626	13.18911	8.63937
C	-2.04910	13.08813	3.83573
C	-3.75879	10.31382	5.30321
C	0.79615	8.08154	8.81537
C	7.10988	1.63461	3.80288
C	0.39004	1.99611	3.50988
C	3.28273	3.89962	-2.43516
C	8.62858	4.45181	1.74798
H	2.91839	11.70491	1.81968
H	1.50339	11.29925	2.81830
H	2.73570	12.44076	3.45269
H	1.17313	6.65957	5.14312
H	-0.22942	9.70859	3.11536
H	1.08292	9.54584	1.92075
H	-0.44620	8.78248	1.68452
H	0.78425	6.75691	0.54600
H	2.48739	6.88876	0.67387
H	1.67902	5.34583	0.99621
H	-1.58196	6.06698	3.81830
H	-0.80008	4.93817	2.70236
H	-1.65888	6.33995	2.07772
H	5.88743	6.83936	5.45010
H	5.65585	7.22957	3.79102
H	5.43496	5.56144	4.38663
H	2.62557	9.07526	7.09985
H	4.29296	8.64829	7.29161
H	3.89306	9.77992	6.06340
H	2.97707	4.76480	6.98437
H	3.55637	5.98451	8.12600
H	4.68125	5.15670	7.05059

H	-0.96641	13.08539	6.36065
H	-0.10373	12.94379	7.87116
H	-1.11892	13.51420	9.87999
H	-3.07769	14.37656	11.10958
H	-5.31410	14.41576	10.02102
H	-5.54903	13.63526	7.67217
H	-3.57842	12.81852	6.43917
H	-1.41145	9.29768	7.97994
H	-2.64395	10.52058	8.15357
H	0.93136	11.16488	9.00524
H	1.67336	11.69348	11.30253
H	0.08147	11.48831	13.20204
H	-2.25308	10.74873	12.77169
H	-2.99259	10.26548	10.46066
H	3.00724	15.12903	6.65510
H	1.81766	16.45211	4.93923
H	0.16706	15.34278	3.46718
H	2.34825	11.52157	7.32535
H	1.65326	13.35373	8.95616
H	3.19151	14.16021	8.64450
H	3.17667	12.56276	9.39225
H	4.79036	11.75737	7.57845
H	4.73847	13.36679	6.83463
H	4.36001	11.94436	5.86672
H	-0.48069	11.68743	3.50130
H	-2.27189	12.68180	4.82258
H	-2.76146	12.65037	3.12833
H	-2.21882	14.16985	3.86665
H	0.64853	13.02267	1.65577
H	-0.66008	14.20980	1.77352
H	-1.02678	12.54842	1.30680
H	-4.07535	7.44232	5.10241
H	-3.60963	5.42105	6.45096
H	-1.47202	5.23428	7.64505

H	-1.74340	10.24706	4.57765
H	-3.49423	10.67947	6.29495
H	-4.62611	9.65457	5.41209
H	-4.06686	11.16601	4.68916
H	-3.07947	10.02297	2.58592
H	-3.96534	8.63894	3.23251
H	-2.26843	8.45666	2.79956
H	1.42347	6.98954	7.11184
H	0.83549	9.04841	8.31905
H	1.74283	7.93624	9.34593
H	-0.00418	8.11039	9.56120
H	0.61316	4.76015	7.88918
H	-0.14118	5.53894	9.29690
H	1.61078	5.56641	9.09370
H	3.58411	1.38611	2.59637
H	4.49833	0.42468	1.44950
H	3.15904	-0.54962	-0.22442
H	1.24964	-0.46904	-1.80199
H	-0.21816	1.53308	-1.87835
H	0.22408	3.44184	-0.33957
H	2.11250	3.32788	1.24932
H	6.47673	3.09624	0.17832
H	5.02828	2.71551	-0.72928
H	4.79824	0.66808	-1.79092
H	5.70677	-1.56339	-2.36403
H	7.63842	-2.47989	-1.09637
H	8.68014	-1.12382	0.70703
H	7.77636	1.10974	1.25359
H	5.78311	1.35907	6.33957
H	3.58944	0.69625	7.27036
H	1.52106	1.55195	6.21679
H	6.27998	3.56251	3.41619
H	6.53853	1.10687	3.03793
H	7.27250	0.94500	4.63791

H	8.09211	1.89594	3.39441
H	8.12815	3.95148	4.95743
H	7.38493	2.93447	6.19688
H	6.63246	4.46772	5.75593
H	1.41871	3.86950	3.33342
H	0.95111	1.46130	2.74045
H	-0.52726	2.38233	3.05266
H	0.10155	1.27341	4.28130
H	0.98424	4.66005	5.64916
H	0.11845	3.15632	6.01061
H	-0.51845	4.27823	4.80760
H	5.35667	5.81523	-3.22517
H	7.74093	6.37057	-2.87344
H	8.69827	6.24966	-0.58788
H	3.03177	4.78622	-0.51140
H	3.60463	2.94271	-2.01707
H	3.82123	4.06213	-3.37489
H	2.21832	3.80013	-2.66670
H	3.02323	7.19263	-1.36363
H	1.73296	6.18126	-2.04102
H	3.16996	6.56314	-3.00132
H	6.72852	5.17537	2.39868
H	8.26665	3.46374	1.44984
H	9.03823	4.36997	2.75988
H	9.44934	4.72122	1.07459
H	7.19816	7.59473	2.19555
H	8.79906	7.24875	1.53908
H	8.39976	6.79178	3.20025
S	4.00518	8.81315	2.27762
S	4.60116	10.77254	3.61179
C	4.09092	9.20501	0.56288
C	6.26632	10.06885	3.69189
C	6.72789	9.51825	4.89035
C	8.08719	9.25699	5.05856

C	8.98814	9.54019	4.03426
C	8.52711	10.08528	2.83418
C	7.17278	10.34949	2.66134
H	6.02611	9.30956	5.68944
H	8.44093	8.83302	5.99444
H	10.04677	9.33944	4.16926
H	9.22725	10.31105	2.03475
H	6.80839	10.79257	1.73920
C	3.03497	9.82251	-0.11975
C	3.15507	10.08818	-1.48069
C	4.31742	9.73860	-2.16754
C	5.36174	9.10859	-1.49150
C	5.25441	8.83966	-0.13151
H	2.12569	10.08222	0.40642
H	2.33195	10.56331	-2.00610
H	4.40232	9.94307	-3.23052
H	6.25704	8.80229	-2.02091
H	6.05451	8.32767	0.39074

238

int7

C	4.00702	-1.79871	-4.02717
C	2.67876	-2.29408	-4.04068
C	1.94989	-2.30249	-5.25326
C	2.57344	-1.83558	-6.41473
C	3.87469	-1.34968	-6.40607
C	4.58168	-1.33253	-5.20914
N	2.10642	-2.61994	-2.79385
C	1.94227	-3.78077	-2.14483
N	2.28045	-5.02634	-2.63042
C	3.05268	-5.10607	-3.88323
C	3.56077	-6.48106	-4.23213
C	2.84198	-7.28494	-5.12321
C	3.30274	-8.55044	-5.47650
C	4.50369	-9.02629	-4.95321

C	5.24651	-8.22065	-4.09245
C	4.78054	-6.95571	-3.74198
C	0.51646	-2.78456	-5.35127
C	-0.40764	-1.68451	-5.88688
C	4.81077	-1.76157	-2.74397
C	5.09617	-0.32563	-2.30293
Y	1.10493	-1.28350	-1.10343
C	2.91228	-0.78193	0.65629
Al	1.17739	-0.75337	1.81001
C	1.45682	-2.17816	3.13755
Y	-0.42095	1.67560	0.81571
C	1.33644	1.14634	2.60499
Al	-1.75390	-0.52923	-0.81384
C	-3.36592	-1.65810	-0.74441
N	-1.37108	3.63335	1.78789
C	-0.93551	4.97242	1.82215
C	-0.28275	5.54746	2.93857
C	0.20597	6.85219	2.83857
C	0.06568	7.59885	1.67802
C	-0.56993	7.03147	0.57908
C	-1.06290	5.72713	0.62363
C	-0.05443	4.78398	4.22920
C	1.37632	4.24284	4.29987
C	-1.74676	5.11967	-0.58773
C	-1.50175	5.86202	-1.90036
N	-2.33873	1.68161	2.21930
C	-3.32459	0.70856	2.49715
C	-4.59175	0.69897	1.86223
C	-5.46693	-0.36254	2.11324
C	-5.12979	-1.40056	2.96526
C	-3.89510	-1.37639	3.60394
C	-2.98421	-0.34020	3.39735
C	-5.07425	1.77554	0.90666
C	-5.30791	1.21644	-0.50173

C	-1.66213	-0.33478	4.15033
C	-1.42538	-1.59392	4.98188
C	2.12068	4.99227	-0.80973
C	-0.41604	-0.73641	0.63008
C	-2.07952	1.49040	-1.15018
N	1.35857	-3.60526	-0.93200
C	1.48546	-4.47564	0.17281
C	2.75634	-4.87972	0.66054
C	2.82954	-5.72572	1.76892
C	1.69045	-6.16076	2.43092
C	0.45291	-5.71701	1.98863
C	0.32365	-4.87461	0.88256
C	4.06586	-4.41378	0.05816
C	4.94201	-3.69140	1.08915
C	-1.07338	-4.42878	0.50122
C	-1.79245	-3.78882	1.69365
C	-0.89936	-1.02805	-2.62770
C	-2.36724	3.00827	2.45682
N	-3.27630	3.63857	3.27737
C	-4.14248	2.90481	4.20130
C	-4.15693	3.42574	5.61457
C	-2.98795	3.77622	6.28827
C	-3.03615	4.24651	7.59860
C	-4.25838	4.35183	8.25958
C	-5.43048	3.98146	7.60098
C	-5.37768	3.52863	6.28658
C	-3.40134	5.10213	3.32164
C	-4.81848	5.59852	3.45203
C	-5.24275	6.21661	4.63044
C	-6.54064	6.70746	4.74654
C	-7.43112	6.58463	3.68172
C	-7.01226	5.98120	2.49657
C	-5.71152	5.49877	2.38317
C	1.38079	-6.14464	-2.33231

C	0.10533	-6.13200	-3.14574
C	-0.25706	-7.22571	-3.93444
C	-1.44675	-7.21699	-4.66268
C	-2.29390	-6.11262	-4.60554
C	-1.95015	-5.02171	-3.80716
C	-0.76325	-5.03599	-3.08317
C	-0.32853	5.62691	5.47968
C	-3.25440	4.97099	-0.36783
C	-6.38159	2.42710	1.37413
C	-1.49242	0.88157	5.06801
C	4.83393	-5.58410	-0.55563
C	-1.90864	-5.59559	-0.03825
C	0.36612	-4.03685	-6.22263
C	6.10238	-2.57740	-2.84546
H	1.97453	4.24499	-1.59162
H	1.27462	5.67857	-0.78499
H	3.03819	5.55871	-0.98466
H	-1.10794	-1.27582	1.29726
H	-2.63311	2.13157	-0.45096
H	-1.25383	2.06381	-1.59522
H	-2.76227	1.37540	-1.99913
H	-1.80245	-0.75564	-3.18646
H	-0.11119	-0.47879	-3.16643
H	-0.76628	-2.09683	-2.84616
H	-3.92430	-1.57857	0.19331
H	-3.05914	-2.70591	-0.85820
H	-4.06740	-1.44889	-1.56236
H	3.53924	-0.76836	1.55698
H	3.23803	0.10144	0.09450
H	3.27715	-1.67954	0.12970
H	0.51514	1.59853	3.18547
H	2.08205	0.89614	3.36760
H	1.82987	1.93723	2.02277
H	0.55189	-2.63024	3.54618

H	2.06254	-1.83681	3.98836
H	2.01032	-2.99165	2.65207
H	-2.97466	5.50839	2.40913
H	-2.81204	5.50061	4.15244
H	-4.55529	6.29326	5.46801
H	-6.85757	7.17797	5.67263
H	-8.44559	6.96231	3.77206
H	-7.69742	5.88886	1.65874
H	-5.38326	5.03726	1.45878
H	-3.82651	1.86574	4.21766
H	-5.16862	2.90779	3.82574
H	-2.03772	3.68787	5.77740
H	-2.11691	4.53025	8.10300
H	-4.29759	4.71971	9.28073
H	-6.38869	4.06201	8.10610
H	-6.29523	3.27817	5.76092
H	0.71768	7.29033	3.68978
H	0.45205	8.61231	1.62594
H	-0.67749	7.61346	-0.33027
H	-0.73282	3.92447	4.23005
H	-1.32502	6.07607	5.47625
H	0.40242	6.43511	5.57944
H	-0.23909	5.00695	6.37729
H	1.53129	3.67954	5.22686
H	2.09915	5.06490	4.26723
H	1.58307	3.57913	3.46309
H	-1.34354	4.10560	-0.70367
H	-3.46164	4.36965	0.51741
H	-3.72597	4.47875	-1.22464
H	-3.71906	5.95388	-0.23338
H	-0.43822	5.99808	-2.11693
H	-1.97550	6.84954	-1.89822
H	-1.94242	5.29495	-2.72625
H	-6.43092	-0.37616	1.61266

H	-5.81985	-2.22141	3.13626
H	-3.64079	-2.18351	4.27977
H	-4.30319	2.54821	0.84910
H	-6.29748	2.90768	2.34859
H	-7.18307	1.68423	1.43590
H	-6.70086	3.18960	0.65573
H	-5.50471	2.03018	-1.20862
H	-6.17679	0.54982	-0.51052
H	-4.45549	0.64186	-0.85339
H	-0.87099	-0.28393	3.39179
H	-1.52241	1.81774	4.51142
H	-0.52646	0.82964	5.58156
H	-2.27631	0.91096	5.83226
H	-1.49895	-2.50716	4.38681
H	-2.14427	-1.66227	5.80664
H	-0.42353	-1.56250	5.41697
H	1.14449	-6.12219	-1.26692
H	1.93230	-7.06934	-2.51240
H	0.39960	-8.09044	-3.97756
H	-1.71127	-8.07539	-5.27374
H	-3.22083	-6.10536	-5.17169
H	-2.60958	-4.16132	-3.73698
H	-0.51911	-4.20831	-2.42703
H	3.90082	-4.42878	-3.78241
H	2.45970	-4.74197	-4.72369
H	1.91089	-6.91354	-5.53946
H	2.72781	-9.16085	-6.16708
H	4.86657	-10.01262	-5.22735
H	6.19602	-8.57265	-3.69962
H	5.37406	-6.32578	-3.08933
H	3.80729	-6.03582	2.12750
H	1.76778	-6.81969	3.29070
H	-0.44473	-6.02784	2.51498
H	3.84041	-3.70515	-0.74251

H	4.20603	-6.12510	-1.26374
H	5.15387	-6.28882	0.21948
H	5.73257	-5.23047	-1.07372
H	5.80428	-3.22621	0.59856
H	5.32594	-4.39195	1.83795
H	4.38550	-2.91570	1.61738
H	-0.97770	-3.66896	-0.28082
H	-1.41847	-6.10226	-0.87116
H	-2.88457	-5.24057	-0.38675
H	-2.08327	-6.33623	0.75039
H	-1.16380	-3.03334	2.16538
H	-2.04283	-4.53839	2.45260
H	-2.72404	-3.31283	1.37921
H	2.01603	-1.85092	-7.34760
H	4.33315	-0.98817	-7.32193
H	5.59972	-0.95247	-5.18969
H	0.19242	-3.04502	-4.34280
H	0.86476	-4.90408	-5.78586
H	0.77614	-3.87455	-7.22515
H	-0.69075	-4.29852	-6.32232
H	-0.27245	-0.74271	-5.35338
H	-1.45410	-1.99150	-5.78741
H	-0.21822	-1.49666	-6.94985
H	4.19607	-2.22209	-1.96752
H	5.89951	-3.61349	-3.13397
H	6.62161	-2.59016	-1.88139
H	6.78778	-2.15552	-3.58838
H	4.17467	0.25520	-2.19324
H	5.73078	0.19243	-3.03131
H	5.60585	-0.31696	-1.33530
S	1.50798	1.47317	-1.33747
S	2.16099	4.23805	0.82710
C	1.08878	1.91473	-3.00153
C	3.69608	3.34900	0.79286

C	4.21869	2.92689	2.02316
C	5.39908	2.19256	2.06890
C	6.09284	1.90137	0.89525
C	5.58713	2.34484	-0.32405
C	4.38624	3.04730	-0.38489
H	3.70566	3.18351	2.94385
H	5.78732	1.86620	3.02947
H	7.02379	1.34381	0.93216
H	6.12123	2.13328	-1.24464
H	3.99163	3.34432	-1.34819
C	0.27352	3.01657	-3.29115
C	-0.02060	3.35267	-4.61052
C	0.50162	2.60355	-5.66345
C	1.33374	1.51999	-5.38423
C	1.62945	1.17830	-4.06726
H	-0.12643	3.61565	-2.48139
H	-0.65826	4.20914	-4.81170
H	0.27057	2.86707	-6.69109
H	1.75912	0.92427	-6.18545
H	2.28908	0.33811	-3.87228

222

Complex 3

C	3.02587	-0.64750	-3.12729
C	2.05143	-1.65133	-3.36527
C	1.41141	-1.72356	-4.62539
C	1.80741	-0.83387	-5.62831
C	2.77745	0.13284	-5.40915
C	3.37129	0.22659	-4.15606
N	1.68580	-2.45774	-2.26495
C	2.00358	-3.71883	-1.95713
N	2.79911	-4.56937	-2.69422
C	3.44908	-4.09225	-3.92608
C	4.61913	-4.93206	-4.36602
C	4.46814	-5.86388	-5.39722

C	5.54557	-6.63758	-5.82257
C	6.79513	-6.47887	-5.22630
C	6.96333	-5.53188	-4.21705
C	5.88338	-4.76063	-3.79629
C	0.27587	-2.68390	-4.93726
C	-1.02245	-1.91863	-5.23131
C	3.73316	-0.55860	-1.78853
C	4.11378	0.86467	-1.38405
Y	0.33129	-2.15823	-0.27625
C	1.76774	-1.94399	1.82852
Al	-0.10013	-1.56149	2.66778
C	-0.19756	-2.74581	4.24042
Y	-1.61327	0.67696	1.29166
C	0.05057	0.41450	3.27202
Al	-2.67483	-1.64540	-0.29809
C	-4.44046	-2.48768	-0.08267
N	-1.70058	3.06154	1.38536
C	-0.98152	4.10665	0.77342
C	0.28320	4.51032	1.26025
C	0.98765	5.50626	0.57930
C	0.47987	6.10179	-0.56900
C	-0.74326	5.66998	-1.06641
C	-1.47554	4.66803	-0.42893
C	0.91092	3.88993	2.49067
C	2.20796	3.15289	2.13525
C	-2.78167	4.21010	-1.04440
C	-2.59083	3.69350	-2.47140
N	-3.33650	1.82988	2.27880
C	-4.63872	1.44586	2.63857
C	-5.79503	2.03345	2.06301
C	-7.05579	1.60761	2.48727
C	-7.20933	0.60072	3.42956
C	-6.07520	-0.03052	3.92486
C	-4.79316	0.35318	3.53098

C	-5.73639	3.07508	0.96355
C	-6.32948	2.51534	-0.33538
C	-3.61078	-0.42049	4.07800
C	-3.82838	-1.93289	3.99760
C	-1.47030	-1.72435	1.26235
C	-2.99022	0.34253	-0.83129
N	1.40819	-4.11330	-0.79642
C	1.76609	-5.14699	0.09239
C	3.05752	-5.25324	0.67248
C	3.29100	-6.25019	1.62281
C	2.28864	-7.11583	2.04044
C	1.01734	-6.98548	1.49684
C	0.73613	-6.01928	0.53049
C	4.20668	-4.33576	0.30537
C	4.85632	-3.67690	1.52826
C	-0.67408	-5.92855	-0.01605
C	-1.66051	-5.47881	1.06618
C	-1.73211	-2.52232	-1.90757
C	-2.81042	3.07286	2.14291
N	-3.32030	4.16786	2.80528
C	-4.00290	3.94649	4.07644
C	-3.07770	3.70892	5.24484
C	-1.73916	3.35901	5.06805
C	-0.92331	3.08005	6.16277
C	-1.44015	3.14757	7.45374
C	-2.77622	3.50507	7.64066
C	-3.58574	3.78838	6.54479
C	-2.62143	5.46243	2.69345
C	-3.35801	6.58048	3.37704
C	-2.97362	7.00047	4.65334
C	-3.66813	8.01832	5.30256
C	-4.75507	8.62869	4.67938
C	-5.13928	8.22264	3.40178
C	-4.44165	7.20653	2.75443

C	2.49626	-6.00325	-2.68117
C	1.36081	-6.39090	-3.59997
C	1.52453	-7.39550	-4.55608
C	0.46652	-7.77015	-5.38401
C	-0.77204	-7.14411	-5.26218
C	-0.94758	-6.14597	-4.30419
C	0.11082	-5.77612	-3.48163
C	1.16755	4.91471	3.60035
C	-3.83324	5.32195	-1.00019
C	-6.43360	4.37617	1.36899
C	-3.24401	0.01976	5.49674
C	5.27496	-5.09733	-0.48371
C	-1.12368	-7.24143	-0.66307
C	0.55929	-3.61711	-6.12140
C	4.96676	-1.46795	-1.76471
H	-2.09870	-2.44674	1.81117
H	-3.69647	0.94848	-0.24184
H	-2.20098	1.00956	-1.21316
H	-3.55957	0.08979	-1.73431
H	-2.51629	-2.29778	-2.64084
H	-0.81320	-2.30080	-2.46849
H	-1.76511	-3.61500	-1.78223
H	-5.04461	-1.92458	0.64095
H	-4.37180	-3.51705	0.29024
H	-5.01480	-2.52128	-1.01770
H	2.28738	-1.63653	2.74470
H	2.32690	-1.39766	1.04916
H	1.99733	-3.01487	1.72968
H	-0.78147	0.94855	3.76077
H	0.71841	0.18594	4.11262
H	0.62265	1.13139	2.66941
H	-0.61111	-3.72965	3.98671
H	-0.80981	-2.33560	5.05225
H	0.80194	-2.92404	4.65899

H	-2.50295	5.69860	1.63750
H	-1.61546	5.38488	3.11617
H	-2.13739	6.51035	5.14403
H	-3.36133	8.33279	6.29584
H	-5.29835	9.42189	5.18477
H	-5.97999	8.70088	2.90757
H	-4.73972	6.89172	1.75902
H	-4.68203	3.10069	3.97949
H	-4.63087	4.81749	4.27600
H	-1.32987	3.29409	4.06765
H	0.11366	2.80242	5.99883
H	-0.80922	2.92520	8.30912
H	-3.18752	3.56443	8.64433
H	-4.62561	4.06855	6.69530
H	1.95433	5.82164	0.96302
H	1.03774	6.88303	-1.07702
H	-1.13498	6.10580	-1.98146
H	0.20272	3.15025	2.87235
H	0.24989	5.42095	3.91266
H	1.87520	5.68344	3.27294
H	1.59324	4.42461	4.48207
H	2.62010	2.65185	3.01830
H	2.96631	3.85165	1.76537
H	2.03750	2.40006	1.36025
H	-3.14671	3.36996	-0.44869
H	-4.04146	5.63043	0.02755
H	-4.77393	4.98583	-1.44718
H	-3.49537	6.20456	-1.55354
H	-1.84054	2.90232	-2.50774
H	-2.26295	4.48825	-3.14931
H	-3.53450	3.29178	-2.85586
H	-7.93739	2.07204	2.05384
H	-8.19890	0.29153	3.75252
H	-6.18488	-0.84766	4.63108

H	-4.69084	3.31085	0.76680
H	-5.97778	4.80630	2.26354
H	-7.49543	4.20968	1.57802
H	-6.36897	5.11514	0.56416
H	-6.22773	3.23960	-1.15037
H	-7.39464	2.28934	-0.21967
H	-5.82520	1.59221	-0.63361
H	-2.74145	-0.20326	3.44639
H	-3.02943	1.08898	5.53803
H	-2.36149	-0.52262	5.85186
H	-4.07098	-0.18669	6.18514
H	-4.14365	-2.23120	2.99442
H	-4.59037	-2.26819	4.70867
H	-2.90188	-2.45952	4.23738
H	2.26902	-6.30877	-1.65999
H	3.40549	-6.53433	-2.97160
H	2.48958	-7.88641	-4.65114
H	0.61163	-8.55108	-6.12505
H	-1.59643	-7.43421	-5.90697
H	-1.90963	-5.65375	-4.19378
H	-0.03349	-5.01176	-2.72729
H	3.78890	-3.07422	-3.75575
H	2.72341	-4.04975	-4.73990
H	3.49499	-5.98188	-5.86514
H	5.41060	-7.35917	-6.62328
H	7.63739	-7.08047	-5.55548
H	7.93910	-5.38756	-3.76227
H	6.02072	-4.01184	-3.02417
H	4.28372	-6.33738	2.05550
H	2.49437	-7.87689	2.78731
H	0.22136	-7.64792	1.82563
H	3.80971	-3.53636	-0.32536
H	4.86961	-5.53455	-1.39642
H	5.69464	-5.90596	0.12452

H	6.09829	-4.43029	-0.75997
H	5.61599	-2.95814	1.20289
H	5.35794	-4.41572	2.16179
H	4.12583	-3.14626	2.14067
H	-0.66335	-5.16394	-0.79714
H	-0.42033	-7.56503	-1.43440
H	-2.10603	-7.11924	-1.13150
H	-1.20730	-8.04177	0.08004
H	-1.35582	-4.52784	1.51183
H	-1.72755	-6.21991	1.87005
H	-2.66226	-5.34420	0.64474
H	1.32563	-0.89327	-6.60078
H	3.05736	0.82023	-6.20178
H	4.11373	0.99620	-3.97220
H	0.11198	-3.30600	-4.05511
H	1.36632	-4.32366	-5.91863
H	0.82256	-3.04978	-7.01989
H	-0.32909	-4.21388	-6.34627
H	-1.27328	-1.20830	-4.44211
H	-1.85979	-2.61688	-5.33437
H	-0.93878	-1.35897	-6.16933
H	3.03369	-0.93434	-1.03523
H	4.69588	-2.51164	-1.93726
H	5.47151	-1.41263	-0.79426
H	5.68089	-1.16857	-2.54012
H	3.26931	1.54749	-1.49450
H	4.94796	1.24882	-1.98202
H	4.43047	0.87950	-0.33640
S	0.66600	0.59632	-0.24595
C	0.49514	1.46338	-1.78587
C	0.97270	2.77531	-1.87259
C	0.93888	3.44697	-3.09190
C	0.42446	2.82573	-4.22720
C	-0.07575	1.52813	-4.13403

C	-0.04402	0.84998	-2.91991
H	1.36056	3.26821	-0.98799
H	1.30859	4.46627	-3.14148
H	0.40429	3.35038	-5.17759
H	-0.47983	1.03175	-5.00943
H	-0.42486	-0.16124	-2.84904

216

Int8

C	-3.07990	8.09403	1.84827
C	-3.58831	6.97435	1.18408
C	-4.90950	7.00628	0.72942
C	-5.71799	8.11373	0.96989
C	-5.20881	9.21485	1.65717
C	-3.88377	9.20629	2.08825
C	-2.70280	5.78595	0.91847
N	-2.96326	4.63394	1.79828
C	-3.13243	4.98076	3.21388
C	-1.85388	5.45324	3.86716
C	-1.80281	6.67045	4.54919
C	-0.62406	7.09366	5.16306
C	0.52046	6.30154	5.10202
C	0.47657	5.07942	4.43173
C	-0.70166	4.66178	3.82332
C	-2.40405	3.41006	1.49374
N	-2.57842	2.31961	2.28228
C	-3.66156	2.03680	3.13437
C	-5.01107	2.10806	2.70046
C	-6.03104	1.77217	3.59339
C	-5.76217	1.34088	4.88470
C	-4.43915	1.22106	5.28725
C	-3.38282	1.54979	4.43683
C	-5.41930	2.50956	1.29657
C	-6.21305	3.81734	1.31154
C	-1.97101	1.35762	4.95157

C	-1.72464	-0.10661	5.32697
Y	-1.05011	0.93520	1.20700
C	1.22508	1.48238	2.29328
Al	1.10802	-0.56362	2.63363
C	-0.64883	-1.19150	2.00151
Al	-2.41601	-1.71853	1.29614
C	-3.91077	-2.05429	2.53001
Y	0.37354	-1.88502	-0.15089
C	0.26157	0.50130	-0.94226
C	2.13405	-3.29710	-1.89611
N	1.14748	-3.85300	-1.15688
C	0.80277	-5.20748	-0.95480
C	-0.12576	-5.82804	-1.82412
C	-0.48357	-7.15784	-1.59525
C	0.01822	-7.86370	-0.50829
C	0.86592	-7.22173	0.38650
C	1.26005	-5.89754	0.18833
C	-0.72878	-5.07982	-2.99453
C	-2.24379	-5.26874	-3.09352
C	2.09960	-5.19776	1.23367
C	1.21543	-4.82799	2.43210
N	3.08280	-3.99857	-2.60848
C	4.36879	-3.36575	-2.89837
C	5.41435	-3.61067	-1.83818
C	5.08701	-3.60006	-0.48154
C	6.07158	-3.76308	0.48906
C	7.40117	-3.94495	0.11184
C	7.73531	-3.97054	-1.24152
C	6.74607	-3.81013	-2.20840
C	3.17674	-5.46728	-2.50322
C	4.09940	-6.06486	-3.53257
C	5.29575	-6.66320	-3.13292
C	6.16088	-7.22010	-4.07280
C	5.83952	-7.17803	-5.42726

C	4.64485	-6.58471	-5.83616
C	3.77962	-6.03671	-4.89421
N	2.12475	-1.95209	-1.80170
C	2.58929	-0.98623	-2.72027
C	3.55921	-0.03375	-2.31547
C	3.82346	1.06731	-3.13168
C	3.19153	1.23381	-4.35611
C	2.30077	0.25957	-4.78619
C	1.98543	-0.84711	-3.99475
C	4.38376	-0.20525	-1.05655
C	4.10720	0.89239	-0.02783
C	1.04026	-1.88520	-4.56198
C	-0.22591	-1.26879	-5.16558
C	5.88257	-0.26231	-1.37611
C	1.77565	-2.72500	-5.61456
C	-1.47541	-1.88397	-2.08976
O	-2.37812	-1.86341	-2.77637
C	-2.05141	-3.43719	0.23037
C	-0.05231	-5.45236	-4.31549
C	3.32600	-5.99624	1.67460
C	1.69254	-0.84655	4.49874
C	2.52177	-1.58433	1.54741
C	-3.05041	-0.19844	0.02962
N	-1.58904	3.14646	0.46152
C	-1.19279	3.95039	-0.62777
C	-1.93689	3.85689	-1.82848
C	-1.54940	4.61444	-2.93412
C	-0.43147	5.43889	-2.88543
C	0.32742	5.48100	-1.72268
C	-0.02272	4.74008	-0.59125
C	-3.14590	2.95063	-1.93338
C	-2.95588	1.89775	-3.02973
C	0.89940	4.76086	0.61017
C	1.10619	6.15870	1.19995

C	-4.43785	3.74505	-2.13997
C	2.25022	4.14193	0.22757
C	-6.23545	1.40630	0.61010
C	-1.66688	2.28935	6.12923
H	-0.74409	3.70074	3.32564
H	2.79308	4.79268	-0.46670
H	1.22051	6.09938	-1.69023
H	0.19640	0.13722	-1.97829
H	-0.22513	1.48982	-1.03548
H	1.32793	0.74544	-0.82966
H	-0.61068	-2.07890	2.66855
H	-3.57359	0.62209	0.53909
H	-2.50371	0.23767	-0.81876
H	-3.85313	-0.77608	-0.44376
H	-2.12616	-4.14905	1.06582
H	-2.94424	-3.60010	-0.38693
H	-1.22797	-3.84604	-0.37292
H	-4.20606	-1.11897	3.02316
H	-3.64538	-2.76408	3.32383
H	-4.80146	-2.45183	2.02580
H	3.40810	-0.99454	1.81009
H	2.70206	-1.79819	0.48364
H	2.63046	-2.53276	2.08651
H	0.65607	2.10056	3.00668
H	2.26315	1.57353	2.63589
H	1.24179	2.01373	1.33117
H	2.78233	-0.97113	4.55774
H	1.24368	-1.74256	4.94532
H	1.43384	-0.00284	5.14990
H	-2.84875	5.46245	-0.10993
H	-1.65933	6.10048	1.00827
H	-2.04756	8.08614	2.18516
H	-3.47340	10.06714	2.60842
H	-5.83834	10.08000	1.84402

H	-6.74356	8.12085	0.61215
H	-5.30340	6.15897	0.17940
H	-3.52930	4.11575	3.74517
H	-3.89244	5.76278	3.27376
H	1.35856	4.44697	4.38349
H	1.43945	6.63192	5.57730
H	-0.60035	8.04521	5.68670
H	-2.69224	7.29350	4.59119
H	-0.14445	6.02900	-3.75080
H	-2.13459	4.55459	-3.84814
H	0.44282	4.13982	1.38528
H	0.17775	6.57590	1.59822
H	1.49947	6.85379	0.45073
H	1.81842	6.11740	2.02931
H	2.87784	3.99318	1.11237
H	2.11963	3.17757	-0.27093
H	-3.23419	2.42051	-0.98270
H	-4.59536	4.45491	-1.32280
H	-5.30357	3.07547	-2.17539
H	-4.41260	4.31417	-3.07550
H	-2.02037	1.34901	-2.88300
H	-2.91507	2.35535	-4.02405
H	-3.78254	1.17962	-3.02260
H	-7.06249	1.83829	3.25815
H	-6.57183	1.08467	5.56148
H	-4.21441	0.86019	6.28689
H	-4.51508	2.67214	0.70400
H	-5.64283	4.61560	1.78861
H	-7.15111	3.69536	1.86346
H	-6.46795	4.12885	0.29259
H	-6.39505	1.65106	-0.44555
H	-7.22017	1.29695	1.07602
H	-5.73425	0.43805	0.66879
H	-1.28234	1.61790	4.14051

H	-1.84904	3.33539	5.87082
H	-0.61913	2.19230	6.43362
H	-2.29055	2.03991	6.99468
H	-1.86664	-0.75617	4.46021
H	-2.40764	-0.42852	6.12005
H	-0.70261	-0.24358	5.68797
H	2.18422	-5.89258	-2.62512
H	3.52595	-5.74905	-1.50358
H	5.55704	-6.67400	-2.07873
H	7.08900	-7.67957	-3.74558
H	6.51445	-7.60678	-6.16232
H	4.38543	-6.55230	-6.89044
H	2.85260	-5.57455	-5.21787
H	4.21673	-2.29607	-3.03205
H	4.72739	-3.74740	-3.85711
H	7.00413	-3.84933	-3.26357
H	8.76710	-4.12293	-1.54506
H	8.17093	-4.07205	0.86734
H	5.79676	-3.74506	1.53936
H	4.05653	-3.45416	-0.17813
H	1.22563	-7.75668	1.26089
H	-0.26852	-8.89859	-0.34670
H	-1.17438	-7.64533	-2.27706
H	2.45999	-4.26839	0.78637
H	3.97031	-6.23851	0.82442
H	3.04781	-6.93316	2.16789
H	3.91749	-5.41445	2.38875
H	1.78006	-4.28127	3.19428
H	0.79817	-5.72840	2.89521
H	0.36896	-4.20237	2.12702
H	-0.52777	-4.01840	-2.81637
H	1.00118	-5.16913	-4.30678
H	-0.53058	-4.93611	-5.15437
H	-0.11689	-6.53054	-4.49717

H	-2.73279	-5.06478	-2.13785
H	-2.50398	-6.28922	-3.39309
H	-2.66021	-4.59361	-3.84829
H	1.82765	0.36245	-5.75859
H	3.40490	2.09915	-4.97639
H	4.55337	1.80108	-2.80067
H	0.73644	-2.54982	-3.74691
H	2.62958	-3.24301	-5.17222
H	2.14970	-2.08083	-6.41741
H	1.11179	-3.47082	-6.06210
H	-0.68663	-0.54423	-4.48900
H	-0.96076	-2.04811	-5.39292
H	-0.00841	-0.74596	-6.10231
H	4.11019	-1.16582	-0.61847
H	6.10143	-0.99569	-2.15528
H	6.44742	-0.55027	-0.48377
H	6.25656	0.71005	-1.71447
H	3.06474	0.88712	0.29478
H	4.32712	1.88161	-0.44213
H	4.73350	0.75669	0.86088

216

TS6

C	-2.92992	8.17467	2.58804
C	-3.47093	7.33889	1.60683
C	-4.75338	7.60681	1.12015
C	-5.49070	8.67733	1.61901
C	-4.94831	9.49793	2.60729
C	-3.66422	9.24726	3.08786
C	-2.68172	6.16881	1.08805
N	-3.06120	4.89260	1.72409
C	-3.37396	4.99244	3.14713
C	-2.17515	5.24447	4.02776
C	-2.36147	5.78687	5.30242
C	-1.28250	5.95366	6.16627

C	0.00269	5.58985	5.76319
C	0.19835	5.06393	4.48853
C	-0.88506	4.89429	3.62989
C	-2.52808	3.71715	1.24977
N	-2.74665	2.52286	1.85772
C	-3.84248	2.13648	2.65461
C	-5.18639	2.33857	2.24399
C	-6.22204	1.92741	3.08588
C	-5.97457	1.30355	4.30012
C	-4.65944	1.05343	4.66708
C	-3.58651	1.43936	3.86218
C	-5.57519	2.97103	0.92184
C	-6.33252	4.28567	1.13282
C	-2.18914	1.08228	4.32595
C	-2.07325	-0.42247	4.58394
Y	-1.19876	1.32169	0.68666
C	0.96078	1.80501	1.95832
Al	0.91345	-0.28565	2.04964
C	-0.80338	-0.88293	1.25170
Al	-2.53918	-1.30946	0.38777
C	-4.04624	-1.88492	1.51028
Y	0.26393	-1.73463	-0.82441
C	0.40734	1.03714	-1.50947
C	2.18551	-3.34606	-2.11178
N	1.19348	-3.79406	-1.31439
C	0.89552	-5.09097	-0.83724
C	-0.05038	-5.88345	-1.53174
C	-0.38025	-7.13989	-1.01875
C	0.17526	-7.60766	0.16645
C	1.06403	-6.80169	0.86697
C	1.43063	-5.54255	0.38854
C	-0.67082	-5.42126	-2.83562
C	-2.16403	-5.73962	-2.93636
C	2.34201	-4.66292	1.21568

C	1.56807	-4.09515	2.41057
N	3.15741	-4.12288	-2.69553
C	4.41050	-3.48982	-3.10635
C	5.45833	-3.47969	-2.01969
C	5.13847	-3.09359	-0.71599
C	6.11939	-3.03835	0.26921
C	7.43749	-3.37569	-0.03660
C	7.76381	-3.77170	-1.33240
C	6.77773	-3.82687	-2.31543
C	3.31409	-5.54218	-2.33694
C	4.21343	-6.28562	-3.28929
C	5.41605	-6.82597	-2.83074
C	6.26014	-7.51951	-3.69623
C	5.91157	-7.67263	-5.03583
C	4.71168	-7.13561	-5.50320
C	3.86735	-6.45117	-4.63455
N	2.15705	-2.00174	-2.24008
C	2.46239	-1.21161	-3.36785
C	3.33599	-0.10362	-3.21744
C	3.41518	0.84638	-4.23815
C	2.69324	0.70583	-5.41688
C	1.91811	-0.43279	-5.59904
C	1.79205	-1.40239	-4.60286
C	4.26607	0.02557	-2.02633
C	4.05216	1.30891	-1.22195
C	0.99874	-2.65706	-4.91152
C	-0.42412	-2.38437	-5.40557
C	5.73071	-0.07201	-2.47379
C	1.77833	-3.49890	-5.93000
C	-0.51772	-0.26165	-2.51130
O	-1.07455	0.27348	-3.40746
C	-2.12078	-2.76847	-1.01847
C	0.07840	-6.01011	-4.03412
C	3.61736	-5.36970	1.67696

C	1.37580	-0.69874	3.92603
C	2.46909	-0.94046	0.92167
C	-3.14029	0.36240	-0.68106
N	-1.67304	3.59993	0.21600
C	-1.31000	4.51753	-0.79645
C	-2.13080	4.62352	-1.94560
C	-1.76101	5.49459	-2.97180
C	-0.58941	6.23766	-2.90367
C	0.24088	6.08564	-1.80107
C	-0.08974	5.22946	-0.74842
C	-3.40812	3.82344	-2.09026
C	-3.40009	2.96252	-3.35651
C	0.90145	5.06019	0.38291
C	1.22097	6.36820	1.11174
C	-4.63721	4.73507	-2.05062
C	2.18845	4.41095	-0.14084
C	-6.41041	2.00438	0.07225
C	-1.77186	1.89213	5.55715
H	-0.72451	4.48250	2.64154
H	2.71518	5.08430	-0.82526
H	1.17400	6.64068	-1.75382
H	1.15369	1.21731	-2.28743
H	-0.22056	1.94400	-1.51705
H	0.99797	0.95697	-0.58139
H	-0.84608	-1.79086	1.89080
H	-3.73554	1.09349	-0.11934
H	-2.58208	0.90546	-1.45724
H	-3.87397	-0.19819	-1.27388
H	-1.67355	-3.69625	-0.62615
H	-3.17047	-3.03480	-1.19537
H	-1.74222	-2.61078	-2.04213
H	-4.36245	-1.06579	2.16862
H	-3.79118	-2.73538	2.15524
H	-4.92259	-2.18169	0.91911

H	3.23192	-0.17598	1.12218
H	2.57453	-1.10496	-0.15991
H	2.83182	-1.85608	1.40278
H	0.29680	2.37359	2.63039
H	1.92471	1.81419	2.48221
H	1.15363	2.42442	1.07304
H	2.46759	-0.75222	4.04162
H	0.97283	-1.65496	4.27864
H	1.02289	0.07455	4.61933
H	-2.83689	6.07709	0.01494
H	-1.61575	6.35942	1.24291
H	-1.93526	7.96780	2.97291
H	-3.23349	9.88642	3.85311
H	-5.52248	10.33373	2.99647
H	-6.48651	8.87432	1.23270
H	-5.17401	6.97134	0.34695
H	-3.87099	4.07989	3.47279
H	-4.10141	5.79671	3.27451
H	1.19266	4.77787	4.15819
H	0.84383	5.71861	6.43779
H	-1.44394	6.37131	7.15597
H	-3.36095	6.07589	5.61826
H	-0.31790	6.91396	-3.70877
H	-2.40429	5.58473	-3.84286
H	0.45197	4.37559	1.10569
H	0.32879	6.81754	1.55602
H	1.66310	7.10448	0.43278
H	1.93676	6.18820	1.92034
H	2.86885	4.16659	0.68178
H	1.97160	3.49273	-0.69425
H	-3.46555	3.14442	-1.23661
H	-4.68237	5.29372	-1.11202
H	-5.55844	4.15062	-2.13906
H	-4.61772	5.45923	-2.87199

H	-2.51301	2.32563	-3.40456
H	-3.41638	3.57990	-4.26125
H	-4.28394	2.31626	-3.37919
H	-7.24853	2.09626	2.77246
H	-6.79538	0.99880	4.94246
H	-4.45420	0.54148	5.60242
H	-4.66410	3.19622	0.36396
H	-5.72600	5.00956	1.68009
H	-7.25346	4.11812	1.70092
H	-6.61294	4.72982	0.17255
H	-6.58371	2.42248	-0.92484
H	-7.38839	1.82056	0.52873
H	-5.91240	1.03872	-0.04162
H	-1.48983	1.33541	3.52241
H	-1.82751	2.96611	5.36847
H	-0.74342	1.64748	5.84325
H	-2.42062	1.66289	6.40999
H	-2.33304	-0.98794	3.68683
H	-2.73592	-0.73790	5.39633
H	-1.05198	-0.68259	4.86647
H	2.33356	-6.01173	-2.32645
H	3.72183	-5.62591	-1.32344
H	5.69925	-6.68428	-1.79166
H	7.19320	-7.93248	-3.32395
H	6.57009	-8.20759	-5.71395
H	4.43209	-7.25238	-6.54631
H	2.93921	-6.02910	-5.00474
H	4.19949	-2.47500	-3.44025
H	4.79236	-4.02900	-3.97592
H	7.02859	-4.15394	-3.32111
H	8.78624	-4.04475	-1.57763
H	8.20443	-3.33393	0.73132
H	5.85173	-2.72695	1.27468
H	4.11919	-2.81675	-0.47241

H	1.47905	-7.15233	1.80773
H	-0.09435	-8.58803	0.54815
H	-1.08839	-7.76172	-1.55801
H	2.64209	-3.82413	0.58312
H	4.18968	-5.76036	0.83080
H	3.39798	-6.20411	2.35092
H	4.26169	-4.67021	2.21814
H	2.19130	-3.42124	3.00532
H	1.21608	-4.90150	3.06278
H	0.68692	-3.53289	2.08321
H	-0.55706	-4.33418	-2.87784
H	1.10941	-5.65481	-4.06342
H	-0.40210	-5.71720	-4.97326
H	0.09482	-7.10425	-3.98554
H	-2.70602	-5.40413	-2.04912
H	-2.34353	-6.81296	-3.06015
H	-2.59266	-5.23651	-3.80914
H	1.38496	-0.57158	-6.53546
H	2.75662	1.45914	-6.19641
H	4.07149	1.70304	-4.10871
H	0.91076	-3.24219	-3.99417
H	2.77226	-3.75062	-5.55066
H	1.90829	-2.94681	-6.86683
H	1.24899	-4.42869	-6.16049
H	-0.98192	-1.76551	-4.70083
H	-0.95883	-3.33058	-5.54302
H	-0.42354	-1.86671	-6.37064
H	4.06893	-0.82327	-1.36983
H	5.91074	-0.96488	-3.07703
H	6.39094	-0.12381	-1.60208
H	6.02062	0.79932	-3.07120
H	3.04617	1.36185	-0.80360
H	4.20217	2.19890	-1.84253
H	4.76265	1.36045	-0.38995

216

Int9

C	-2.88264	7.96948	3.16883
C	-3.43374	7.16940	2.16450
C	-4.71875	7.46021	1.69739
C	-5.44918	8.51390	2.23937
C	-4.89651	9.29781	3.25181
C	-3.60917	9.02711	3.71100
C	-2.64998	6.01846	1.59470
N	-3.08998	4.71113	2.11069
C	-3.46047	4.70584	3.52349
C	-2.32835	5.08158	4.44849
C	-2.60722	5.69237	5.67328
C	-1.57977	6.01402	6.55562
C	-0.25453	5.73680	6.21966
C	0.03218	5.13353	4.99716
C	-0.99968	4.80846	4.11989
C	-2.54108	3.57122	1.56175
N	-2.82136	2.32387	2.00661
C	-3.97439	1.87843	2.67683
C	-5.28978	2.20229	2.25162
C	-6.37924	1.70618	2.97304
C	-6.21544	0.87204	4.06785
C	-4.92924	0.49297	4.43252
C	-3.80762	0.96494	3.75190
C	-5.59767	3.02535	1.01589
C	-6.39242	4.28787	1.35819
C	-2.43118	0.51513	4.19472
C	-2.41477	-0.91776	4.72664
Y	-1.20571	1.21153	0.70503
C	0.79679	1.56826	2.26514
Al	0.77145	-0.50916	2.30699
C	-0.80005	-1.21162	1.34076
Al	-2.24720	-1.56180	0.04300

C	-3.67576	-2.78609	0.61102
Y	0.69974	-1.77490	-0.41786
C	0.56145	2.19813	-2.56720
C	2.34402	-3.58309	-1.85179
N	1.46127	-3.93895	-0.89515
C	1.21336	-5.19427	-0.28763
C	0.20281	-6.03270	-0.81299
C	-0.10199	-7.22542	-0.15440
C	0.56050	-7.59872	1.00837
C	1.54353	-6.76445	1.52636
C	1.88311	-5.56489	0.89822
C	-0.54652	-5.67978	-2.08034
C	-2.03261	-5.44277	-1.79534
C	2.91852	-4.66080	1.52771
C	2.31160	-3.95191	2.74123
N	3.23530	-4.42283	-2.47751
C	4.46497	-3.85927	-3.03579
C	5.59142	-3.77359	-2.03382
C	5.38324	-3.21990	-0.76800
C	6.43150	-3.10085	0.13821
C	7.70930	-3.53778	-0.21014
C	7.92712	-4.09311	-1.46931
C	6.87248	-4.21249	-2.37315
C	3.37164	-5.82675	-2.05718
C	4.12229	-6.66684	-3.05564
C	5.38898	-7.16504	-2.74431
C	6.09022	-7.94569	-3.66161
C	5.53015	-8.23449	-4.90399
C	4.26146	-7.74831	-5.22013
C	3.56176	-6.97460	-4.29914
N	2.29944	-2.25633	-2.10040
C	2.55671	-1.56540	-3.29639
C	3.43302	-0.45014	-3.26697
C	3.56679	0.34891	-4.40367

C	2.87415	0.06762	-5.57485
C	2.03728	-1.04118	-5.61024
C	1.85705	-1.86190	-4.49505
C	4.26817	-0.12759	-2.04480
C	3.92363	1.24525	-1.45930
C	0.93677	-3.05831	-4.61781
C	-0.44734	-2.69515	-5.16279
C	5.76716	-0.21713	-2.35196
C	1.58244	-4.14402	-5.48384
C	0.03457	1.55951	-1.33544
O	0.45542	0.39408	-1.05841
C	-1.41176	-2.05136	-1.81007
C	-0.36120	-6.73737	-3.17273
C	4.21461	-5.38356	1.89669
C	1.14847	-1.02606	4.17449
C	2.49206	-0.85558	1.16873
C	-3.11957	0.21075	-0.62240
N	-1.61285	3.55041	0.59467
C	-1.18905	4.48568	-0.36313
C	-1.95298	4.66116	-1.54522
C	-1.48149	5.50682	-2.55011
C	-0.26156	6.16230	-2.43457
C	0.50315	5.95748	-1.29314
C	0.06744	5.12712	-0.25742
C	-3.27864	3.95611	-1.74673
C	-3.31657	3.13584	-3.03877
C	0.98108	4.91485	0.93122
C	1.27350	6.21354	1.68894
C	-4.43927	4.95490	-1.70982
C	2.29333	4.25086	0.49702
C	-6.34969	2.18557	-0.02472
C	-1.83623	1.49388	5.20864
H	-0.77088	4.33910	3.16958
H	2.87990	4.91805	-0.14366

H	1.46608	6.45298	-1.19933
H	1.26904	1.57528	-3.12421
H	-0.29359	2.47935	-3.19275
H	1.00778	3.15962	-2.28294
H	-1.30113	-1.67972	2.20577
H	-3.69372	0.82371	0.08782
H	-2.63937	0.89039	-1.34893
H	-3.88447	-0.29806	-1.22068
H	-0.83442	-2.95633	-2.04812
H	-2.38663	-2.25806	-2.26704
H	-0.99859	-1.22649	-2.41036
H	-4.28146	-2.31696	1.39752
H	-3.27696	-3.71806	1.02962
H	-4.36178	-3.06014	-0.20056
H	3.15018	-0.46994	1.95681
H	2.72603	-0.22813	0.29925
H	2.89916	-1.86048	0.97340
H	0.02932	2.13912	2.81367
H	1.68668	1.65565	2.89995
H	1.07071	2.14035	1.36623
H	2.23114	-1.02549	4.36071
H	0.78391	-2.02436	4.43919
H	0.71034	-0.32008	4.88943
H	-2.75494	6.01105	0.51083
H	-1.58752	6.16899	1.80888
H	-1.88531	7.74887	3.53753
H	-3.16921	9.63729	4.49443
H	-5.46514	10.12066	3.67539
H	-6.44752	8.72668	1.86806
H	-5.14806	6.85425	0.90621
H	-3.83864	3.71800	3.78680
H	-4.29078	5.40286	3.66505
H	1.05911	4.91184	4.72244
H	0.54794	5.99224	6.90537

H	-1.81224	6.49012	7.50400
H	-3.63728	5.92915	5.92781
H	0.09098	6.81734	-3.22572
H	-2.08193	5.64754	-3.44521
H	0.46917	4.22937	1.61088
H	0.36028	6.67786	2.07022
H	1.77344	6.94360	1.04382
H	1.92948	6.02011	2.54388
H	2.90720	3.99392	1.36666
H	2.11053	3.33400	-0.07006
H	-3.40418	3.25320	-0.91944
H	-4.47766	5.47968	-0.75208
H	-5.39809	4.44809	-1.85422
H	-4.33241	5.70730	-2.49841
H	-2.49855	2.41110	-3.07057
H	-3.23607	3.77113	-3.92717
H	-4.25825	2.58159	-3.10747
H	-7.38264	1.97152	2.65105
H	-7.07691	0.50134	4.61540
H	-4.79244	-0.18881	5.26503
H	-4.65618	3.34007	0.56646
H	-5.85592	4.91150	2.07543
H	-7.36589	4.03671	1.79210
H	-6.57861	4.88243	0.45768
H	-6.53406	2.77023	-0.93215
H	-7.32033	1.85613	0.35988
H	-5.78243	1.29431	-0.30288
H	-1.78256	0.52049	3.31090
H	-1.78169	2.50326	4.79850
H	-0.82488	1.19154	5.49906
H	-2.45442	1.53107	6.11260
H	-2.91300	-1.60235	4.03356
H	-2.90935	-0.99796	5.70066
H	-1.38484	-1.25396	4.85871

H	2.37723	-6.24276	-1.91799
H	3.88142	-5.87938	-1.08842
H	5.83191	-6.92468	-1.78214
H	7.07536	-8.32493	-3.40563
H	6.07629	-8.83986	-5.62157
H	3.81443	-7.97695	-6.18332
H	2.57584	-6.59629	-4.54666
H	4.25053	-2.87297	-3.44534
H	4.76981	-4.48621	-3.87684
H	7.03916	-4.66136	-3.34886
H	8.91811	-4.44041	-1.74752
H	8.52940	-3.44633	0.49597
H	6.25020	-2.66105	1.11465
H	4.39644	-2.85911	-0.50225
H	2.05730	-7.04632	2.44108
H	0.31000	-8.52934	1.50902
H	-0.87422	-7.87152	-0.56318
H	3.17315	-3.90175	0.78367
H	4.66585	-5.86887	1.02633
H	4.04866	-6.14809	2.66246
H	4.94442	-4.67197	2.29434
H	3.00318	-3.21576	3.16196
H	2.06257	-4.67441	3.52588
H	1.38521	-3.43230	2.47509
H	-0.11916	-4.74681	-2.45558
H	0.69674	-6.91535	-3.38453
H	-0.84601	-6.41719	-4.10119
H	-0.80359	-7.69568	-2.88095
H	-2.17419	-4.68745	-1.01873
H	-2.51703	-6.36403	-1.45449
H	-2.55102	-5.10540	-2.69944
H	1.50036	-1.27546	-6.52553
H	2.99183	0.69767	-6.45154
H	4.23838	1.20285	-4.36908

H	0.79260	-3.47140	-3.61700
H	2.56440	-4.42104	-5.09657
H	1.71211	-3.79084	-6.51281
H	0.95482	-5.04159	-5.51317
H	-0.90351	-1.88703	-4.58716
H	-1.11188	-3.56453	-5.11256
H	-0.39737	-2.38026	-6.21039
H	4.03638	-0.88585	-1.29319
H	6.03169	-1.18759	-2.77827
H	6.35308	-0.08690	-1.43628
H	6.07287	0.55933	-3.06184
H	2.86479	1.30913	-1.20033
H	4.13794	2.04739	-2.17393
H	4.50937	1.43878	-0.55427

216

Int8 without disperion

C	5.25934	-7.11691	-3.15990
C	4.21030	-6.27651	-3.54599
C	3.92185	-6.15735	-4.91130
C	4.66600	-6.85177	-5.86231
C	5.70718	-7.68953	-5.46324
C	5.99938	-7.82259	-4.10771
C	3.37163	-5.59474	-2.48410
N	3.31466	-4.12472	-2.58376
C	4.61917	-3.52416	-2.87948
C	5.71689	-3.84878	-1.88359
C	5.50256	-3.80263	-0.50236
C	6.54818	-4.03767	0.38926
C	7.82811	-4.31823	-0.08761
C	8.05265	-4.36854	-1.46227
C	7.00272	-4.14203	-2.35040
C	2.30249	-3.38854	-1.98454
N	2.25596	-2.03286	-1.99734
C	2.77776	-1.13804	-2.97615

C	3.81075	-0.21123	-2.64723
C	4.19491	0.75861	-3.57808
C	3.62268	0.83605	-4.83873
C	2.63431	-0.07728	-5.17278
C	2.18856	-1.04808	-4.27031
C	4.56446	-0.23553	-1.32686
C	6.08868	-0.27937	-1.51762
C	1.08684	-1.97950	-4.74472
C	1.64344	-3.02367	-5.72171
Y	0.43878	-1.89474	-0.29911
C	-1.36115	-1.73595	-2.27978
O	-2.24294	-1.61990	-2.98386
N	1.27749	-3.92048	-1.26607
C	0.91409	-5.26925	-0.97809
C	0.06878	-5.99254	-1.86950
C	-0.40800	-7.25014	-1.49081
C	-0.08660	-7.81837	-0.26530
C	0.73772	-7.11511	0.60060
C	1.24493	-5.85346	0.27205
C	-0.32638	-5.47775	-3.24757
C	0.14472	-6.42066	-4.36715
C	2.12011	-5.15501	1.29856
C	3.34680	-5.98906	1.69376
Al	1.07550	-0.54165	2.52114
C	1.64535	-0.93925	4.38154
C	2.57699	-1.40290	1.40072
Al	-2.40996	-1.82225	1.15489
C	-2.05106	-3.43762	-0.06820
C	-0.65136	-1.22087	1.83531
Y	-1.18792	0.95713	1.14456
C	-3.24186	-0.24378	0.06669
N	-1.67283	3.28193	0.51838
C	-1.28633	4.12500	-0.56022
C	-2.06378	4.10029	-1.75359

C	-1.68630	4.89137	-2.84176
C	-0.54829	5.68556	-2.80493
C	0.24155	5.66398	-1.66459
C	-0.09161	4.89409	-0.54489
C	-3.31164	3.24252	-1.90066
C	-4.58173	4.09391	-2.03283
C	0.89178	4.89115	0.61531
C	2.23025	4.27407	0.17809
N	-2.67549	2.39070	2.32085
C	-3.75310	2.11276	3.20569
C	-5.11851	2.16330	2.79545
C	-6.11963	1.80090	3.70222
C	-5.83214	1.36955	4.98857
C	-4.50385	1.29753	5.38067
C	-3.46054	1.65809	4.52364
C	-5.58491	2.60081	1.41407
C	-6.44243	1.52806	0.72166
C	-2.04734	1.55239	5.07294
C	-1.83434	2.46294	6.29180
C	0.12928	0.53944	-1.01785
C	1.09798	1.53414	2.32670
C	-2.45613	3.52400	1.59365
N	-2.95082	4.76162	1.97745
C	-3.26430	5.04622	3.37981
C	-2.15360	5.68144	4.19525
C	-2.48079	6.63181	5.16971
C	-1.50127	7.17248	6.00029
C	-0.17145	6.77660	5.86293
C	0.16661	5.83477	4.89271
C	-0.81788	5.29024	4.06878
C	-2.72656	5.96564	1.14929
C	-3.65827	7.12714	1.42995
C	-4.95195	7.16749	0.89661
C	-5.78139	8.26625	1.11008

C	-5.32449	9.35376	1.85349
C	-4.03222	9.33578	2.37447
C	-3.20755	8.23225	2.16143
C	1.15277	6.28563	1.20288
C	-6.38309	3.90990	1.49063
C	-3.79030	-2.42702	2.43552
C	-3.20425	2.27698	-3.09014
C	-1.70148	0.09921	5.42597
C	-1.84364	-5.27249	-3.37712
C	1.31142	-4.77948	2.54945
C	4.18870	0.96619	-0.45095
C	-0.08442	-1.23064	-5.39764
H	-0.54605	4.54843	3.32409
H	2.73835	4.91962	-0.54690
H	1.15104	6.25896	-1.63947
H	0.73046	0.19464	-1.87508
H	-0.71153	1.05374	-1.51421
H	0.76482	1.35079	-0.62123
H	-0.65297	-2.03181	2.59484
H	-3.76209	0.52943	0.65094
H	-2.82294	0.24198	-0.82769
H	-4.06016	-0.85610	-0.33562
H	-2.14112	-4.22226	0.69770
H	-2.94114	-3.53659	-0.70463
H	-1.22310	-3.79813	-0.69673
H	-4.15100	-1.60474	3.06582
H	-3.40517	-3.20145	3.11251
H	-4.66788	-2.85631	1.93288
H	3.39717	-0.72784	1.67913
H	2.78638	-1.58709	0.33564
H	2.78293	-2.34346	1.92833
H	0.46249	2.10144	3.02685
H	2.10160	1.62561	2.76462
H	1.18110	2.11972	1.40139

H	2.72466	-1.14005	4.42998
H	1.14058	-1.81869	4.80116
H	1.45161	-0.10793	5.07031
H	-2.83608	5.68293	0.10439
H	-1.69759	6.31693	1.27592
H	-2.20080	8.22606	2.56986
H	-3.66142	10.18323	2.94463
H	-5.96838	10.21371	2.01629
H	-6.78157	8.27717	0.68549
H	-5.31125	6.33478	0.30067
H	-3.57176	4.12243	3.86783
H	-4.13715	5.70570	3.40113
H	1.19954	5.51708	4.77880
H	0.59522	7.19894	6.50652
H	-1.77674	7.90833	6.75111
H	-3.51468	6.95304	5.27436
H	-0.27217	6.29870	-3.65821
H	-2.30000	4.88064	-3.73922
H	0.46924	4.26249	1.40551
H	0.24718	6.74835	1.60272
H	1.56950	6.96320	0.45003
H	1.87629	6.22157	2.02270
H	2.89917	4.14541	1.03604
H	2.09346	3.30075	-0.30067
H	-3.40505	2.64143	-0.99130
H	-4.71539	4.74967	-1.16834
H	-5.46936	3.45663	-2.11246
H	-4.54473	4.72702	-2.92635
H	-2.29295	1.67358	-3.03877
H	-3.18411	2.81442	-4.04459
H	-4.06413	1.59844	-3.11218
H	-7.15693	1.85087	3.38143
H	-6.62957	1.08918	5.67106
H	-4.26253	0.95599	6.38379

H	-4.70310	2.77732	0.79067
H	-5.80183	4.71057	1.95181
H	-7.29490	3.77529	2.08296
H	-6.68824	4.23658	0.48993
H	-6.66296	1.82612	-0.30955
H	-7.40179	1.39522	1.23300
H	-5.94569	0.55577	0.69714
H	-1.36172	1.89012	4.28872
H	-2.06658	3.50714	6.06634
H	-0.79141	2.41400	6.62406
H	-2.46211	2.15207	7.13444
H	-1.76858	-0.55151	4.54934
H	-2.38220	-0.29126	6.19064
H	-0.68428	0.02957	5.82175
H	2.35926	-5.98435	-2.54727
H	3.75979	-5.88685	-1.50081
H	5.50204	-7.21483	-2.10498
H	6.80855	-8.47178	-3.78455
H	6.28471	-8.23518	-6.20431
H	4.42695	-6.74327	-6.91681
H	3.10695	-5.51696	-5.23325
H	4.50031	-2.44587	-2.95223
H	4.93425	-3.86324	-3.87105
H	7.18046	-4.19891	-3.42180
H	9.04448	-4.59285	-1.84555
H	8.64313	-4.49931	0.60765
H	6.36221	-3.99759	1.45903
H	4.51166	-3.57566	-0.11880
H	0.99482	-7.55138	1.56247
H	-0.47326	-8.79565	0.00958
H	-1.05043	-7.79639	-2.17666
H	2.48797	-4.23092	0.84072
H	3.96236	-6.24432	0.82640
H	3.05649	-6.92418	2.18438

H	3.97375	-5.43195	2.39851
H	1.92894	-4.23082	3.26853
H	0.93023	-5.67552	3.05147
H	0.44682	-4.15427	2.30290
H	0.16190	-4.50874	-3.39063
H	1.21996	-6.61239	-4.33207
H	-0.09014	-5.99514	-5.34902
H	-0.36128	-7.39010	-4.30397
H	-2.23778	-4.62082	-2.59543
H	-2.37526	-6.22751	-3.30227
H	-2.08989	-4.83562	-4.35155
H	2.18153	-0.03053	-6.15944
H	3.94299	1.59270	-5.54942
H	4.97611	1.46314	-3.30562
H	0.70428	-2.51009	-3.86554
H	2.46928	-3.57478	-5.26752
H	2.02323	-2.53873	-6.62813
H	0.86908	-3.73812	-6.02241
H	-0.43965	-0.40380	-4.77635
H	-0.92231	-1.91250	-5.57863
H	0.19914	-0.81004	-6.36789
H	4.27807	-1.14939	-0.80002
H	6.39813	-1.09723	-2.17361
H	6.58164	-0.42620	-0.55092
H	6.47245	0.65482	-1.94186
H	3.12046	0.97891	-0.22111
H	4.43377	1.90605	-0.95783
H	4.74005	0.94527	0.49595

216

TS6 without dispersion

C	5.06594	-7.48165	-3.04996
C	4.13778	-6.52106	-3.46430
C	3.84751	-6.42046	-4.82935
C	4.46888	-7.25597	-5.75503

C	5.38664	-8.21552	-5.32898
C	5.68162	-8.32710	-3.97169
C	3.42185	-5.68838	-2.42076
N	3.34521	-4.25089	-2.71682
C	4.63403	-3.67940	-3.12582
C	5.75331	-3.85785	-2.11762
C	5.62467	-3.41387	-0.79635
C	6.68601	-3.52787	0.09888
C	7.89546	-4.08784	-0.31353
C	8.03365	-4.53564	-1.62544
C	6.96758	-4.42420	-2.51756
C	2.34152	-3.43588	-2.22312
N	2.32126	-2.09553	-2.43418
C	2.71324	-1.36339	-3.58912
C	3.63884	-0.28556	-3.46306
C	3.82066	0.59699	-4.53256
C	3.15286	0.43519	-5.73769
C	2.31884	-0.66355	-5.89036
C	2.09150	-1.57402	-4.85415
C	4.52229	-0.08632	-2.23838
C	6.01180	-0.21975	-2.60018
C	1.20095	-2.76769	-5.16909
C	1.78637	-3.57851	-6.33689
Y	0.42510	-1.68958	-0.96677
C	-0.36476	-0.14241	-2.61259
O	-0.88539	0.43434	-3.49618
N	1.30549	-3.83930	-1.44349
C	0.96916	-5.12435	-0.92296
C	0.02256	-5.93882	-1.61112
C	-0.36019	-7.15986	-1.04754
C	0.13603	-7.59120	0.17535
C	1.03335	-6.78221	0.85717
C	1.46037	-5.55666	0.33626
C	-0.56391	-5.57013	-2.96753

C	0.09570	-6.37146	-4.10035
C	2.41280	-4.72273	1.17544
C	3.67339	-5.48970	1.59757
Al	0.96206	-0.30821	1.96559
C	1.44005	-0.83902	3.82035
C	2.56418	-0.81130	0.80247
Al	-2.42955	-1.38424	0.21364
C	-1.98795	-2.67463	-1.34325
C	-0.73730	-0.92176	1.13687
Y	-1.30082	1.35710	0.79869
C	-3.15343	0.35770	-0.68417
N	-1.79195	3.71248	0.37082
C	-1.37380	4.67668	-0.59304
C	-2.11978	4.79750	-1.80004
C	-1.69987	5.69889	-2.78196
C	-0.55329	6.46536	-2.62662
C	0.20127	6.30860	-1.47326
C	-0.17426	5.42533	-0.45536
C	-3.37658	3.98673	-2.07761
C	-4.62559	4.87628	-2.14917
C	0.77105	5.28782	0.72780
C	2.10665	4.67299	0.27577
N	-2.79220	2.61294	2.05915
C	-3.89245	2.21623	2.87450
C	-5.24061	2.26677	2.41530
C	-6.26227	1.78471	3.23940
C	-6.00781	1.23465	4.48694
C	-4.69437	1.16860	4.92647
C	-3.63297	1.64884	4.15411
C	-5.66585	2.82266	1.06480
C	-6.44263	1.78829	0.23298
C	-2.23994	1.54385	4.75169
C	-2.12175	2.30727	6.07953
C	0.41929	1.19257	-1.41683

C	0.88849	1.79833	2.08860
C	-2.58372	3.82827	1.45770
N	-3.09925	5.00425	1.97039
C	-3.45137	5.12474	3.38822
C	-2.37013	5.68776	4.29134
C	-2.73041	6.54911	5.33411
C	-1.77800	7.01737	6.23731
C	-0.44313	6.63752	6.10560
C	-0.07211	5.78386	5.06797
C	-1.02940	5.31113	4.17121
C	-2.87137	6.29799	1.29120
C	-3.82892	7.40588	1.67876
C	-5.12891	7.46035	1.16202
C	-5.98216	8.51456	1.47996
C	-5.54345	9.54319	2.31300
C	-4.24594	9.51125	2.81972
C	-3.39749	8.45124	2.50323
C	1.04353	6.61513	1.45003
C	-6.52087	4.08630	1.23353
C	-3.86253	-2.24869	1.26250
C	-3.24903	3.15749	-3.36376
C	-1.83460	0.07610	4.94431
C	-2.08521	-5.77225	-3.04034
C	1.69282	-4.17790	2.41678
C	4.29311	1.26297	-1.54492
C	-0.24656	-2.37125	-5.49217
H	-0.73187	4.63637	3.37423
H	2.64367	5.35853	-0.38887
H	1.11592	6.88422	-1.35644
H	1.16089	1.49990	-2.15956
H	-0.31771	2.01409	-1.43179
H	0.97580	1.15569	-0.46564
H	-0.85071	-1.75576	1.86147
H	-3.72446	1.09533	-0.10019

H	-2.62820	0.89747	-1.48709
H	-3.93466	-0.18757	-1.23031
H	-1.65541	-3.65643	-0.97451
H	-3.04051	-2.82731	-1.61852
H	-1.51789	-2.50425	-2.32537
H	-4.20305	-1.61193	2.08850
H	-3.52245	-3.19250	1.70943
H	-4.74693	-2.48805	0.65680
H	3.28938	-0.03272	1.08030
H	2.70327	-0.88386	-0.28653
H	2.96392	-1.74591	1.21756
H	0.28934	2.18339	2.92908
H	1.91816	1.81681	2.46991
H	0.91033	2.57978	1.31510
H	2.49553	-1.13725	3.88381
H	0.84921	-1.67945	4.20306
H	1.30892	-0.01264	4.53023
H	-2.94724	6.13494	0.21821
H	-1.85120	6.64305	1.48823
H	-2.38719	8.43291	2.90263
H	-3.88991	10.31342	3.46036
H	-6.20611	10.36896	2.55699
H	-6.98690	8.53804	1.06689
H	-5.47488	6.67462	0.49839
H	-3.75052	4.14687	3.76230
H	-4.33744	5.76243	3.45897
H	0.96510	5.47902	4.95815
H	0.30234	7.00399	6.80579
H	-2.07870	7.68476	7.04051
H	-3.76812	6.85870	5.43509
H	-0.24401	7.16287	-3.39991
H	-2.28674	5.79560	-3.69173
H	0.30585	4.60593	1.44653
H	0.13095	7.07043	1.84328

H	1.51852	7.34291	0.78376
H	1.72100	6.45409	2.29533
H	2.75310	4.46855	1.13646
H	1.95953	3.73893	-0.27475
H	-3.50804	3.29050	-1.24443
H	-4.76973	5.44673	-1.22746
H	-5.52250	4.26924	-2.31482
H	-4.55478	5.59449	-2.97359
H	-2.35779	2.52459	-3.35966
H	-3.19125	3.79982	-4.24957
H	-4.12424	2.50988	-3.48656
H	-7.28756	1.83354	2.88224
H	-6.81986	0.86062	5.10408
H	-4.47958	0.73799	5.90080
H	-4.76717	3.09741	0.50421
H	-5.99708	4.85141	1.81021
H	-7.45605	3.85733	1.75631
H	-6.78546	4.50801	0.25735
H	-6.61839	2.16801	-0.77964
H	-7.42180	1.57840	0.67643
H	-5.90845	0.83859	0.15513
H	-1.54290	2.00743	4.04603
H	-2.40251	3.35836	5.97218
H	-1.09002	2.27068	6.44600
H	-2.75982	1.86449	6.85232
H	-1.83128	-0.46945	3.99569
H	-2.52786	-0.43465	5.62187
H	-0.83334	0.00458	5.37801
H	2.41091	-6.07491	-2.31102
H	3.92297	-5.84131	-1.45723
H	5.31161	-7.56546	-1.99411
H	6.39827	-9.06805	-3.62788
H	5.86951	-8.86919	-6.05008
H	4.23232	-7.15844	-6.81122

H	3.13519	-5.67577	-5.16888
H	4.49186	-2.62243	-3.33773
H	4.93198	-4.14682	-4.06883
H	7.07665	-4.78505	-3.53744
H	8.96987	-4.97747	-1.95592
H	8.72349	-4.17447	0.38465
H	6.57044	-3.17338	1.11960
H	4.69008	-2.96649	-0.46882
H	1.41367	-7.10514	1.82274
H	-0.17919	-8.54278	0.59430
H	-1.06759	-7.78659	-1.58330
H	2.73214	-3.87131	0.56564
H	4.22521	-5.87981	0.73798
H	3.43158	-6.33424	2.25163
H	4.34811	-4.83069	2.15392
H	2.35585	-3.54083	3.00978
H	1.35142	-4.99748	3.05895
H	0.81206	-3.58648	2.14701
H	-0.35599	-4.50884	-3.13491
H	1.17267	-6.20257	-4.15821
H	-0.33938	-6.09711	-5.06797
H	-0.06191	-7.44656	-3.95741
H	-2.60496	-5.29879	-2.20474
H	-2.35176	-6.83489	-3.04445
H	-2.47393	-5.34122	-3.96937
H	1.81757	-0.81971	-6.84224
H	3.29850	1.13833	-6.55291
H	4.51744	1.42355	-4.41684
H	1.17746	-3.42132	-4.29351
H	2.84290	-3.81051	-6.17467
H	1.71849	-3.02270	-7.27834
H	1.23746	-4.51683	-6.47262
H	-0.71017	-1.81000	-4.67898
H	-0.85109	-3.26443	-5.68800

H	-0.29183	-1.74012	-6.38665
H	4.28211	-0.88131	-1.52655
H	6.23339	-1.16634	-3.09996
H	6.62788	-0.17082	-1.69590
H	6.33195	0.58953	-3.26572
H	3.25823	1.38632	-1.21902
H	4.53180	2.09723	-2.21406
H	4.93718	1.35390	-0.66309

216

Int9 without dispersion

C	2.94243	-7.86703	-3.48261
C	2.61524	-6.56651	-3.08247
C	1.52070	-5.93923	-3.68453
C	0.76960	-6.59555	-4.65873
C	1.09936	-7.89424	-5.04329
C	2.18935	-8.52940	-4.45018
C	3.39985	-5.91741	-1.95801
N	3.69188	-4.49176	-2.12802
C	4.53017	-4.19520	-3.30534
C	5.68010	-5.15673	-3.52296
C	6.77960	-5.19018	-2.65677
C	7.85697	-6.03826	-2.90132
C	7.85812	-6.86240	-4.02670
C	6.77517	-6.83090	-4.90237
C	5.69507	-5.98592	-4.64911
C	2.91267	-3.51453	-1.53042
N	2.78470	-2.23899	-1.94875
C	3.40206	-1.52912	-3.01268
C	4.57340	-0.76245	-2.74875
C	5.16120	-0.02327	-3.77875
C	4.61572	0.01274	-5.05520
C	3.44092	-0.68514	-5.29619
C	2.81415	-1.44578	-4.30316
C	5.22281	-0.70324	-1.37472

C	6.63762	-1.29896	-1.37152
C	1.49117	-2.10498	-4.65749
C	1.59388	-3.05852	-5.85577
Y	0.98988	-1.69811	-0.36267
C	0.49578	1.71344	-1.13497
O	0.70804	0.46406	-1.07876
N	2.12924	-3.76036	-0.43682
C	2.26992	-4.76740	0.55990
C	1.20134	-5.68479	0.76914
C	1.25364	-6.57626	1.84354
C	2.34028	-6.61426	2.70605
C	3.39658	-5.74201	2.48565
C	3.38722	-4.81321	1.43990
C	-0.00572	-5.74709	-0.15035
C	-0.26457	-7.16043	-0.69139
C	4.60115	-3.90760	1.30924
C	5.85039	-4.71533	0.92921
Al	0.72986	-0.38530	2.36091
C	1.01038	-1.09886	4.18628
C	2.54811	-0.58130	1.33392
Al	-2.05027	-1.48151	-0.20435
C	-1.05400	-2.34027	-1.83262
C	-0.73468	-1.08348	1.22417
Y	-1.18589	1.32451	0.63438
C	-2.67563	0.28257	-1.15575
N	-1.80594	3.67831	0.55461
C	-1.28328	4.76452	-0.19143
C	-1.79361	5.00164	-1.50140
C	-1.24810	6.02257	-2.28408
C	-0.19441	6.80559	-1.83107
C	0.33567	6.54340	-0.57585
C	-0.17401	5.53608	0.25108
C	-2.92756	4.18130	-2.09834
C	-4.17836	5.03313	-2.35505

C	0.53183	5.30297	1.57844
C	1.98477	4.85138	1.35560
N	-3.04507	2.27663	1.78381
C	-4.24262	1.72737	2.31525
C	-5.50459	1.87503	1.66694
C	-6.64766	1.32225	2.25235
C	-6.59500	0.60443	3.43756
C	-5.35795	0.39831	4.03041
C	-4.18014	0.92113	3.48868
C	-5.70086	2.58033	0.33346
C	-6.20993	1.60616	-0.74249
C	-2.88125	0.59437	4.20819
C	-2.72888	1.39153	5.51116
C	1.32121	2.48325	-2.11315
C	0.64374	1.69190	2.46267
C	-2.81443	3.57843	1.44377
N	-3.51166	4.64222	1.99783
C	-4.16275	4.53568	3.30436
C	-3.38191	5.08385	4.48364
C	-4.08187	5.64246	5.55971
C	-3.41134	6.08485	6.69733
C	-2.02221	5.98563	6.77417
C	-1.31473	5.43657	5.70676
C	-1.99103	4.98637	4.57246
C	-3.24629	6.02629	1.55502
C	-4.33436	7.02998	1.87848
C	-5.49060	7.12795	1.09535
C	-6.45908	8.09256	1.36422
C	-6.28060	8.98809	2.41797
C	-5.12583	8.91446	3.19416
C	-4.16200	7.94417	2.92419
C	0.53227	6.54137	2.48714
C	-6.66989	3.76414	0.44939
C	-3.70217	-2.45829	0.26874

C	-2.50575	3.47285	-3.39366
C	-2.75823	-0.91033	4.49002
C	-1.25905	-5.20750	0.54902
C	4.87302	-3.11164	2.59590
C	5.26440	0.73567	-0.83808
C	0.42061	-1.03727	-4.93937
H	-1.43291	4.55131	3.74884
H	2.57899	5.65194	0.90111
H	1.17586	7.13623	-0.22243
H	1.93878	1.84879	-2.75865
H	0.67101	3.14005	-2.70110
H	1.95967	3.16858	-1.54013
H	-1.25147	-1.63712	2.02950
H	-3.37905	0.97195	-0.66187
H	-2.01094	0.88216	-1.80047
H	-3.31184	-0.25256	-1.87214
H	-0.53585	-3.31135	-1.77240
H	-1.98648	-2.59104	-2.35554
H	-0.51753	-1.71048	-2.55786
H	-4.39155	-1.79004	0.80149
H	-3.52939	-3.31976	0.92316
H	-4.24203	-2.82955	-0.61322
H	3.11445	-0.13588	2.16134
H	2.79285	0.05996	0.47437
H	3.05455	-1.54708	1.18229
H	-0.20523	2.17551	2.97382
H	1.46355	1.77882	3.18791
H	0.96359	2.35396	1.64265
H	2.06212	-1.00406	4.49019
H	0.75508	-2.16311	4.26238
H	0.42156	-0.57244	4.94754
H	-3.09891	6.01358	0.47670
H	-2.31090	6.38566	1.99671
H	-3.26442	7.89295	3.53411

H	-4.97105	9.61441	4.01073
H	-7.03248	9.74453	2.62567
H	-7.34844	8.15143	0.74264
H	-5.63147	6.44769	0.26170
H	-4.39443	3.48932	3.49748
H	-5.12483	5.05546	3.24966
H	-0.23252	5.35267	5.75643
H	-1.49596	6.33429	7.65839
H	-3.97380	6.51419	7.52219
H	-5.16405	5.73496	5.50085
H	0.21366	7.59944	-2.45048
H	-1.66047	6.20507	-3.27364
H	0.00447	4.49460	2.09482
H	-0.47701	6.88081	2.73270
H	1.05784	7.37895	2.01610
H	1.04555	6.32171	3.42970
H	2.45831	4.58382	2.30655
H	2.04289	3.98392	0.69261
H	-3.18813	3.40717	-1.37128
H	-4.52369	5.52624	-1.44248
H	-4.99765	4.41327	-2.73570
H	-3.98197	5.81539	-3.09680
H	-1.62891	2.83838	-3.23677
H	-2.25916	4.19081	-4.18386
H	-3.31774	2.83794	-3.76485
H	-7.60515	1.45282	1.75446
H	-7.49851	0.19353	3.87933
H	-5.29849	-0.18917	4.94215
H	-4.73378	2.96923	0.00274
H	-6.34695	4.47468	1.21283
H	-7.67724	3.42540	0.71507
H	-6.74778	4.29466	-0.50633
H	-6.25251	2.10071	-1.71979
H	-7.22051	1.25489	-0.50732

H	-5.57043	0.72482	-0.83166
H	-2.05495	0.87660	3.54777
H	-2.74670	2.46979	5.33496
H	-1.78183	1.14519	6.00494
H	-3.53987	1.15241	6.20892
H	-2.94267	-1.50163	3.58893
H	-3.47080	-1.23635	5.25558
H	-1.75677	-1.14938	4.85763
H	2.86106	-6.05783	-1.02276
H	4.35126	-6.44620	-1.84439
H	3.79935	-8.36307	-3.03266
H	2.45925	-9.53982	-4.74547
H	0.51240	-8.40623	-5.80073
H	-0.07774	-6.09145	-5.11545
H	1.25117	-4.93020	-3.38603
H	4.93600	-3.19237	-3.19169
H	3.91301	-4.18595	-4.21132
H	4.85015	-5.97118	-5.33242
H	6.76751	-7.46535	-5.78448
H	8.70055	-7.52041	-4.22129
H	8.70081	-6.04887	-2.21673
H	6.79217	-4.54645	-1.78321
H	4.25450	-5.77055	3.15253
H	2.36624	-7.31632	3.53477
H	0.42452	-7.26150	1.99900
H	4.40799	-3.19221	0.50334
H	5.68761	-5.30006	0.02105
H	6.12157	-5.41389	1.72863
H	6.70771	-4.05190	0.76662
H	5.66755	-2.37549	2.42961
H	5.20398	-3.77009	3.40616
H	3.98288	-2.58357	2.94633
H	0.21403	-5.10497	-1.00957
H	0.61641	-7.57291	-1.19074

H	-1.08275	-7.14008	-1.41936
H	-0.55598	-7.85208	0.10661
H	-1.10760	-4.19135	0.92782
H	-1.52667	-5.83679	1.40573
H	-2.11410	-5.19157	-0.13488
H	2.98945	-0.63759	-6.28406
H	5.09023	0.58833	-5.84505
H	6.06753	0.54015	-3.57067
H	1.16757	-2.68745	-3.78846
H	2.30963	-3.86588	-5.68030
H	1.90352	-2.52873	-6.76294
H	0.62058	-3.51702	-6.06214
H	0.33347	-0.32612	-4.11229
H	-0.55927	-1.49983	-5.10207
H	0.67063	-0.46267	-5.83815
H	4.60358	-1.29520	-0.69496
H	6.64547	-2.33143	-1.73043
H	7.05800	-1.28853	-0.35966
H	7.31293	-0.72444	-2.01538
H	4.27495	1.20137	-0.85966
H	5.93624	1.36401	-1.43353
H	5.62635	0.75097	0.19576

References

[1] *SAINTPlus Data Reduction and Correction Program* v. 6.02a, Bruker AXS, Madison, WI, 2000.

[2] G. M. Sheldrick, *SADABS*, A Program for Empirical Absorption Correction, University of Göttingen, Göttingen, Germany, 1998.

[3] G. M. Sheldrick, *SHELXL-97*, Program for the Refinement of Crystal Structures, University of Göttingen, Göttingen, Germany, 1997.

[4] Gaussian 09, Revision D.01, M. J. Frisch, G. W. Trucks, H. B. Schlegel, G. E. Scuseria, M. A. Robb, J. R. Cheeseman, G. Scalmani, V. Barone, G. A. Petersson, H. Nakatsuji, X. Li, M. Caricato, A. Marenich, J. Bloino, B. G. Janesko, R. Gomperts, B. Mennucci, H. P. Hratchian, J. V. Ortiz, A. F. Izmaylov, J. L. Sonnenberg, D. Williams-Young, F. Ding, F. Lipparini, F. Egidi, J. Goings, B. Peng, A. Petrone, T. Henderson, D. Ranasinghe, V. G. Zakrzewski, J. Gao, N. Rega, G. Zheng, W. Liang, M. Hada, M. Ehara, K. Toyota, R. Fukuda, J. Hasegawa, M. Ishida, T. Nakajima, Y. Honda, O. Kitao, H. Nakai, T. Vreven, K. Throssell, J. A. Montgomery, J. J. E. Peralta, F. Ogliaro, M. Bearpark, J. J. Heyd, E. Brothers, K. N. Kudin, V. N. Staroverov, T. Keith, R. Kobayashi, J. Normand, K. Raghavachari, A. Rendell, J. C. Burant, S. S. Iyengar, J. Tomasi, M. Cossi, J. M. Millam, M. Klene, C. Adamo, R. Cammi, J. W. Ochterski, R. L. Martin, K. Morokuma, O. Farkas, J. B. Foresman and D. J. Fox, Gaussian, Inc., Wallingford CT, 2016.

[5] A. D. J. Becke, *J. Chem. Phys.,* 1993, **98,** 5648−5652 and references therein.

[6] (a) D. Andrae, U. Haeussermann, M. Dolg, H. Stoll and H. Preuss, *Theor. Chim. Acta*., 1990, **77,** 123−141; (b) J. M. L. Martin and A. Sundermann, *J. Chem. Phys.* 2001, **114,** 3408−3420.

[7] A. Bergner, M. Dolg, W. Kuechle, H. Stoll and H. Preuss, *Mol. Phys.,* 1993, **80,** 1431−1441.

[8] (a) P. C. Hariharan and J. A. Pople, *Theor. Chem. Acc.,* 1973, **28,** 213−222; (b) W. J. Hehre, R. Ditchfield and J. A. Pople, *J. Chem. Phys.,* 1972, **56,** 2257−2261.
